# Identification of specific biomarkers for gastric adenocarcinoma by ITRAQ proteomic approach

**DOI:** 10.1038/srep38871

**Published:** 2016-12-12

**Authors:** Xiaoxiao Wang, Qiaoming Zhi, Songbai Liu, Sheng-Li Xue, Congcong Shen, Yangxin Li, Chaofan Wu, Zaixiang Tang, Weichang Chen, Jenny Lee Song, Meiyu Bao, Yao-Hua Song, Jin Zhou

**Affiliations:** 1 Cyrus Tang Hematology Center, Collaborative Innovation Center of Hematology, Jiangsu Institute of Hematology, First Affiliated Hospital, Soochow University, Suzhou, China; 2 Department of General Surgery, the First Affiliated Hospital of Soochow University, Suzhou, China; 3 Suzhou Vocational Health College, Suzhou Key Laboratory of Biotechnology for Laboratory Medicine, Suzhou 215009, Jiangsu Province, China; 4 Department of Hematology, the First Affiliated Hospital of Soochow University, Jiangsu Institute of Hematology, the First Affiliated Hospital of Soochow University; Collaborative Innovation Center of Hematology, Soochow University, Suzhou, China; 5 Department of Cardiovascular Surgery & Institute of Cardiovascular Science, First Affiliated Hospital of Soochow University, Suzhou, Jiangsu 215123, P. R. China; 6 Department of Biostatistics, School of Public Health, Medical College of Soochow University, China; 7 Department of Gastroenterology, the First Affiliated Hospital of Soochow University, China

## Abstract

The aim of this study was to identify biomarkers for gastric cancer (GC) by iTRAQ. Using proteins extracted from a panel of 4 pairs of gastric adenocarcinoma samples (stage III-IV, Her-2 negative), we identified 10 up regulated and 9 down regulated proteins in all four pairs of GC samples compared to adjacent normal gastric tissue. The up regulated proteins are mainly involved in cell motility, while the down regulated proteins are mitochondrial enzymes involved in energy metabolism. The expression of three up regulated proteins (ANXA1, NNMT, fibulin-5) and one of the down regulated proteins (UQCRC1) was validated by Western Blot in 97 GC samples. ANXA1 was up regulated in 61.36% of stage I/II GC samples compared to matched adjacent normal gastric tissue, and its expression increased further in stage III/IV samples. Knockdown of ANXA1 by siRNA significantly inhibited GC cell migration and invasion, whereas over expression of ANXA1 promoted migration and invasion. We found decreased expression of UQCRC1 in all stages of GC samples. Our data suggest that increased cell motility and decreased mitochondrial energy metabolism are important hallmarks during the development of GC.

Gastric cancer is a serious threat to public health worldwide. It was estimated that about 10,000 Americans will die from gastric cancer in 2016^1^. Although stage I GC may be curable by surgery alone, many patients with advanced GCs die due to recurrence of the disease after initial tumor resection and failure to response to subsequent chemotherapy^2^,^3^. Chemotherapy failure results from genetic mutations, epigenetic alterations of DNA and post-translational modification of proteins. These changes result in aberrant expression of certain proteins, which leads to altered cellular metabolism, proliferation and metastatic potential. In order to improve outcomes, it is crucial to identify proteins that can be used as markers for early diagnosis and treatment. Several biomarkers, including Her-2^4^, EGFR^5^, VEGF^6^, and HGF/MET^7^, have been identified in the past. Among these markers, only Her-2 inhibition has led to clinical benefit^4^. However, only 8–18% of GC tumors are Her-2 positive^8^,^9^. Therefore, a new comprehensive molecular classification from proteomic study on Her-2 negative GCs may bring new therapeutic strategies into clinical practice in the near future.

iTRAQ (isobaric tags for relative and absolute quantitation) is one of the new techniques used in modern proteomics that couples stable isotopes labeling and tandem mass spectrometry to compare the relative amount of proteins from normal and diseased samples in a single experiment. iTRAQ-based quantitative proteomics have revealed myoferlin as a novel prognostic predictor in pancreatic adenocarcinoma^10^. This present study utilized the iTRAQ approach to profile the differentially expressed proteins in Her-2 negative stage III–IV human gastric adenocarcinoma. We performed Gene Ontology (GO) and pathway analyses in order to explore the role of dysregulated proteins in tumor progression. Selected differentially expressed proteins were validated by Western Blot and immunofluorecence staining. We also performed migration and invasion experiments using a human gastric cancer line in order to exam the role of these proteins in tumor metastasis.

## Results

### Detection and quantification of proteins in gastric cancer

Four pairs of gastric adenocarcinoma samples (stage III-IV, Her-2 negative) were used for this study to identify differentially expressed proteins involved in the development and progression of gastric cancer (Table 1). Both tumor (A) and adjacent normal gastric tissue (B) were taken from each patient during surgical resection. Proteins from samples 31A, 31B, 10A, 10B, 5A, 5B, 101A, 101B were labeled with ITRAQ reagents 113,114,115,116,117,118,119,121, respectively. Thus, the ratio of labels 113 and 114 would indicate the relative abundance of proteins from sample 31A/31B, etc. Data was analyzed by Protein Pilot Software v. 5.0 (AB SCIEX, USA) using the Paragon algorithm based on Homo sapiens data base^11^. An automatic decoy database search strategy was used to determine the false discovery rate (FDR) using the Proteomics System Performance Evaluation Pipeline Software (PSPEP). The FDR was calculated as the false positive matches divided by the total matches. A total of 3245 proteins with the value of global FDR from fit less than 1% were detected. In order to reduce false positives, we included an additional 1.3-fold and a 0.77-fold cutoff for up or down regulated proteins, respectively. Based on this selection, 120 proteins were found differentially expressed in the gastric tumors compared to their adjacent normal gastric tissues. Nineteen proteins were selected for further analysis because these proteins are either up or down regulated in all 4 pairs of samples (Tables 2 and 3).

### Classification of differentially expressed proteins

The functional classification of the 19 proteins was cataloged according to biological processes (BPs), molecular functions (MFs), and cellular components (CCs) according to the GO database. The proteins under the category of BPs are involved in tricarboxylic acid cycle (15%), isocitrate metabolic process (10%), response to activity (10%), small molecule metabolic process (42%), and oxidation reduction process (10%). The proteins under the category of MFs are involved in calcium ion binding (31%), NAD binding (10%), integrin binding (10%), and protein complex binding (10%). The proteins under the category of CCs were classified as mitochondrial respiratory chain (10%), mitochondrial inner membrane (26%), mitochondrial matrix (20%), extracellular matrix (15%), extracellular vesicular exosome (10%), proteinaceous extracellular matrix (15%), and neuromuscular junction (10%).

**Figure 1:**
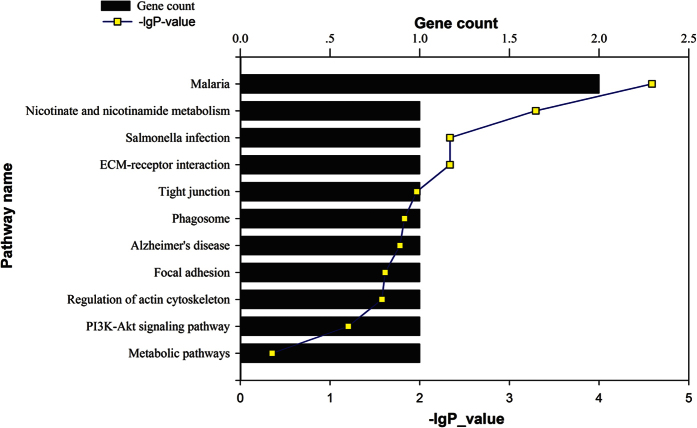
Pathway analysis of up regulated proteins based on KEGG. The up regulated proteins were mapped to KEGG pathways based on their gene ID. The top pathways include regulation of actin cytoskeleton, tight junction, focal adhesion and PI3K-Akt signaling pathway.

**Figure 2:**
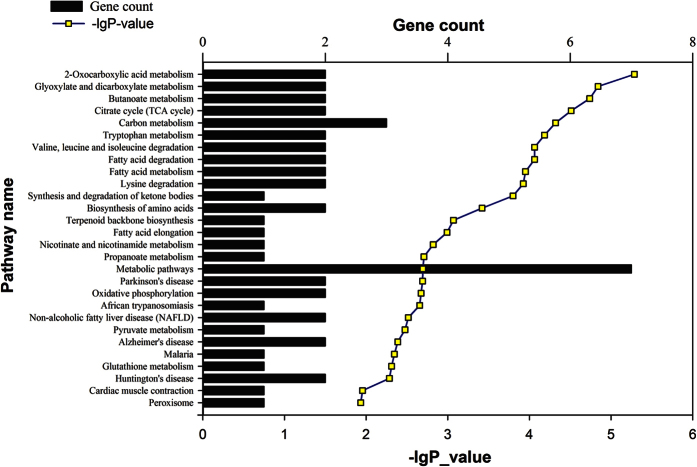
Pathway analysis of down regulated proteins based on KEGG. The down regulated proteins were mapped to KEGG pathways based on their gene ID. The top pathways include metabolic pathways and carbon metabolism.

The differentially expressed proteins were mapped to KEGG pathways based on their gene ID. The up regulated proteins are involved in the following pathways: malaria, nicotinate and nicotinamide metabolism, salmonella infection, ECM-receptor interaction, tight junction, phagosome, Alzheimer’s disease, focal adhesion, regulation of actin cytoskeleton, PI3K-Akt signaling pathway and metabolic pathways (Fig. 1). The down regulated proteins are involved in the following pathways: 2-oxocarboxylate acid metabolism, glyoxylate and dicarboxylate metabolism, butanoate metabolism, citrate cycle, carbon metabolism, tryptophan metabolism, valine leucine and isoleucine degradation, fatty acid degradation, fatty acid metabolism, lysine degradation, synthesis and degradation of ketone bodies, biosynthesis of amino acids, terpenoid backbone biosynthesis, fatty acid elongation, nicotinate and nicotinamide metabolism, propanoate metabolism, metabolic pathways, Parkinson’s disease, oxidative phosphorylation, African trypanosomiasis, non-alcoholic fatty liver disease, pyruvate metabolism, Alzheimer’s disease, malaria, glutathione metabolism, Huntington’s disease, cardiac muscle contraction and peroxisome (Fig. 2).

**Figure 3:**
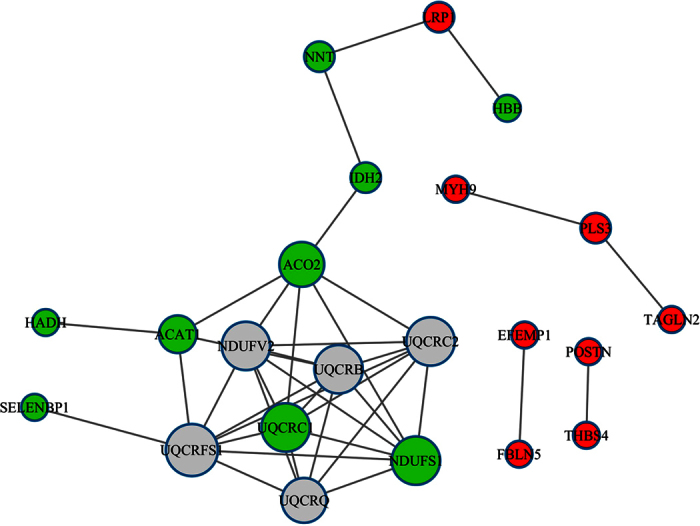
Functional protein interaction networks. The network of interactions of the 19 differentially expressed proteins between gastric cancer and adjacent normal gastric tissue was predicted using the String data base. Red: up regulated proteins; green: down regulated proteins; grey: other related proteins.

Figure 3 shows a network of interactions among the 19 proteins using the String data base (http://string-db.org), which is helpful in dissecting out groups of proteins involved in a particular pathway. For example, it is obvious from the network that myosin-9 (MYH9), plastin-3 (PLS3) and transgelin-2 (TAGLN2) are linked together because all of the three proteins are involved in actin binding and cell motility. Previous studies showed that MYH9 promotes cancer cell migration and invasion and were up regulated in non-small cell lung cancer (NSCLC)^12^ and esophageal squamous cell carcinoma (ESCC)^13^. PLS3 has been identified as a novel marker for circulating tumor cells in colorectal cancer (CRC)^14^. Over expression of PLS3 induced epithelial mesenchymal transition in CRC, which showed enhanced migration and invasion ability^14^. MYH9 and PLS3 have never been implicated in gastric cancer. TAGLN2 promote tumor cell migration and invasion^15^ and is highly expressed in human pancreatic cancer^16^ and gastric cancer^17^.

Among the down regulated proteins, 7 of 9 (77.7%) proteins are located within mitochondria (Table 3), which are interconnected as shown in Fig. 3. All of these proteins are enzymes involved in energy metabolism.

Mitochondrial dysfunction and deregulated cellular energy metabolism is one of the hallmarks during cancer development. Deregulated energy metabolism can be caused by either mitochondrial DNA mutations or mitochondrial enzyme defects, which could result in altered signal transduction pathways that eventually modify gene expression. Indeed, our results show that isocitrate dehydrogenase (IDH2), an enzyme in the citric acid cycle, is down regulated in advanced gastric cancer. The main function of IDH2 is to catalyze the oxidative decarboxylation of isocitrate into alpha-ketoglutarate. Defect in IDH2 can cause accumulation of 2-hydroxyglutarate, which renders cells more accommodating for epigenetic modification and potential transformation due to DNA hypermethylation^18^,^19^,^20^,^21^. IDH2 has been considered a tumor suppressor because its loss was associated with progression of GC via NF-κB-dependent increases in MMP7 activity^22^.

ACO2 is another enzyme of the citric acid cycle that is down regulated in stage III/IV gastric cancer (Table 3). Our results are consistent with a previous report that expression of ACO2 was significantly down-regulated in gastric cancer tissues compared with matched adjacent cancer tissues, and reduced expression of ACO2 was associated with clinical stage and pathological differentiation states^23^.

Our data revealed the down regulation of three proteins involved in electron transfer and respiration (NNT, NDUFS1, UQCRC1), and two proteins involved in fatty acid metabolism and ketone body formation (ACAT1, HADH) in stage III/IV GC. NNT is a mitochondrial enzyme that produces NADPH, and this enzyme has not been implicated in cancer so far in the literature. NDUFS1 belongs to the complex I, located at the mitochondrial inner membrane. This protein transfers electrons from NADH to the respiratory chain. NDUFS1 has been identified as candidate gene in ovarian cancer^24^. UQCRC1 is a component of the cytochrome b-c1 complex (complex III), which is part of the mitochondrial respiratory chain. The expression of UQCRC1 is increased in osteosarcoma cells^25^. ACAT1 catalyzes the reversible formation of acetoacetyl-CoA from two molecules of acetyl-CoA. It has been shown that ACAT1 expression was significantly greater in prostate cancer compared to normal prostate tissue^26^. HADH is involved in fatty acid oxidation and has not been implicated in cancer development.

### Validation of iTRAQ results on selected proteins

**Figure 4:**
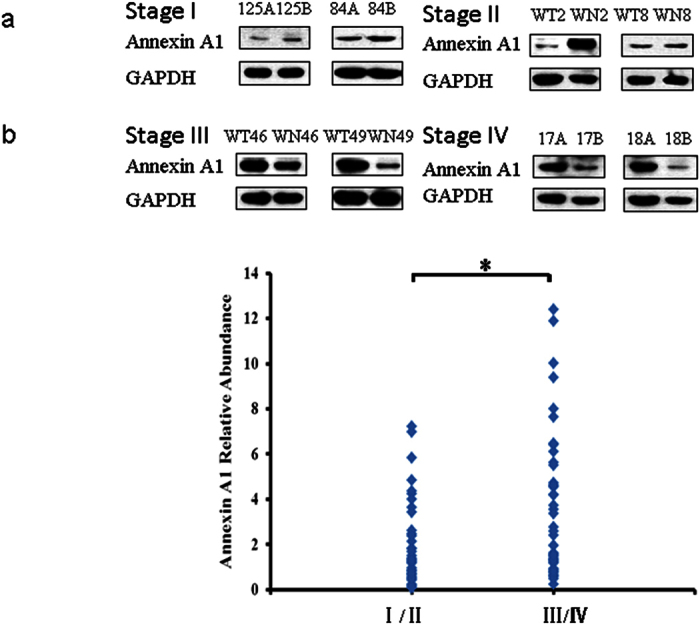
Validation of iTRAQ results by Western Blot. Annexin A1 expression was analyzed in 97 pairs of GC samples and matched adjacent normal gastric tissues by Western Blot. (**a**) Representative Western Blot image of Annexin A1 expression in stages I-IV GC samples and their matched adjacent normal gastric tissue (A or WT. cancer, B or WN. normal gastric tissue). The blots were cropped and full length blots are presented in Supplementary Figure S4. (**b**) Annexin A1 relative abundance in 97 GC samples. The bands were quantified by ImageJ and normalized to GAPDH, and the normalized density from GC samples was then divided by that of their corresponding adjacent normal gastric tissues. *P < 0.05.

**Figure 5:**
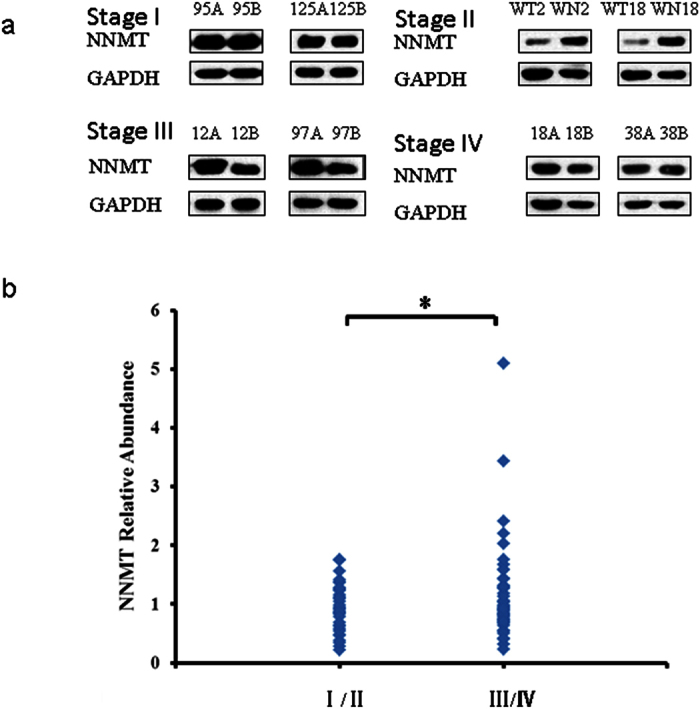
Validation of NNMT expression by Western Blot. (**a**) Representative Western Blot image of NNMT expression in stages I–IV GC samples (A or WT. cancer, B or WN. normal gastric tissue). The blots were cropped and full length blots are presented in Supplementary Figure S4. (**b**) NNMT relative abundance in 97 GC samples. *P < 0.05.

**Figure 6:**
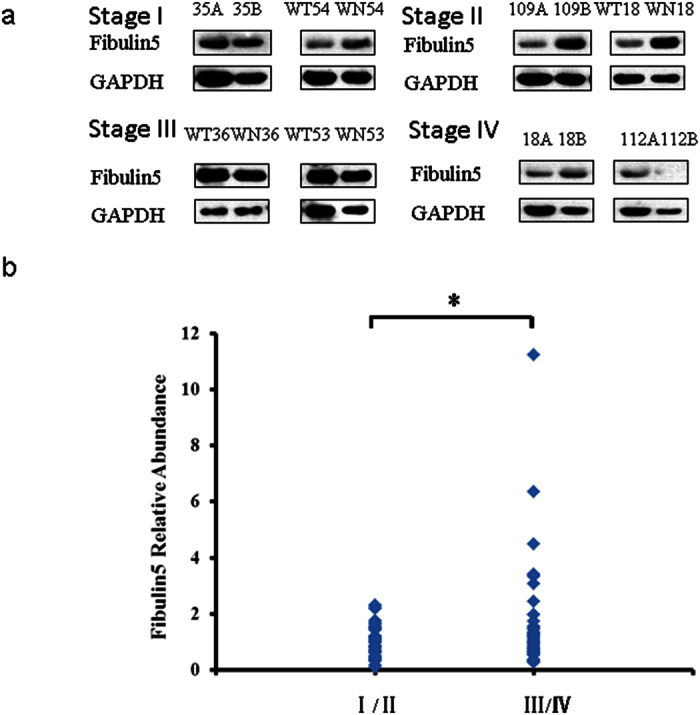
Validation of Fibulin-5 expression by Western Blot. (**a**) Representative Western Blot image of Fibulin-5 expression in stages I–IV GC samples (A or WT. cancer, B or WN. normal gastric tissue). The blots were cropped image and full length blots are presented in Supplementary Figure S4. (**b**) Fibulin-5 relative abundance in 97 GC samples. *P < 0.05.

To confirm the iTRAQ results, three up regulated proteins, namely, Nicotinamide -N-methyltransferase (NNMT), Fibulin-5 (FBLN5), Annexin A1 (ANXA1) and one of the down regulated proteins, cytochrome b-c1 complex subunit 1 (UQCRC1) were chosen for further analysis by Western Blot and immunofluorescence assay. The GC samples used for validation comprised 70 males and 27 female (Table 4). Western Blot analysis revealed that NNMT, FBLN5 and ANXA1 are clearly up regulated in stages III-IV GC (Fig. 4Fig. 5 and Fig. 6) compared to stage I-II samples. NNMT, FBLN5 and ANXA1 were up regulated in 45.45%, 47.73%, and 61.36% of stage I/II GC samples, respectively. Our data suggest that ANXA1 is a better marker for early diagnosis of GC.

**Figure 7:**
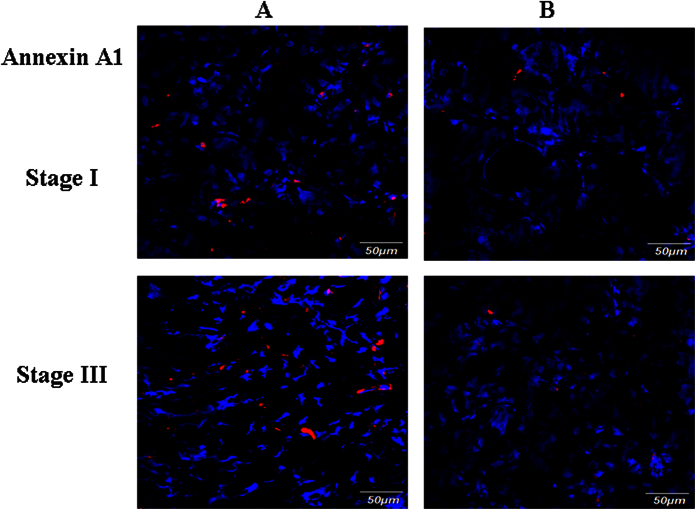
Validation of Annexin A1 expression by immunofluorescence staining. Frozen sections from stage I and III GC (**A**) and their adjacent normal gastric tissues (**B**) were incubated with antibody against human Annexin A1, followed by goat anti-rabbit IgG - Alexa Fluor® 568 conjugate. Nuclei are stained with DAPI (blue).

**Figure 8:**
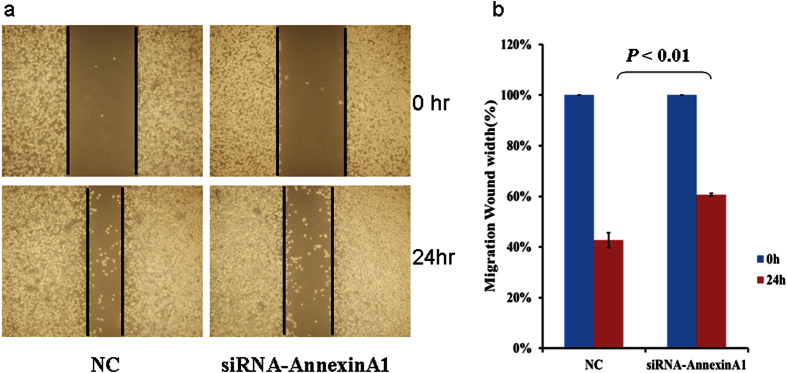
Knockdown of Annexin A1 by siRNA inhibits AGS cell migration. AGS cells were transfected with either control (NC) or Annexin A1 siRNA for 6 hours and migration was assessed by wound healing assay using 6-well plates. (**a**) Representative images of wound healing assay. (**b**) Bar graph showing migration as percentage of AGS cell migration at 0 hour.

**Figure 9:**
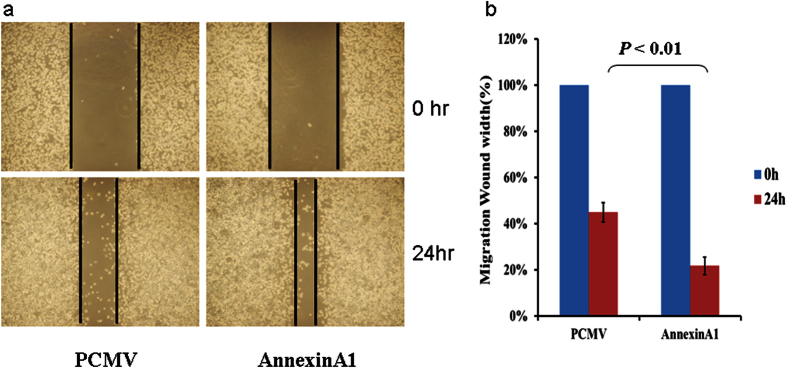
Annexin A1 overexpression in AGS cells promote migration. AGS cells were transfected with either control (PCMV) or an expression plasmid carrying Annexin A1 cDNA for 6 hours and migration was assessed by wound healing assay using 6-well plates. (**a**) Representative images of wound healing assay. (**b**) Bar graph showing migration as percentage of AGS cell migration at 0 hour.

Because of the contradicting reports in the literature regarding the role of ANXA1 in gastric cancer^27^,^28^, we investigated ANXA1 further by examining the expression of ANXA1 on frozen tissue sections by immunofluorescence technique, which confirmed that ANXA1 expression was increased in GC compared to adjacent normal gastric tissue (Fig. 7). In order to investigate the functional role of ANXA1 in GC progression and metastasis, we assessed the impact of ANXA1 knockdown and over expression on gastric cancer cell migration and invasion. Cancer cell migration was assessed using a wound healing assay, which revealed a decreased migration capacity in siRNA-Annexin A1 transfected AGS cells compared to controls (Fig. 8), but increased migration in cells transfected with a plasmid overexpressing Annexin A1 (Fig. 9).

**Figure 10:**
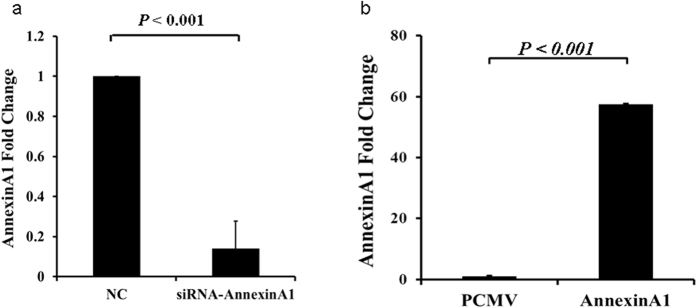
Realtime PCR analysis of Annexin A1 expression. (**a**) Annexin A1 mRNA level was reduced in AGS cells transfected with Annexin A1 siRNA compared to NC. (**b**) Annexin A1 mRNA level increased in AGS cells transfected with Annexin A1 expression plasmid compared to cells transfected with vector alone.

Real-time PCR results confirmed that Annexin A1 mRNA expression was significantly reduced in AGS cells transfected with siRNA (Fig. 10a). whereas its expression was up regulated in cells that were transfected with an expression plasmid carrying Annexin A1 cDNA (Fig. 10b).

**Figure 11:**
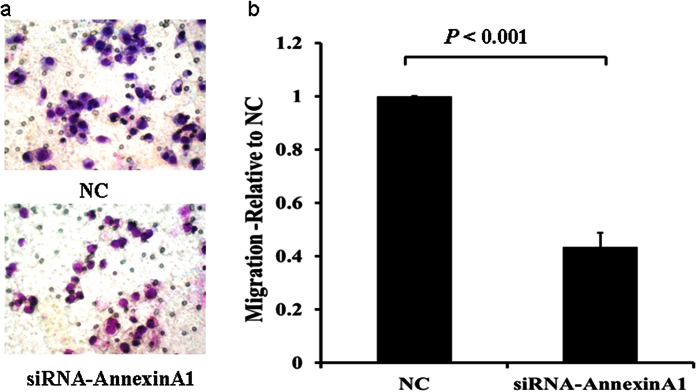
Knockdown of Annexin A1 by siRNA inhibits AGS cell migration. Migration assay was conducted with Annexin A1 siRNA or NC transfected AGS cells using 24-well Transwell chambers. (**a**) Representative images of migrated cells in NC and siRNA transfected cells. (**b**) Cell migration was assessed by counting the number of AGS cells that migrated through the transwell insert by light microscopy on 3 independent membranes, then normalized against the NC treated cells to determine the relative ratio.

**Figure 12:**
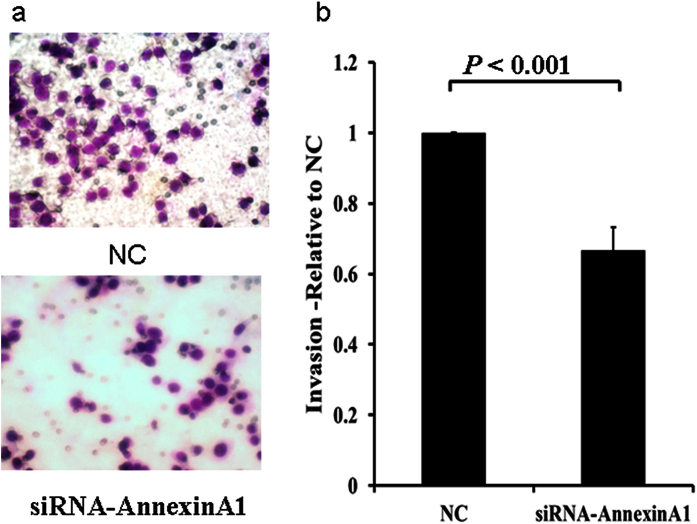
Knockdown of Annexin A1 by siRNA inhibits AGS cell invasion. Invasion assay was conducted with Annexin A1 siRNA or NC transfected AGS cells using 24-well Transwell chambers. (**a**) Representative images of invaded cells in NC and siRNA transfected cells. (**b**) Cell invasion was assessed by counting the number of AGS cells that invaded through the transwell insert by light microscopy on 3 independent membranes, then normalized against the NC treated cells to determine the relative ratio. The procedures for cell invasion are similar to that of migration except that matrigel was added to the upper chamber of the transwell.

**Figure 13:**
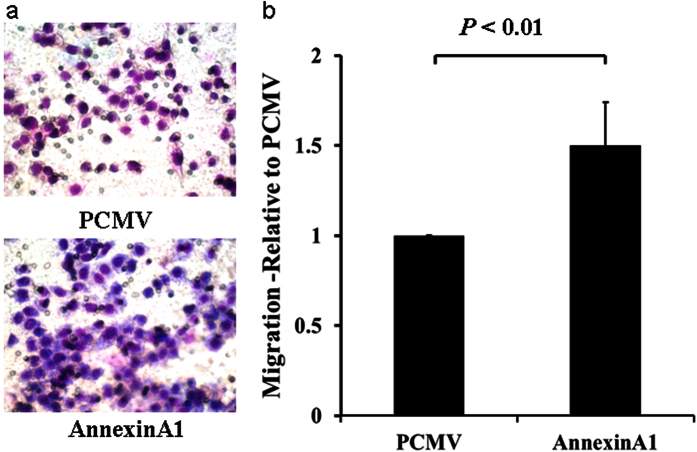
Overexpression of Annexin A1 in AGS cell promotes migration. Migration assay was conducted in AGS cells transfected with an expression plasmid carrying Annexin A1 cDNA or the empty vector PCMV using 24-well Transwell chambers. (**a**) Representative images of migrated cells in PCMV and Annexin A1 plasmid transfected cells. (**b**) Cell migration was assessed by counting the number of AGS cells that migrated through the transwell insert by light microscopy on 3 independent membranes, then normalized against the NC treated cells to determine the relative ratio.

**Figure 14:**
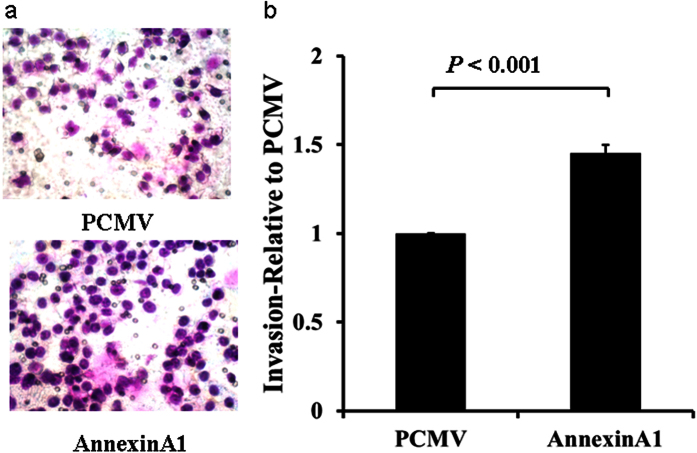
Overexpression of Annexin A1 in AGS cell promotes invasion. Invasion assay was conducted in AGS cells transfected with an expression plasmid carrying Annexin A1 cDNA or the empty vector PCMV using 24-well Transwell chambers. (**a**) Representative images of invaded cells in PCMV and Annexin A1 plasmid transfected cells. (**b**) Cell invasion was assessed by counting the number of AGS cells that invaded through the transwell insert by light microscopy on 3 independent membranes, then normalized against the NC treated cells to determine the relative ratio.

We then performed migration and invasion assays using transwell plates. Consistent with the findings from wound healing assay, migration and invasion were inhibited in AGS cells transfected with siRNA specific for ANXA1 (Figs 11 and 12), but increased in cells transfected with an expression plasmid carrying ANXA1 (Figs 13 and 14).

**Figure 15:**
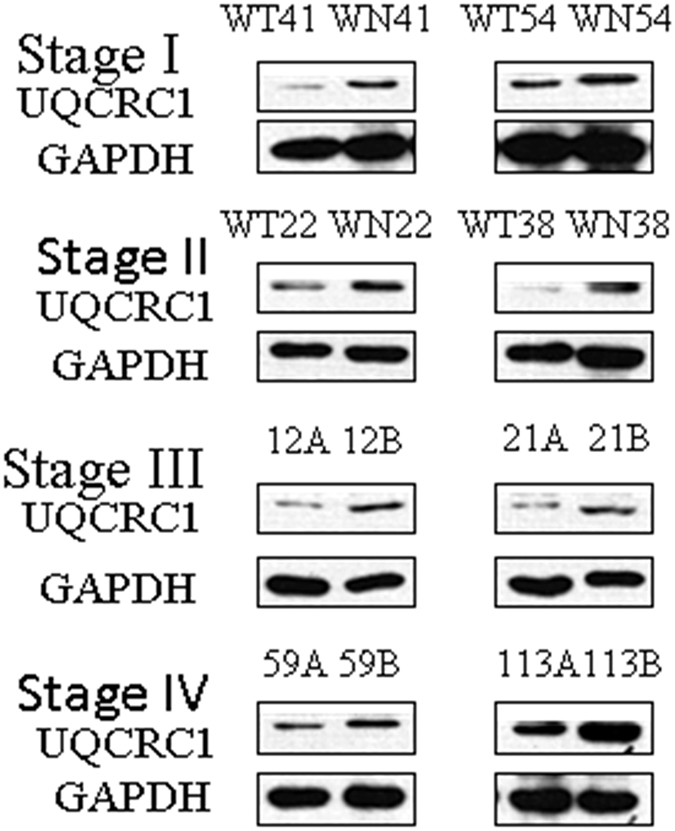
Validation of UQCRC1 expression by Western Blot. Representative Western Blot images of UQCRC1 expression in stages I–IV GC samples (A or WT. cancer, B or WN. normal gastric tissue). The blots were cropped and full length blots are presented in Supplementary Figure S4.

**Figure 16:**
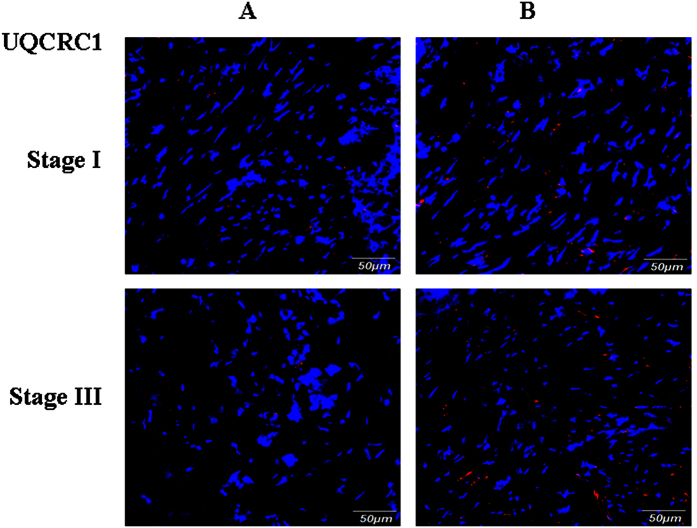
Validation of UQCRC1 expression by immunofluorescence staining. Frozen sections from stage I and III GC (**A**) and their adjacent normal gastric tissues (**B**) were incubated with antibody against human UQCRC1, followed by goat anti-rabbit IgG–Alexa Fluor® 568 conjugate. Nuclei are stained with DAPI (blue).

**Figure 17:**
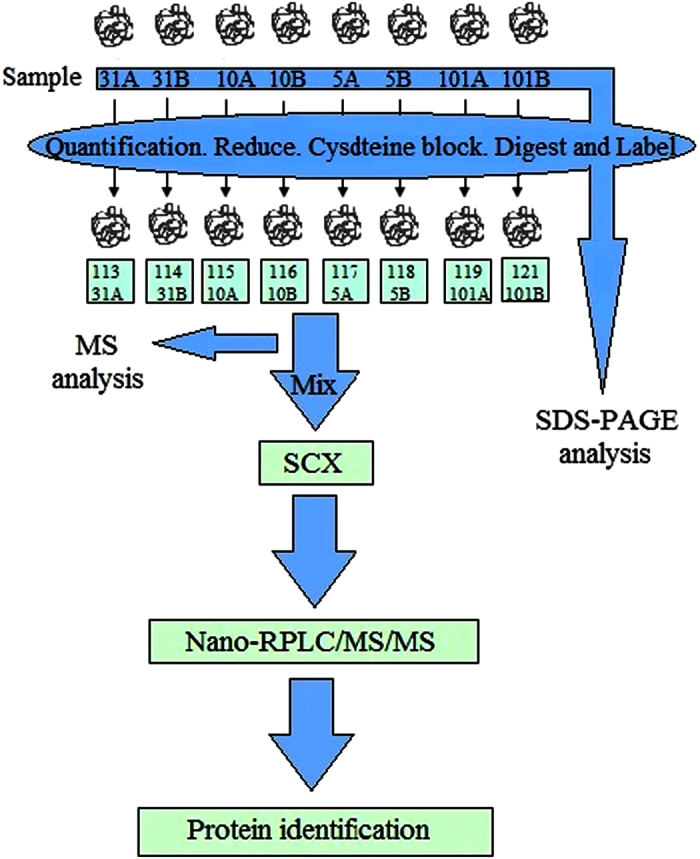
Summary of iTRAQ procedure. The extracted proteins from 4 pairs of stage III/IV gastric cancer were treated with reducing and cysteine blocking reagents. After digestion by trypsin, the peptides were labeled with iTRAQ reagents. The labeled peptides were then mixed and purified by strong cation-exchange (SCX) chromatography. The purified peptides were analyzed by LC-MS/MS for both identification and quantification. Data was analyzed by Protein Pilot Software v. 5.0 (AB SCIEX).

We next validated the expression of UQCRC1 in the above mentioned 97 GC samples. Western Blot analysis revealed that UQCRC1 expression was decreased in all stages of GC samples, compared to adjacent normal gastric tissue (Fig. 15). UQCRC1 expression was decreased in 76.47%, 74.07%, 86.05%, and 70.00% in stages I, II, III, and IV GC samples, respectively. Western Blot data were confirmed by immunofluorecence staining (Fig. 16). Our results suggest that reduced expression of UQCRC1 is associated with tumor progression. The iTRAQ procedures are summarized in Fig. 17.

The images of Western Blot and immunofluorecence staining of all 97 pairs of samples are shown in Supplementary Figures S1 And 2. The specificity of the antibodies was confirmed by blocking the antibodies with their corresponding recombinant proteins. The immunofluorecence staining for ANXA1, NNMT, FBLN5 and UQCRC1 disappeared after the antibodies have been pre-incubated with their corresponding recombinant proteins (Supplementary Fig. S3). Full length blots of Figures 4, 5, 6 and 15 are presented in Supplementary Figure S4.

## Discussion

Management of advanced gastric cancer remains a major challenge in oncology. Response to treatment is often difficult to predict due to the heterogeneity of the disease. A better understanding of tumor biology will lead to the identification of new markers for both early diagnosis and treatment. In the present study, we analyzed protein expression profile in 4 pairs of stage III/IV gastric cancer samples by iTRAQ. We found 19 proteins that are differentially expressed in all 4 pairs of samples. We then selected ANXA1, NNMT, FBLN5 and UQCRC1 for further study. These proteins were chosen because reports on their roles in gastric cancer progression remain controversial.

ANXA1 is a member of a family of calcium and membrane-binding proteins, located on the cytosolic face of plasma membrane^29^. Previous reports suggest that ANXA1 is involved in membrane aggregation, cell matrix interaction, vesicle transport, phagocytosis, proliferation, apoptosis, inflammation and cell transformation^29^. ANXA1 expression is up-regulated in breast cancer^30^, hepatocellular carcinoma^31^, melanoma^32^, and rectal cancer, but down regulated in nasopharyngeal carcinoma^33^ and cervical cancer^34^. In breast cancer, ANXA1 expression is associated with BRCA1/2 mutations^35^. Similarly, high ANXA1 expression correlated with advanced TNM stage and poor survival of human hepatocellular carcinoma (HCC) patients^31^. It has been shown that ANXA1 expressing cancer cells are resistant to chemo- and radiation therapy because these cells acquired cancer stem cell like features^36^,^37^. However, the role of ANXA1 in gastric cancer has not been established^27^,^28^. Gao *et al*. showed profound ANXA1 expression in normal gastric mucosa and glands, but reduced expression in poorly differentiated gastric tumors^27^. Another study from the same group of investigators also showed that ANXA1 expression decreased significantly as gastric cancer progressed and metastasized^38^. Results from these two studies suggest that ANXA1 is a tumor suppressor. By contrast, Sato *et al*. did not detect ANXA1 in normal gastric mucosa, but found ANXA1 expression in 56.3% cases of gastric cancer^28^. Our data demonstrated that ANXA1 is indeed over–expressed in gastric cancer, but expressed at low levels in normal gastric tissue. We further showed that ANXA1 knockdown inhibited AGS cell migration and invasion. These data suggest that ANXA1 overexpression in gastric cancer is involved in tumor progression.

NNMT catalyzes the N-methylation of nicotinamide and other pyridines to form pyridinium ions. This enzyme plays an important role in biotransformation of many drugs and xenobiotic compounds in liver. Using two dimensional gel electrophoresis techniques, it was shown that NNMT was over expressed in gastric tumor compared to normal gastric tissues^39^. Another study showed that NNMT expression is associated with poor prognosis^40^. These are the only two reports that we could find in the literature regarding the involvement of NNMT in gastric cancer. Our findings that NNMT is over expressed in GC are consistent with these previous reports. However, the mechanisms whereby NNMT promotes tumor progression remain poorly understood. Potential mechanisms include activation of Akt signaling^41^, anti-apoptosis^42^ and activation of matrix metalloproteinase-2^43^. NNMT over expression could also impair the methylation potential of cancer cells, resulting hypomethylated histones and increased expression of oncogenes^44^.

FBLN5 is a secreted extracellular matrix protein that plays a critical role in the assembly of elastic fibers. Using immunohistochemistry technique, Hwang *et al*. found high expression of FBLN5 in 48 of 84 nasopharyngeal carcinoma (NPC) samples and FBLN5 expression correlated with advanced tumor stage. They further showed that overexpxression of FBLN5 in NPC cells promote cell migration and invasion through activation of phospho-Akt^45^. There is only one report showing over expression of FBLN5 in gastric cancer^46^. In that study, FBLN5 protein expression correlated with poor differentiation and advanced TNM tumor stage. These data are consistent with our findings that FBLN5 expression is higher in stage III/IV gastric cancer samples, suggesting an important role of FBLN5 expression in cancer progression. However, the mRNA and protein expression of FBLN5 were down-regulated in ovarian carcinomas compared with control tissues^47^. Over expression of FBLN5 in ovarian cancer line SKOV3 inhibited migration and invasion in wound-healing and invasion assays. Over expression of FBLN5 in SKOV3 cells also induced G2/M arrest and increased cyclin B1, CDC2 and CDC25C^47^. Therefore, FBLN5 acts as a tumor suppressor in ovarian cancer. Similarly, FBLN5 over expression inhibited invasion and proliferation capacity of several breast cancer lines including MCF-7, T47D and MDA-MB-231^48^. Interestingly, FBLN5 also prevent these cells from forming mammospheres, a key feature of cancer stem cells^48^. Thus, depending on tumor type, FBLN5 may either promote or inhibit tumor growth through mechanisms that are not totally understood.

UQCRC1 is a component of complex III of mitochondrial respiratory chain. Liu *et al*. compared osteosarcoma cells and human primary cultured osteoblastic cells by two-dimensional gel electrophoresis and identified UQCRC1 as one of the proteins that were up regulated in osteosarcoma cells^25^. Kulawiec *et al*. analyzed the expression of UQCRC1 gene in breast and ovarian tumors and found that 74% of breast carcinomas cases were positive for UQCRC1, with positive correlation to tumor grade^49^. By contrast, only 34% ovarian adeno-carcinomas cases showed UQCRC1 expression, with a negative correlation to tumor grades^49^. Feng *et al*. investigated protein profile of human hepatocarcinoma cell line SMMC-7721 by two-dimensional electrophoresis and identified UQCRC1 as one of the novel proteins for hepatocarcinoma cell^50^. But no control cells were used, therefore, it is not possible to draw any conclusions on whether UQCRC1 was up or down regulated in hepatocarcinoma cells^50^. Using mass spectrometry, UQCRC1 was identified as one of the three up regulated proteins in the serum from patients with esophageal squamous cell carcinoma and the results suggested that UQCRC1 might be a useful serological marker for this type of cancer^51^. UQCRC1 is normally located within mitochondria; it is not known how UQCRC1 is released into circulation. Using two-dimensional gel electrophoresis technique, Cai *et al*. reported down regulation of UQCRC1 in gastric cardia cancer samples and the results were verified by RT-PCR. In our opinion, they should have performed Western Blot to confirm their findings at protein level rather than at mRNA level. Nonetheless, Cai’s findings on UQCRC1 down regulation in gastric cardia cancer samples are consistent with our results in gastric adenocarcinoma. How does UQCRC1 influence tumor progression? Previous studies have shown that deficiency in respiratory complex I activity is associated with overproduction of ROS and high metastatic potential^52^. Whether UQCRC1 deficiency could lead to ROS overproduction, which in turn promotes GC invasion, remain to be investigated.

In conclusion, our study revealed the role of ANXA1, NNMT, Fibulin-5 and UQCRC1 in gastric cancer progression. Knockdown of ANXA1 by siRNA significantly inhibited GC cell migration and invasion, whereas overexpression of ANXA1 promoted migration and invasion. We also revealed the role of decreased expression of UQCRC1, a mitochondrial enzyme involved in energy metabolism, in GC development. Future studies will clarify how these proteins participate in gastric cancer development and progression.

## Materials and Methods

### Patient sample collection

All experiments involving human subjects were performed in accordance with the Code of Ethics of the World Medical Association (Declaration of Helsinki), and the relevant guidelines and regulations of Soochow University. All experimental protocols were approved by the Research Ethics Committee of the First Affiliated Hospital of Soochow University. With informed consent from all subjects, paired specimens of gastric adenocarcinoma and adjacent normal tissues were obtained from patients who underwent surgical resection of gastric adenocarcinoma at the Department of General Surgery, First Affiliated Hospital of Soochow University. None of the patients received any anti-cancer treatment before surgery.

### iTRAQ

The detailed procedure has been described previously^53^. Briefly, the protein samples were acetone-TCA precipitated, digested by trypsin to generate proteolytic peptides which were labeled with iTRAQ reagents. The combined peptide mixtures were analyzed by LC-MS/MS for both identification and quantification (Fig. 9). Functional enrichment analysis was performed using Gene Ontology (GO) (http://www.geneontology.org/). Pathway analysis was performed by KEGG mapping (http://www.genome.jp/kegg/).

### Western Blot

Proteins extracted from patient’s samples were separated by SDS-PAGE and then transferred to PVDF membrane. After incubating with primary antibodies at 4 °C overnight, the membranes were then washed three times with Tris-buffered saline containing Tween-20 (TBST) and incubated with horseradish peroxidase-conjugated secondary antibodies (anti-rabbit or anti-mouse IgG: 1:4000, Sigma, USA) for 2 h at room temperature. The membranes were then washed again in TBST and visualized using an Enhanced ChemiLuminescence Kit (PerkinElmer). Rabbit anti-Annexin A1 (1/1000) and anti-GAPDH (1/5000) were from Cell Signaling, mouse anti-NNMT was from Santa Cruz Biotechnology (1/500), mouse anti-fibulin-5 (1/500) and rabbit anti- UQCRC1 (1/1000) were from ThermoFisher Scientific. The band density was quantified by ImageJ and normalized to GAPDH.

### Immunofluorescence staining

Frozen sections (10 μm) were incubated with Annexin A1 (Cell Signaling Technology), UQCRC1 (ThermoFisher Scientific) antibodies produced in rabbit, NNMT (Santa Cruz Biotechnology), Fibulin-5 (ThermoFisher Scientific) antibodies produced in mouse. After washing, the sections were incubated with goat anti-rabbit IgG - Alexa Fluor® 568 conjugate (Thermo Fisher Scientific), or goat anti-mouse IgG- Alexa Fluor® 488 conjugate, followed by incubation with DAPI to stain nuclei. Images were acquired using a Multiphoton Laser Scanning Microscope (FV1000, Olympus).

### Blocking experiments

The antibodies against ANXA1, NNMT, FBLN5 and UQCRC1 were pre-incubated with their corresponding recombinant proteins (antibody/recombinant protein: 1/5, by weight) diluted in blocking buffer (5% non-fat dry milk, 0.1% Tween-20 in TBS) and incubated at room temperature for 4 hours before adding to tissue sections. Recombinant human ANXA1, NNMT, and UQCRC1 proteins were from Abcam, recombinant human FBLN5 protein was from R & D Systems.

### Cell line

The human gastric carcinoma cell line AGS was purchased from Cell Bank of the Chinese Academy of Sciences (Shanghai, China), and cultured in Ham’s F-12K (Gibco) containing 10% FBS. The AGS cell line was validated by STR (short tandem repeat) analysis performed by Cell Bank of the Chinese Academy of Sciences (Shanghai, China) and the cells was resuscitated immediately after purchase and was used within 6 passages.

### siRNA transfection

For knockdown of Annexin A1, AGS cells were transfected with small interfering RNA (siRNA) targeting Annexin A1. siRNA and negative control (NC) were purchased from GenePharma (Shanghai, China). NC: 5′-UUCUCCGAACGUGUCACGUTT-3′, 5′-ACGUGACACGUUCGGAGAATT-3′AnnexinA1–452: 5′-GCAGCAUAUCUCCAGGAAATT-3′, 5′-UUUCCUGGAGAUAUGCUGCTT-3′, AnnexinA1-743: 5′-GCUUUGCUUUCUCUUGCUATT-3′, 5′-UAGCAAGAGAAAGCAAAGCTT-3′AnnexinA1-1061: 5′-GCCAUGAAAGGUGUUGGAATT-′, 5′-UUCCAACACCUUUCAUGGCTT-3′.

The cells were transfected according to the manufacturer’s instructions. AGS cells were seeded to 6-well plates (1 × 10^6^ cells/well) the night before transfection. The transfection was designed for one RNA amount (A) combined with one amount of Lipofectamine RNAiMAX (B). A. 1.5 μl siRNAs (30 pmol) was added to 150 μl Opti-MEM; B. Lipofectamine RNAiMAX (9 μl, Invitrogen), was added to 150 μl Opti-MEM. A and B was combined and incubated at room temperature for 20 mins. Finally, 250 μl of the mixture was added to each well of the 6-well plates.

### Annexin A1 overexpression

Expression plasmids carrying Annexin A1 was purchased from Sino biological Inc (Beijing, China). The plasmid (3 μg) was diluted in 150 μl Opti-MEM which were then mixed with equal volumes of Opti-MEM containing 6 μl Lipofectamine 2000. The mixture was incubated for 20 minutes at room temperature before adding to cells.

### Wound-healing assay

Cell migration was evaluated by wound healing assay. Briefly, 1 × 10^6^ AGS cells/well were plated in a 6-well plate and cultured overnight to yield a sub-confluent monolayer. The cells were transfected with Annexin A1 siRNA or plasmid overexpressing Annexin A1, then wounded with a 200 μl pipette tip. The remaining cells were washed with PBS. Photographs were taken at 0 and 24 hours. The distance of the remaining open wound area was calculated as percentage of the area at time 0.

### Migration and Invasion assays

Cell invasion was determined using a transwell matrigel invasion assay in 24-well Transwell units (Costar). Matrigel diluted with the precooled serum-free Ham’s F-12K (50 μl) was added to the upper chamber of the Transwell and incubated at 37 °C for 30 minutes. The siRNAs or NC was prepared as described above. Following incubation for 20 minutes, 100 μl of the siRNA mixture was mixed to 100 μl serum-free Ham’s F-12K containing 1 × 10^5^ cells and then transferred to Matrigel coated top chambers. The lower chambers were filled with 500 μl Ham’s F-12K supplemented with 10% FBS. After incubation at 37 °C for 24 hours, the non-invading cells were removed with a cotton swab. The inserts were removed from the top chambers, washed with PBS, fixed and stained with Giemsa. The invaded cells were counted in five random fields under a light microscope. The procedure for cell migration is similar to that of invasion, except that matrigel was not added.

### Real-time PCR

RNA was extracted using the Trizol reagent (Ambion), and cDNA was synthesized using the RevertAid First Strand cDNA Synthesis Kit (ThermoFisher Scientific). Real-time PCR was performed using the ABI 7500 Real Time PCR System (ABI) with the following primers: AnnexinA1-F:AAGCAGGAGAAAGGAGAAAGG AnnexinA1-R:AACTCCAGGTCCAGAACTTTG, GAPDH-F:ACCCAGAAGACTGTGGATGG GAPDH-R:CAGTGAGCTTCCCGTTCAG. Relative expression was calculated from cycle threshold (Ct; relative expression = 2^−(SΔCt−CΔCt)^) values using GAPDH as internal control for each samples.

### Statistical analysis

Data were presented as means ± SD. The t*-*test was used to determine the significance of the differences between two groups for the invasion assay. Mann-whitney test was used to determine the significance for Western Blot experiments for validation of iTRAQ results in 97 stage I-IV gastric cancer samples. *P* < 0.05 was considered statistically significant.

## Additional Information

**How to cite this article**: Wang, X. *et al*. Identification of specific biomarkers for gastric adenocarcinoma by ITRAQ proteomic approach. *Sci. Rep.* **6**, 38871; doi: 10.1038/srep38871 (2016).

**Publisher's note:** Springer Nature remains neutral with regard to jurisdictional claims in published maps and institutional affiliations.

## Supplementary Material

Supplementary Figure

## Figures and Tables

**Table 1:** Demographic characteristics of GC patients for iTRAQ analysis.

Case Number	Age	Gender	Histological grade	Stage	TNM	HER-2
5	60	Female	adenocarcinoma, low differentiation	IIIB	T3N2M0	Negative
10	69	Female	adenocarcinoma, low differentiation	IIIB	T3N2M0	Negative
31	58	Female	adenocarcinoma, low differentiation	IIIB	T3N2M0	Negative
101	58	Male	adenocarcinoma, low differentiation	IV	T4N1M1	Negative

**Table 2:** Proteins that are upregulated in gastric cancer by iTRAQ analysis.

N	Accession	Gene symbol	Name	31A:31B	10A:10B	5A:5B	101A:101B
1	sp|P35579|MYH9_HUMAN	MYH9	Myosin-9	2.729	3.0761	10.8643	6.368
2	sp|Q15063|POSTN_HUMAN	POSTN	Periostin	8.3946	1.7219	17.5388	19.5884
3	sp|Q07954|LRP1_HUMAN	LRP1	Low-density lipoprotein receptor-related protein 1	1.803	4.0551	4.6132	1.4454
4	sp|P04083|ANXA1_HUMAN	ANXA1	Annexin A1	3.0479	1.9953	4.4055	3.1046
5	sp|P37802|TAGL2_HUMAN	TAGLN2	Transgelin-2	6.368	1.9409	8.9536	6.9823
6	sp|Q12805|FBLN3_HUMAN	EFEMP1	EGF-containing fibulin-like extracellular matrix protein 1	1.3062	18.7068	26.3027	7.7983
7	sp|P13797|PLST_HUMAN	PLS3	Plastin-3	2.4889	2.5351	13.0617	3.4356
8	sp|P35443|TSP4_HUMAN	THBS4	Thrombospondin-4	1.7701	12.942	14.3219	2.884
9	sp|Q9UBX5|FBLN5_HUMAN	FBLN5	Fibulin-5	2.6546	17.2187	40.5509	8.4723
10	sp|P40261|NNMT_HUMAN	NNMT	Nicotinamide N-methyltransferase	3.6308	4.8753	31.6228	15.8489

A: tumor, B: adjacent normal gastric tissue.

**Table 3:** Proteins that are downregulated in gastric cancer by iTRAQ analysis.

N	Accession	Gene symbol	Name	31A:31B	10A:10B	5A:5B	101A:101B
1	sp|Q99798|ACON_HUMAN	ACO2	Aconitate hydratase, mitochondrial	0.4571	0.2754	0.4055	0.1556
2	sp|P28331|NDUS1_HUMAN	NDUFS1	NADH-ubiquinone oxidoreductase 75 kDa subunit, mitochondrial	0.5861	0.2421	0.5105	0.3221
3	sp|Q13423|NNTM_HUMAN	NNT	NAD(P) transhydrogenase, mitochondrial	0.3467	0.2535	0.6607	0.1837
4	sp|P48735|IDHP_HUMAN	IDH2	Isocitrate dehydrogenase [NADP], mitochondrial	0.2355	0.0673	0.4487	0.3221
5	sp|P68871|HBB_HUMAN	HBB	Hemoglobin subunit beta	0.3802	0.2188	0.1009	0.1459
6	sp|Q13228|SBP1_HUMAN	SELENBP1	Selenium-binding protein 1	0.631	0.3981	0.6252	0.0766
7	sp|P24752|THIL_HUMAN	ACAT1	Acetyl-CoA acetyltransferase, mitochondrial	0.3532	0.2754	0.2831	0.1067
8	sp|P31930|QCR1_HUMAN	UQCRC1	Cytochrome b-c1 complex subunit 1, mitochondrial	0.5754	0.3133	0.5495	0.4699
9	sp|Q16836|HCDH_HUMAN	HADH	Hydroxyacyl-coenzyme A dehydrogenase, mitochondrial	0.5649	0.4365	0.4656	0.2938

**Table 4:** Demographic characteristics of GC patients for Westernblot validation.

	Age		Gender		Histological grade		Group stage				Lymph node metastasis
	>64	≤64	Male	Female	Moderately differentiation	Poorly differentiation	I	II	III	IV	59
N(%)	54 (55.7)	43 (44.3)	70 (72.2)	27 (27.8)	41 (48.5)	56 (57.7)	17 (17.5)	27 (27.8)	43 (44.3)	10 (10.3)	
Range	65–88	35–64									
