# Supplementary material for: Identification of specific biomarkers for gastric adenocarcinoma by ITRAQ proteomic approach
Source: Sci Rep. 2016 Dec 12;6:38871. doi: 10.1038/srep38871 (PMC5150883; doi:10.1038/srep38871)

Identification of specific biomarkers for gastric adenocarcinoma by ITRAQ proteomic approach.

Xiaoxiao Wang, Qiaoming Zhi, Songbai Liu, Sheng-Li Xue, Congcong Shen,Yangxin Li, Chaofan Wu, Zaixiang Tang, Weichang Chen, Jenny Lee Song, Meiyu Bao, Yao-Hua Song，Jin Zhou.

Supplementary Figures.

Supplementary Figure S1.

Supplementary Figure S2.

Supplementary Figure S3.

Supplementary Figure S4.

Supplementary Figure S1. Validation of iTRAQ results by Western Blot.

ANXA1, NNMT, FBLN5 and UQCRC1 expression were analyzed in 97 pairs of GC samples and matched adjacent normal gastric tissues by Western Blot. (A or WT: cancer, B or WN: adjacent normal gastric tissue).

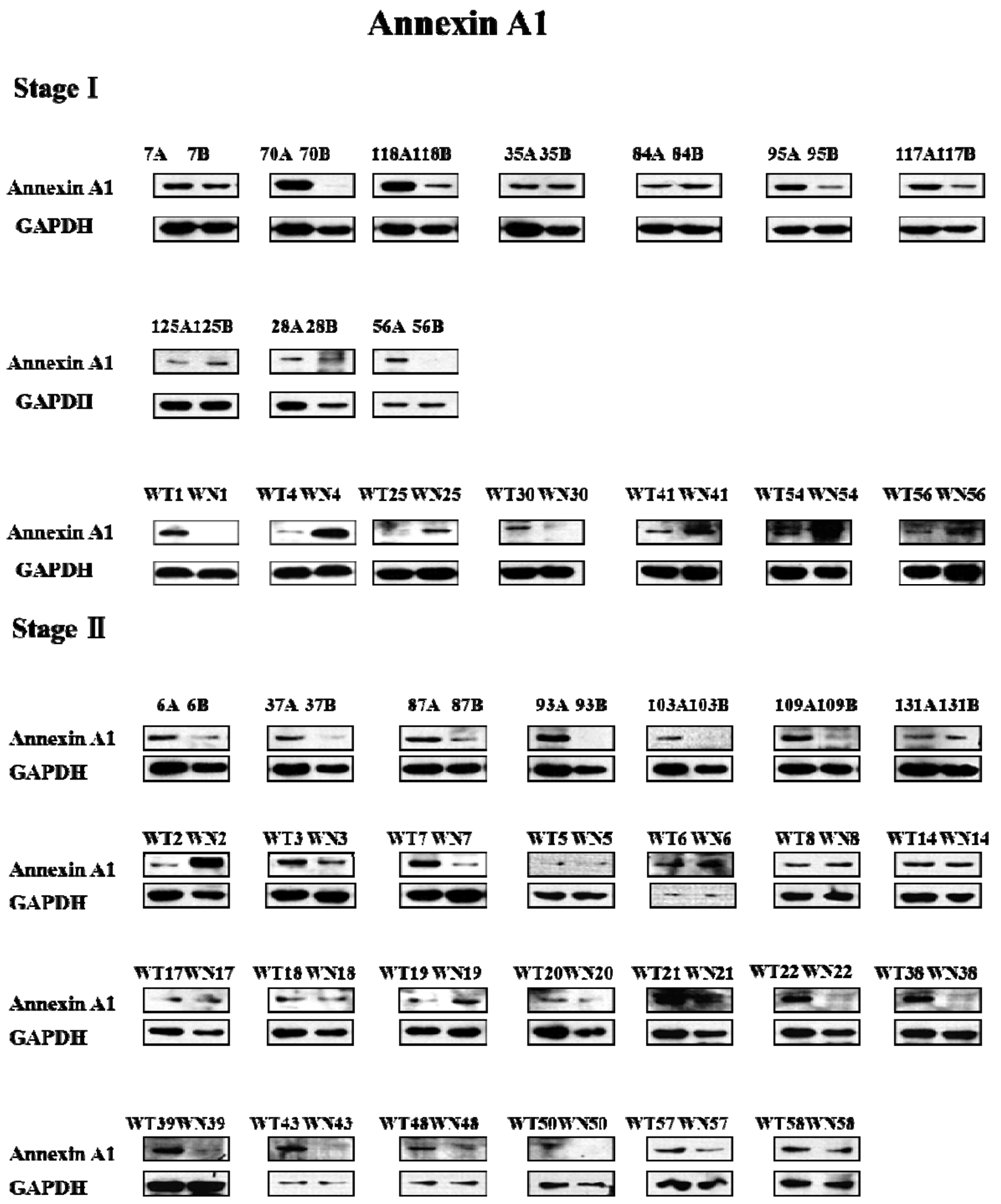

Stage III

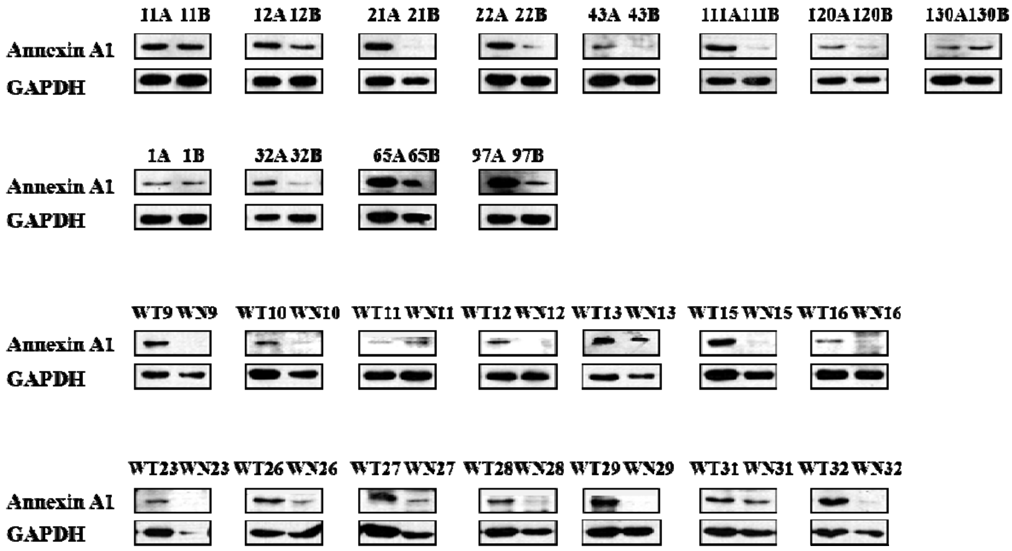

Stage III

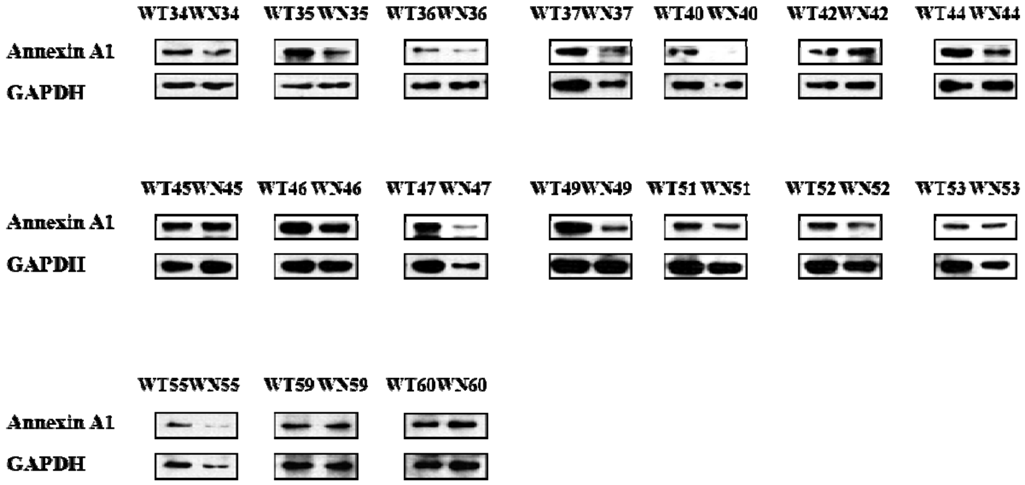

Stage IV

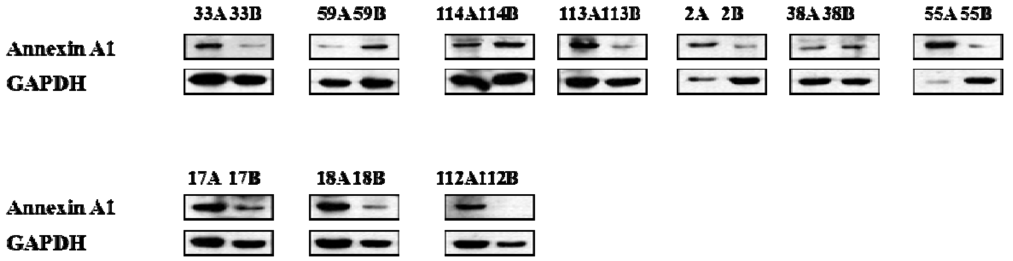

NNMT

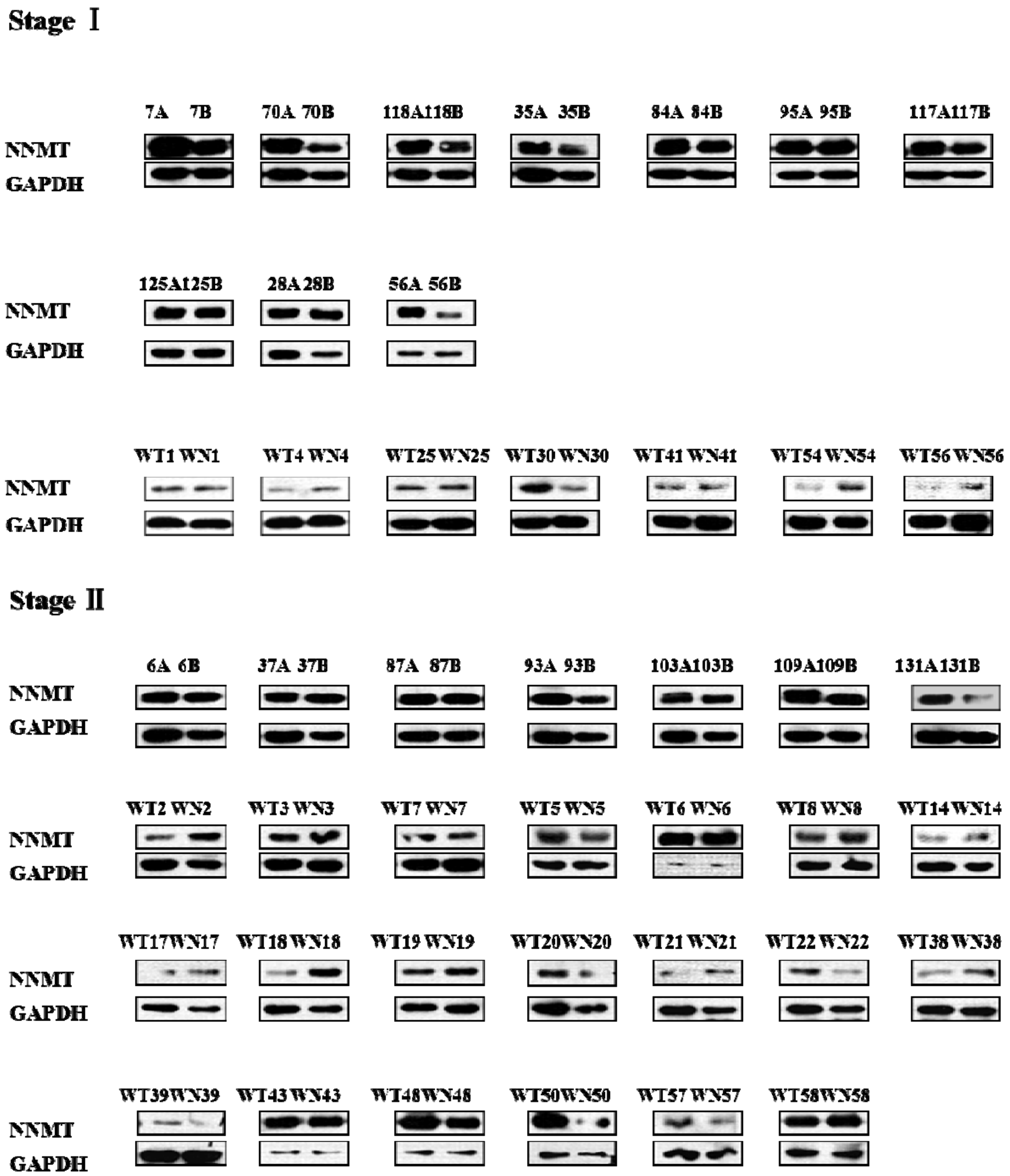
Stage Ⅰ
7A 7B
70A 70B
118A118B
35A 35B
84A 84B
95A 95B
117A117B
NNMT
GAPDH
125A125B
28A 28B
56A 56B
NNMT
GAPDH
WT1 WN1
WT4 WN4
WT25 WN25
WT30 WN30
WT41 WN41
WT54 WN54
WT56 WN56
NNMT
GAPDH
Stage Ⅱ
6A 6B
37A 37B
87A 87B
93A 93B
103A103B
109A109B
131A131B
NNMT
GAPDH
WT2 WN2
WT3 WN3
WT7 WN7
WT5 WN5
WT6 WN6
WT8 WN8
WT14 WN14
NNMT
GAPDH
WT17WN17
WT18 WN18
WT19 WN19
WT20WN20
WT21 WN21
WT22 WN22
WT38 WN38
NNMT
GAPDH
WT39WN39
WT43 WN43
WT48WN48
WT50WN50
WT57 WN57
WT58WN58
NNMT
GAPDH

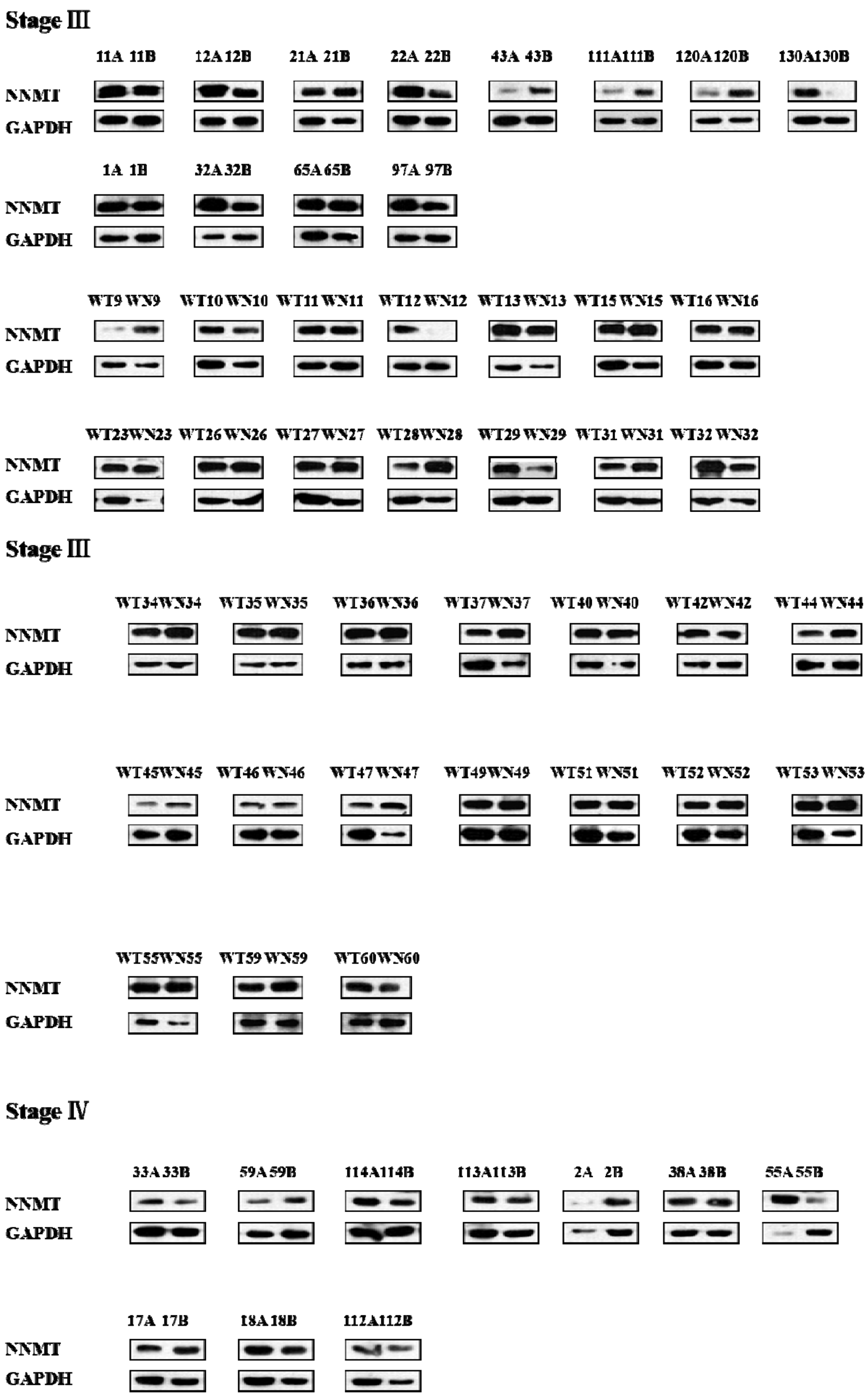
Stage III
11A 11B 12A 12B 21A 21B 22A 22B 43A 43B 111A111B 120A120B 130A130B
NNMT
GAPDH
1A 1B 32A 32B 65A 65B 97A 97B
NNMT
GAPDH
WT9 WN9 WT10 WN10 WT11 WN11 WT12 WN12 WT13 WN13 WT15 WN15 WT16 WN16
NNMT
GAPDH
WT23WN23 WT26 WN26 WT27 WN27 WT28WN28 WT29 WN29 WT31 WN31 WT32 WN32
NNMT
GAPDH
Stage III
WT34WN34 WT35 WN35 WT36WN36 WT37WN37 WT40 WN40 WT42WN42 WT44 WN44
NNMT
GAPDH
WT45WN45 WT46 WN46 WT47 WN47 WT49WN49 WT51 WN51 WT52 WN52 WT53 WN53
NNMT
GAPDH
WT55WN55 WT59 WN59 WT60WN60
NNMT
GAPDH
Stage IV
33A 33B 59A 59B 114A114B 113A113B 2A 2B 38A 38B 55A 55B
NNMT
GAPDH
17A 17B 18A 18B 112A112B
NNMT
GAPDH

Fibulin 5

Stage Ⅰ

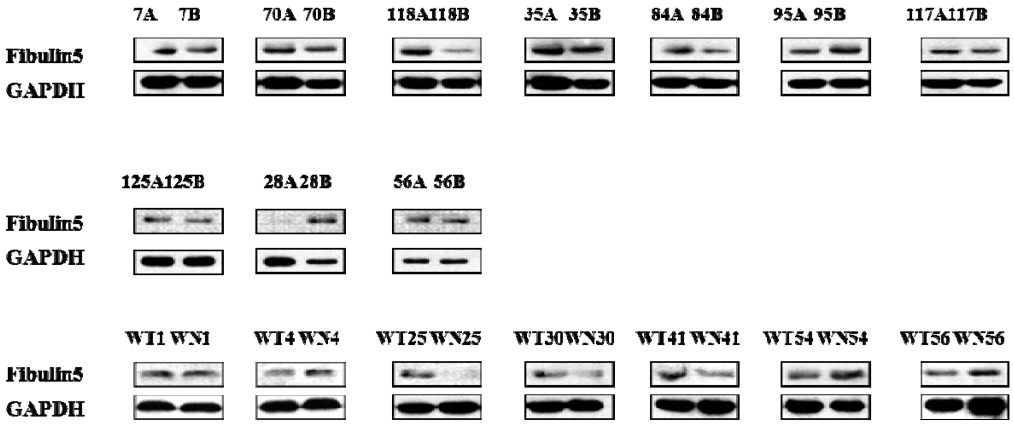

Stage Ⅱ

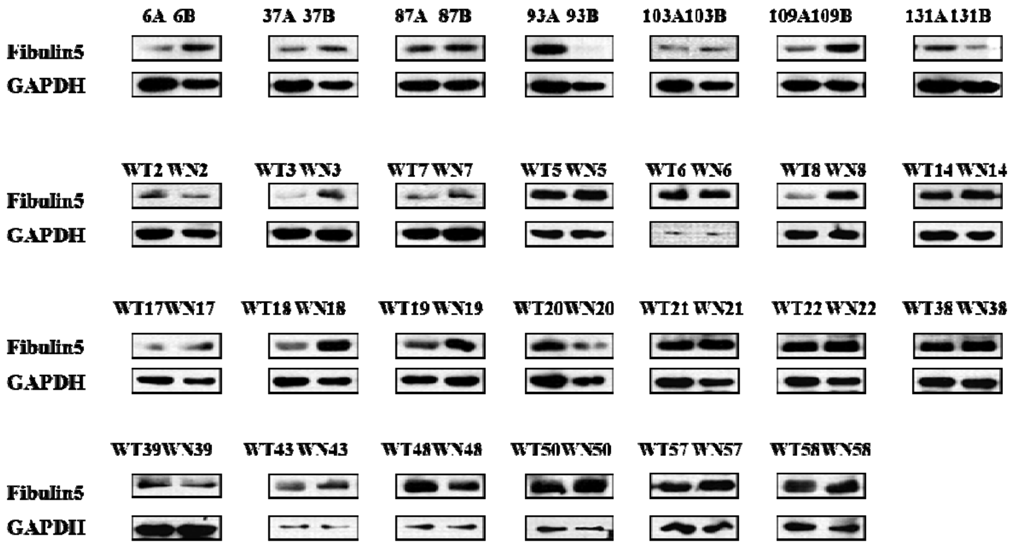

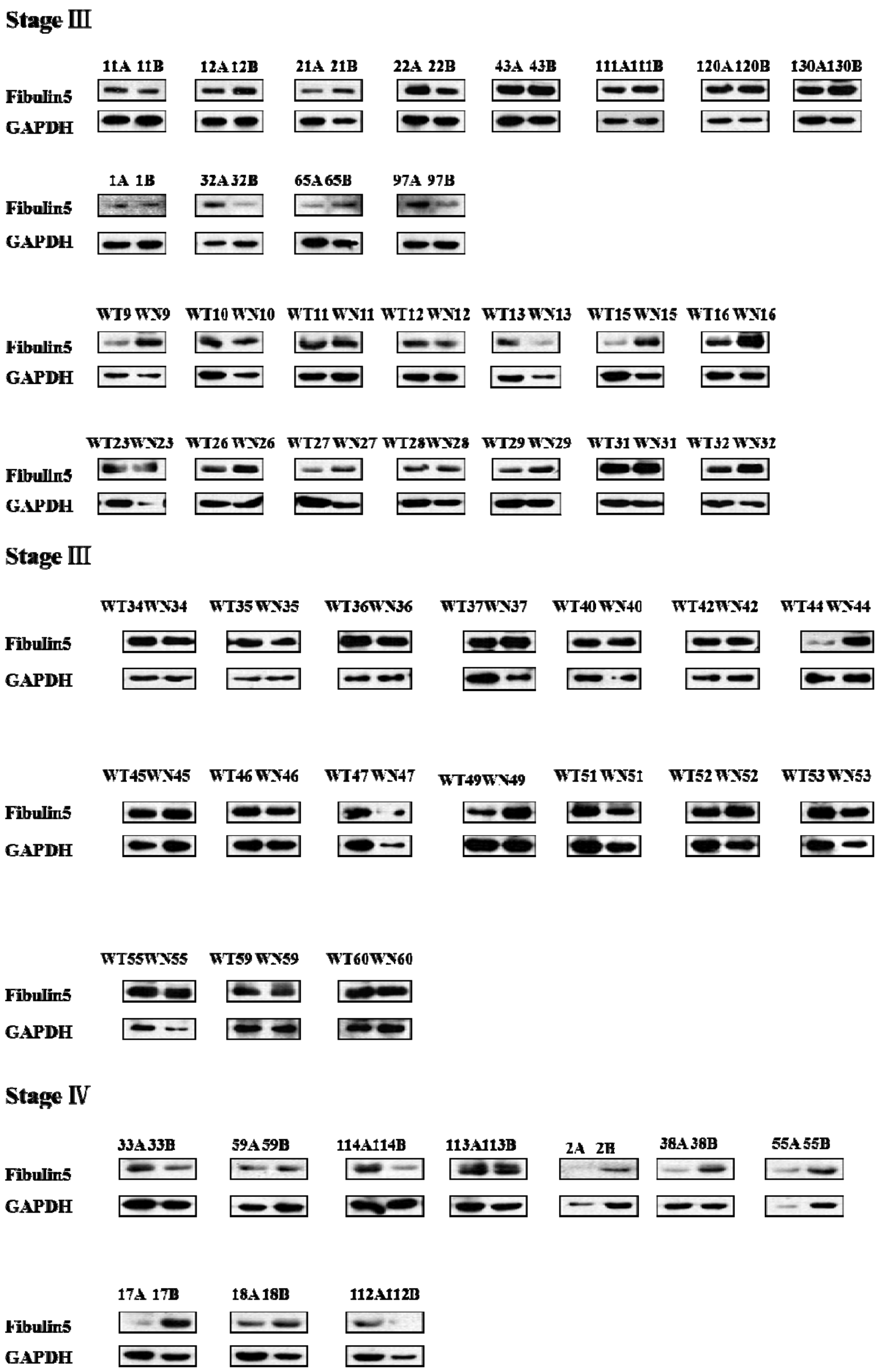
Stage III
11A 11B
12A 12B
21A 21B
22A 22B
43A 43B
111A 111B
120A 120B
130A 130B
Fibulin5
GAPDH
1A 1B
32A 32B
65A 65B
97A 97B
Fibulin5
GAPDH
WT9 WN9
WT10 WN10
WT11 WN11
WT12 WN12
WT13 WN13
WT15 WN15
WT16 WN16
Fibulin5
GAPDH
WT23 WN23
WT26 WN26
WT27 WN27
WT28 WN28
WT29 WN29
WT31 WN31
WT32 WN32
Fibulin5
GAPDH
Stage III
WT34 WN34
WT35 WN35
WT36 WN36
WT37 WN37
WT40 WN40
WT42 WN42
WT44 WN44
Fibulin5
GAPDH
WT45 WN45
WT46 WN46
WT47 WN47
WT49 WN49
WT51 WN51
WT52 WN52
WT53 WN53
Fibulin5
GAPDH
WT55 WN55
WT59 WN59
WT60 WN60
Fibulin5
GAPDH
Stage IV
33A 33B
59A 59B
114A 114B
113A 113B
2A 2B
38A 38B
55A 55B
Fibulin5
GAPDH
17A 17B
18A 18B
112A 112B
Fibulin5
GAPDH

UQCRC1

Stage Ⅰ

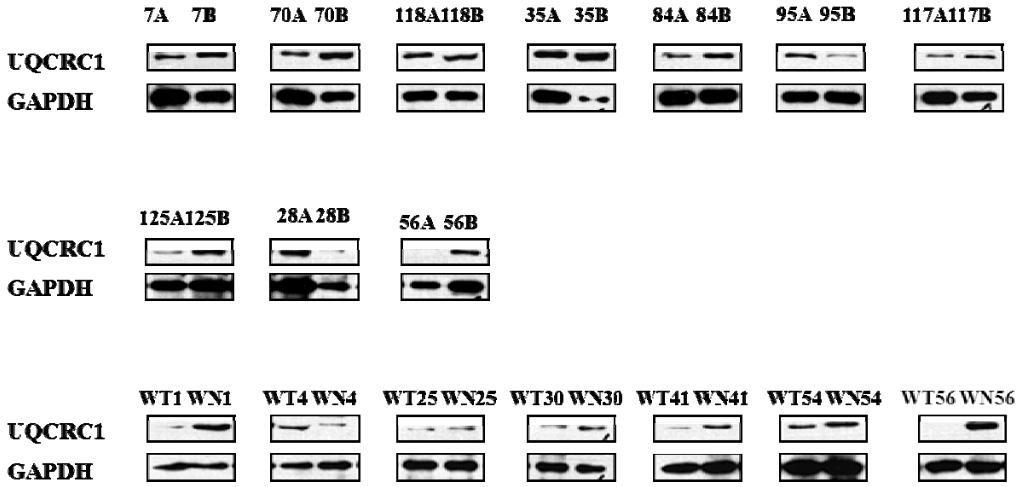

Stage Ⅱ

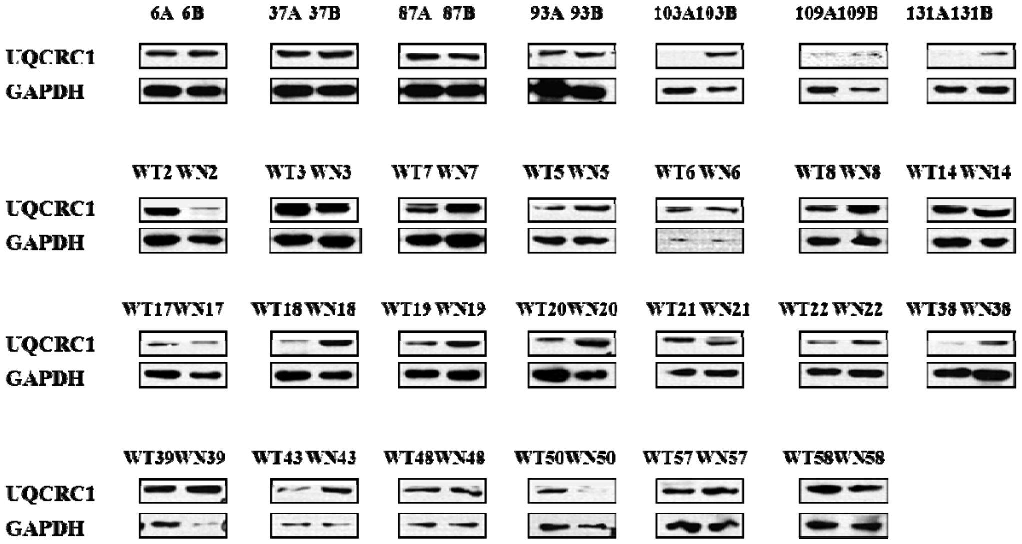

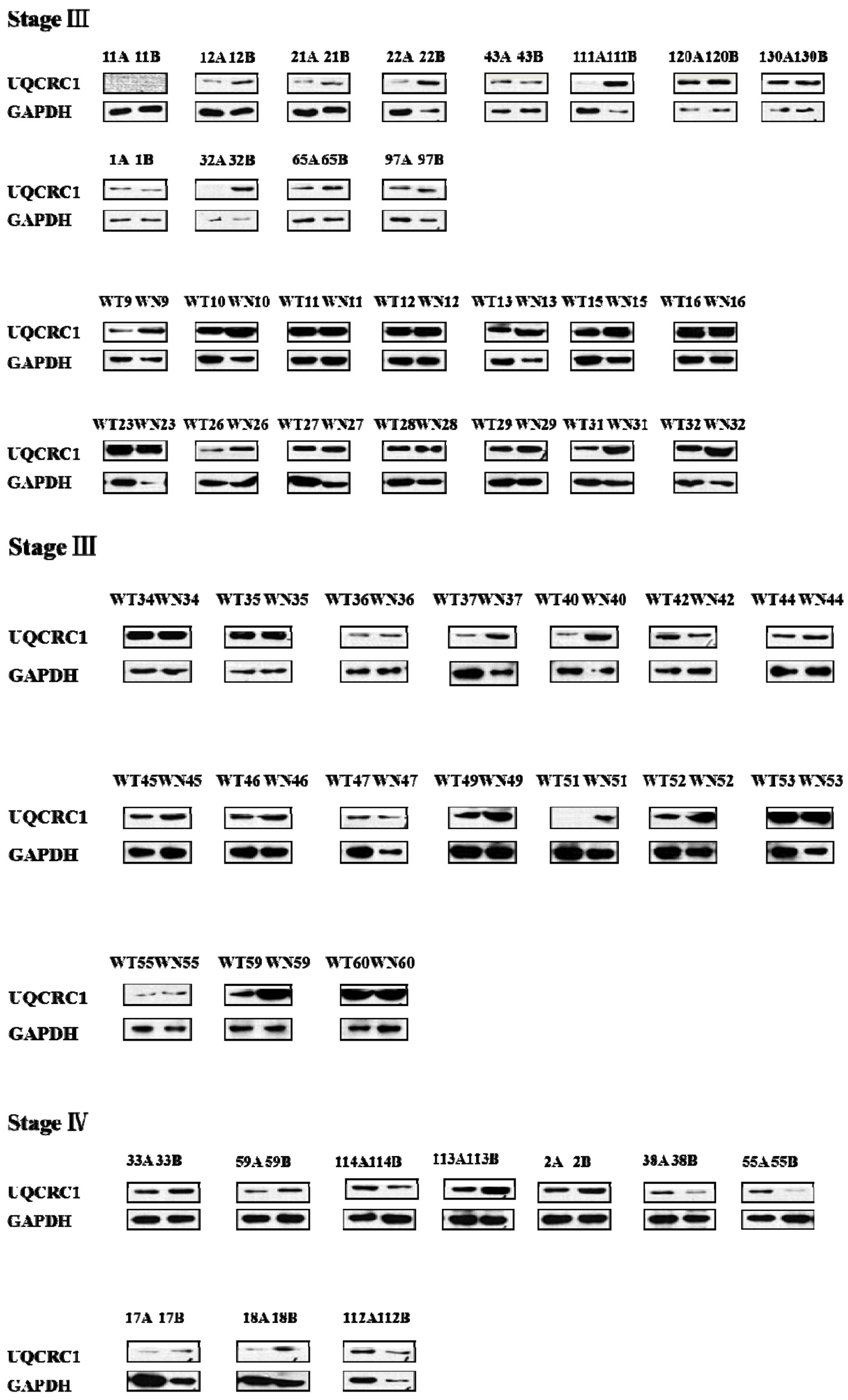
Stage III
11A 11B
12A 12B
21A 21B
22A 22B
43A 43B
111A111B
120A120B
130A130B
UQCRC1
GAPDH
1A 1B
32A 32B
65A 65B
97A 97B
UQCRC1
GAPDH
WT9 WN9
WT10 WN10
WT11 WN11
WT12 WN12
WT13 WN13
WT15 WN15
WT16 WN16
UQCRC1
GAPDH
WT23WN23
WT26 WN26
WT27 WN27
WT28WN28
WT29 WN29
WT31 WN31
WT32 WN32
UQCRC1
GAPDH
Stage III
WT34WN34
WT35 WN35
WT36WN36
WT37WN37
WT40 WN40
WT42WN42
WT44 WN44
UQCRC1
GAPDH
WT45WN45
WT46 WN46
WT47 WN47
WT49WN49
WT51 WN51
WT52 WN52
WT53 WN53
UQCRC1
GAPDH
WT55WN55
WT59 WN59
WT60WN60
UQCRC1
GAPDH
Stage IV
33A 33B
59A 59B
114A114B
113A113B
2A 2B
38A 38B
55A 55B
UQCRC1
GAPDH
17A 17B
18A 18B
112A112B
UQCRC1
GAPDH

Supplementary Figure 2. Validation of iTRAQ results by immunofluorescence staining. Frozen sections from 97 pairs of GC and their adjacent normal gastric tissues were incubated with antibody against human ANXA1, NNMT, FBLN5 and UQCRC1, followed by goat anti-rabbit IgG - Alexa Fluor® 568 conjugate, or goat anti-mouse IgG- Alexa Fluor® 488 conjugate. Nuclei are stained with DAPI (blue). (A or WT: cancer, B or WN: adjacent normal gastric tissue).

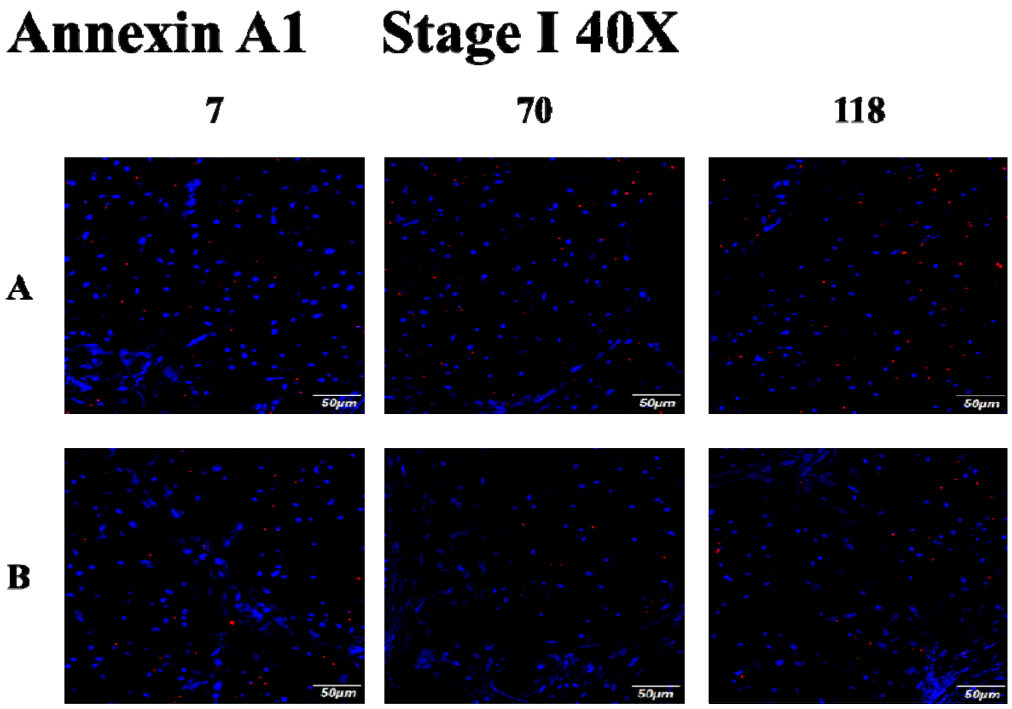

Annexin A1 Stage I 40X

35 84 95

A

B

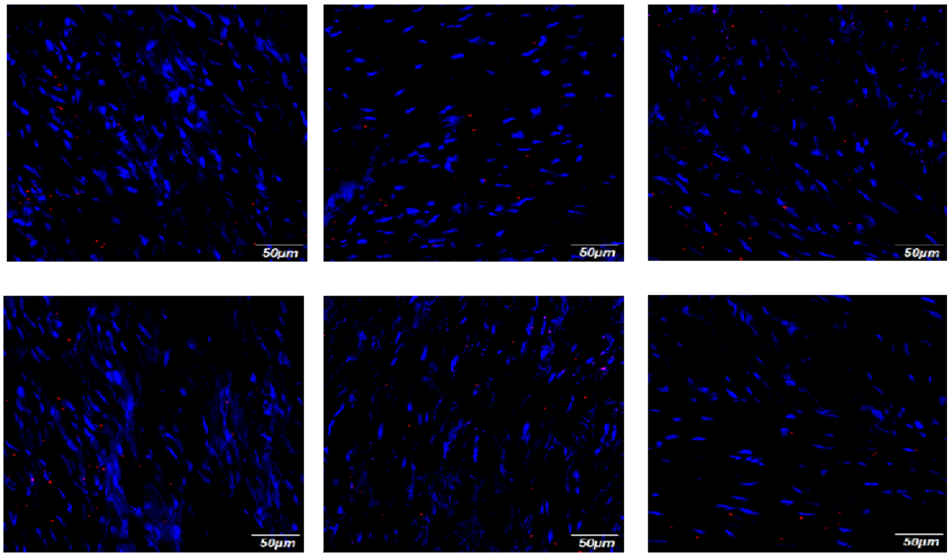

Annexin A1 Stage I 40X

117 125 28

A

B

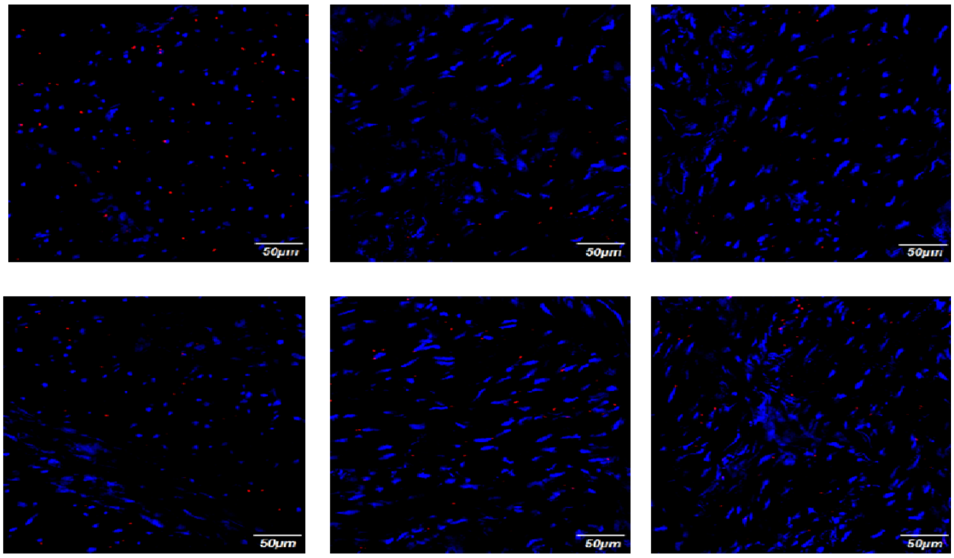

Annexin A1 Stage I 40X

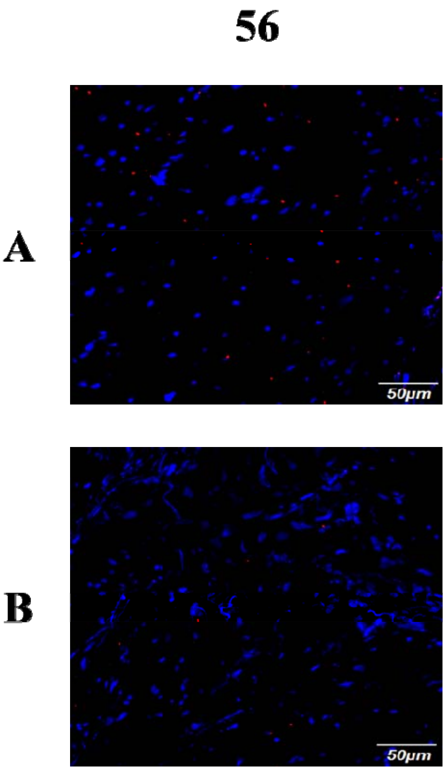

Annexin A1 Stage I 40X

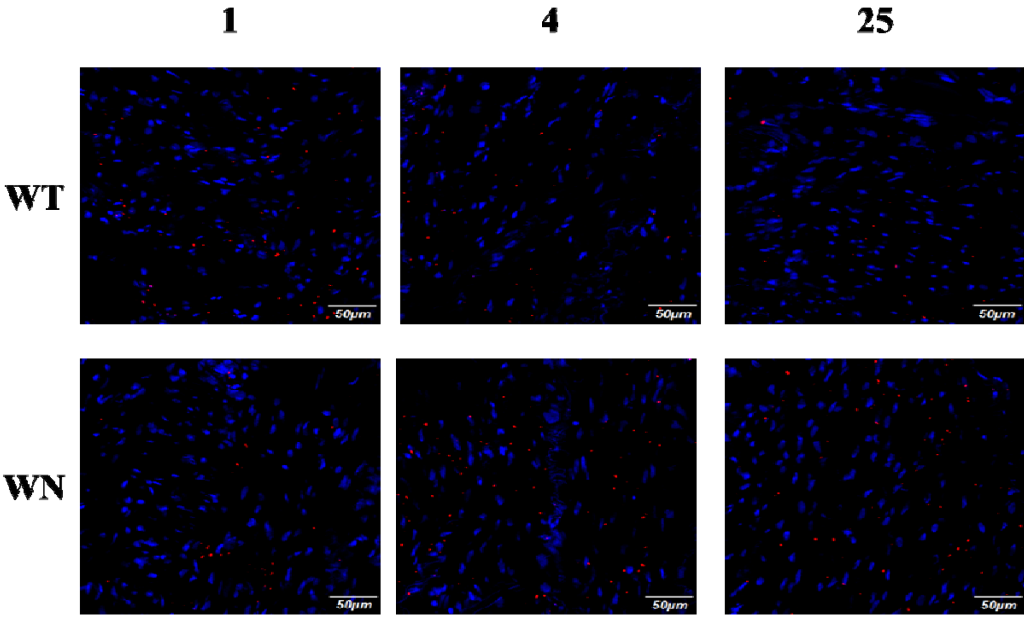

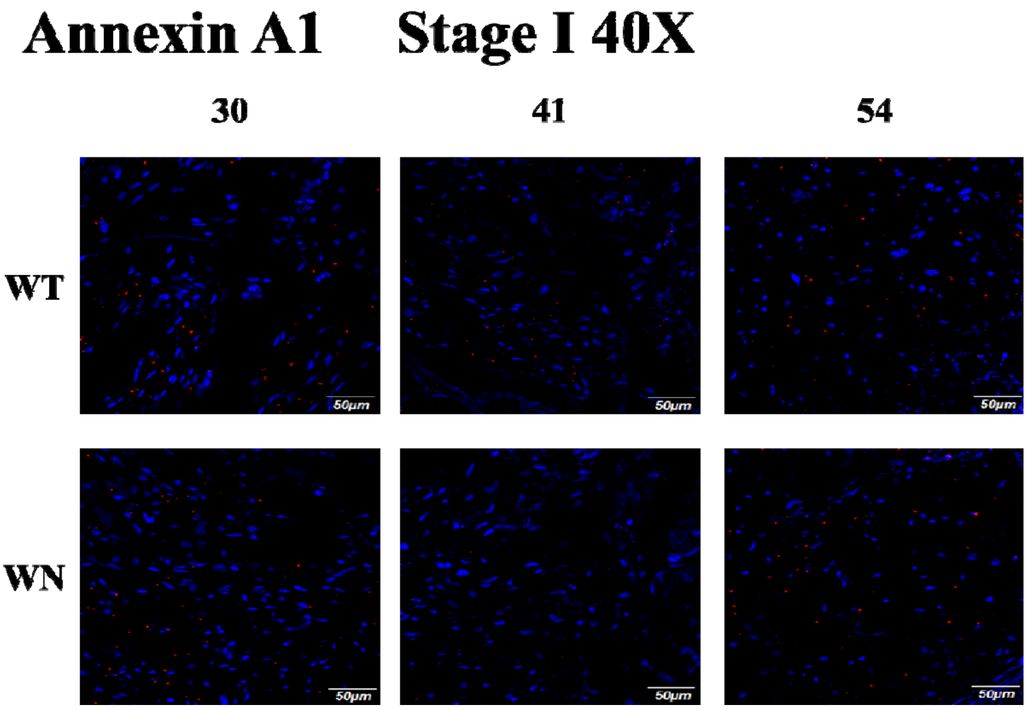
Annexin A1 Stage I 40X
30
41
54
WT
WN
50μm

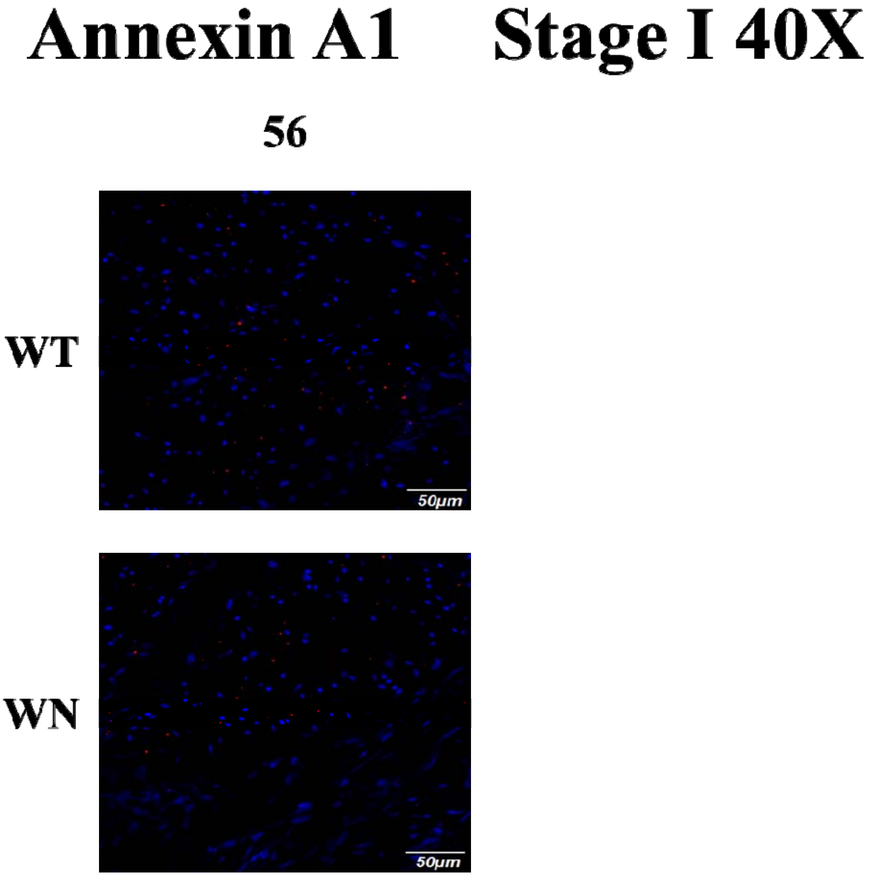
Annexin A1 Stage I 40X
56
WT
WN
50μm

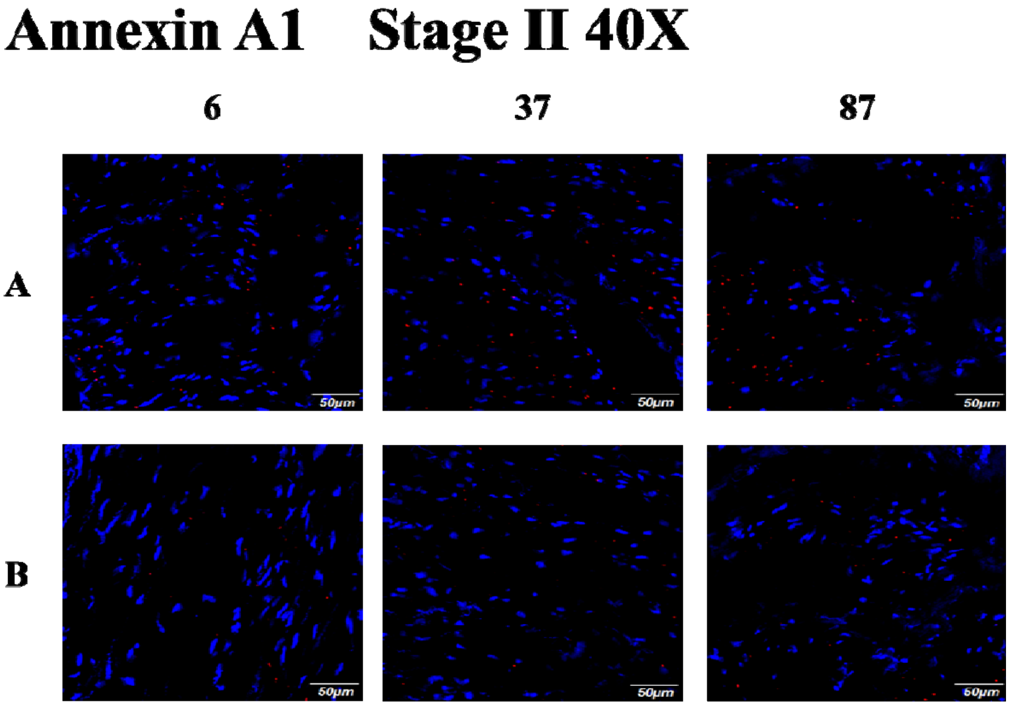
Annexin A1 Stage II 40X
6
37
87
A
B
50μm

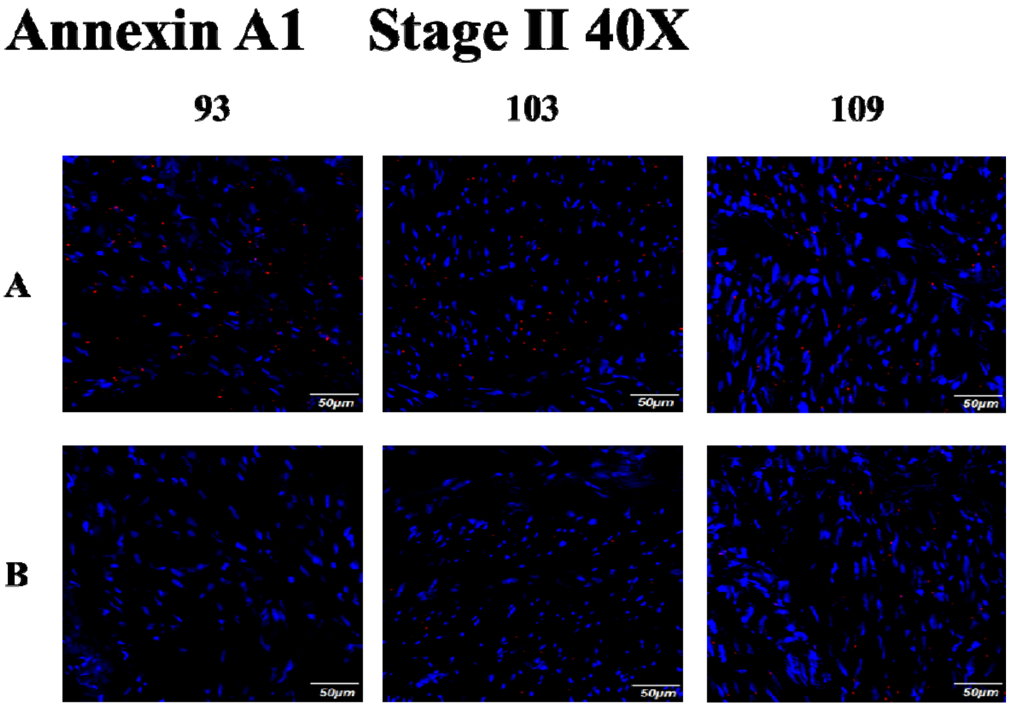
Annexin A1 Stage II 40X
93
103
109
A
B
50μm

Annexin A1 Stage II 40X

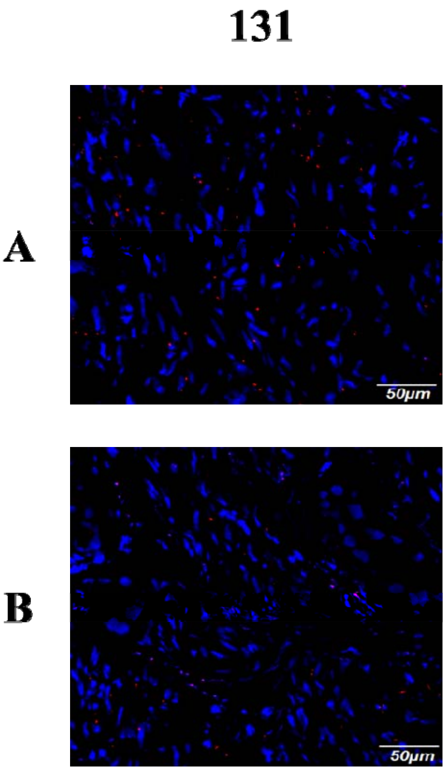

Annexin A1 Stage II 40X

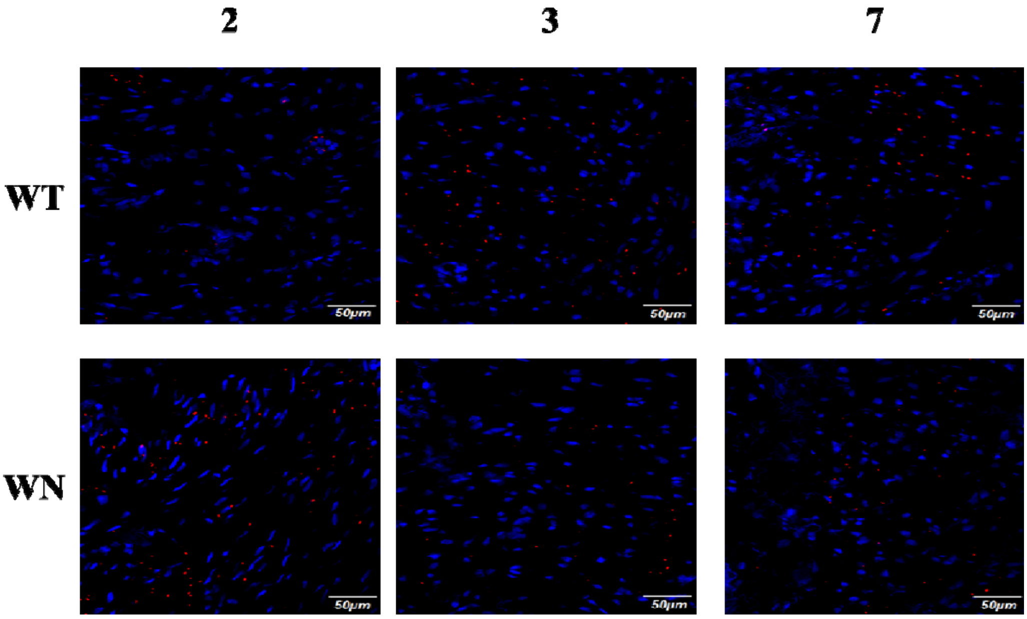

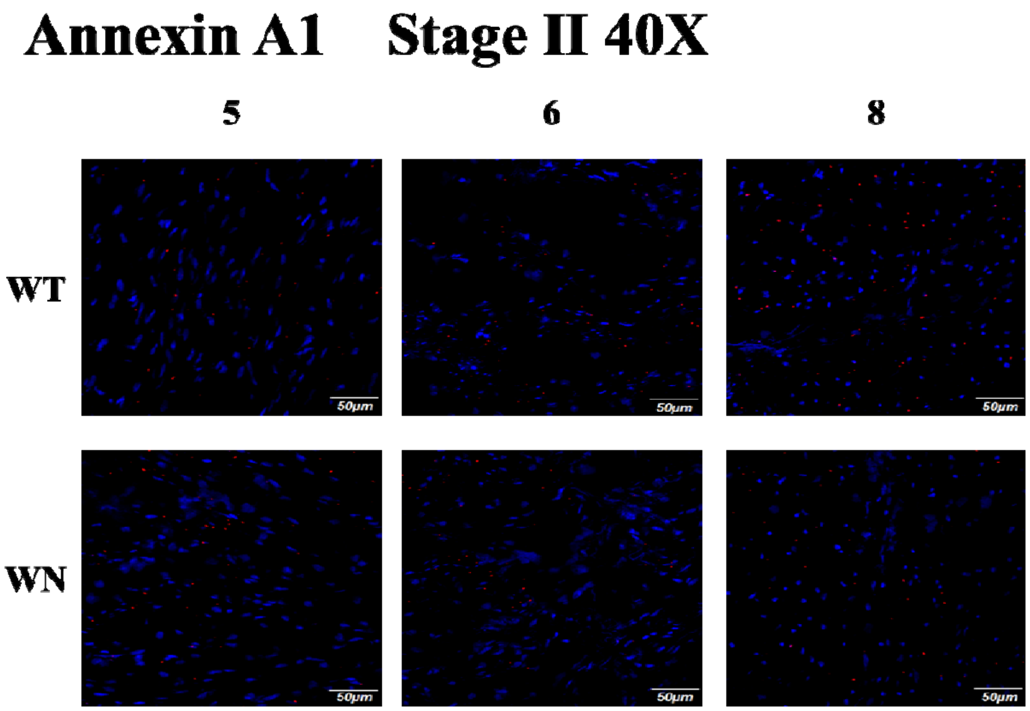
Annexin A1 Stage II 40X
5
6
8
WT
WN
50μm

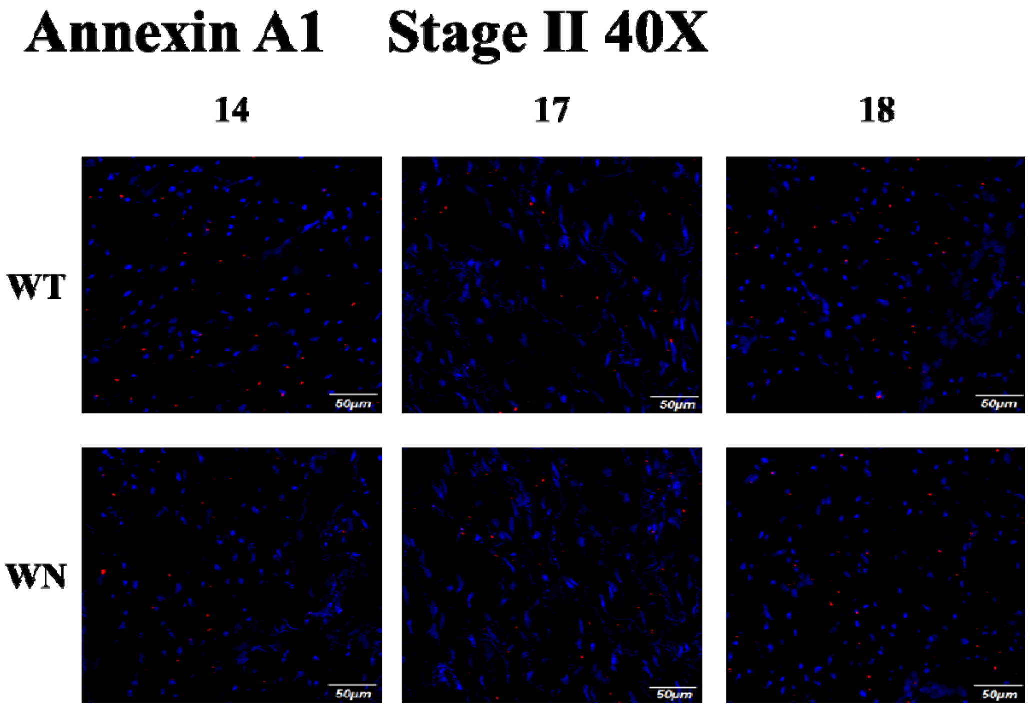
Annexin A1 Stage II 40X
14
17
18
WT
WN
50μm

Annexin A1 Stage II 40X

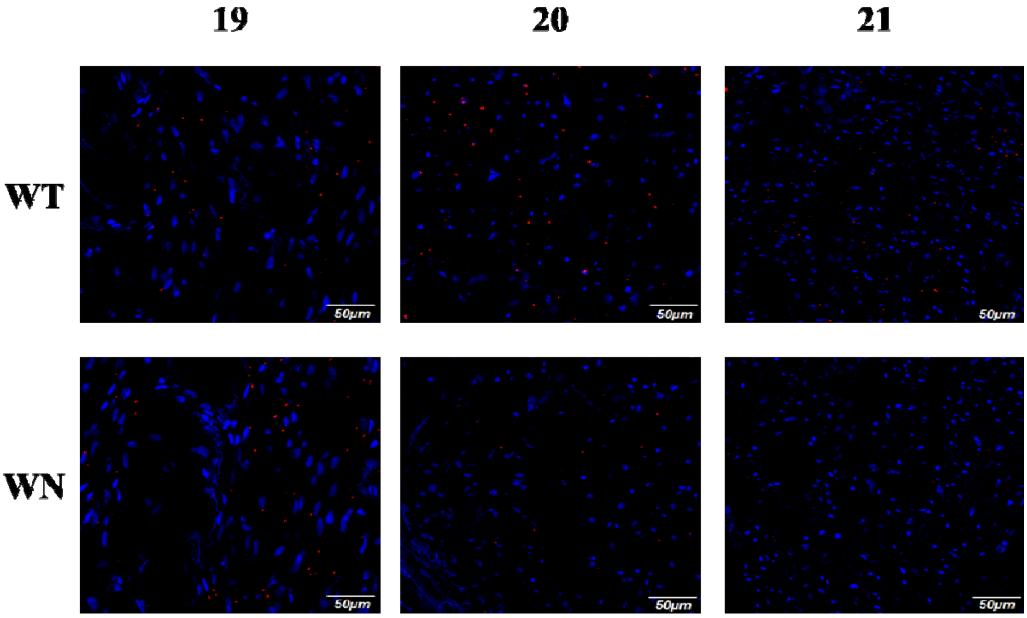

Annexin A1 Stage II 40X

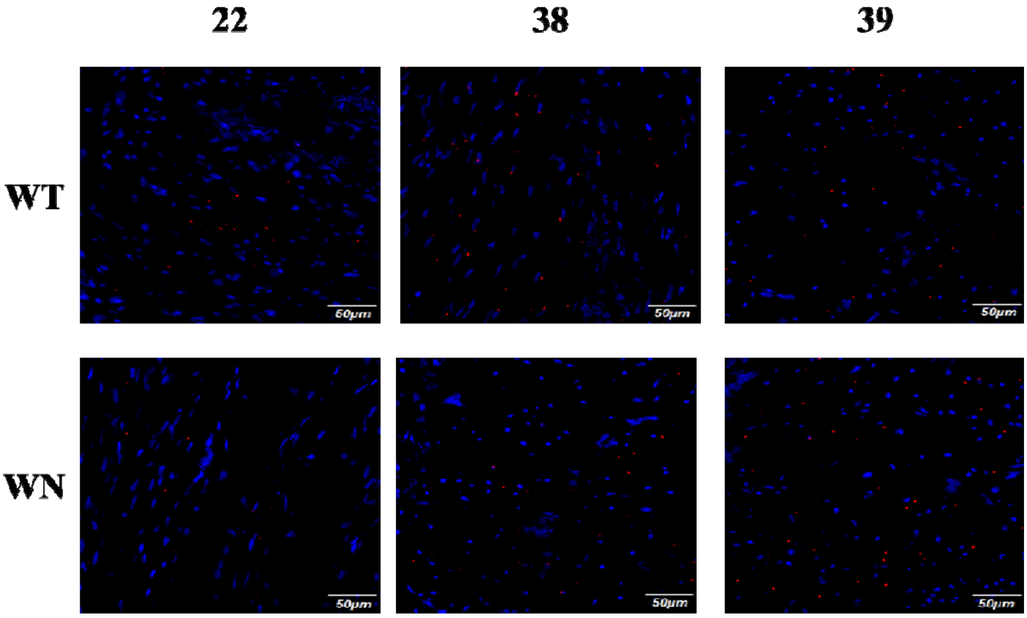

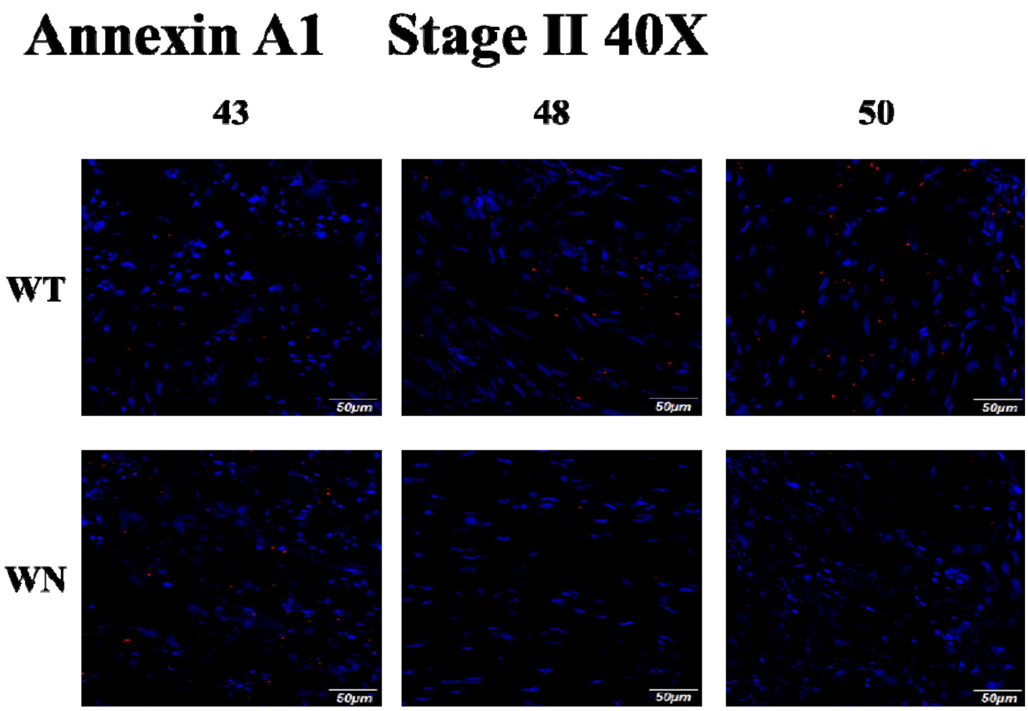
Annexin A1 Stage II 40X
43
48
50
WT
WN
50μm

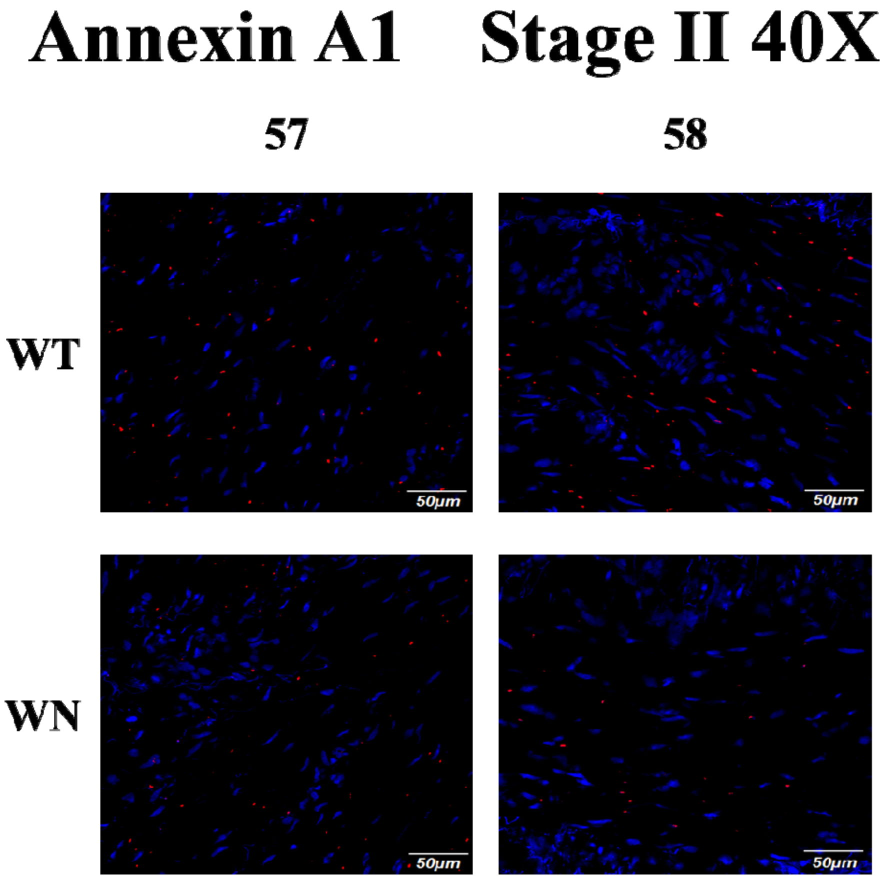
Annexin A1 Stage II 40X
57
58
WT
WN
50μm

Annexin A1 Stage III 40X

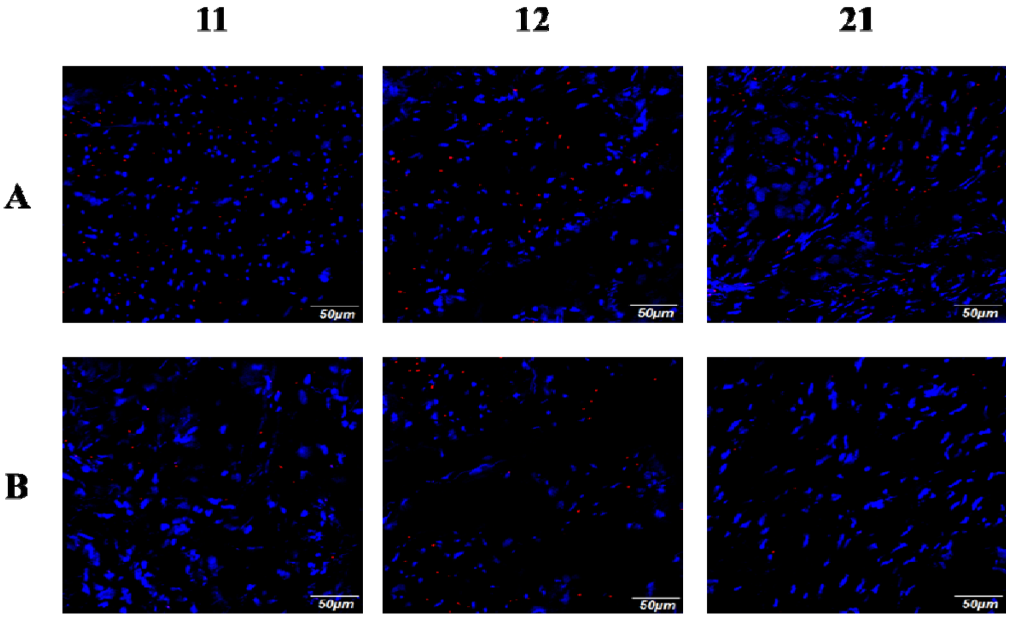

Annexin A1 Stage III 40X

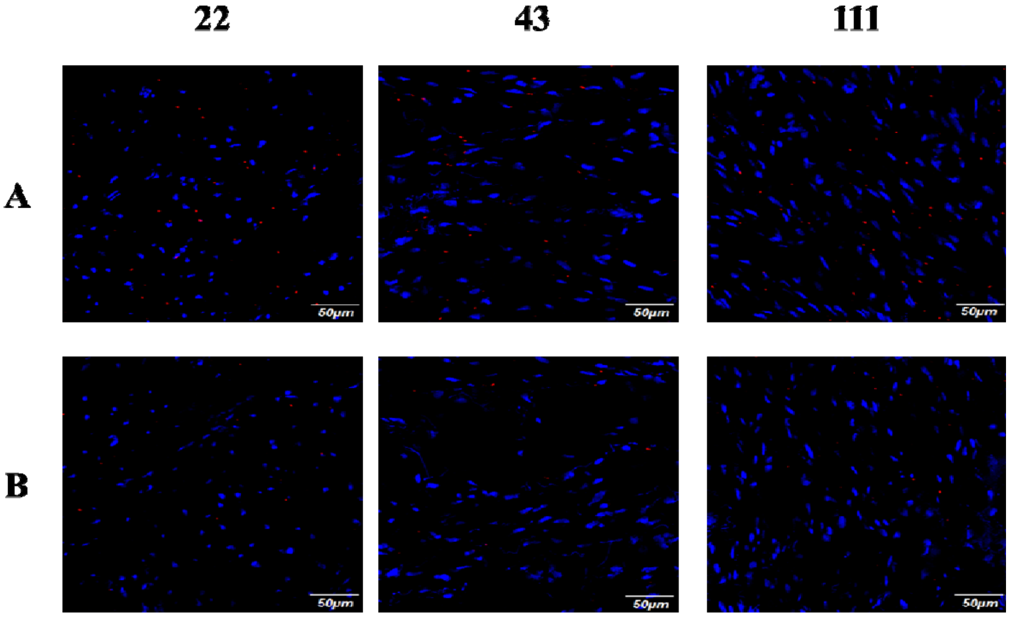

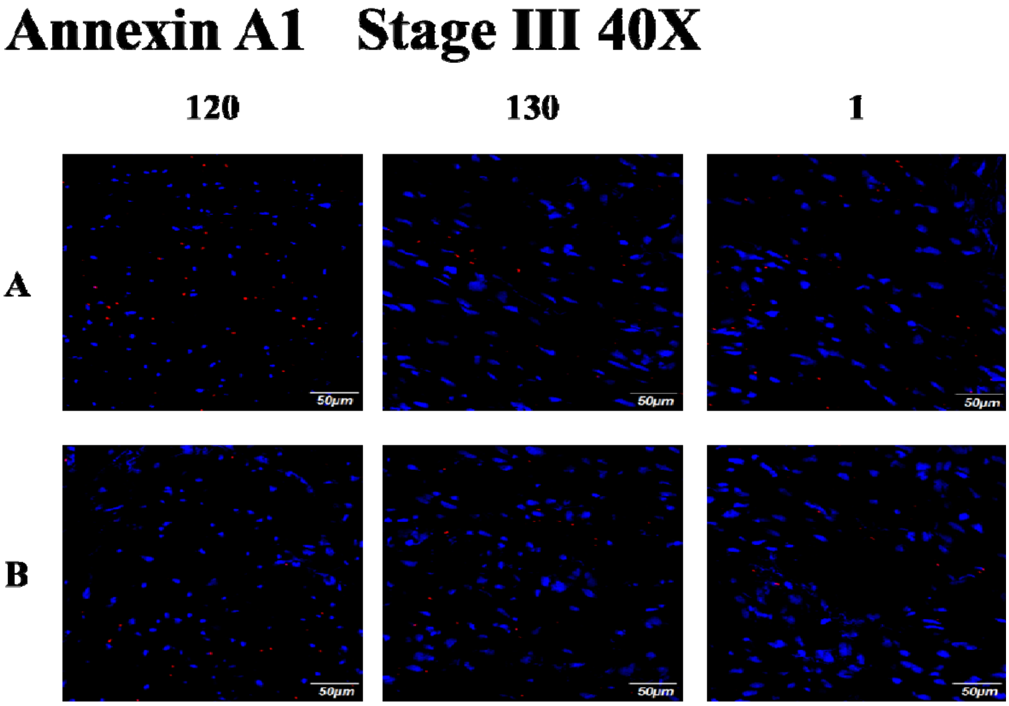
Annexin A1 Stage III 40X
120
130
1
A
B
50μm

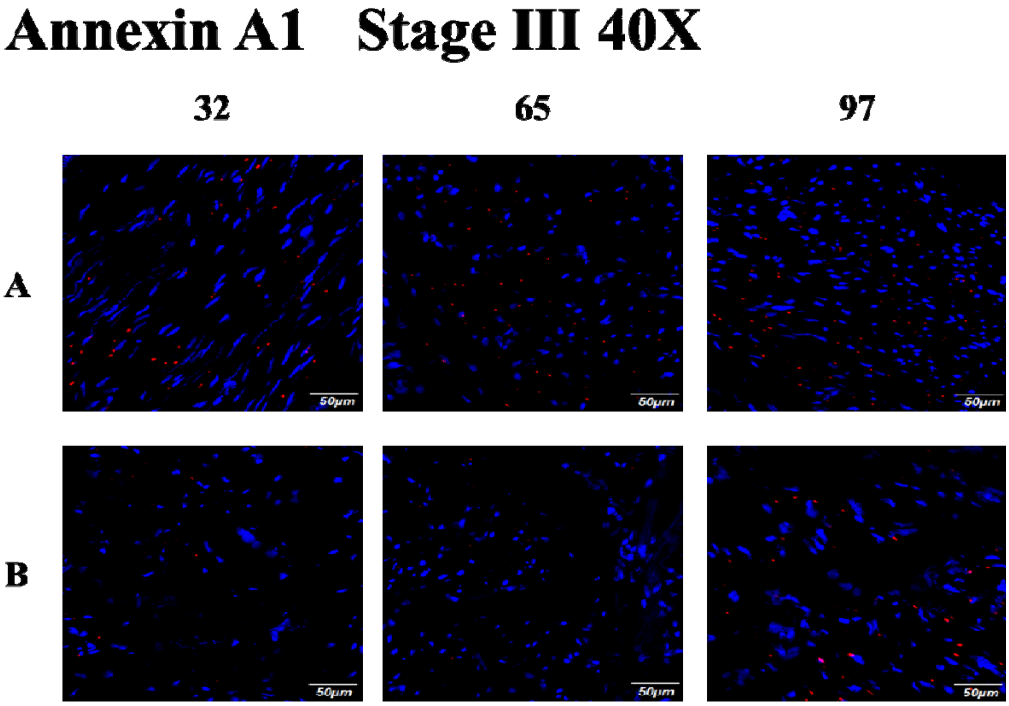
Annexin A1 Stage III 40X
32
65
97
A
B
50μm

Annexin A1 Stage III 40X

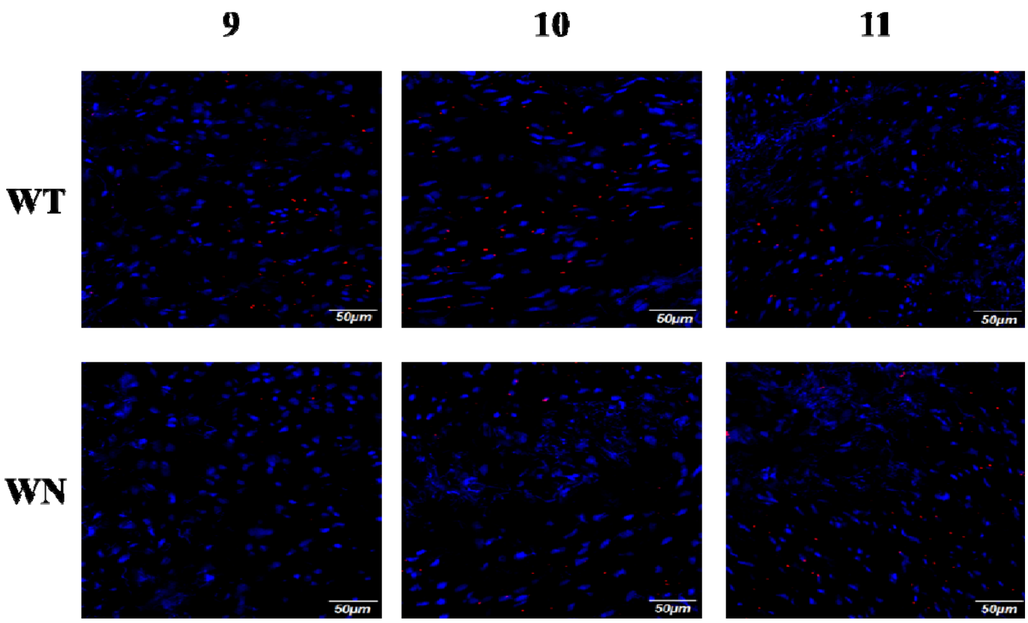

Annexin A1 Stage III 40X

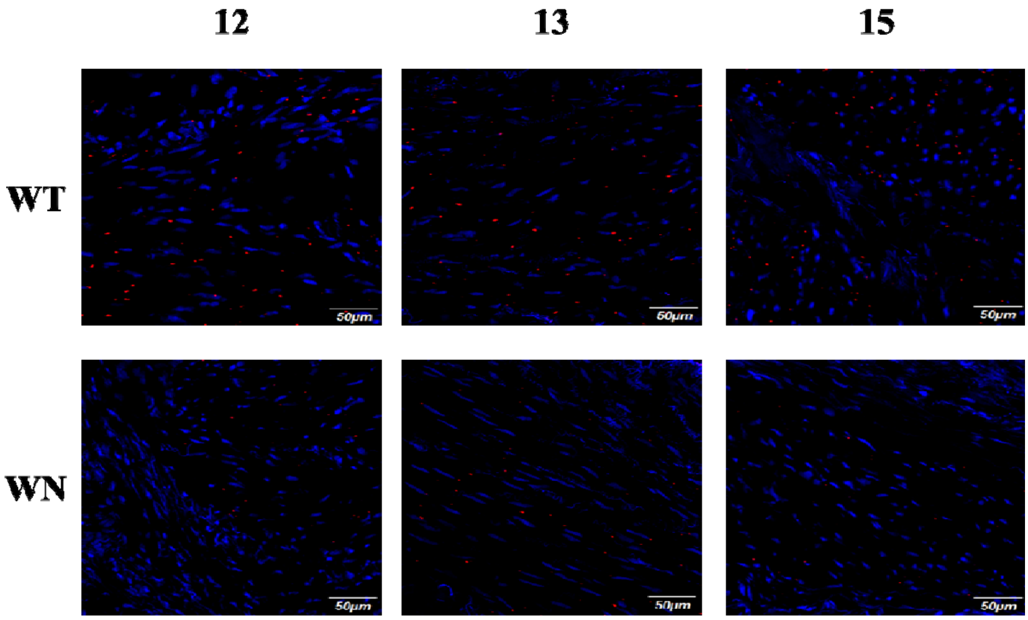

Annexin A1 Stage III 40X

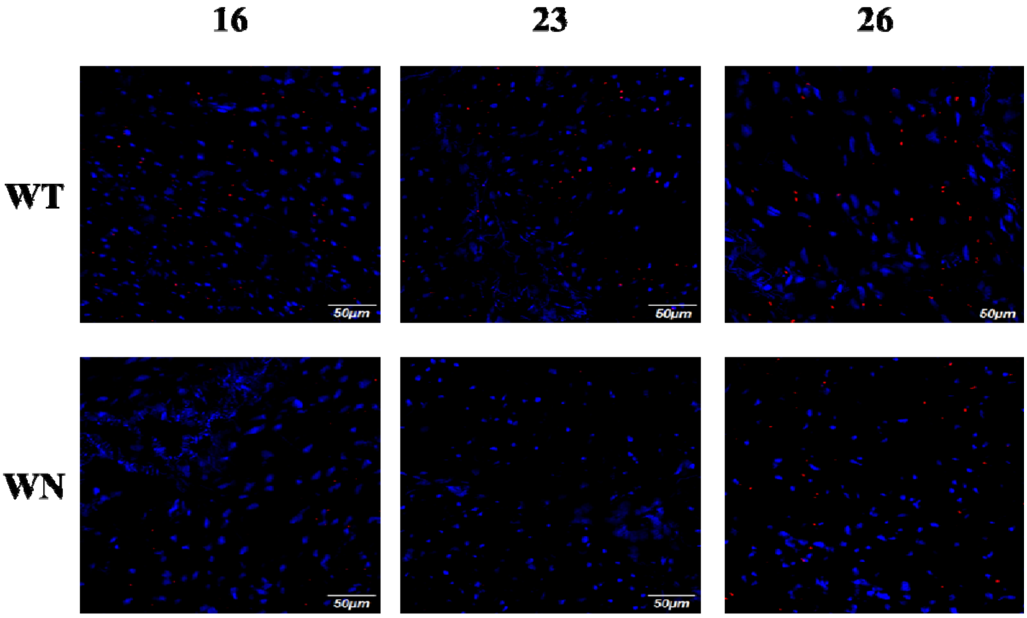

Annexin A1 Stage III 40X

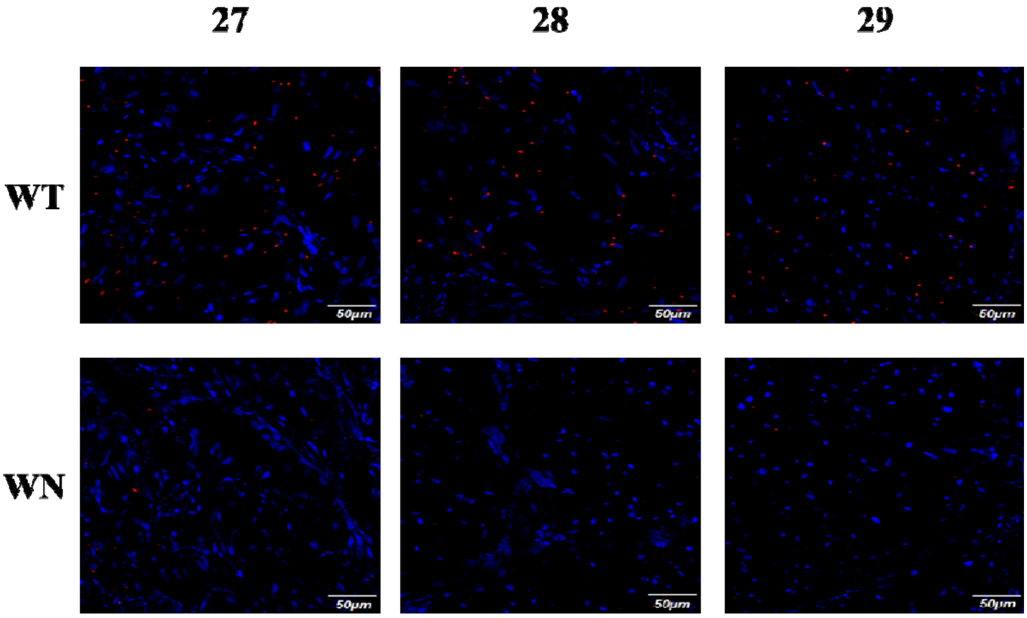

Annexin A1 Stage III 40X

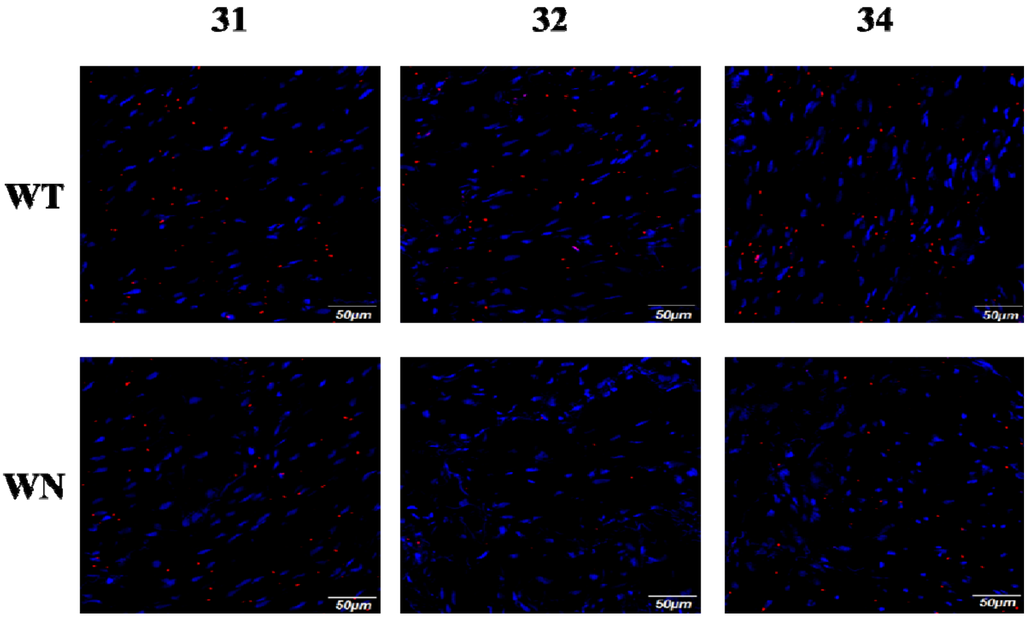

Annexin A1 Stage III 40X

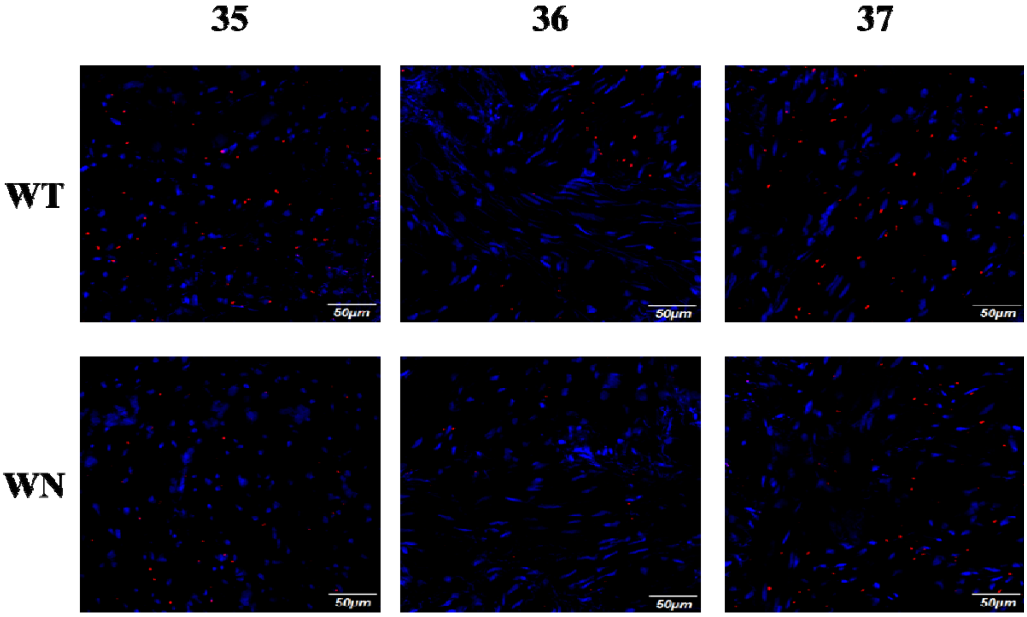

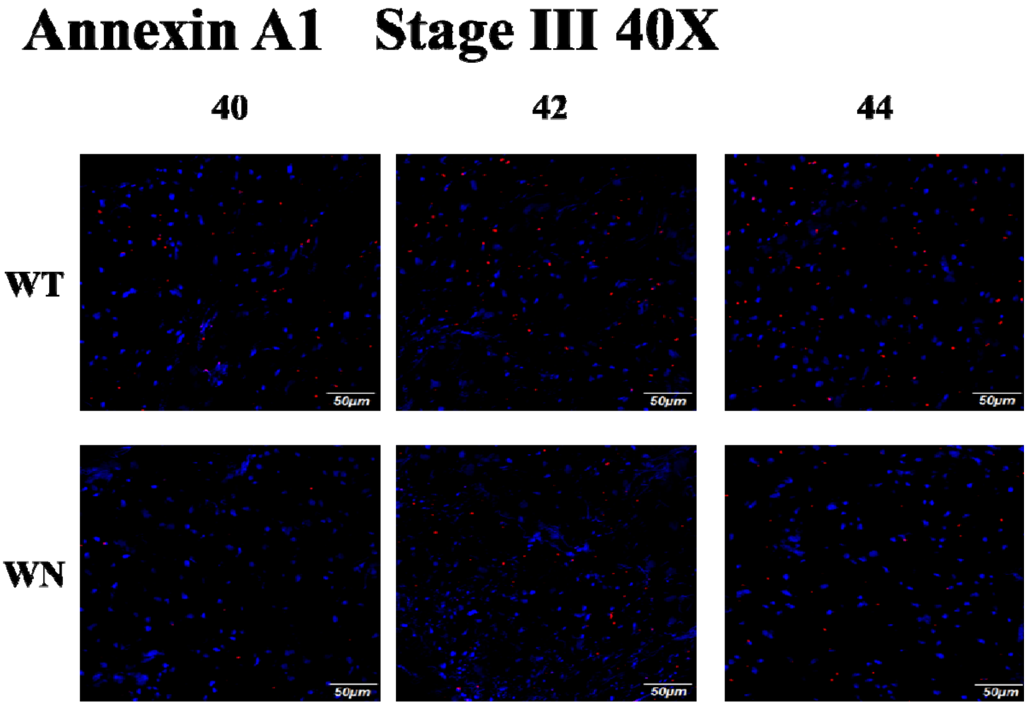
Annexin A1 Stage III 40X
40
42
44
WT
WN
50μm

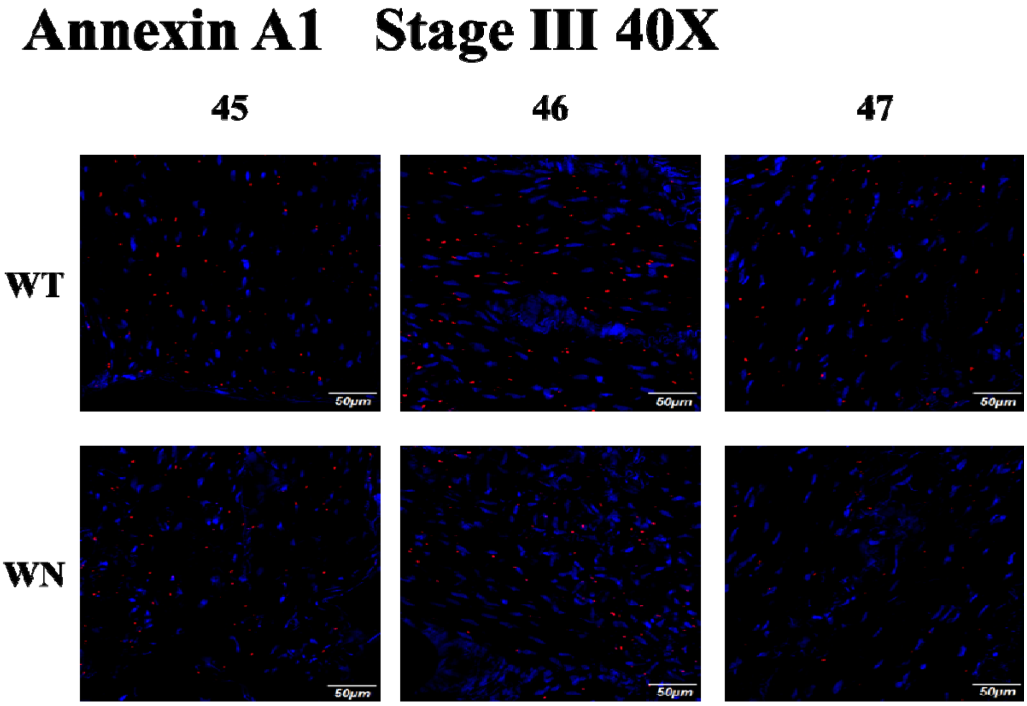
Annexin A1 Stage III 40X
45
46
47
WT
WN
50μm

Annexin A1 Stage III 40X

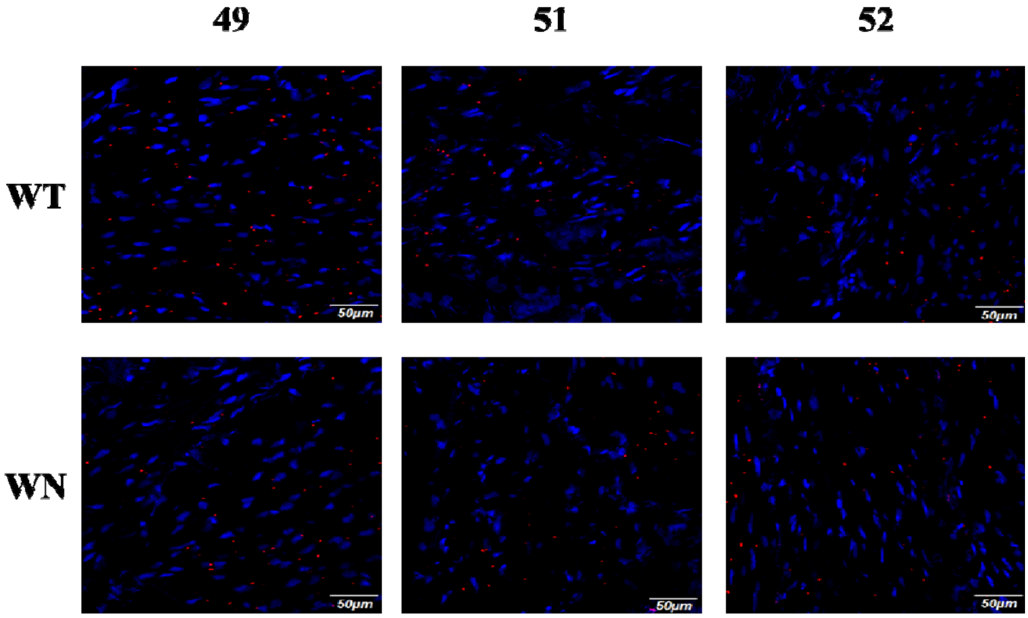

Annexin A1 Stage III 40X

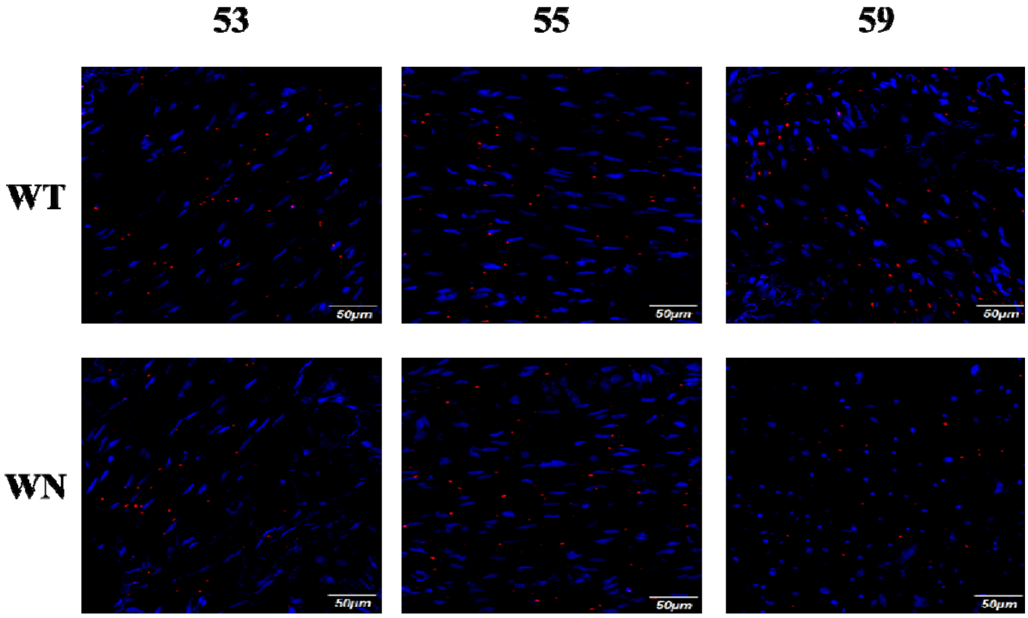

Annexin A1 Stage III 40X

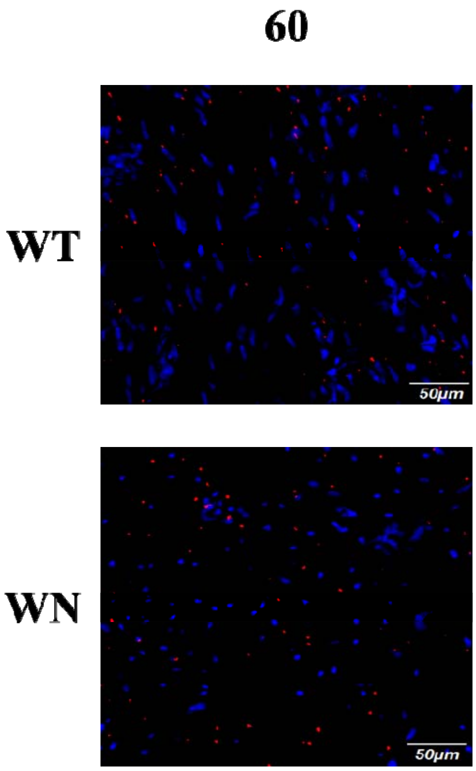

Annexin A1 Stage IV 40X

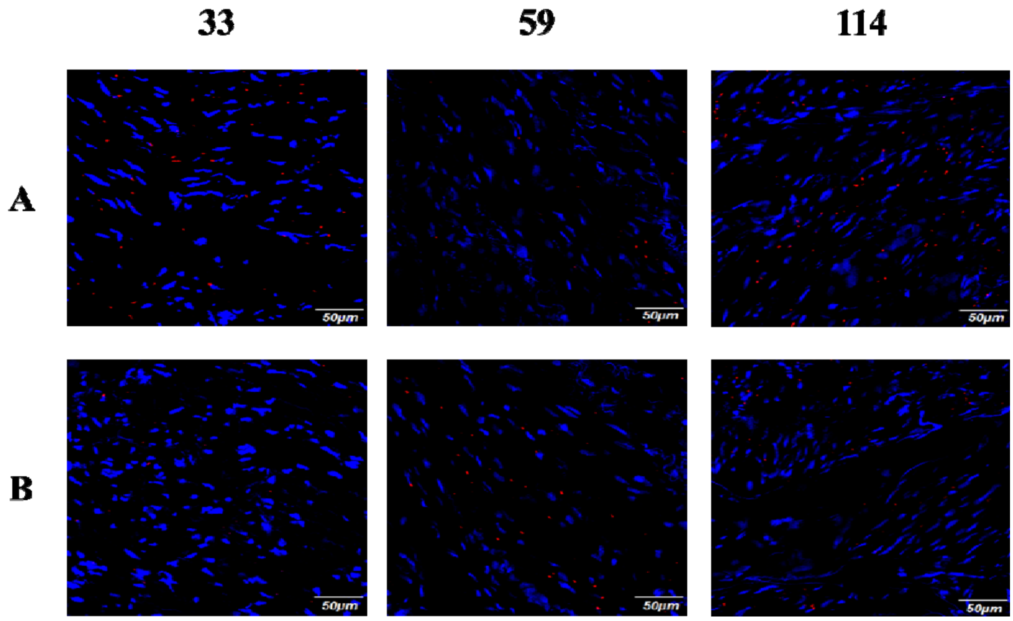

Annexin A1 Stage IV 40X

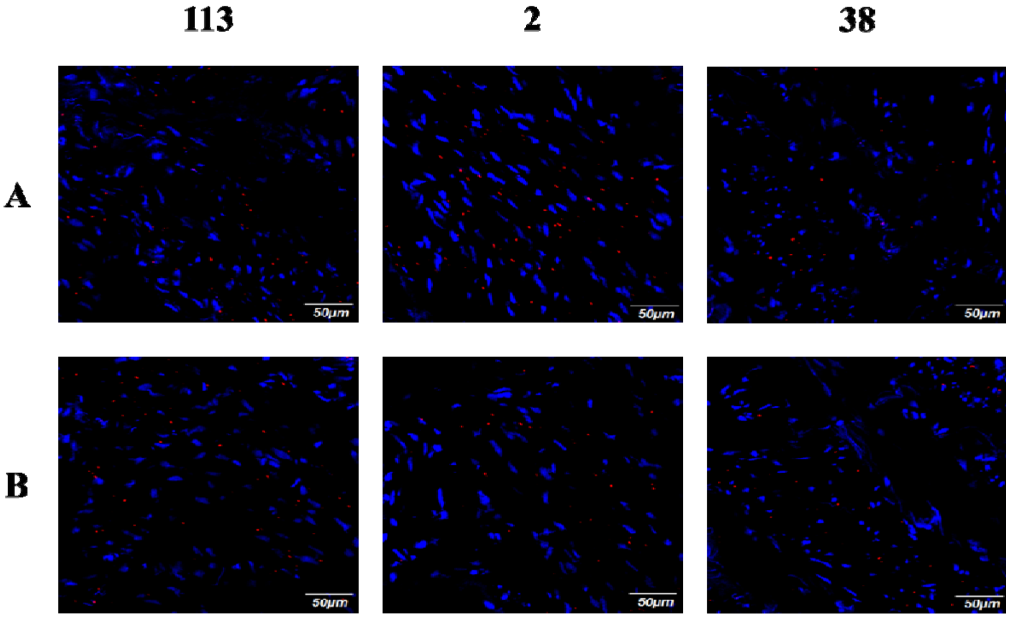

Annexin A1 Stage IV 40X

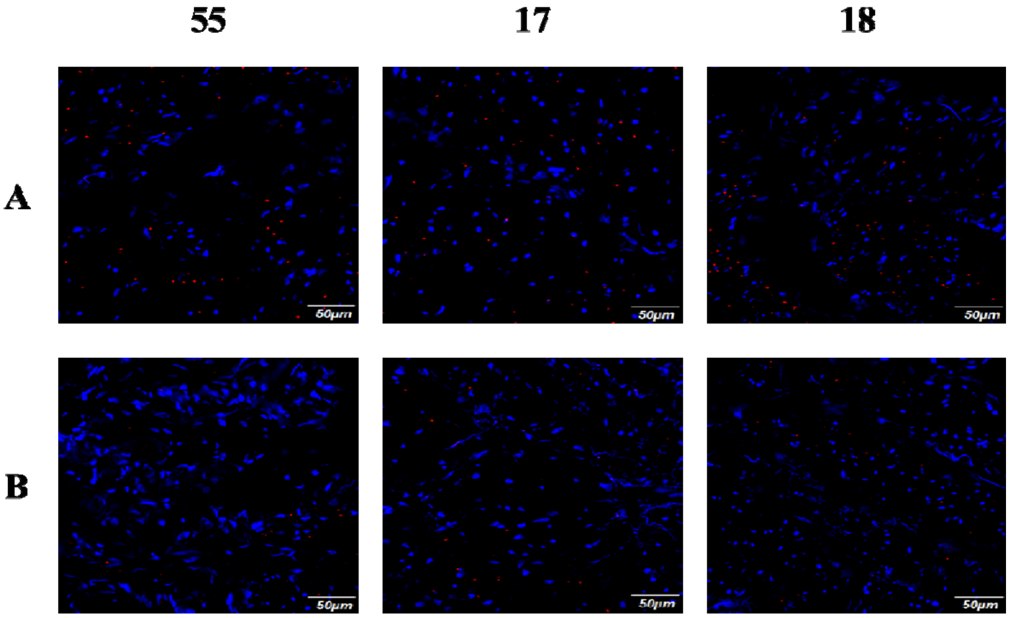

Annexin A1   Stage IV 40X

112

A

B

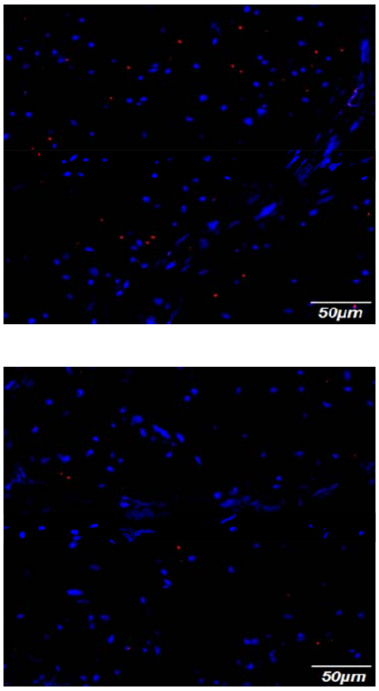

NNMT   Stage I 40X

7   **70**   **118**

A

B

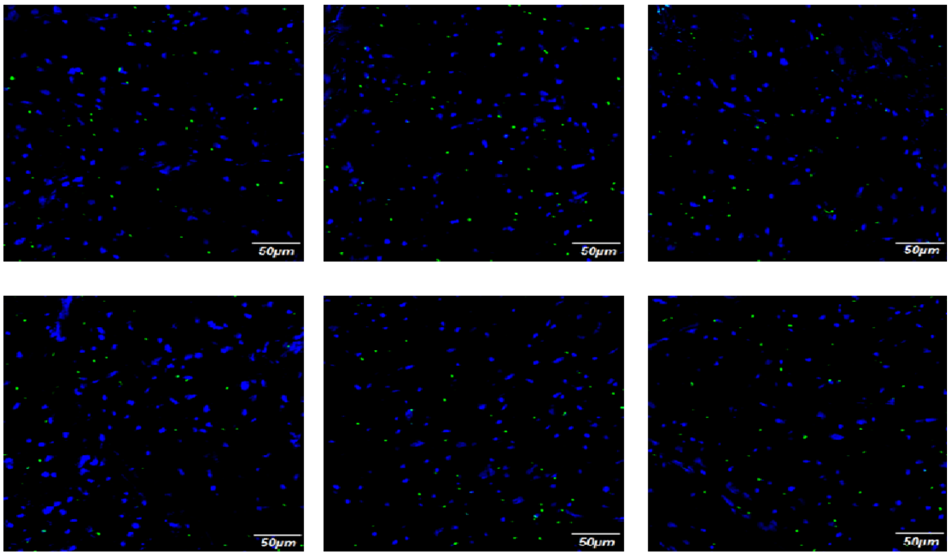

NNMT Stage I 40X

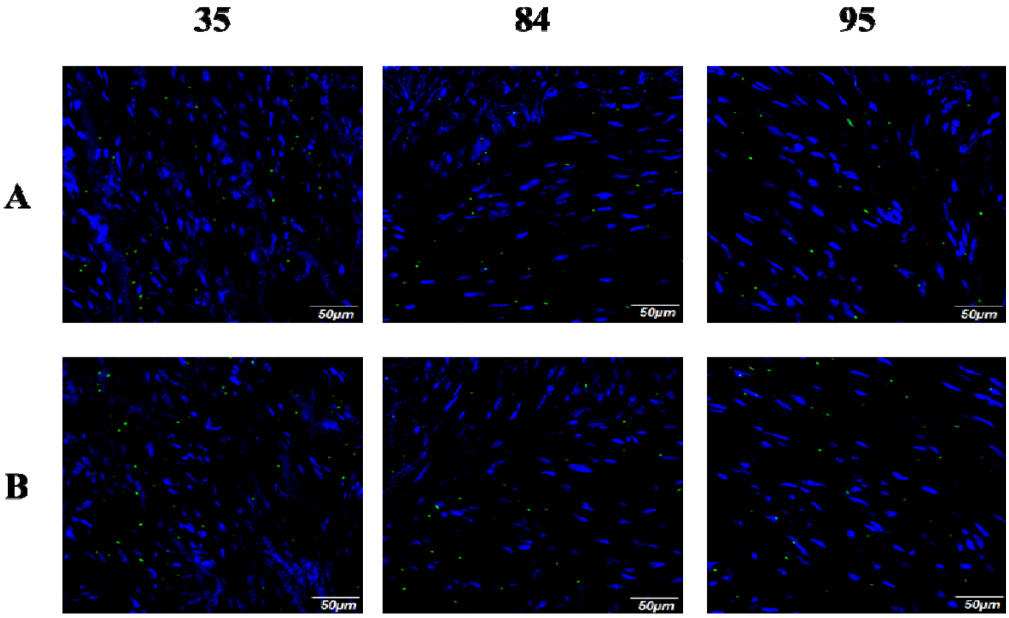

NNMT Stage I 40X

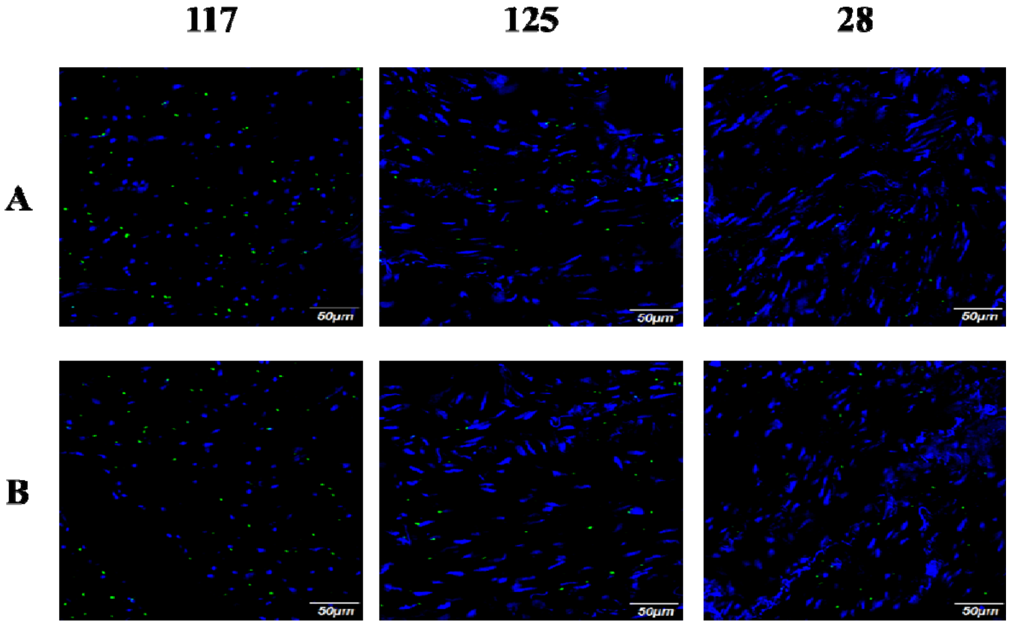

NNMT Stage I 40X

56

A

B

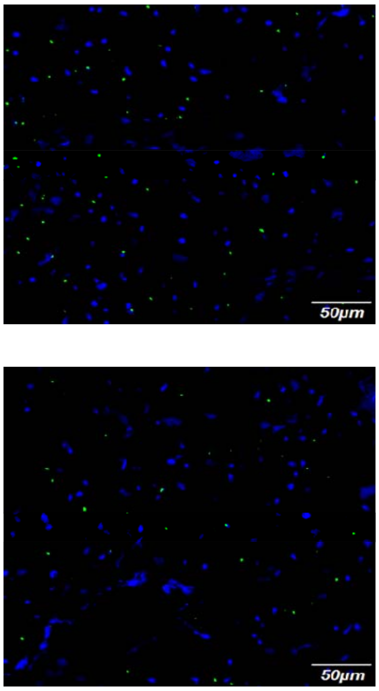

NNMT Stage I 40X

1 4 25

WT

WN

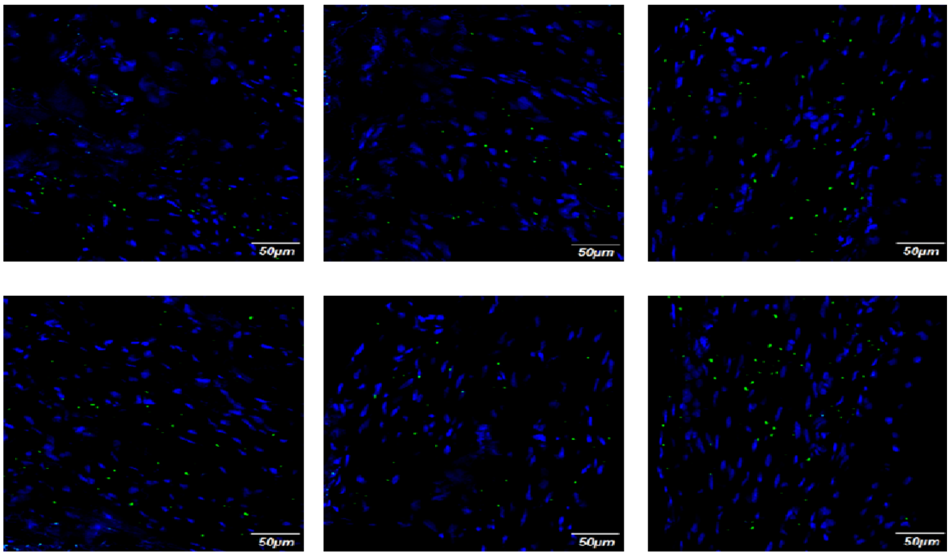

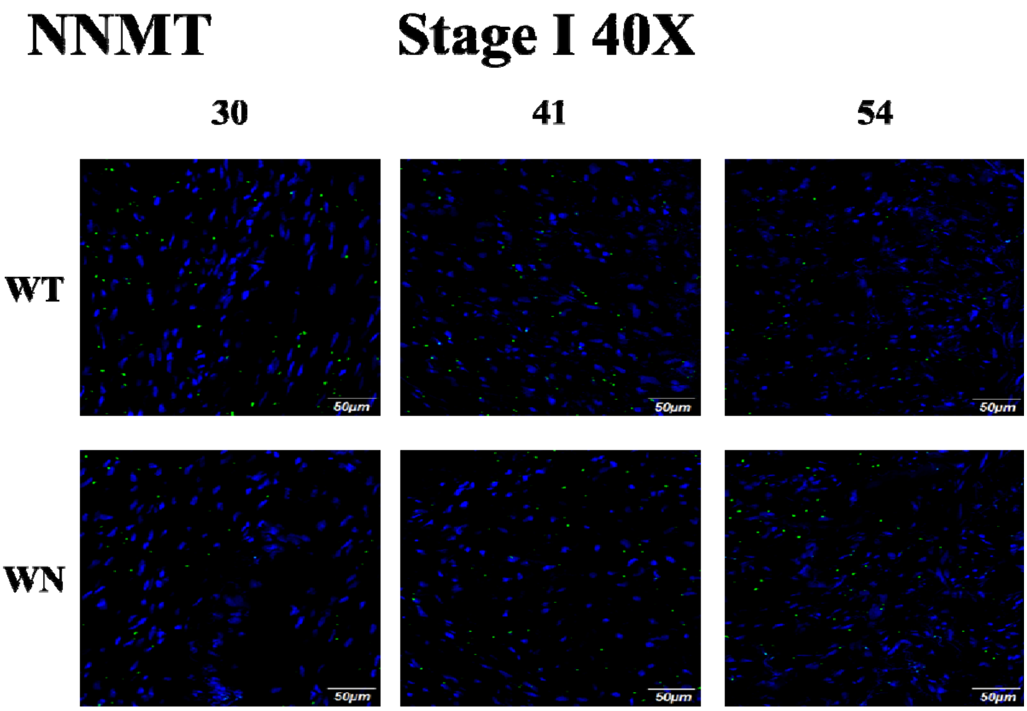
NNMT
Stage I 40X
30
41
54
WT
WN
50μm

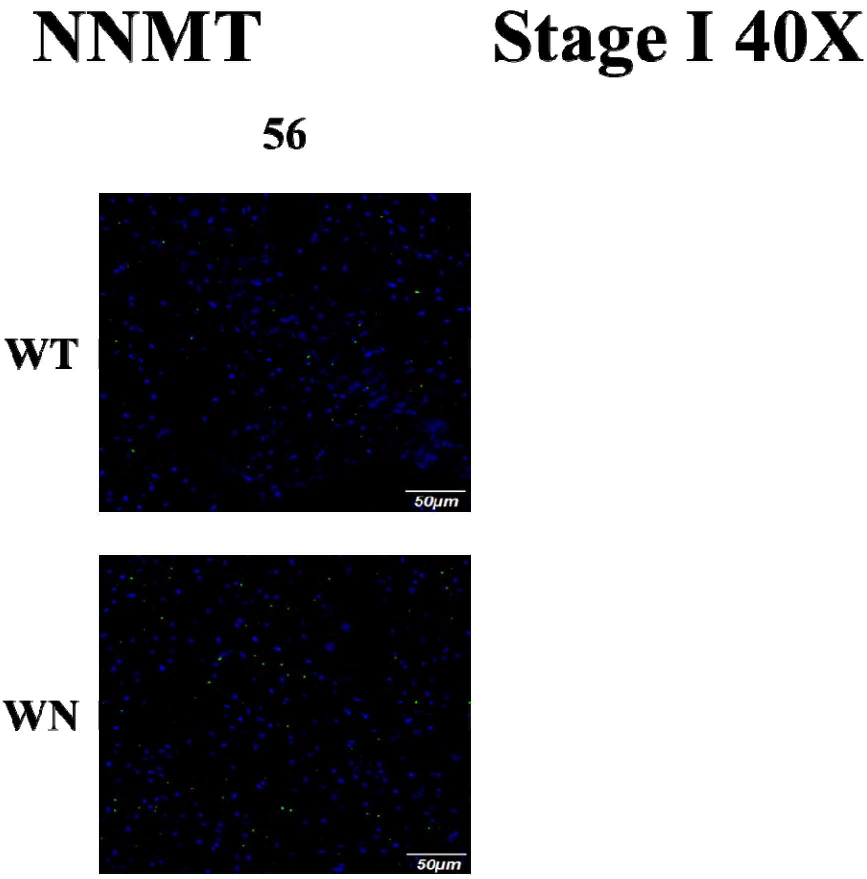
NNMT
Stage I 40X
56
WT
WN
50μm

NNMT Stage II 40X

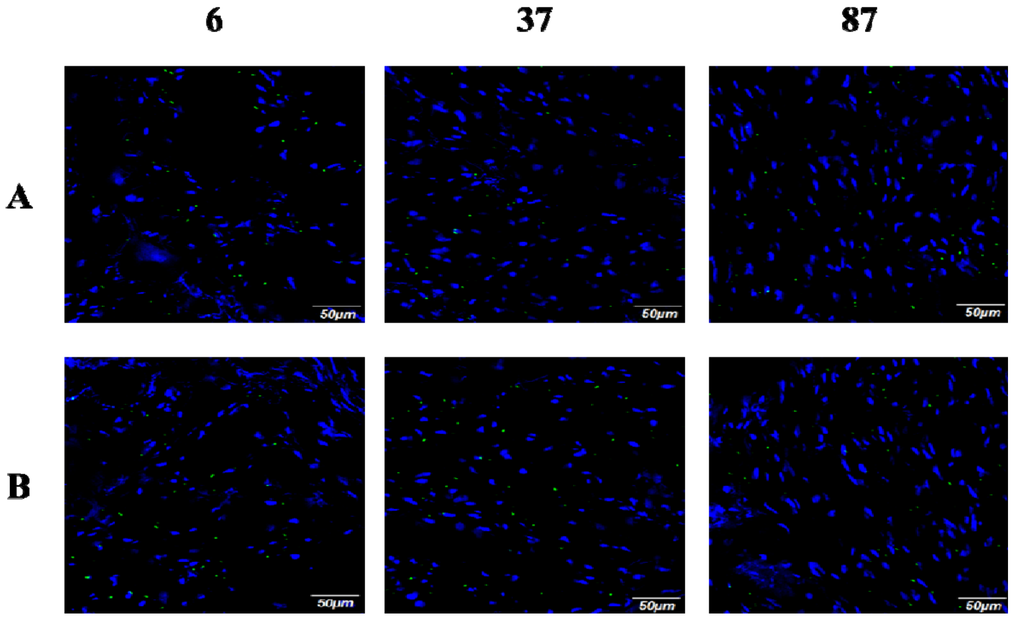

NNMT Stage II 40X

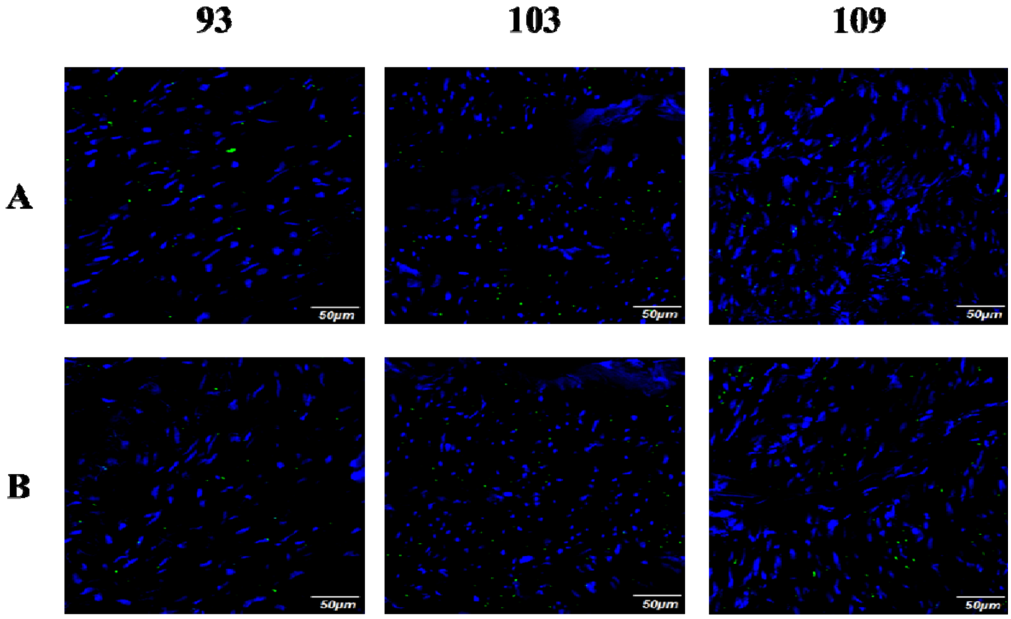

NNMT Stage II 40X

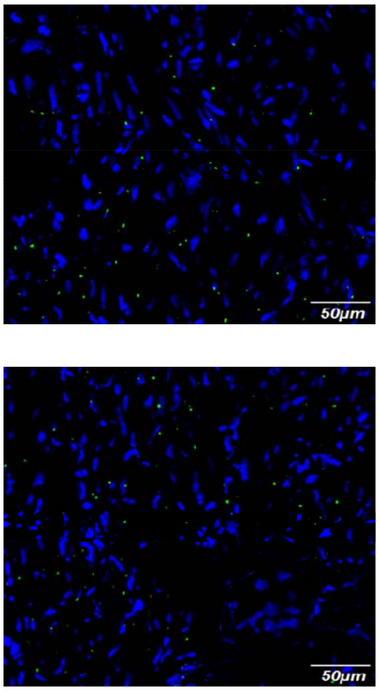

NNMT Stage II 40X

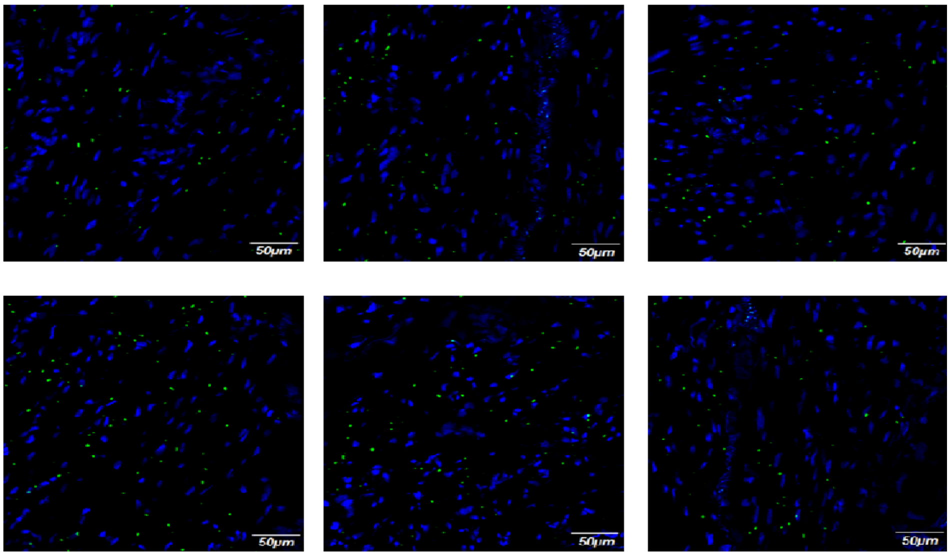

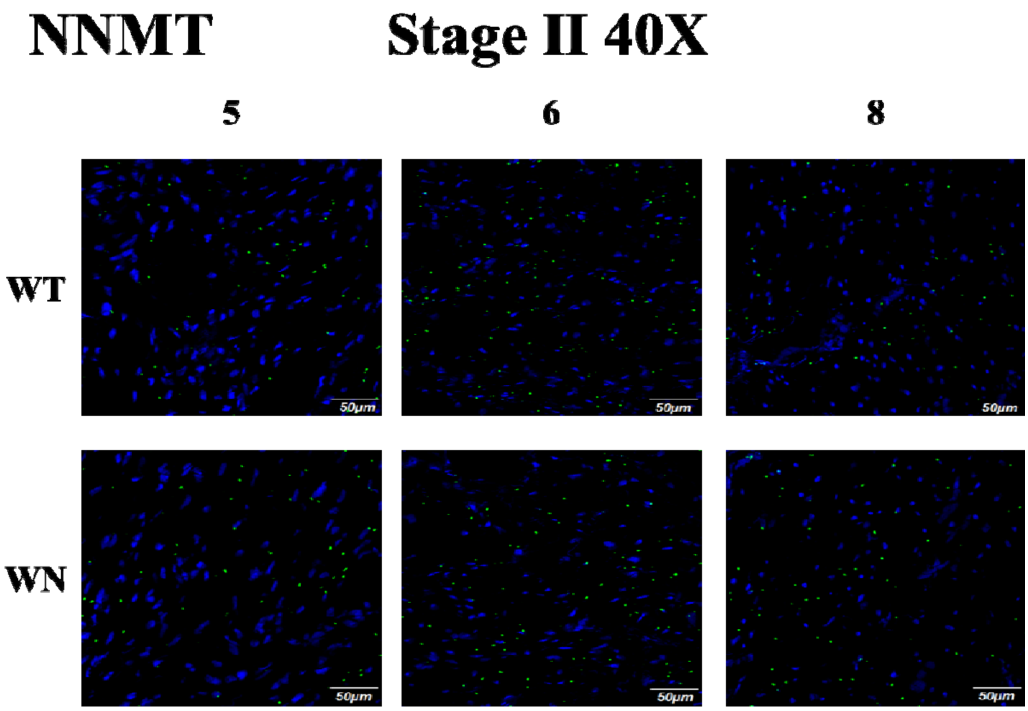
NNMT
Stage II 40X
5
6
8
WT
WN
50μm

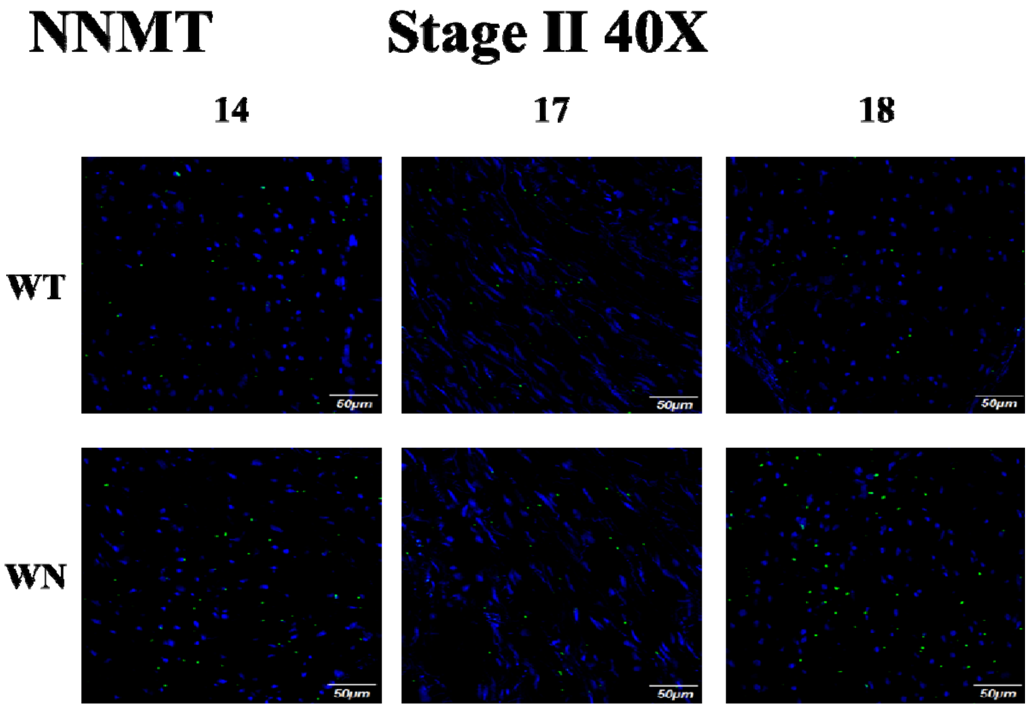
NNMT
Stage II 40X
14
17
18
WT
WN
50μm

NNMT Stage II 40X

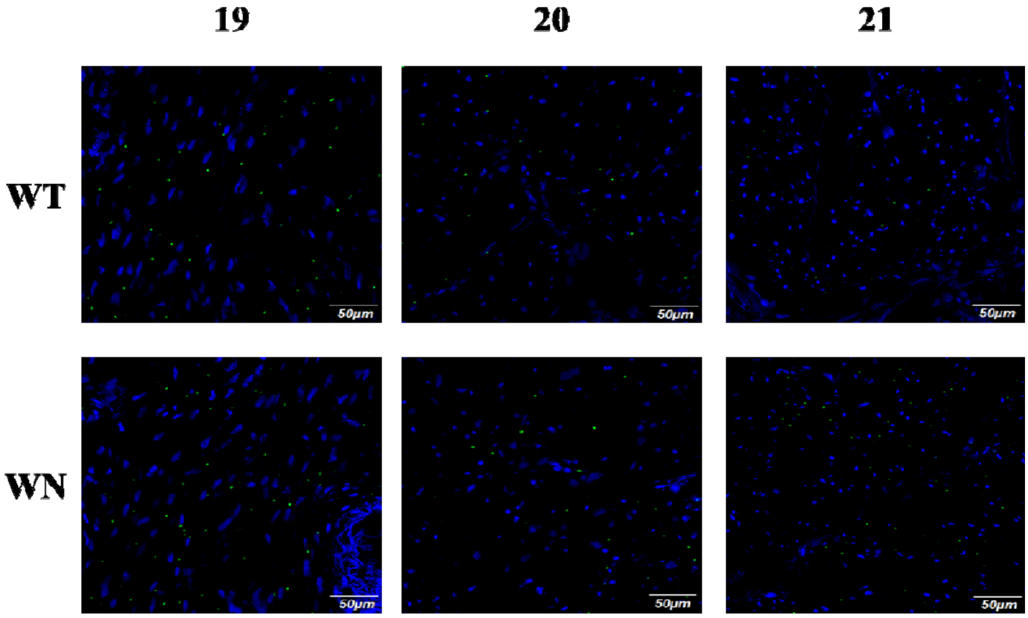

NNMT Stage II 40X

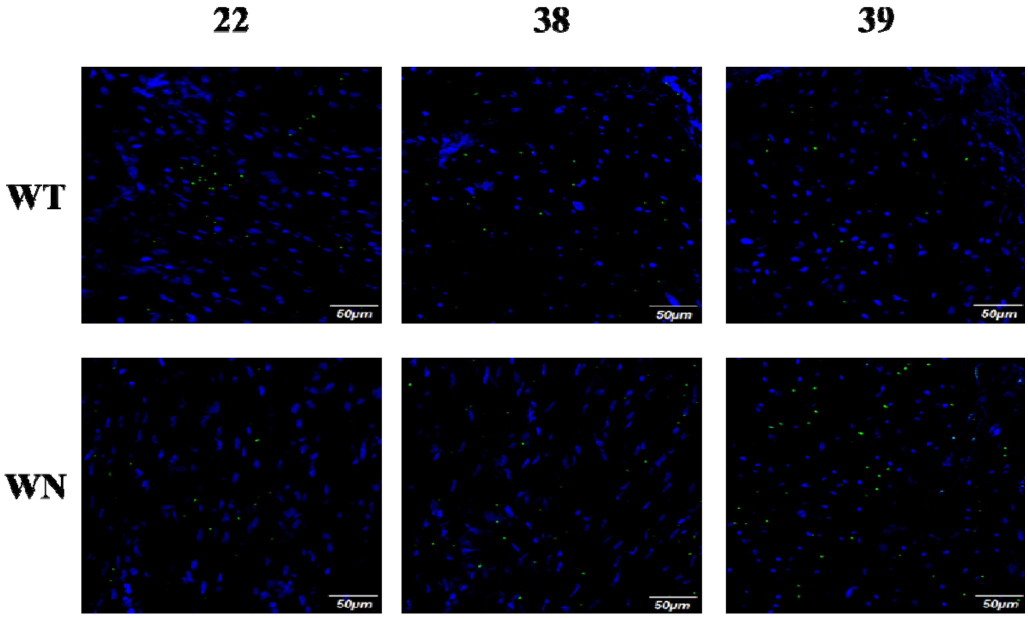

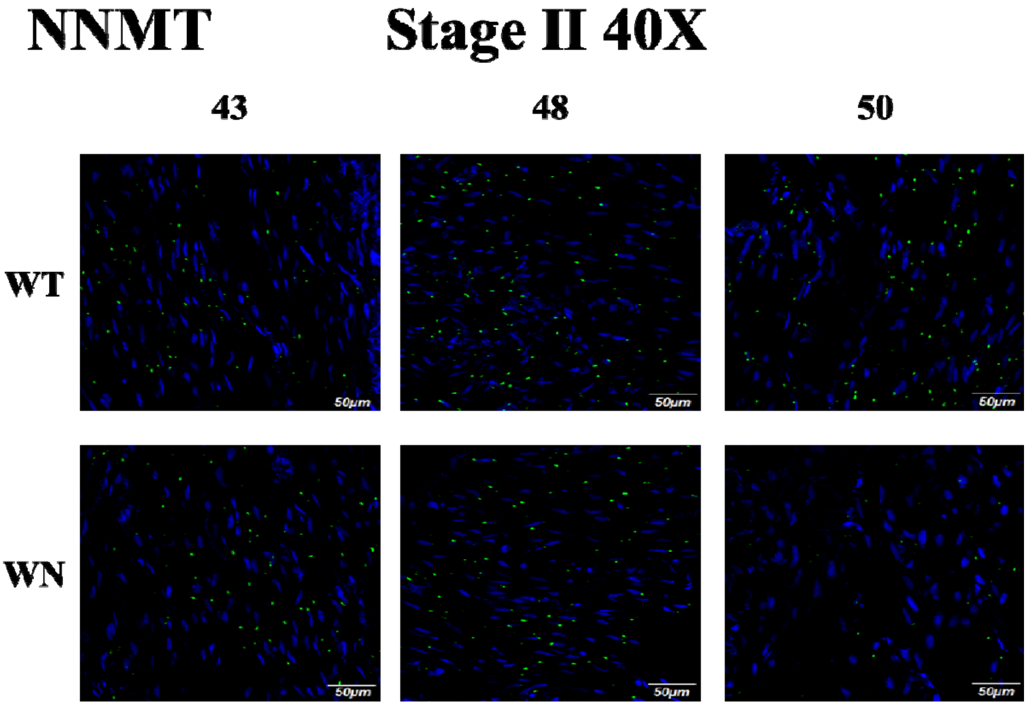
NNMT
Stage II 40X
43
48
50
WT
WN
50μm

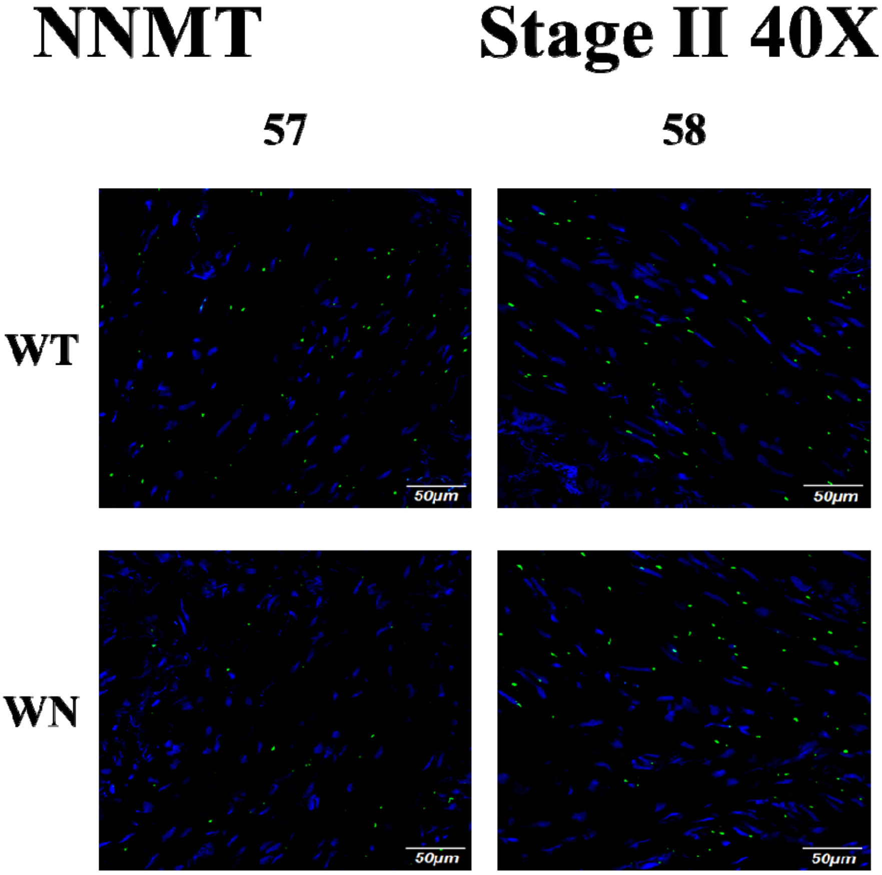
NNMT
Stage II 40X
57
58
WT
WN
50μm

NNMT Stage III 40X

11 12 21

A

B

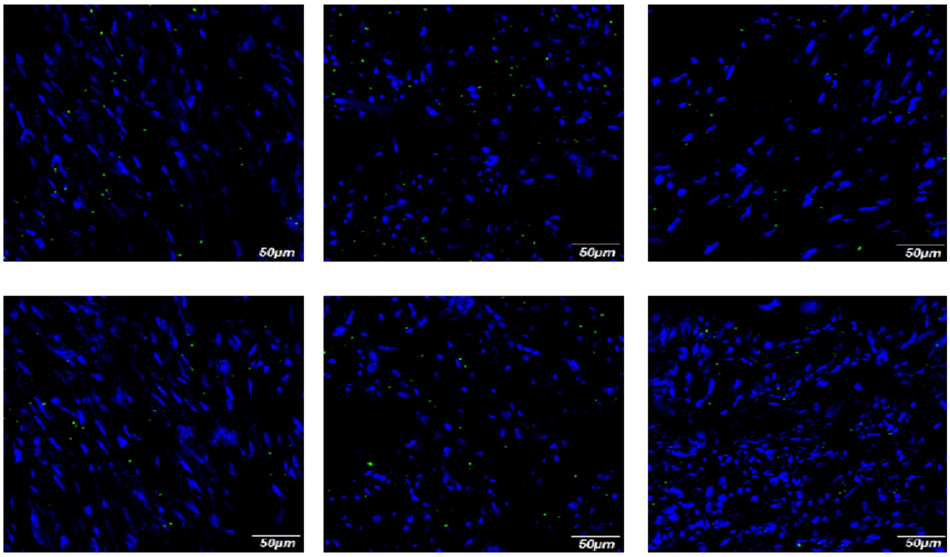

NNMT Stage III 40X

22 43 111

A

B

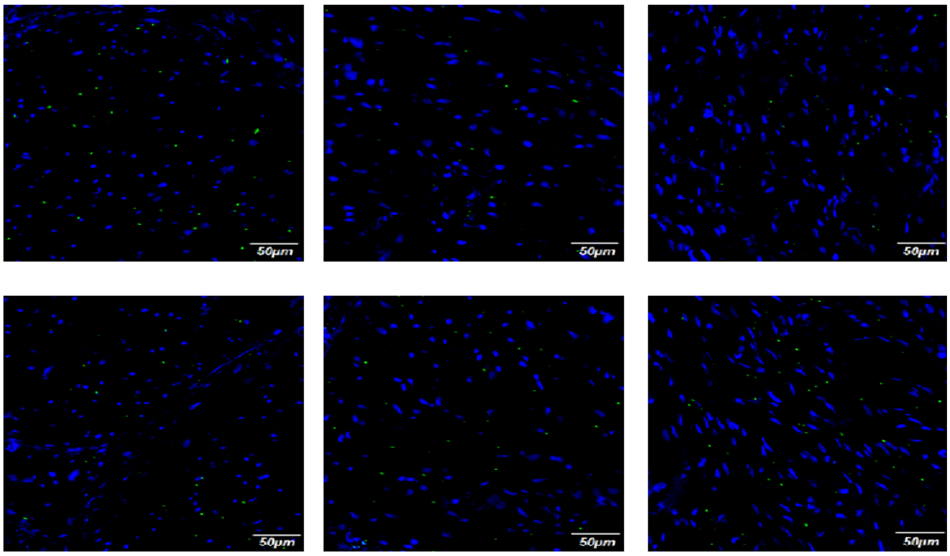

NNMT Stage III 40X

120 130 1

A

B

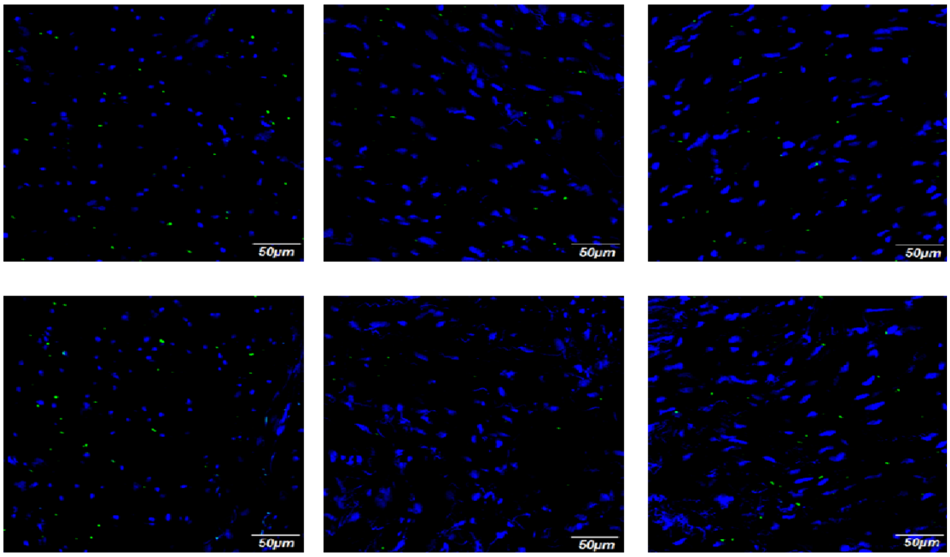

NNMT Stage III 40X

32 65 97

A

B

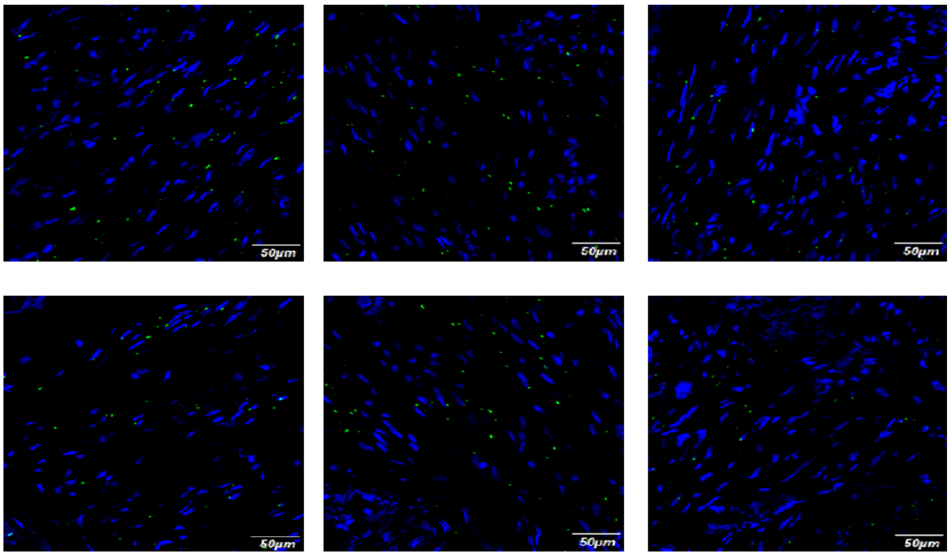

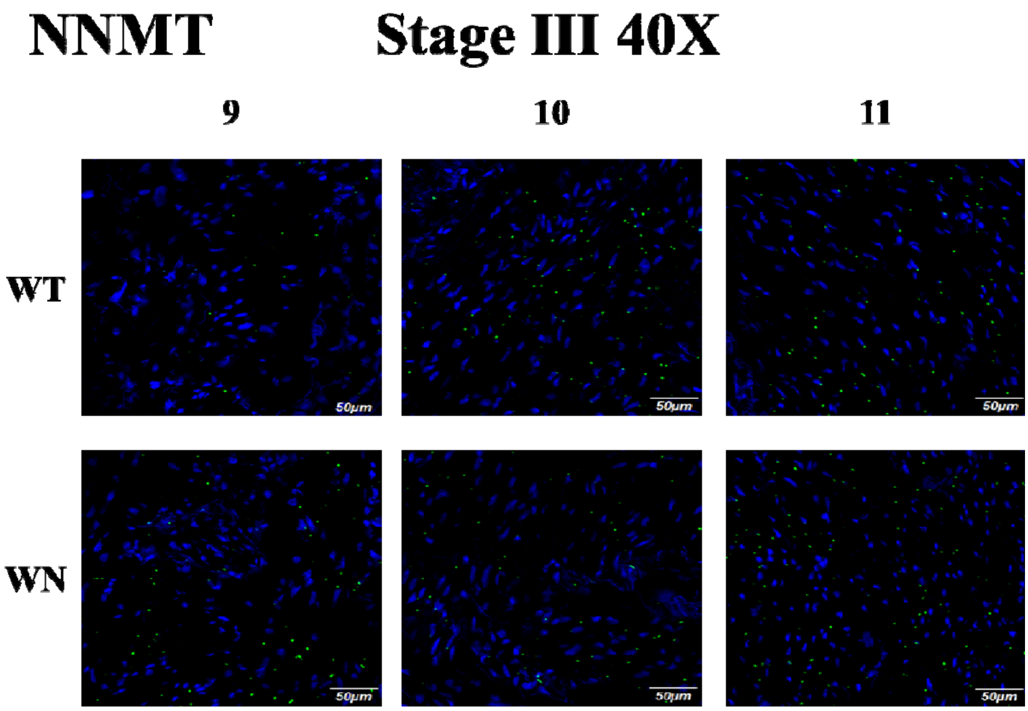
NNMT
Stage III 40X
9
10
11
WT
WN

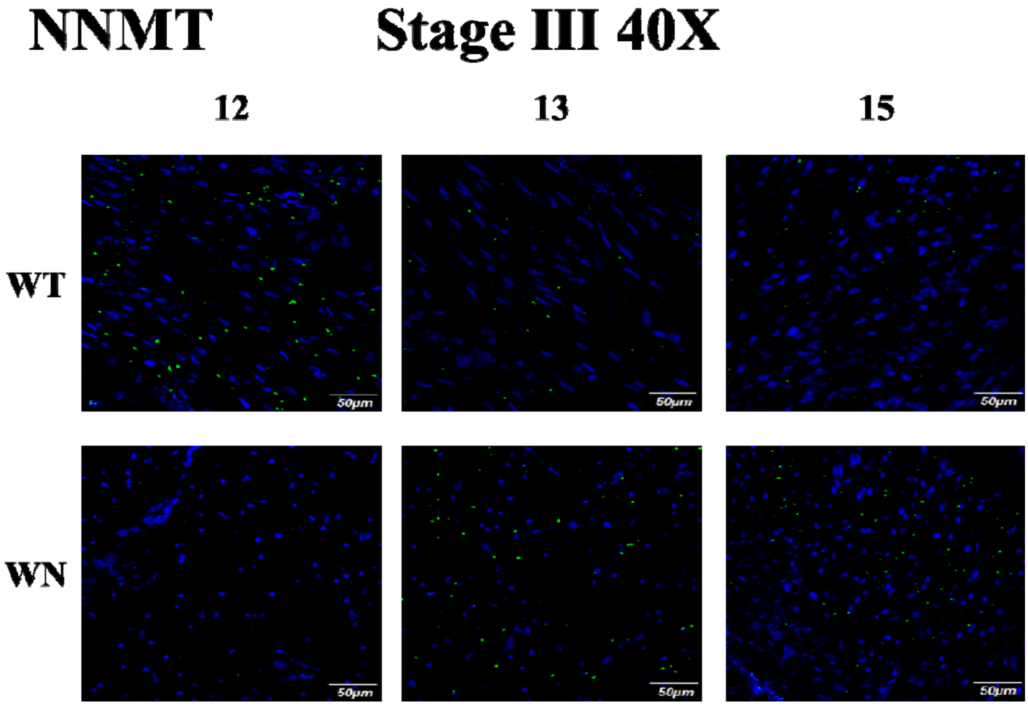
NNMT
Stage III 40X
12
13
15
WT
WN

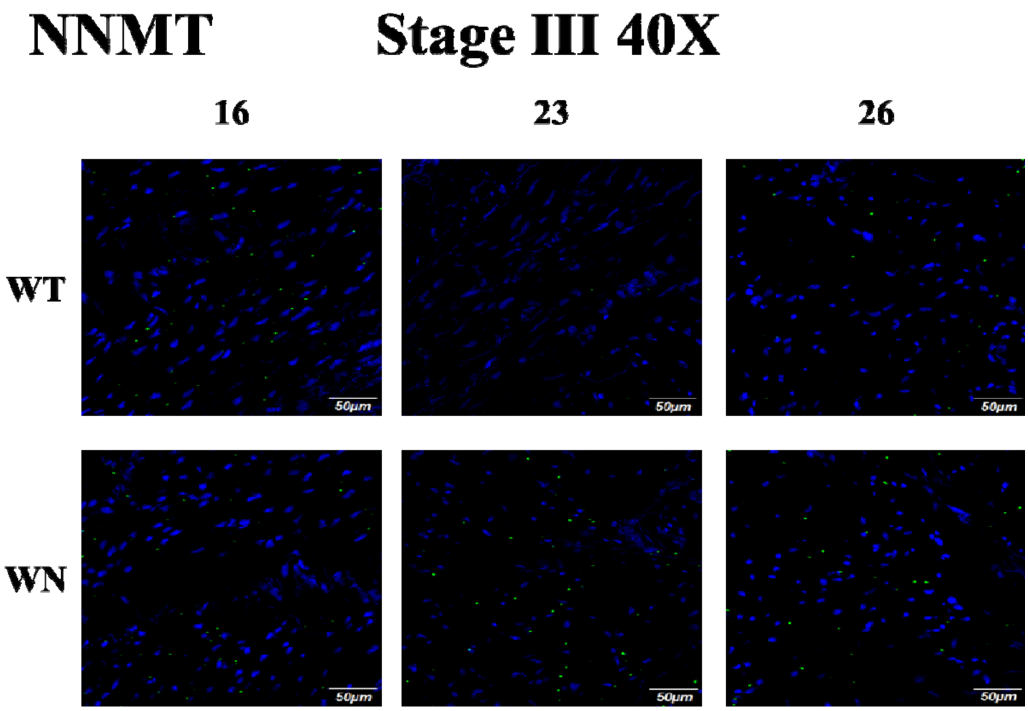
NNMT
Stage III 40X
16
23
26
WT
WN
50μm

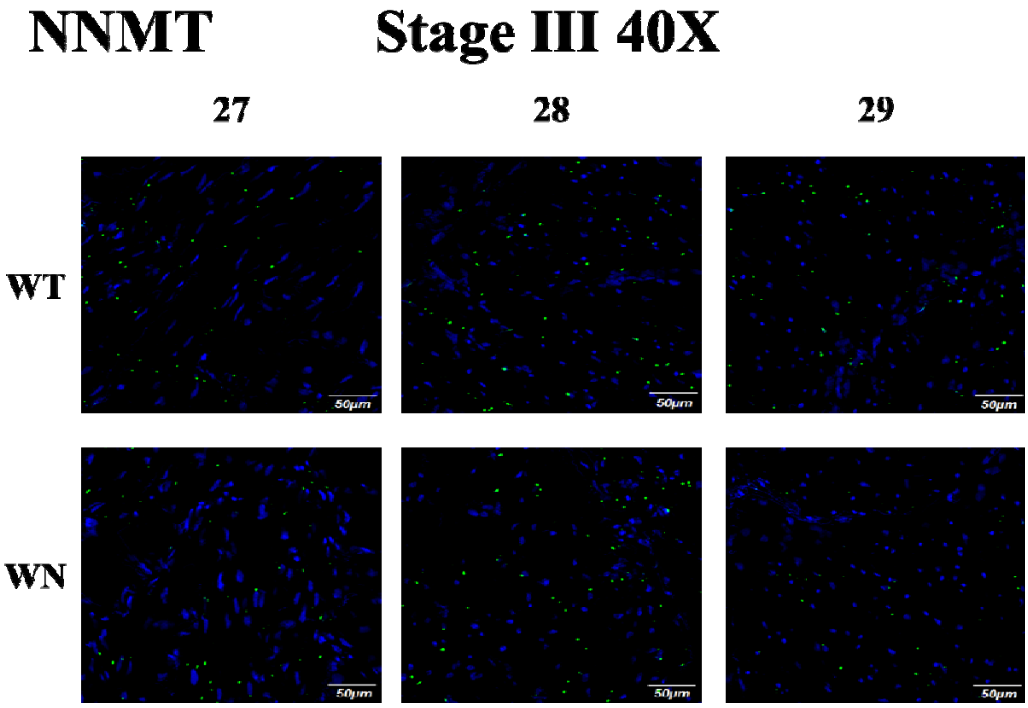
NNMT
Stage III 40X
27
28
29
WT
WN
50μm

NNMT Stage III 40X

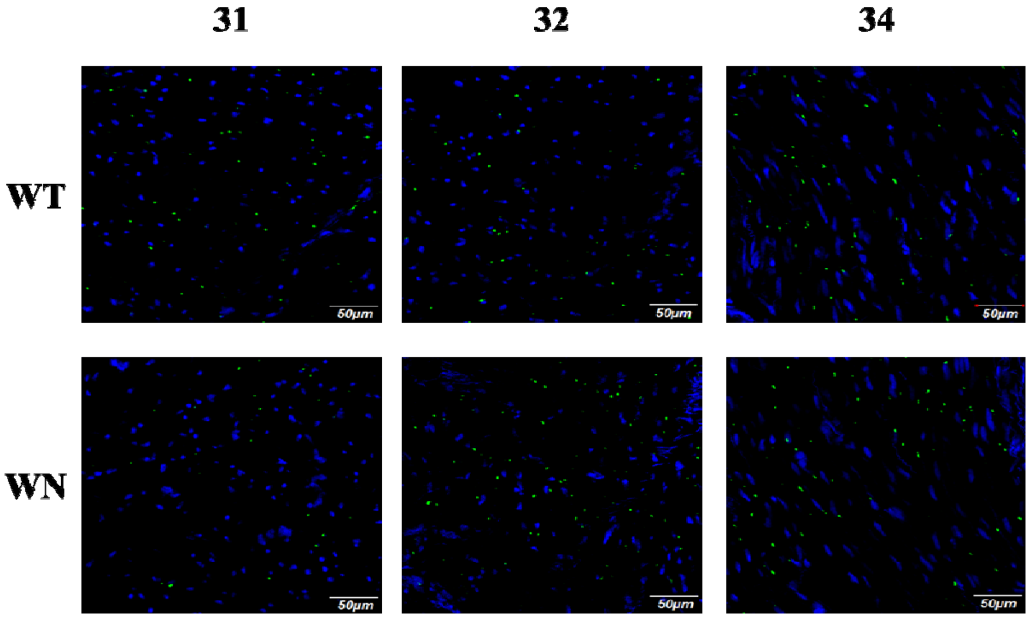

NNMT Stage III 40X

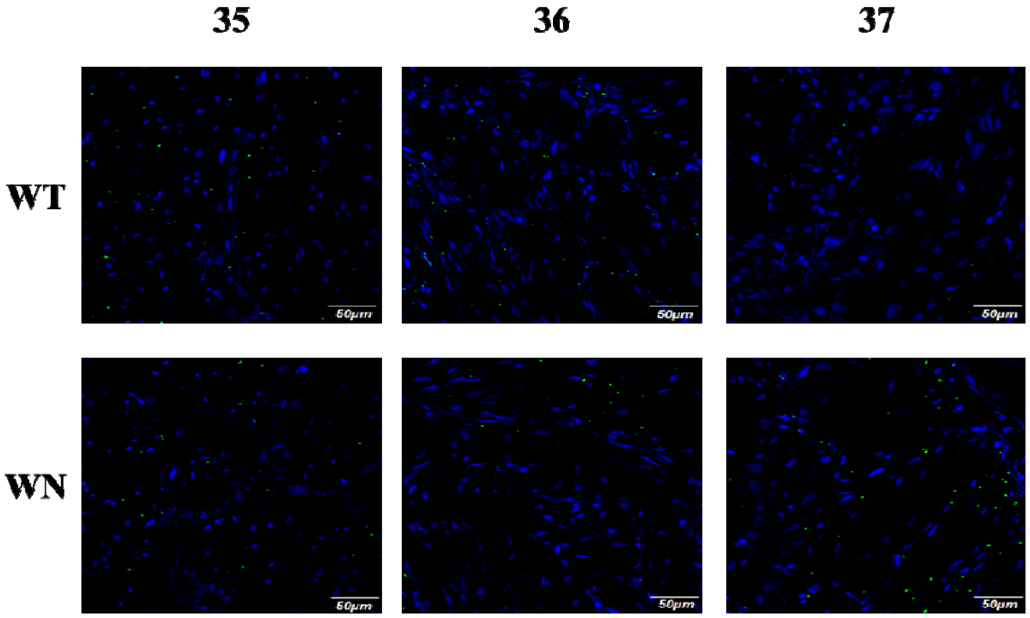

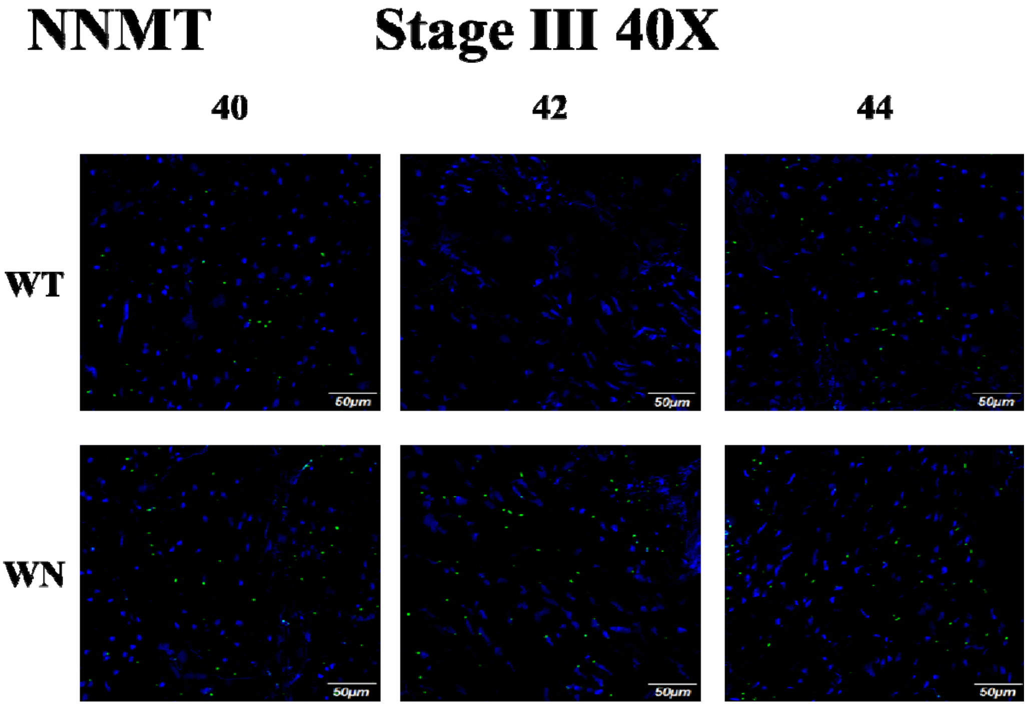
NNMT
Stage III 40X
40
42
44
WT
WN
50μm

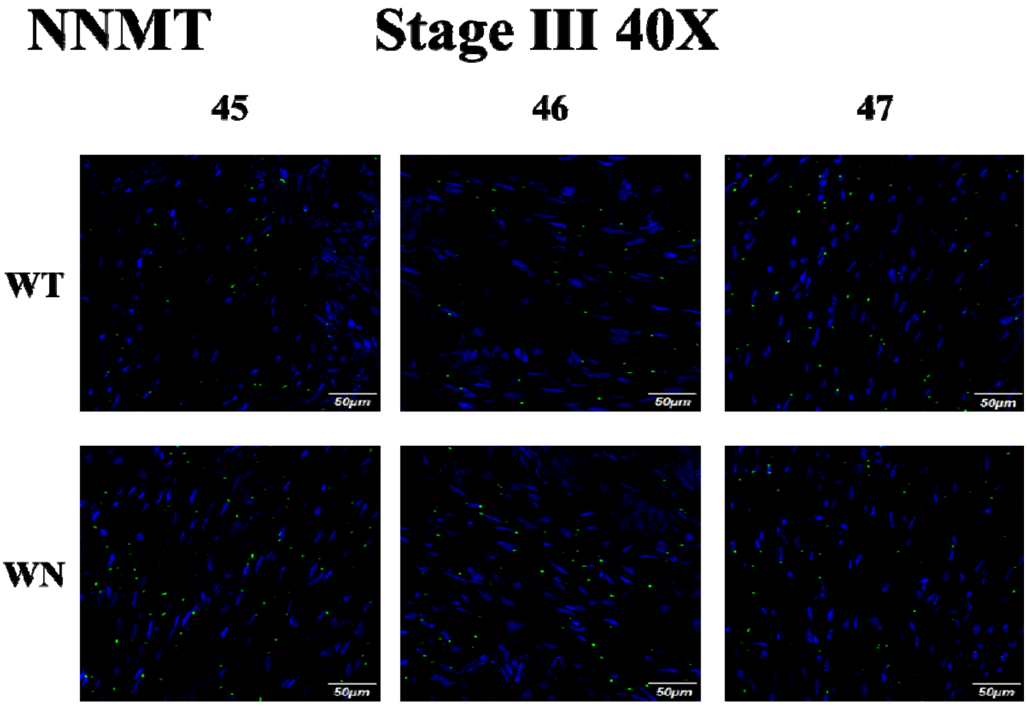
NNMT
Stage III 40X
45
46
47
WT
WN
50μm

NNMT Stage III 40X

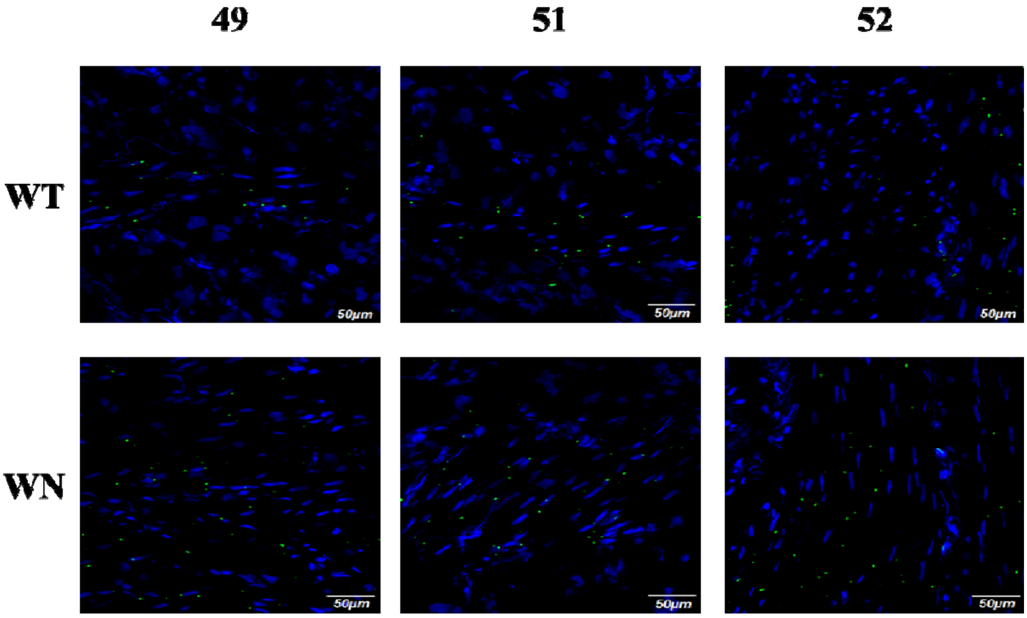

NNMT Stage III 40X

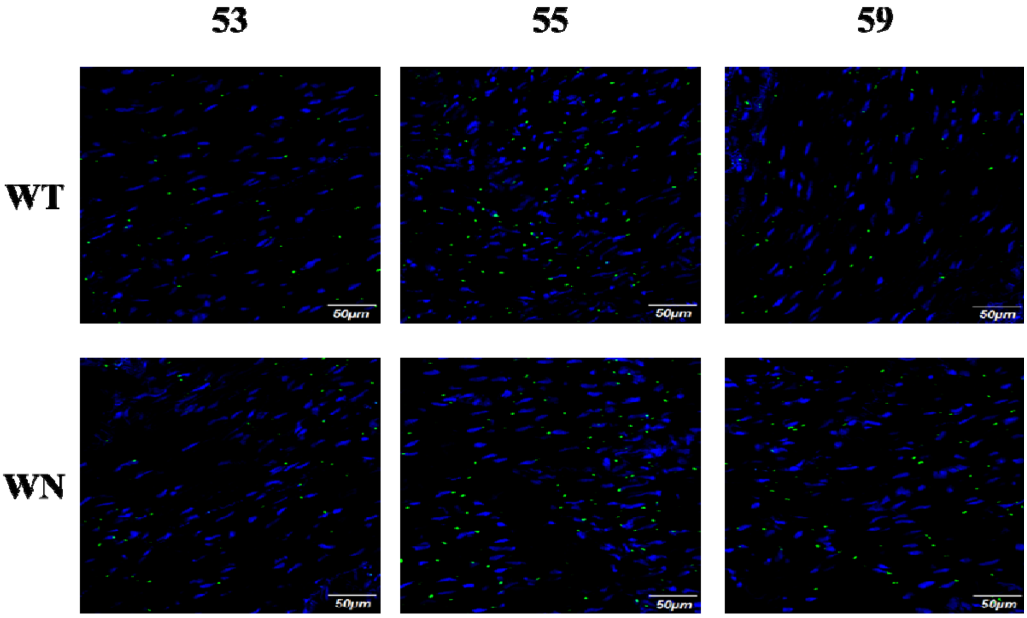

NNMT Stage III 40X

60

WT

WN

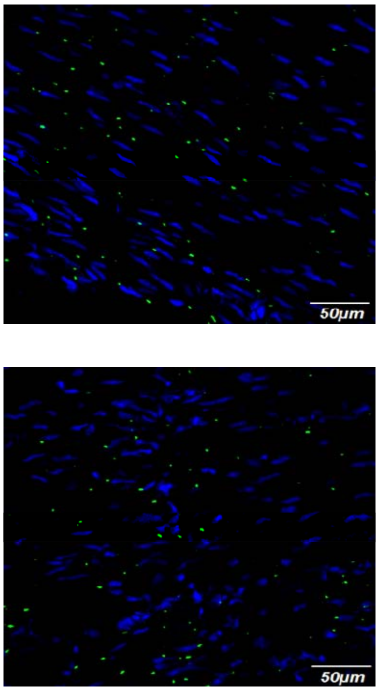

NNMT Stage IV 40X

33 59 114

A

B

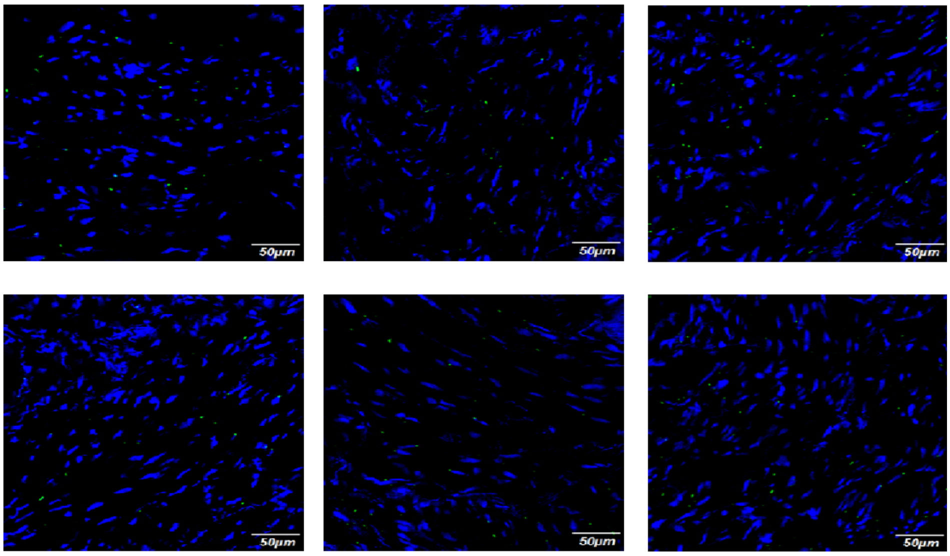

NNMT Stage IV 40X

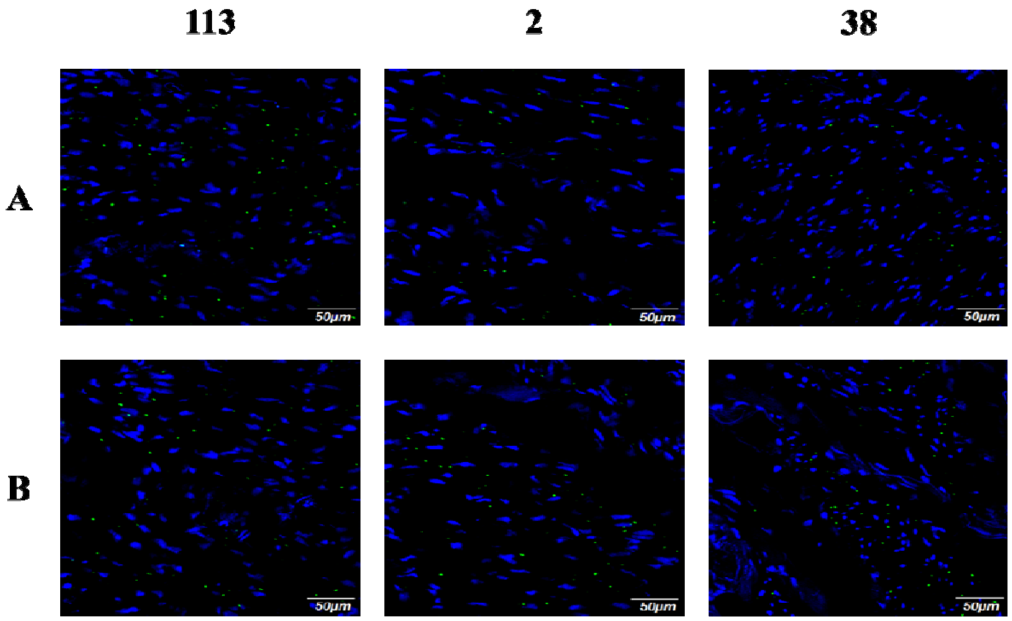

NNMT Stage IV 40X

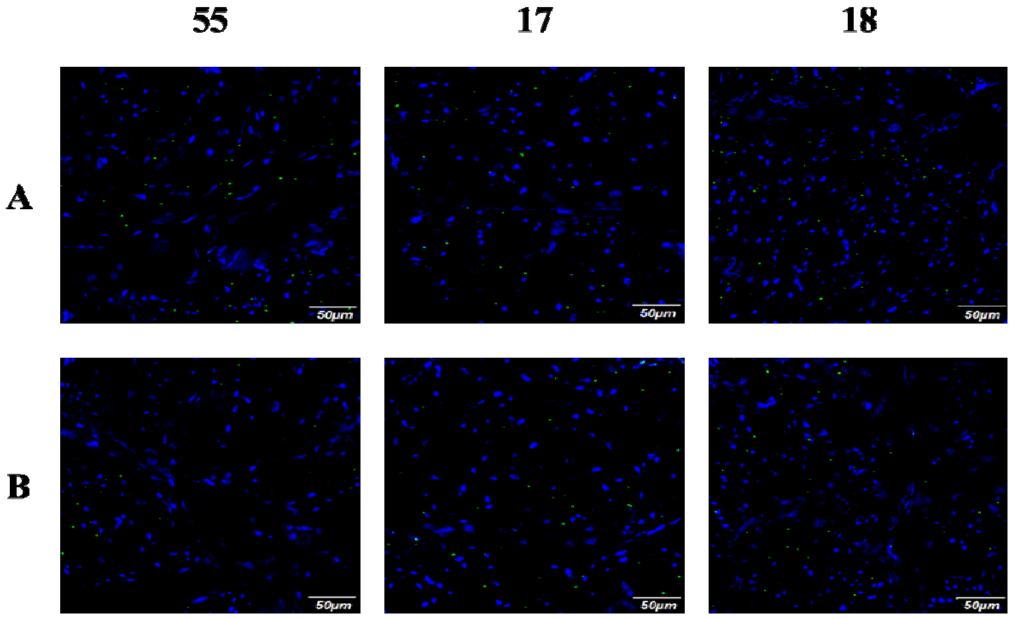

NNMT Stage IV 40X

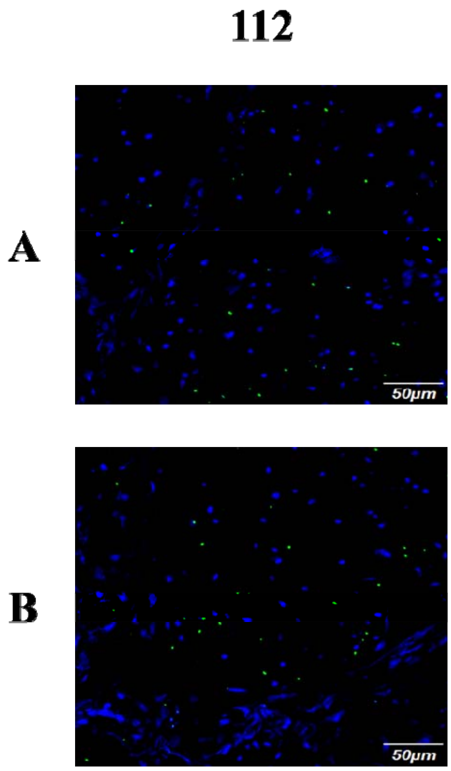

Fibulin5 Stage I 40X

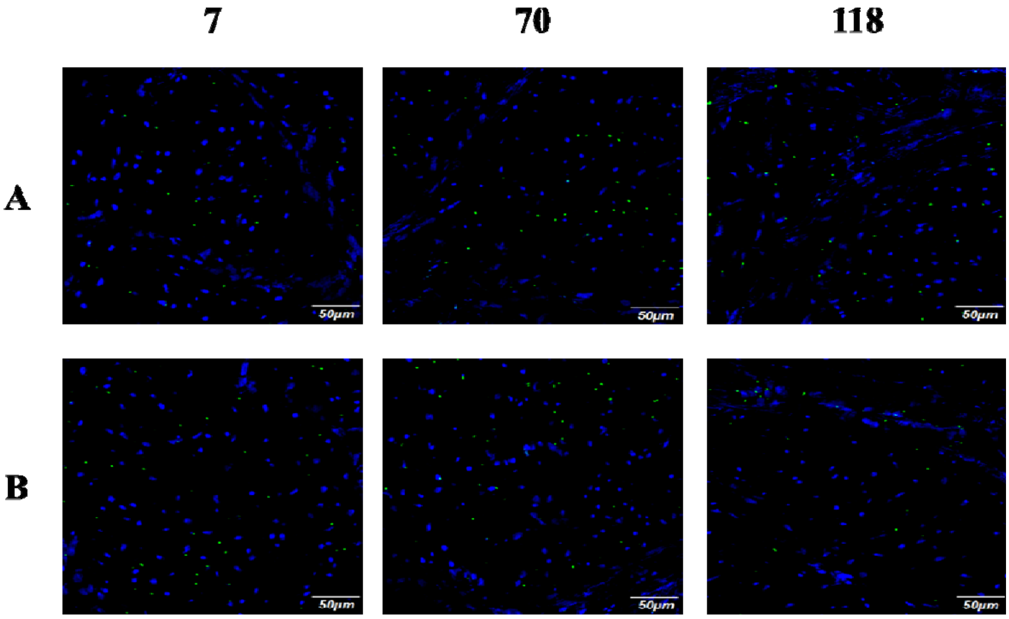

Fibulin5 Stage I 40X

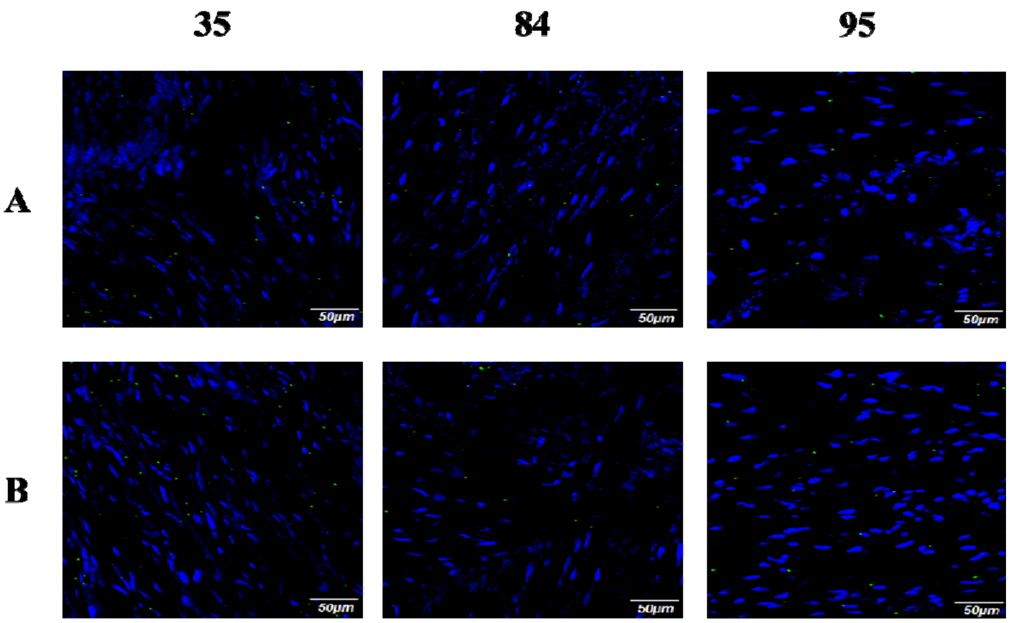

Fibulin5 Stage I 40X

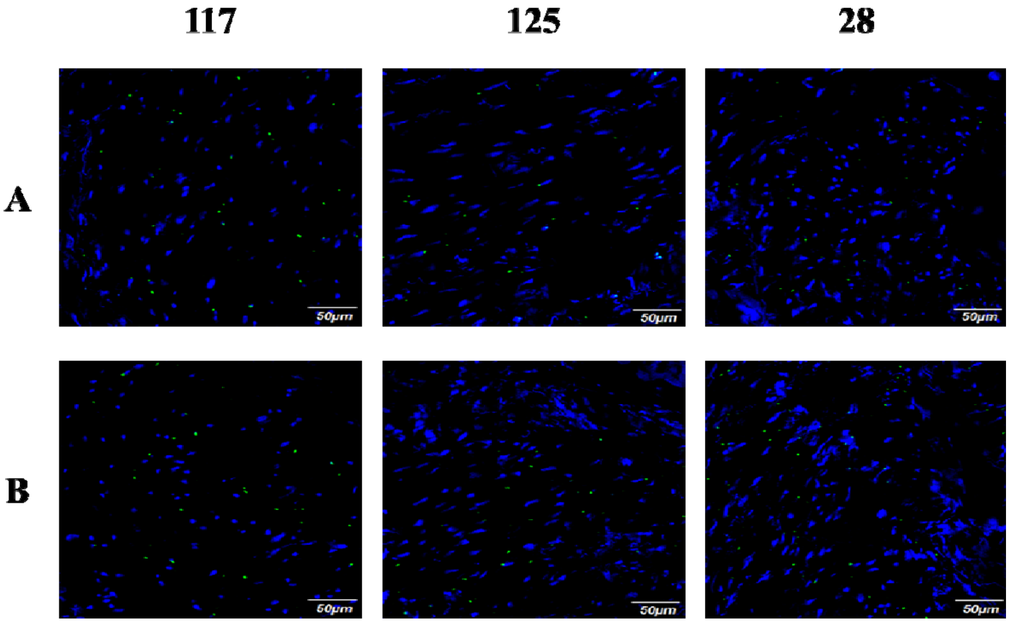

Fibulin5 Stage I 40X

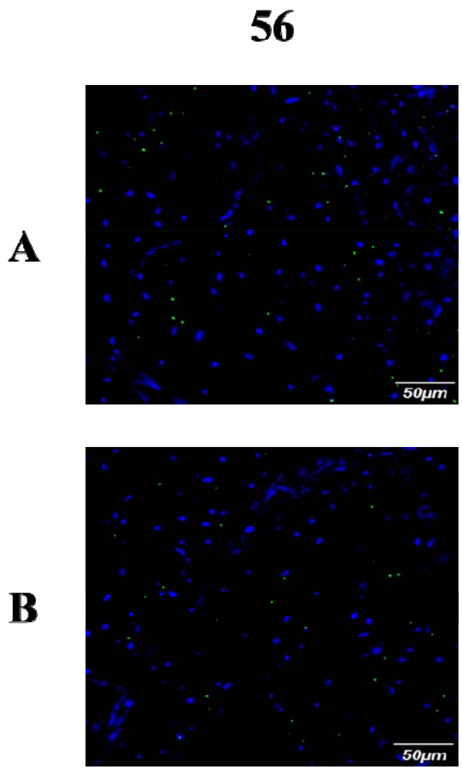

Fibulin5 Stage I 40X

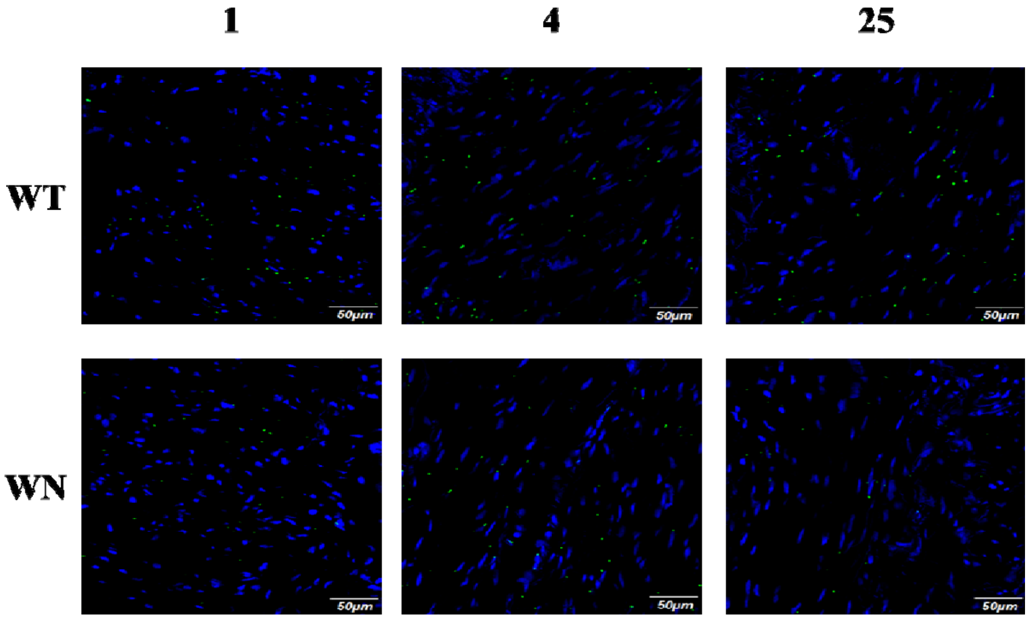

Fibulin5 Stage I 40X

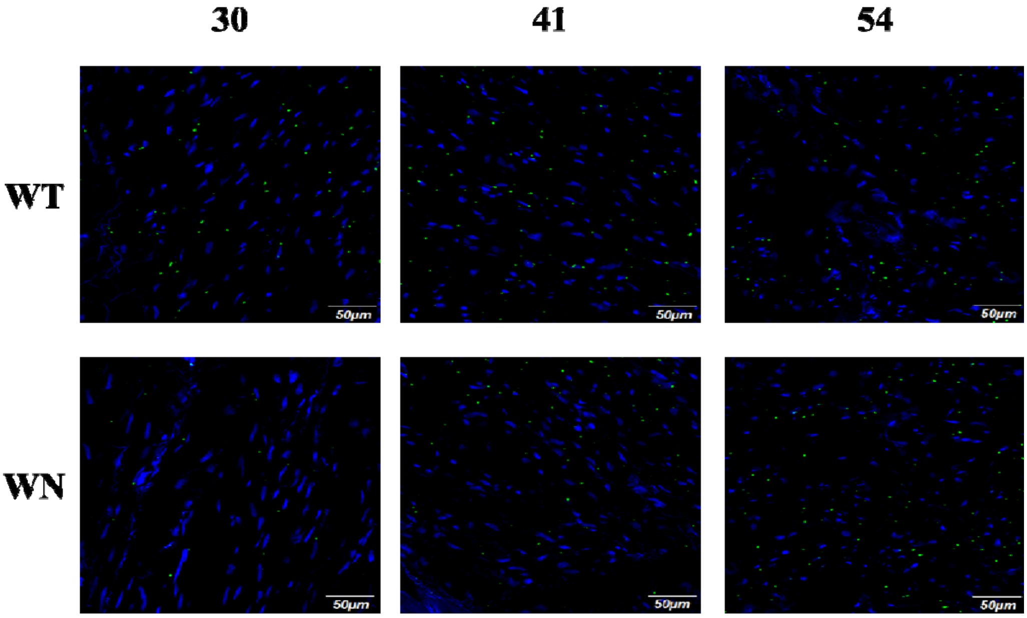

Fibulin5 Stage I 40X

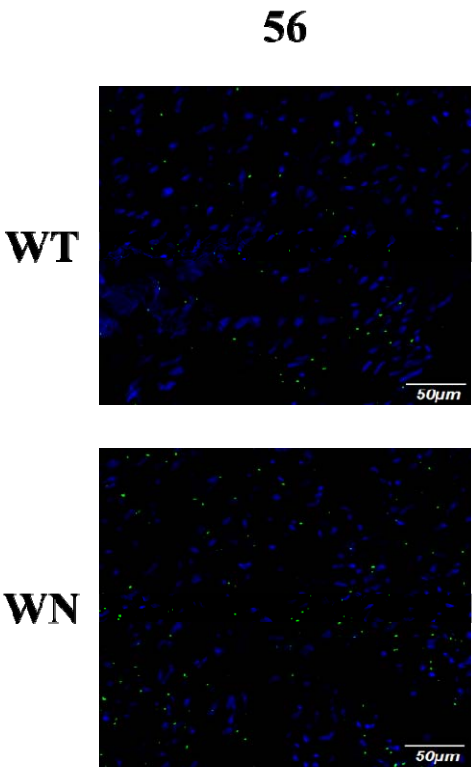

Fibulin5 Stage II 40X

6 37 87

A

B

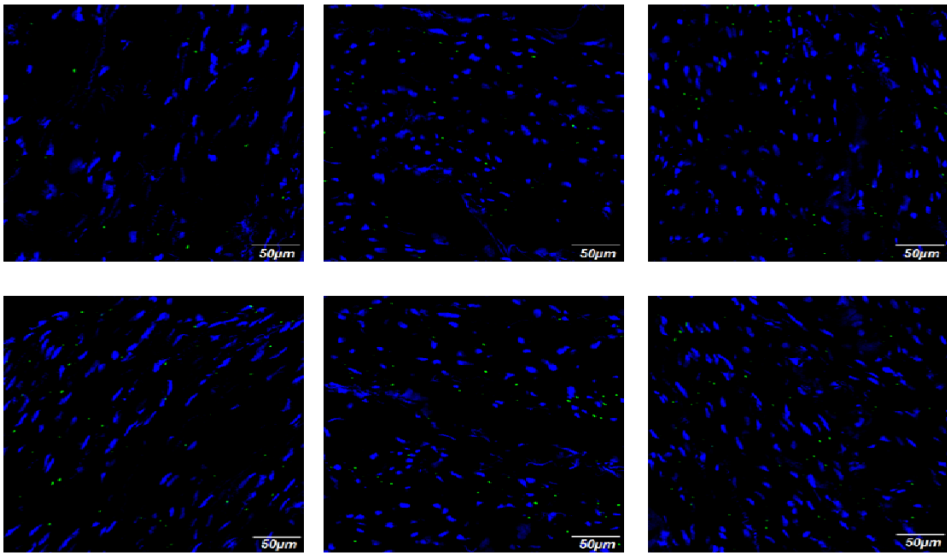

Fibulin5 Stage II 40X

93 103 109

A

B

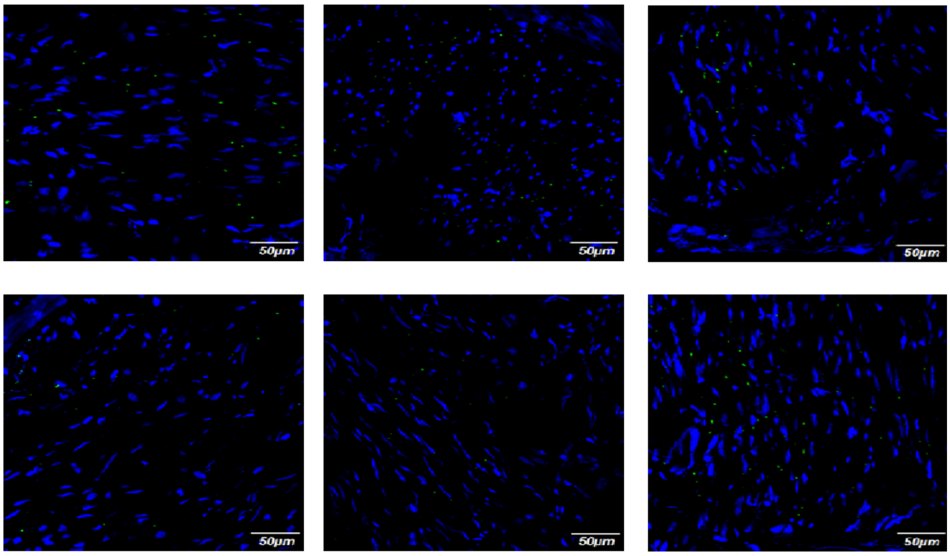

Fibulin5 Stage II 40X

131

A

B

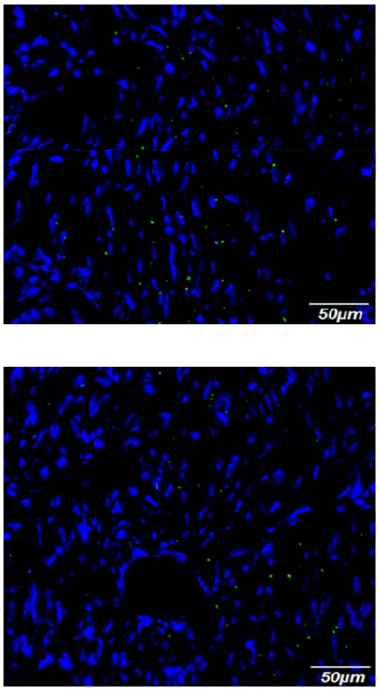

Fibulin5 Stage II 40X

2 **3** **7**

WT

WN

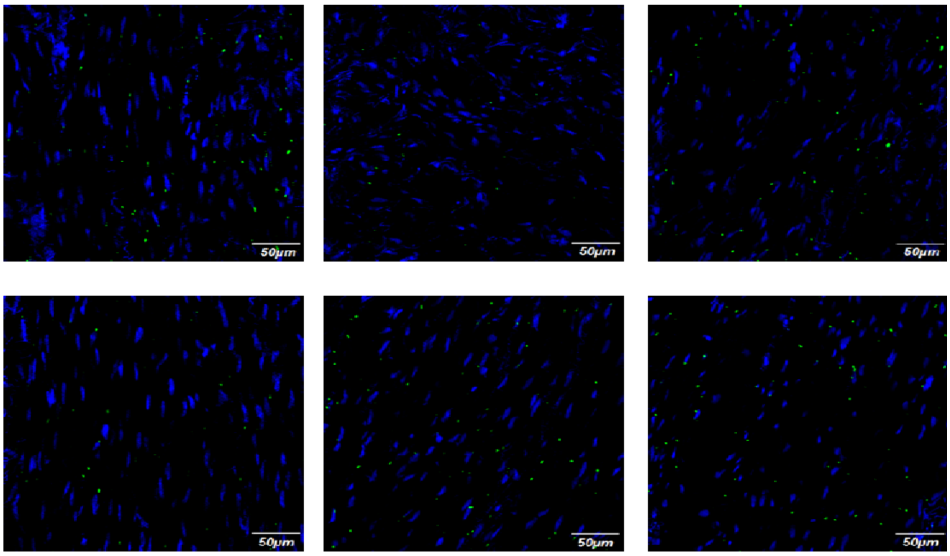

Fibulin5 Stage II 40X

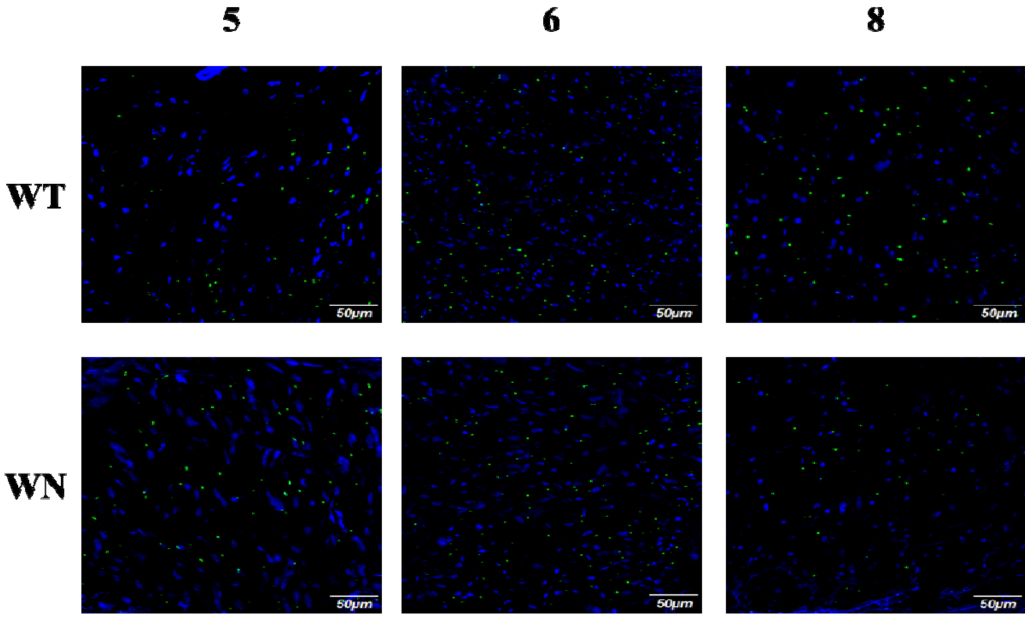

Fibulin5 Stage II 40X

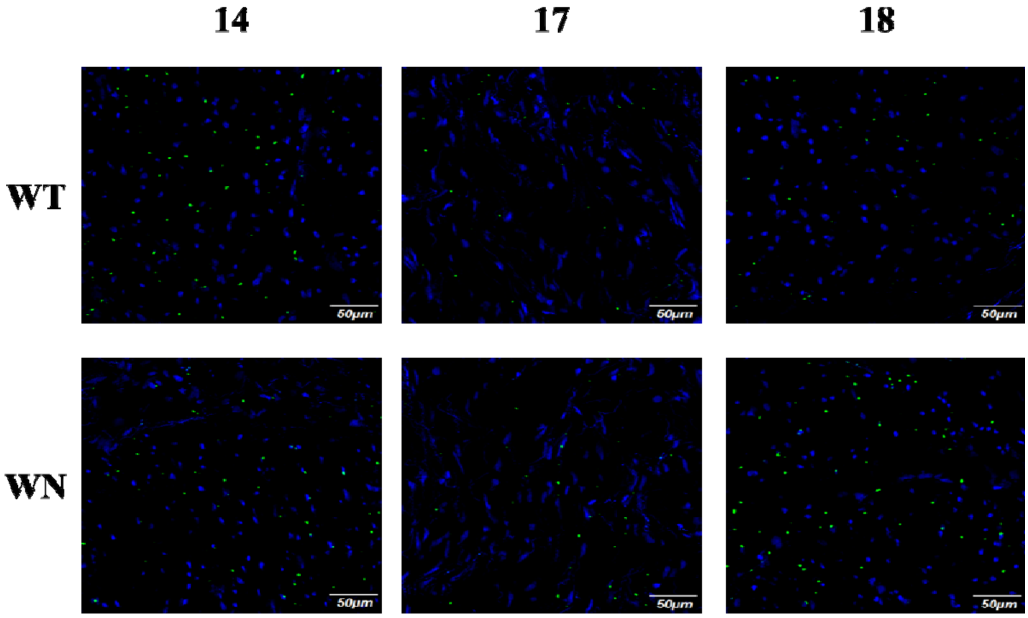

Fibulin5 Stage II 40X

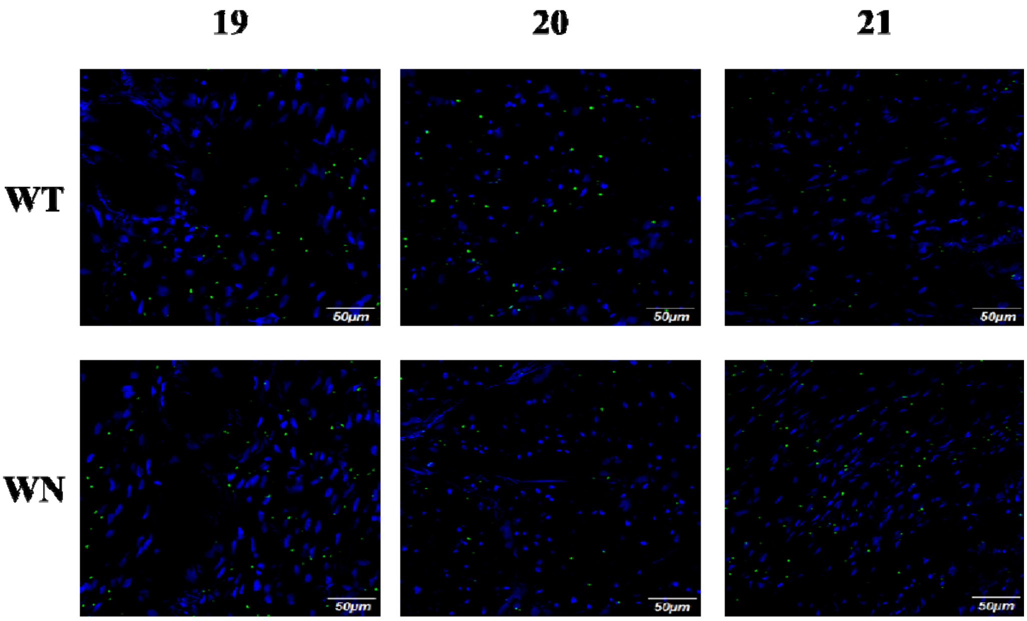

Fibulin5 Stage II 40X

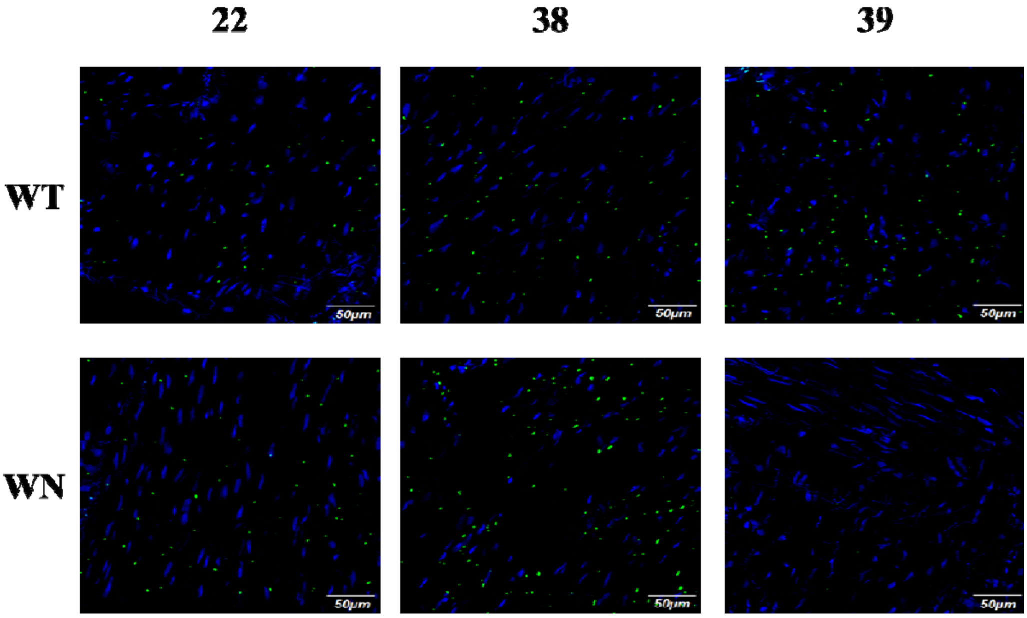

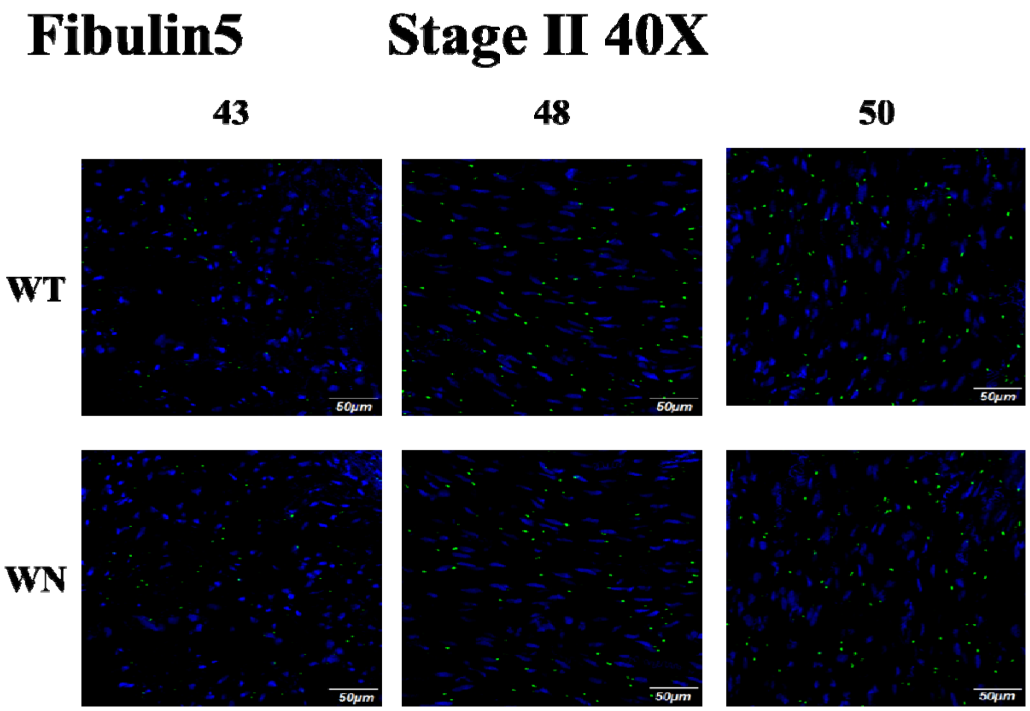
Fibulin5
Stage II 40X
43
48
50
WT
WN
50μm

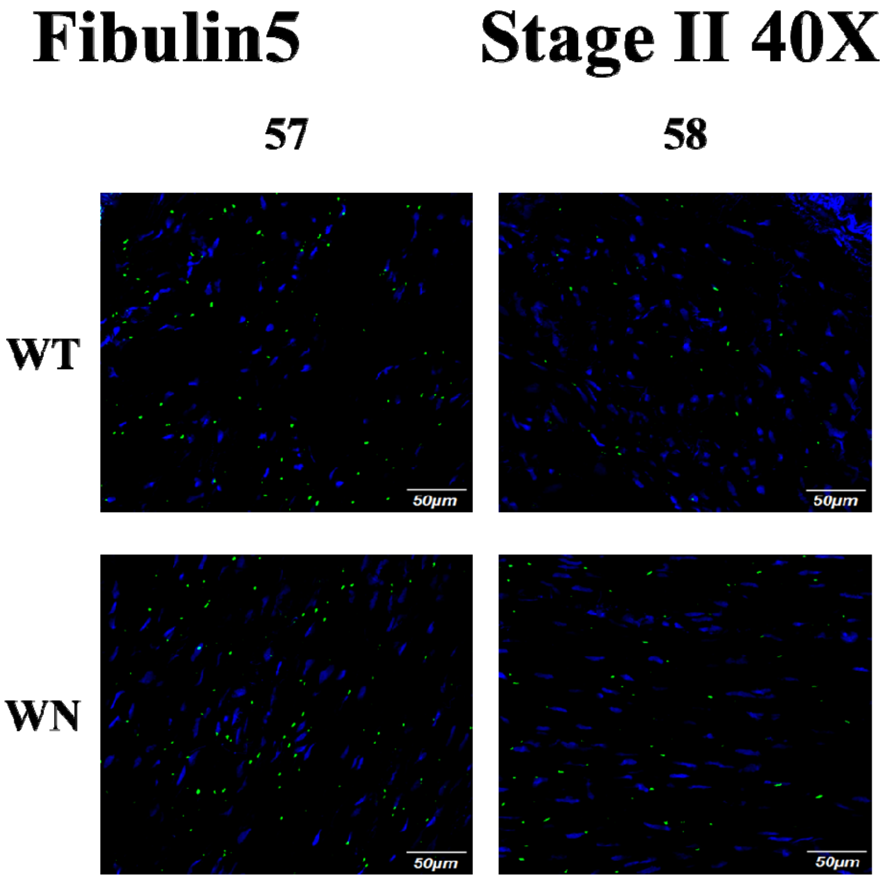
Fibulin5
Stage II 40X
57
58
WT
WN
50μm

Fibulin5 Stage III 40X

11 12 21

A

B

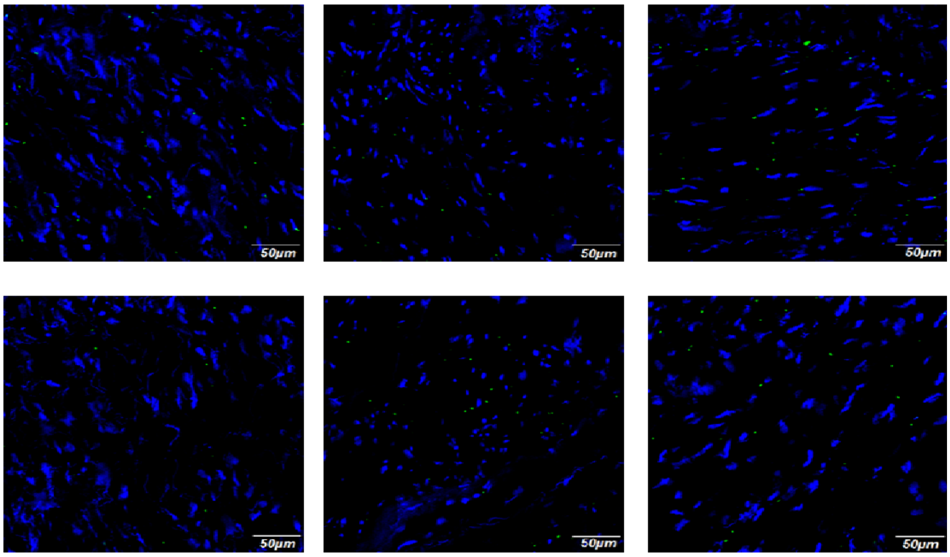

Fibulin5 Stage III 40X

22 43 111

A

B

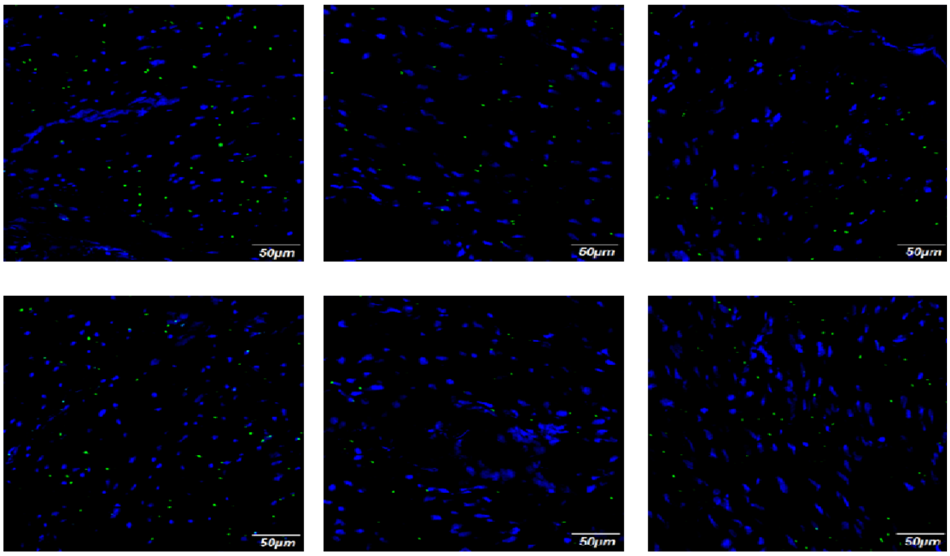

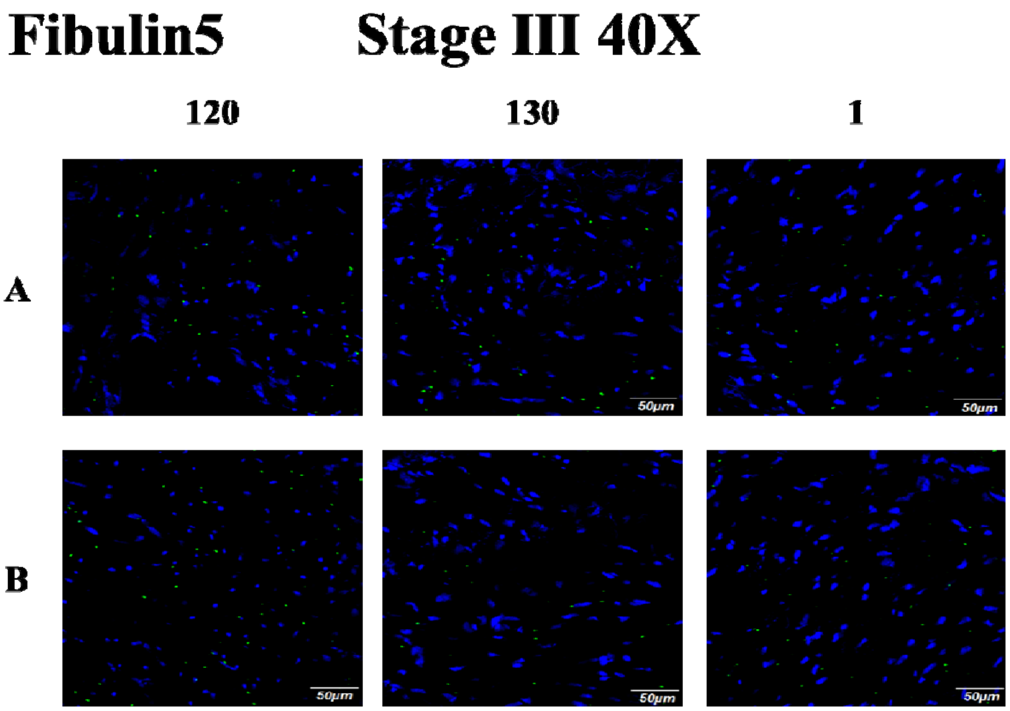
Fibulin5
Stage III 40X
120
130
1
A
B
50μm

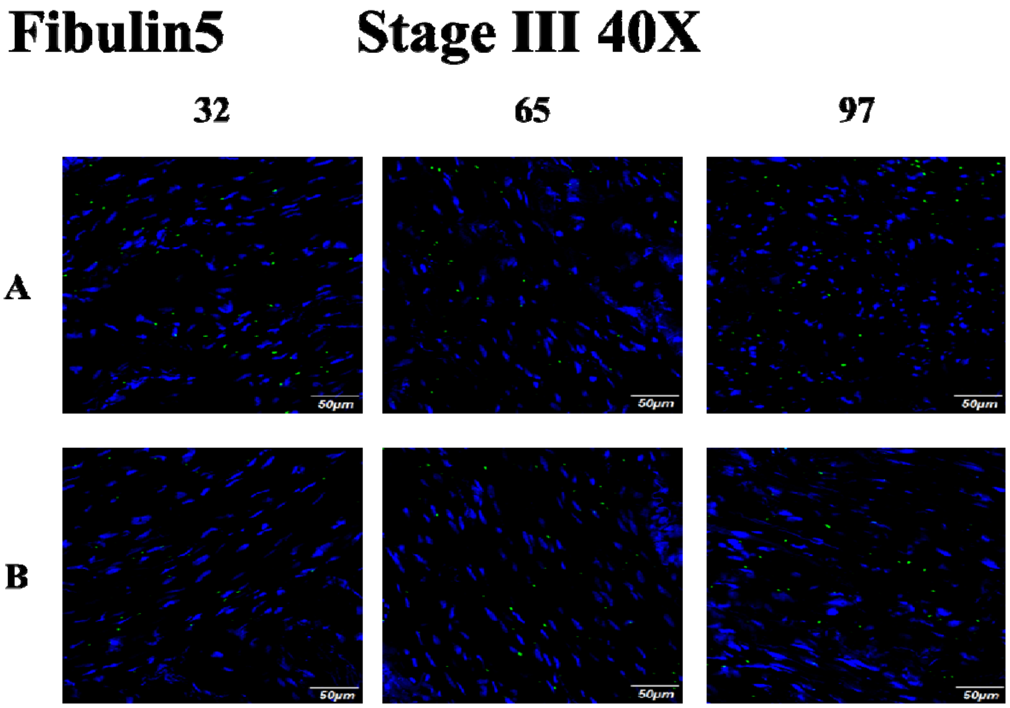
Fibulin5
Stage III 40X
32
65
97
A
B
50μm

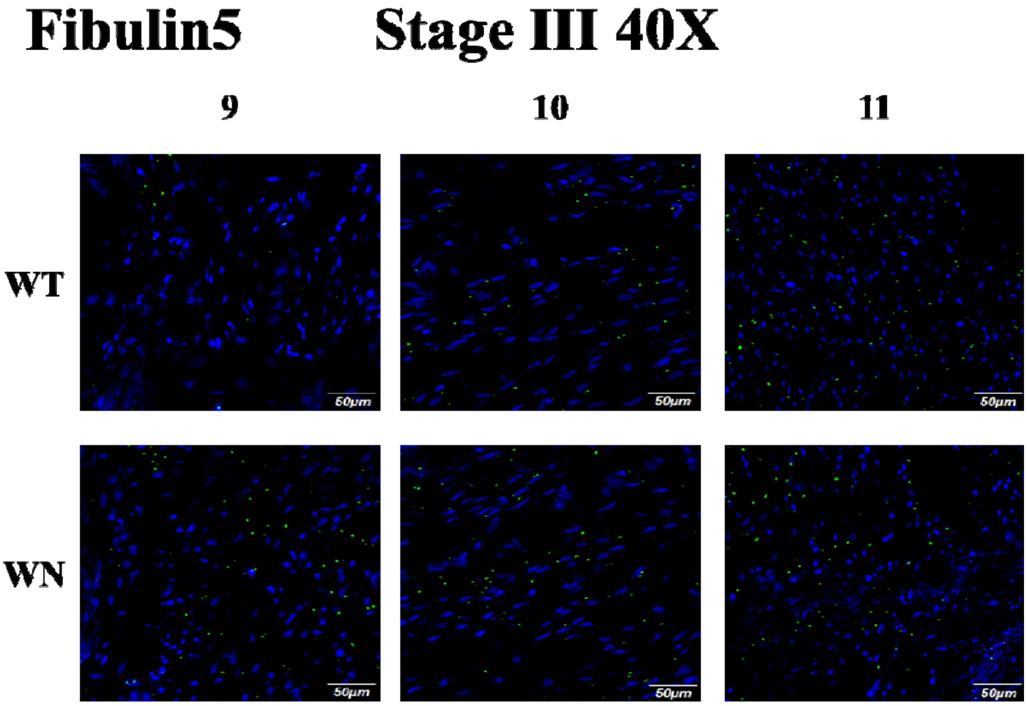
Fibulin5
Stage III 40X
9
10
11
WT
WN
50μm

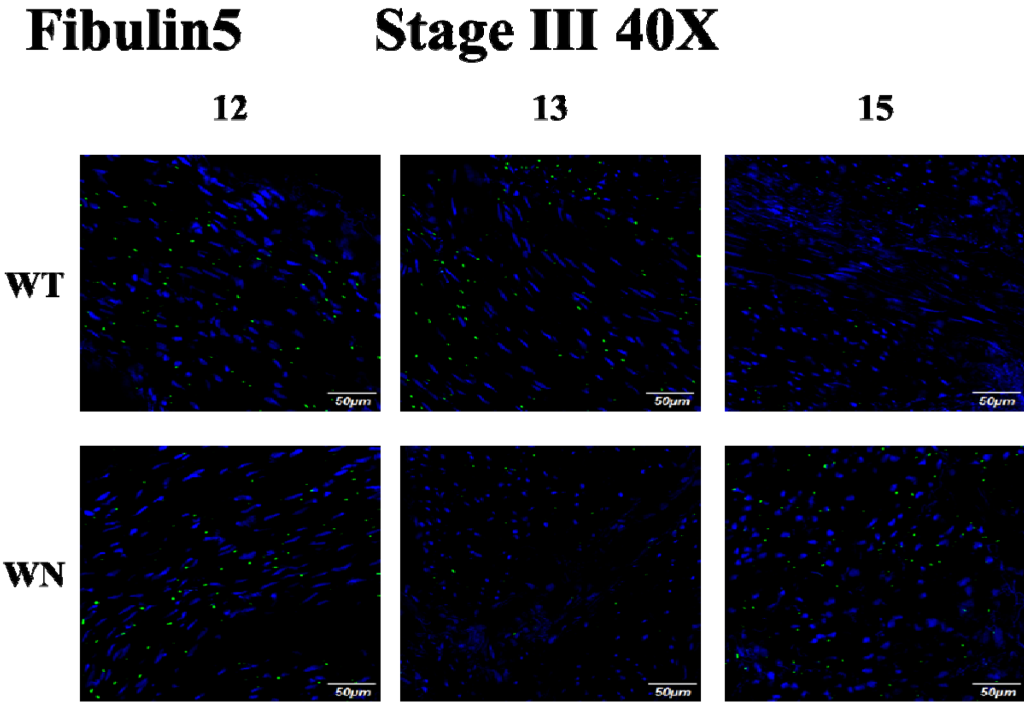
Fibulin5
Stage III 40X
12
13
15
WT
WN
50μm

Fibulin5 Stage III 40X

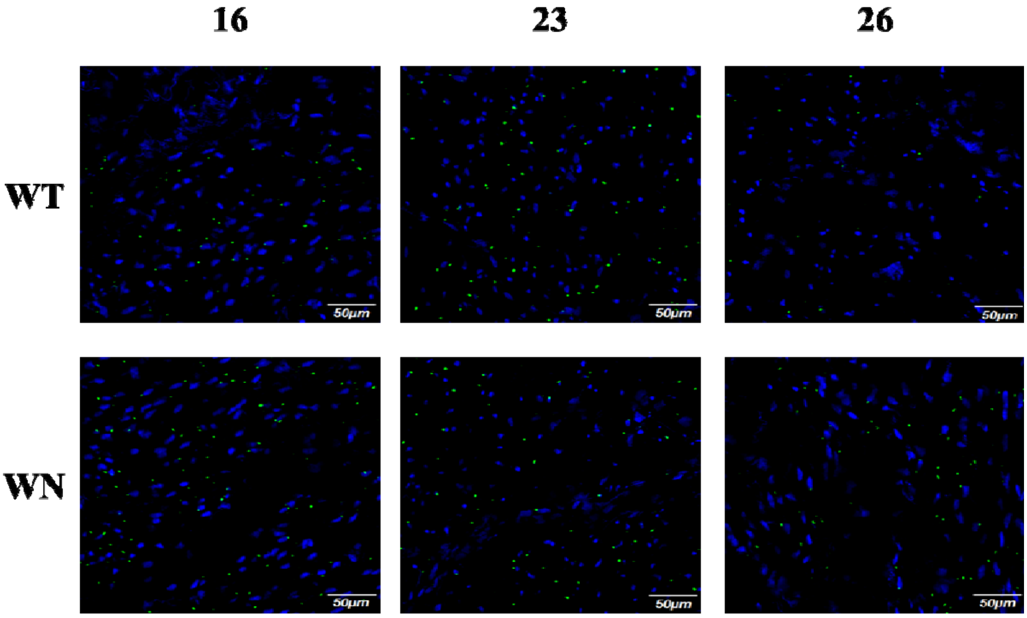

Fibulin5 Stage III 40X

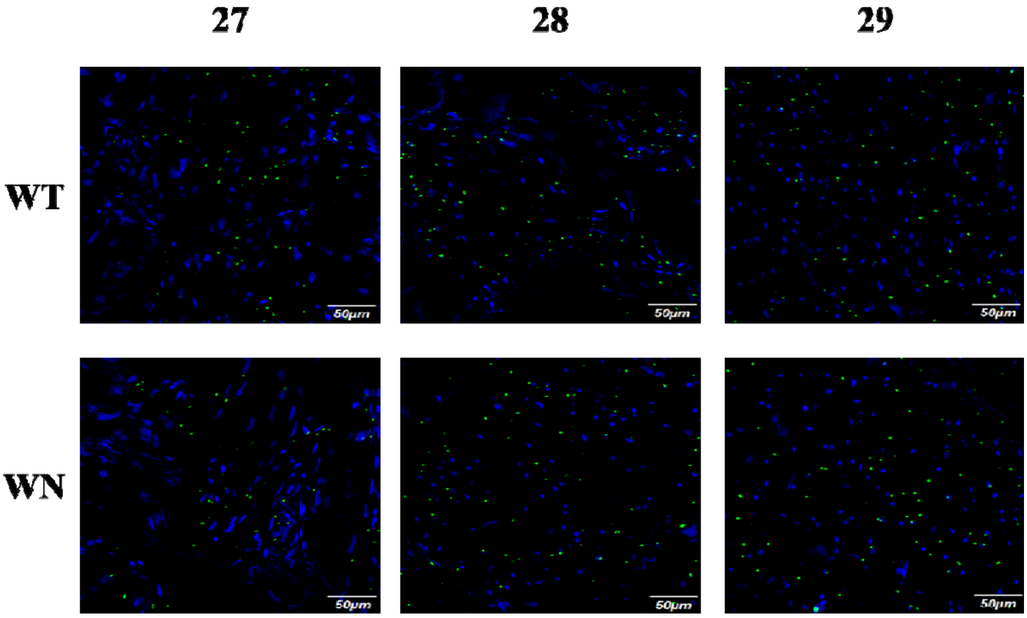

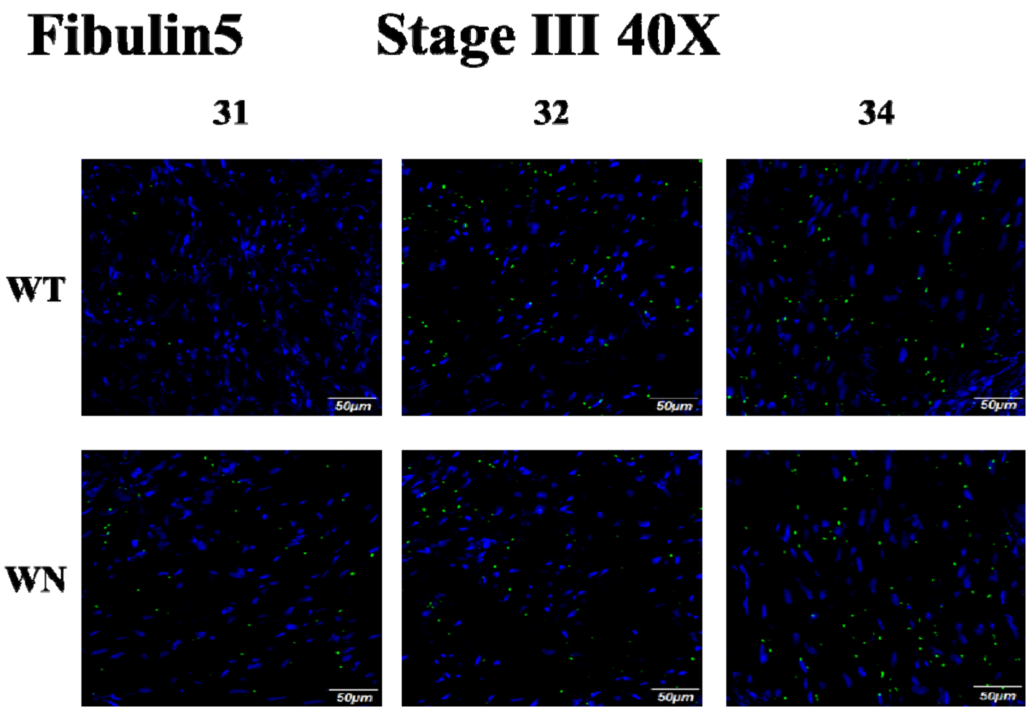
Fibulin5
Stage III 40X
31
32
34
WT
WN
50μm

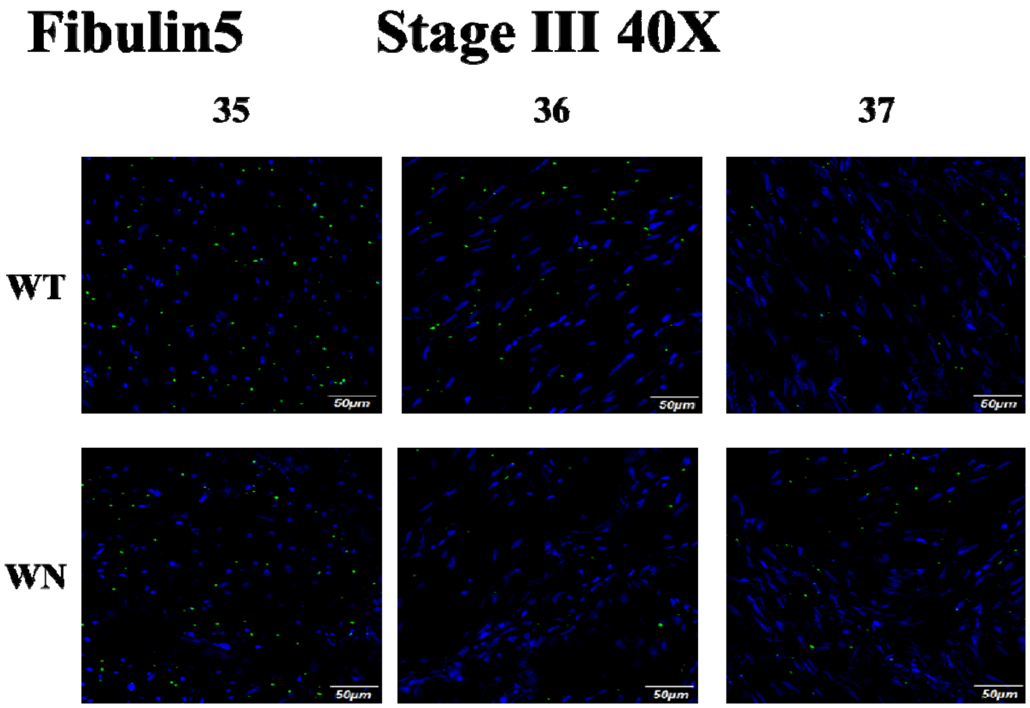
Fibulin5
Stage III 40X
35
36
37
WT
WN
50μm

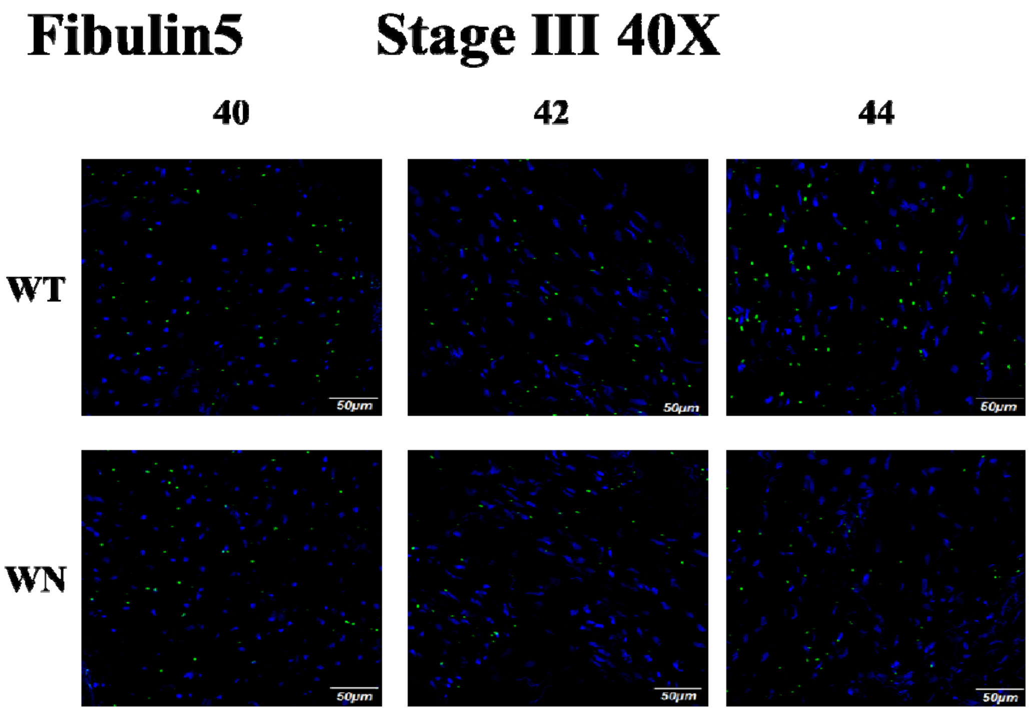
Fibulin5
Stage III 40X
40
42
44
WT
WN
50μm

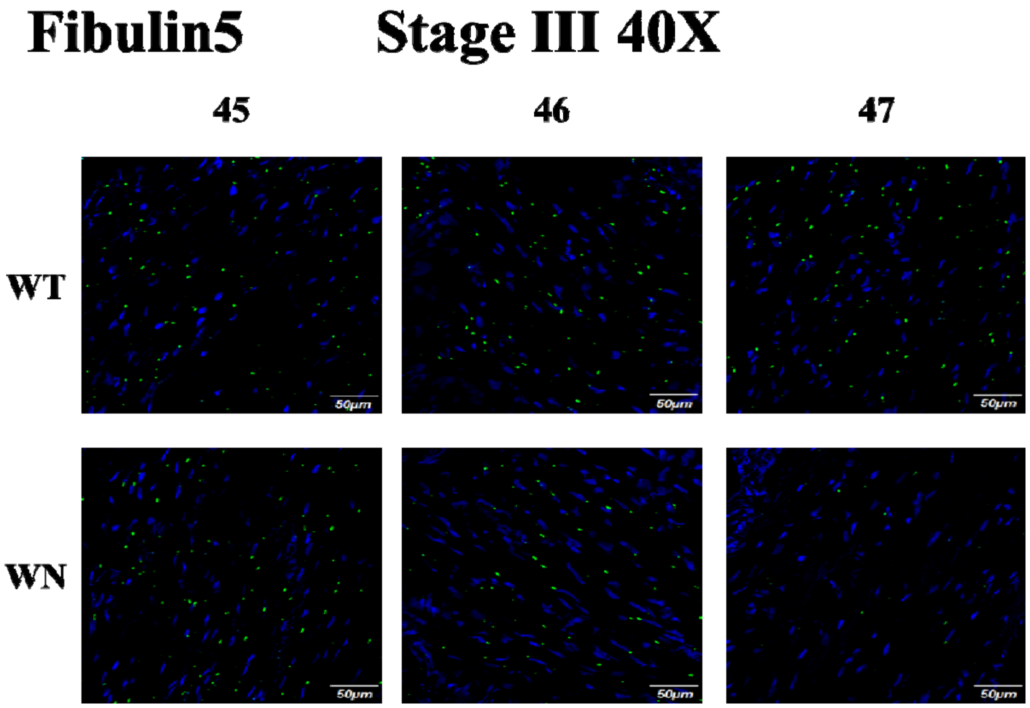
Fibulin5
Stage III 40X
45
46
47
WT
WN
50μm

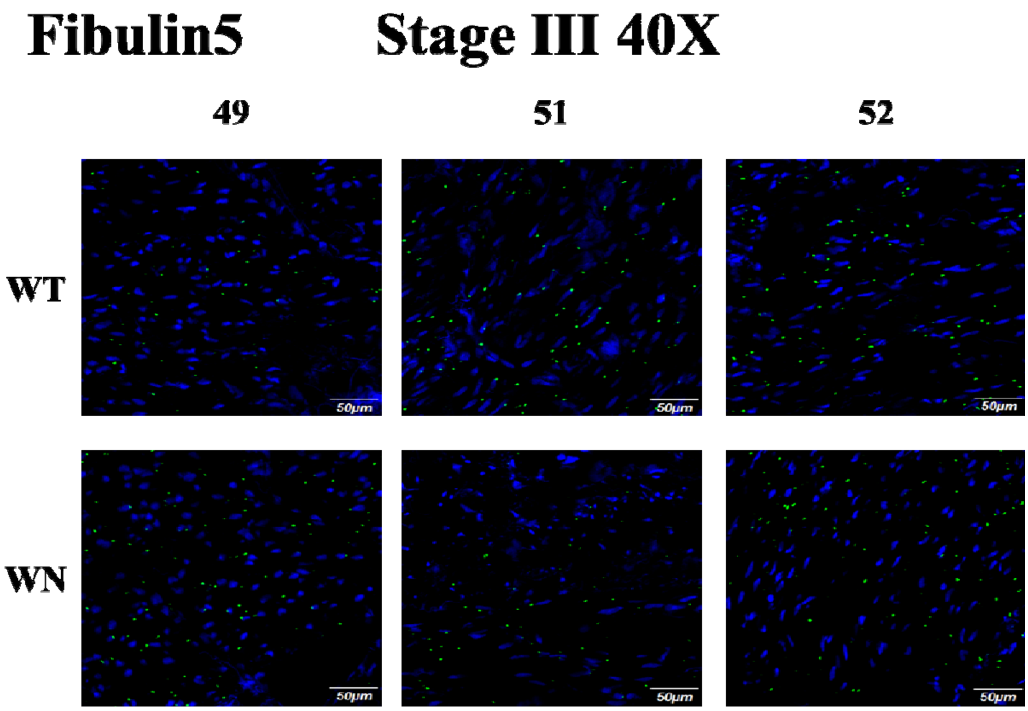
Fibulin5
Stage III 40X
49
51
52
WT
WN
50μm

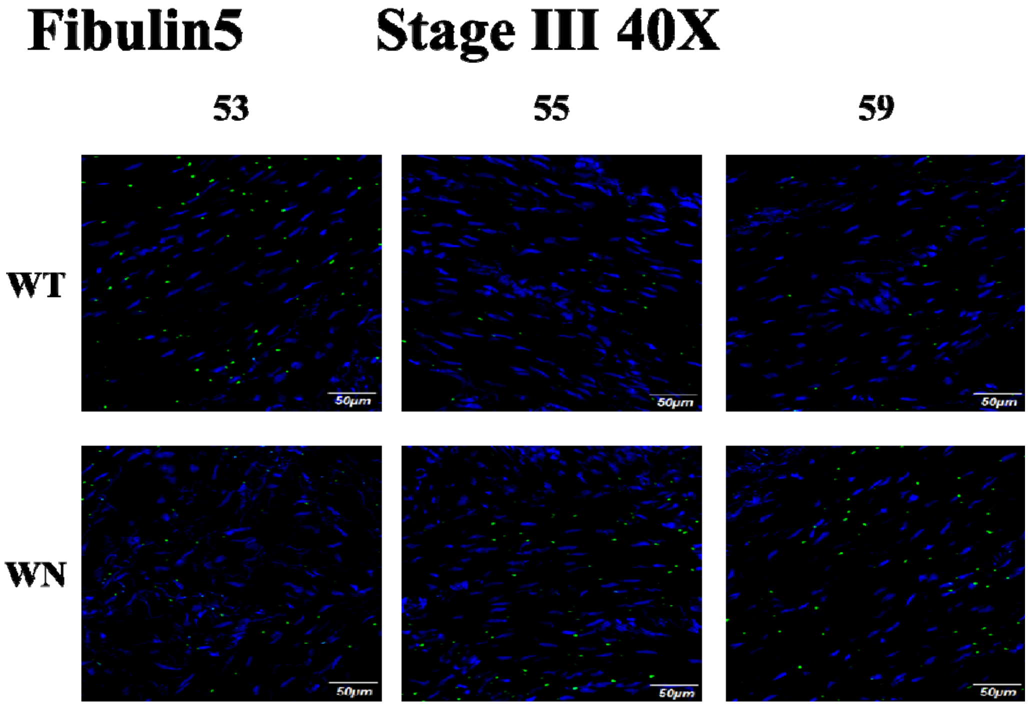
Fibulin5
Stage III 40X
53
55
59
WT
WN
50μm

Fibulin5 Stage III 40X

60

WT

WN

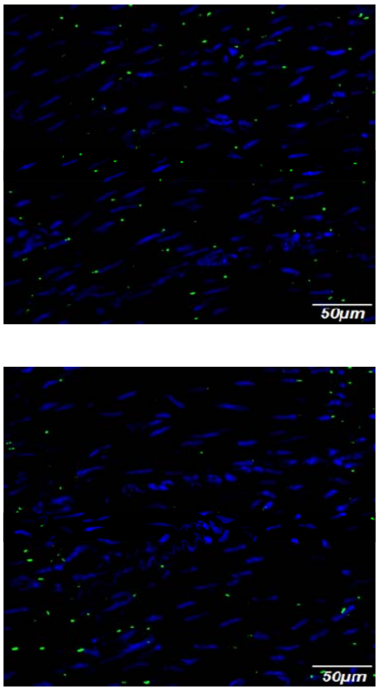

Fibulin5 Stage IV 40X

33 59 114

A

B

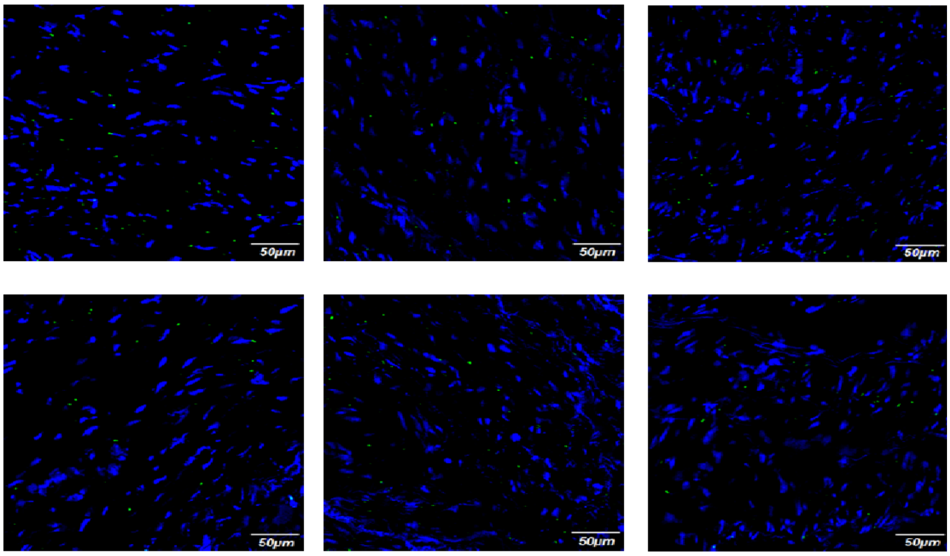

Fibulin5 Stage IV 40X

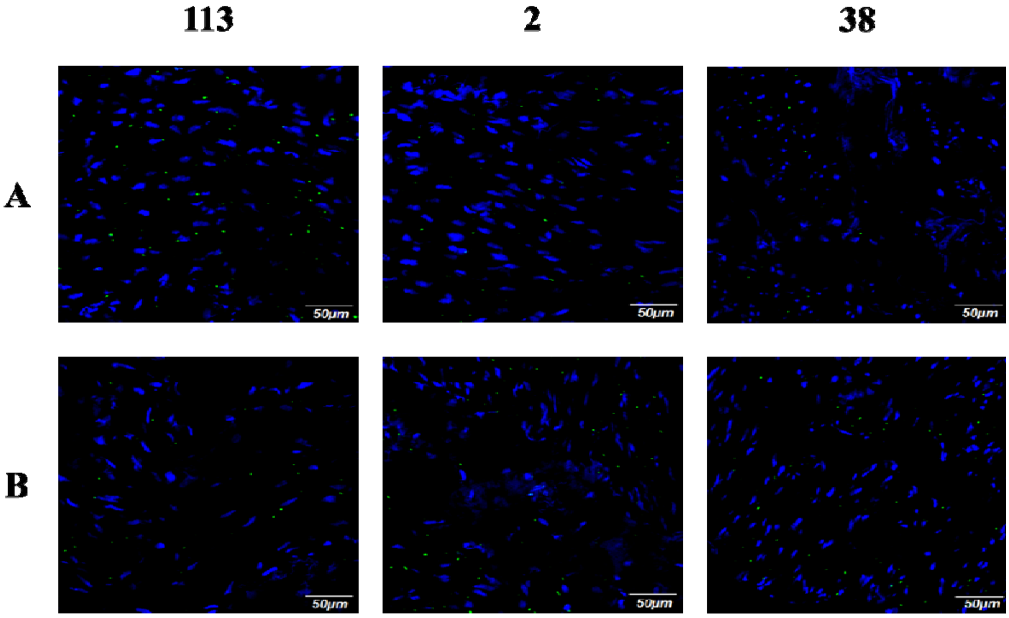

Fibulin5 Stage IV 40X

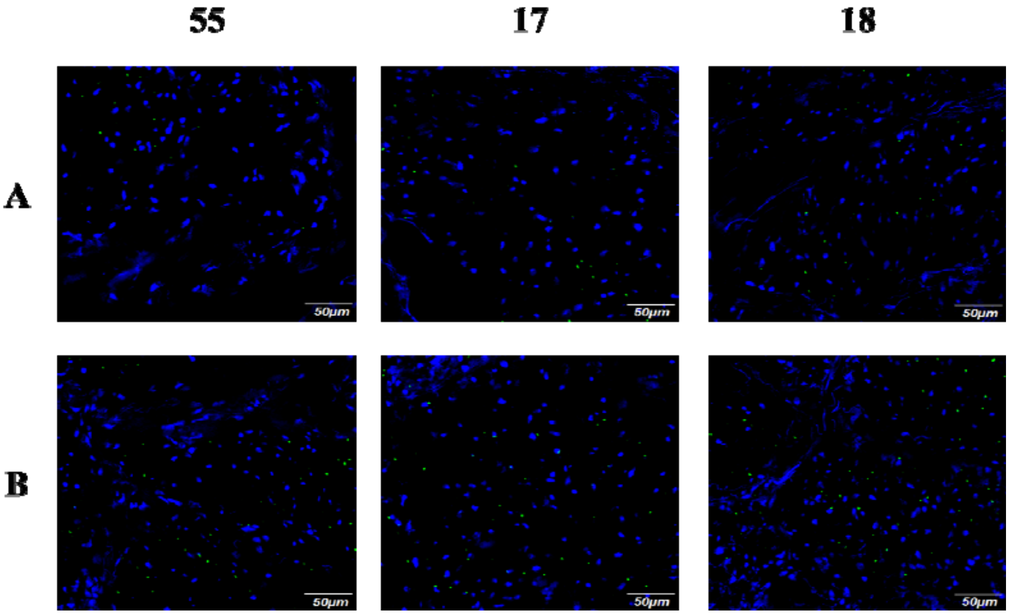

Fibulin5 Stage IV 40X

112

A

B

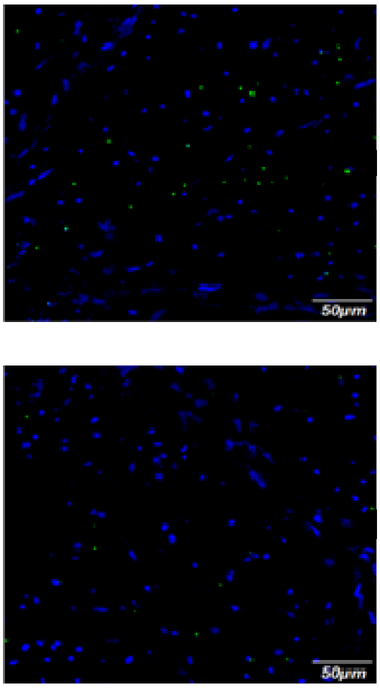

UQCRC1 Stage I 40X

7 70 118

A

B

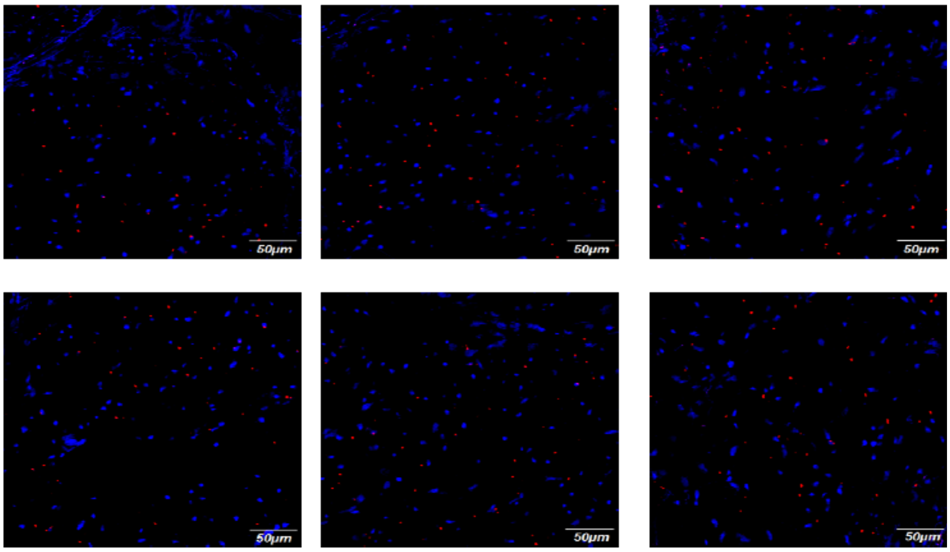

UQCRC1 Stage I 40X

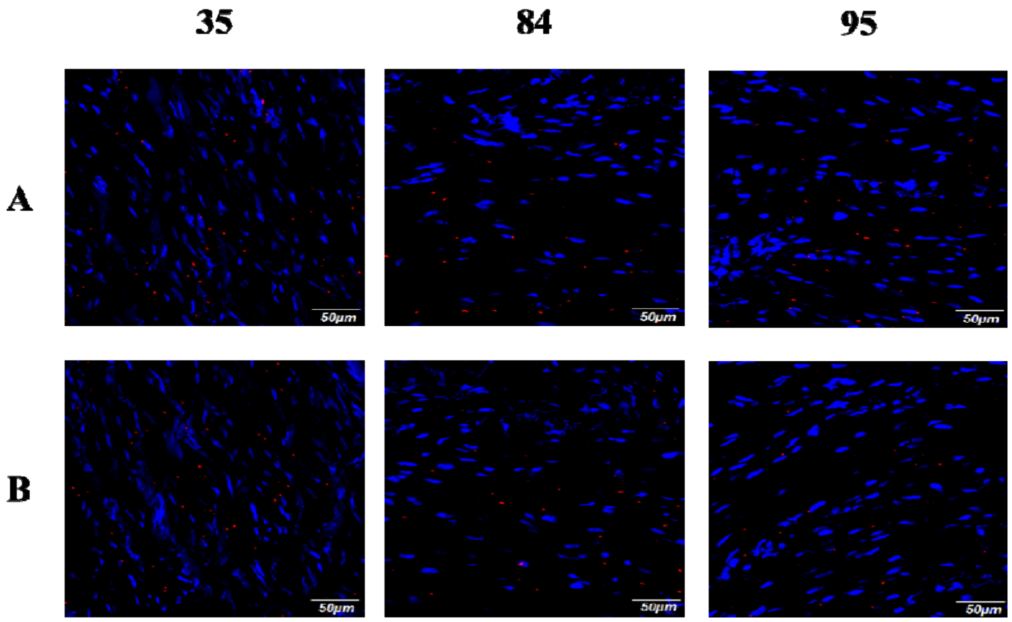

UQCRC1 Stage I 40X

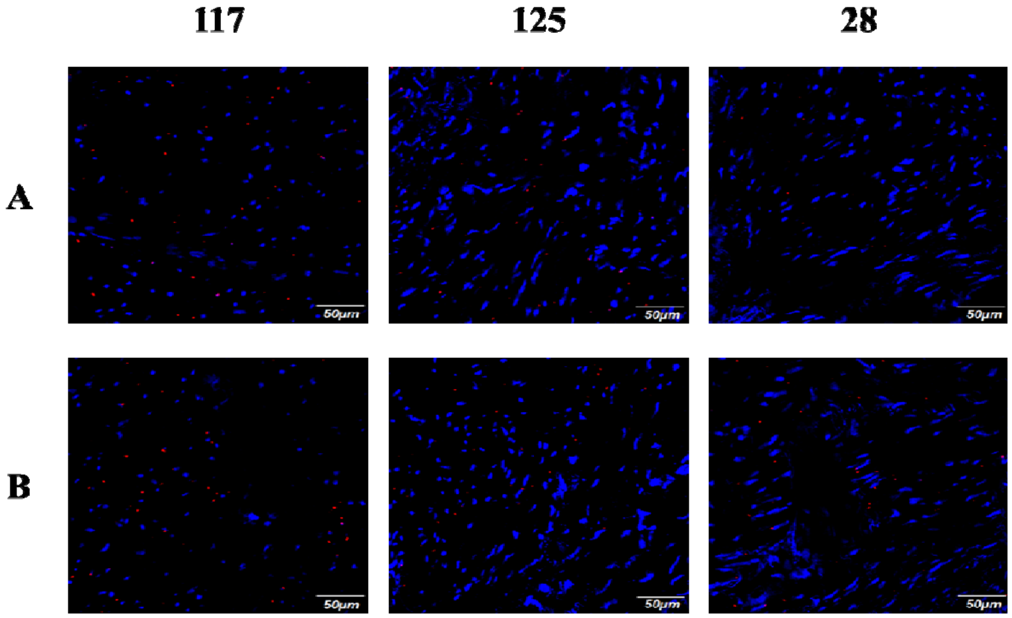

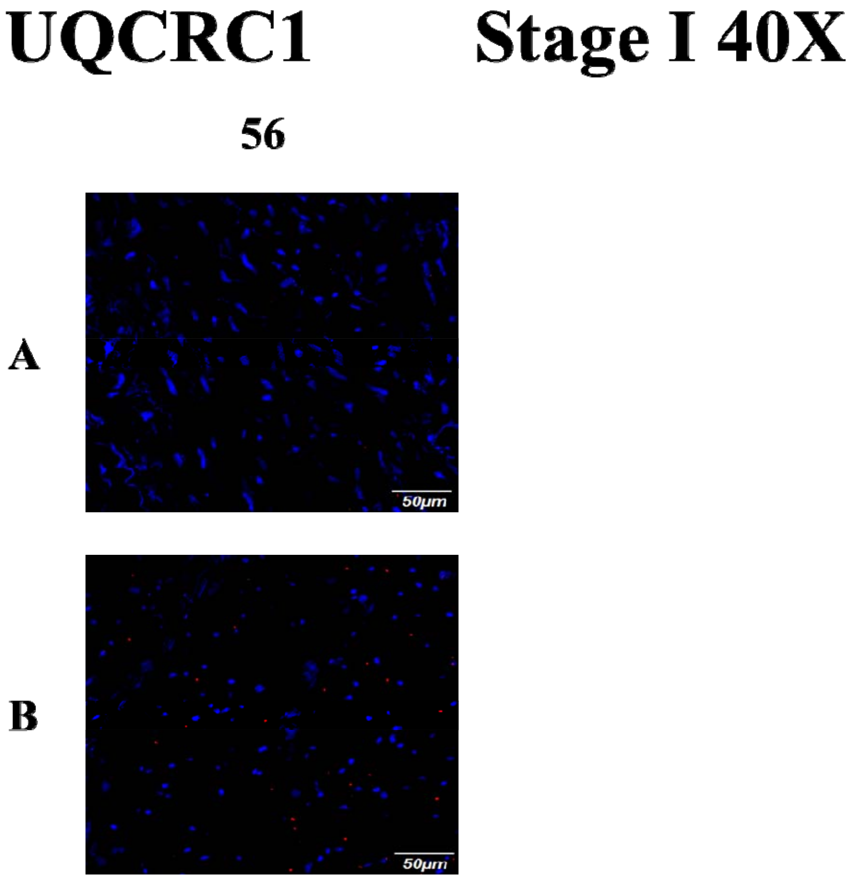
UQCRC1
Stage I 40X
56
A
B
50μm
50μm

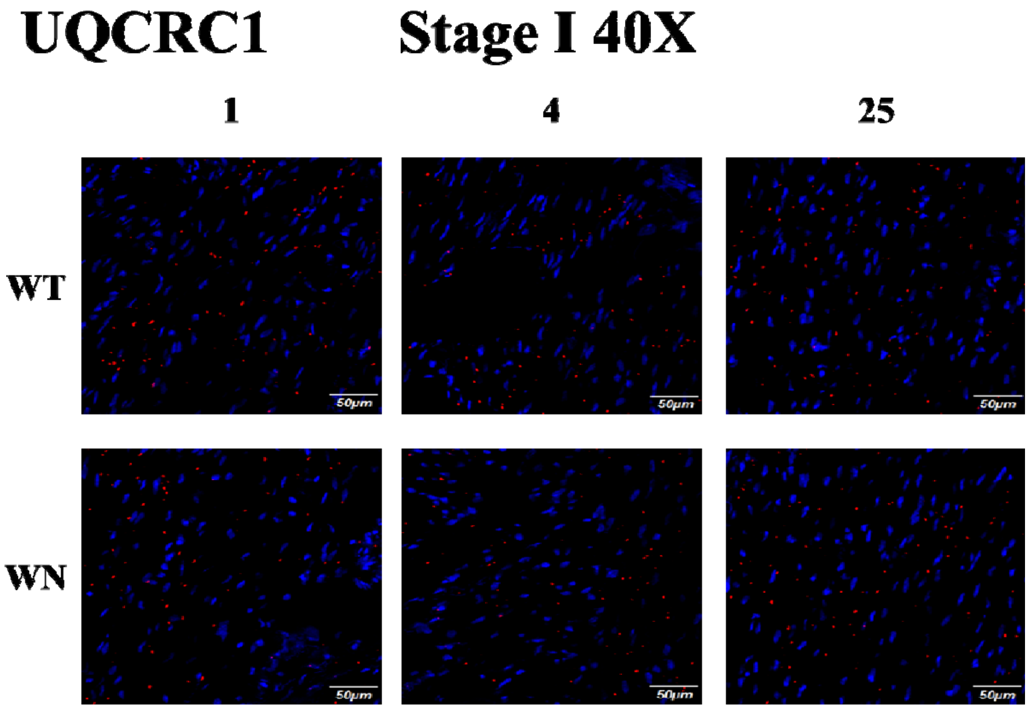
UQCRC1
Stage I 40X
1
4
25
WT
WN
50μm

UQCRC1 Stage I 40X

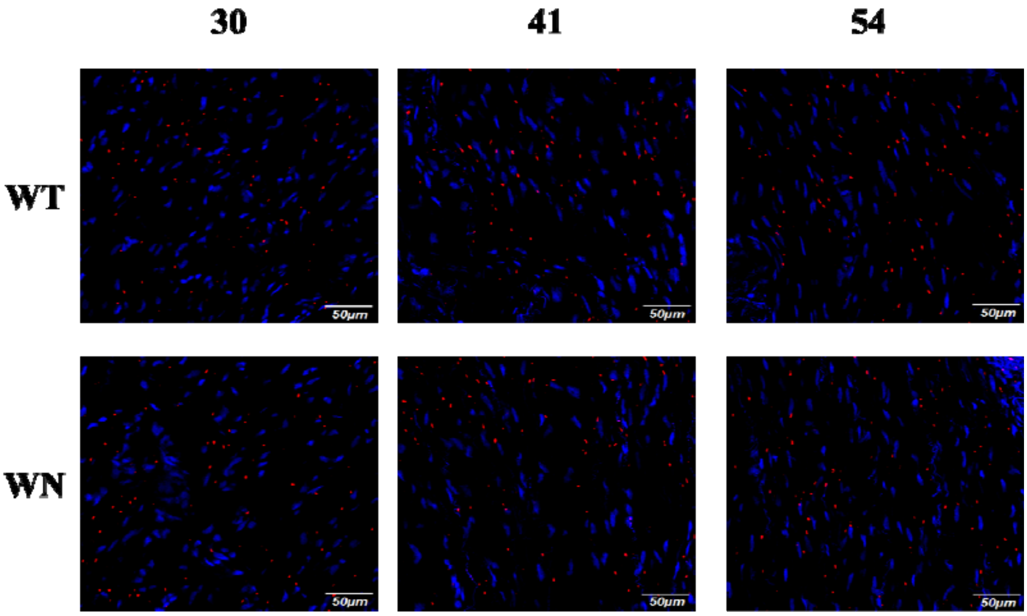

UQCRC1 Stage I 40X

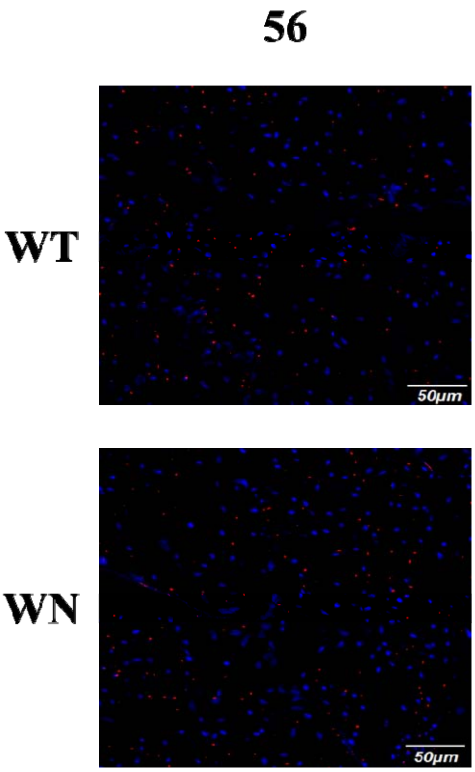

UQCRC1 Stage II 40X

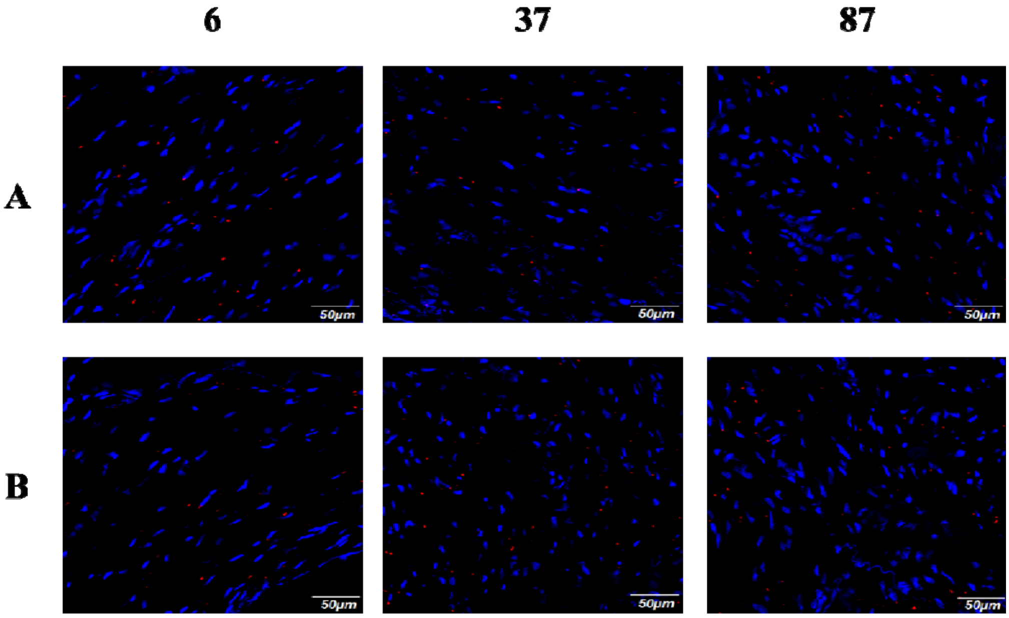

UQCRC1 Stage II 40X

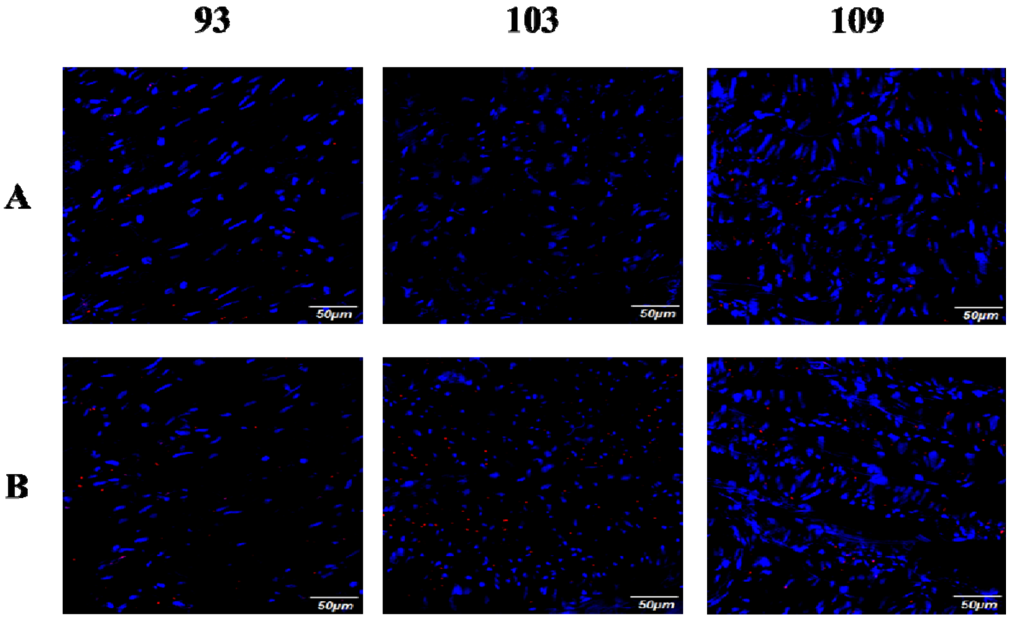

UQCRC1 Stage II 40X

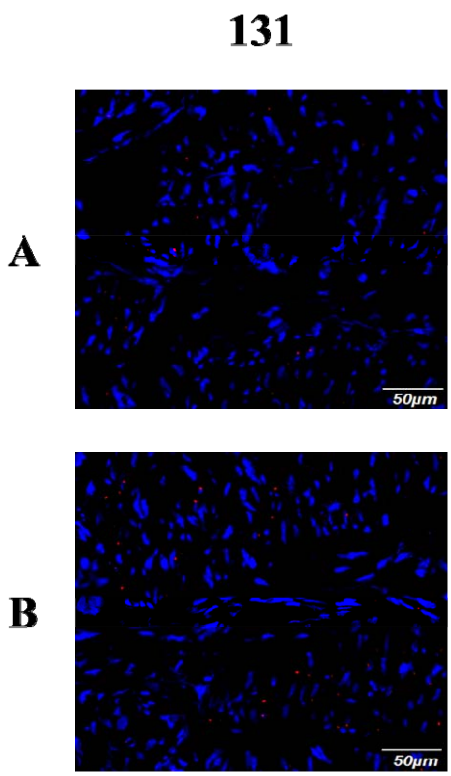

UQCRC1 Stage II 40X

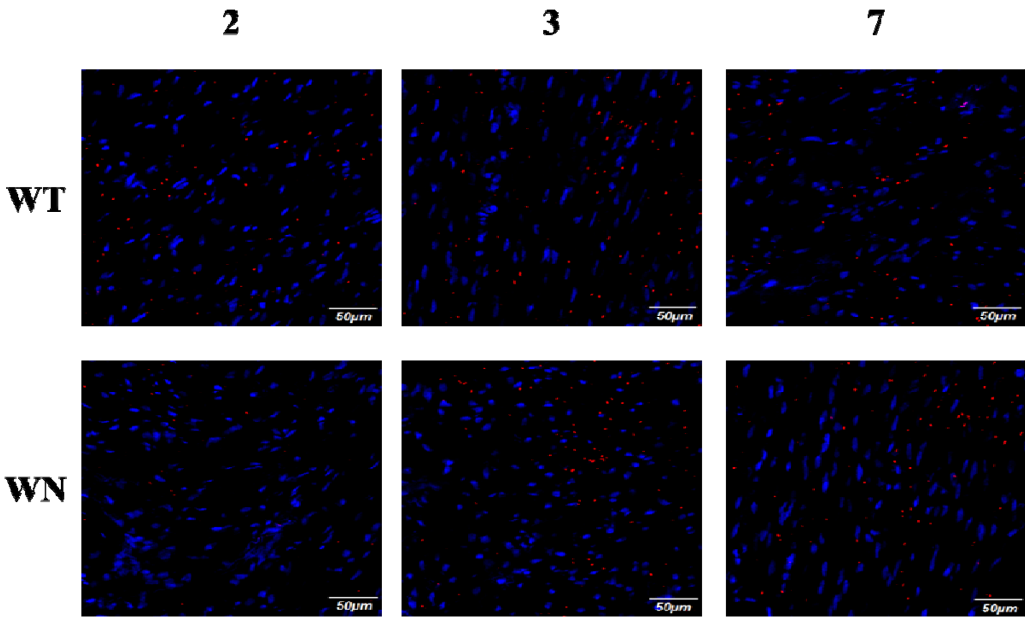

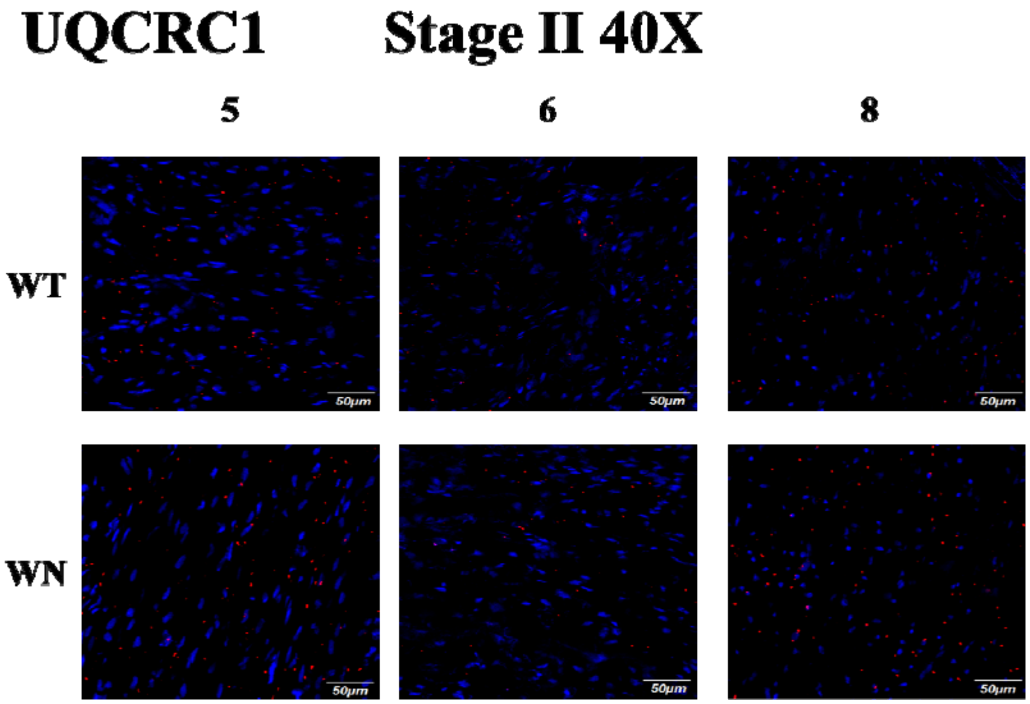
UQCRC1 Stage II 40X
5
6
8
WT
WN
50μm

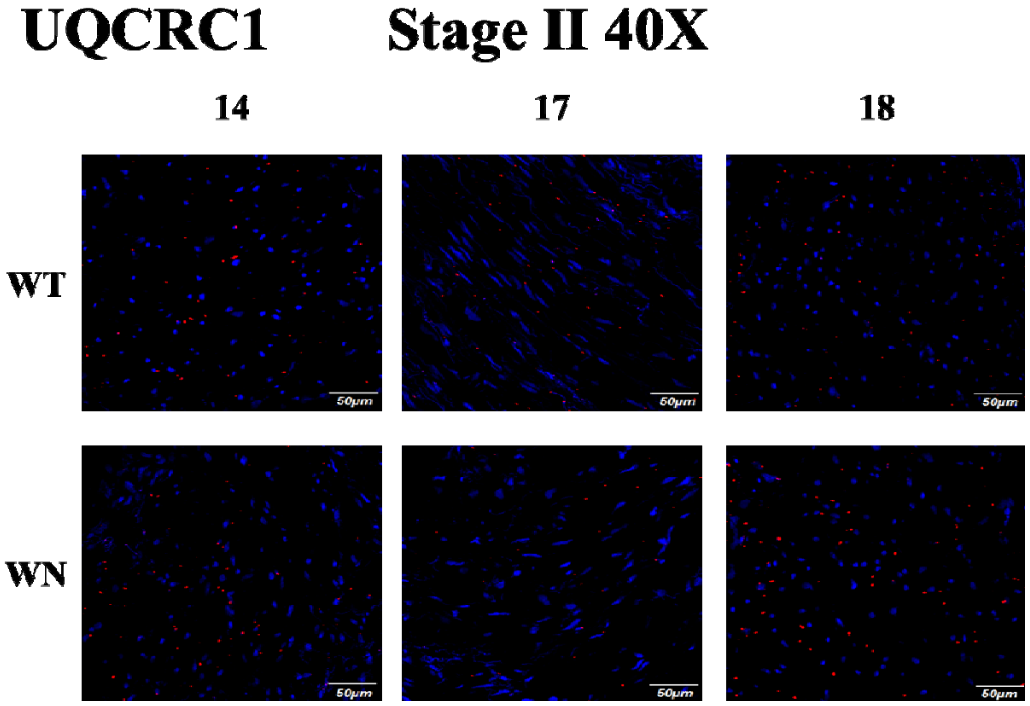
UQCRC1 Stage II 40X
14
17
18
WT
WN
50μm

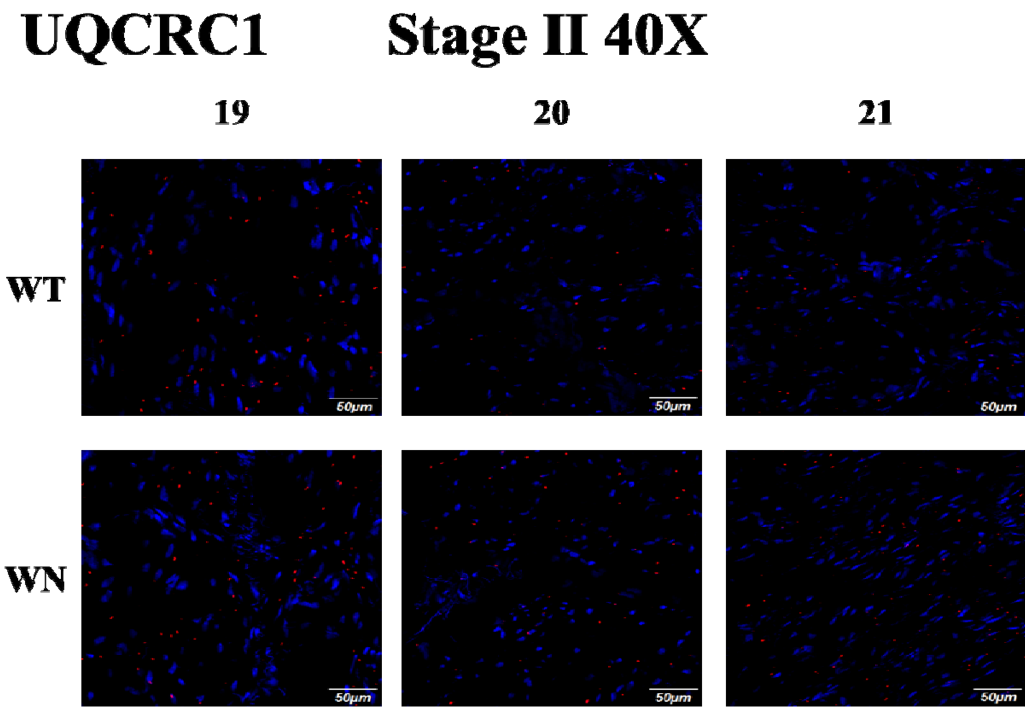
UQCRC1
Stage II 40X
19
20
21
WT
WN
50μm

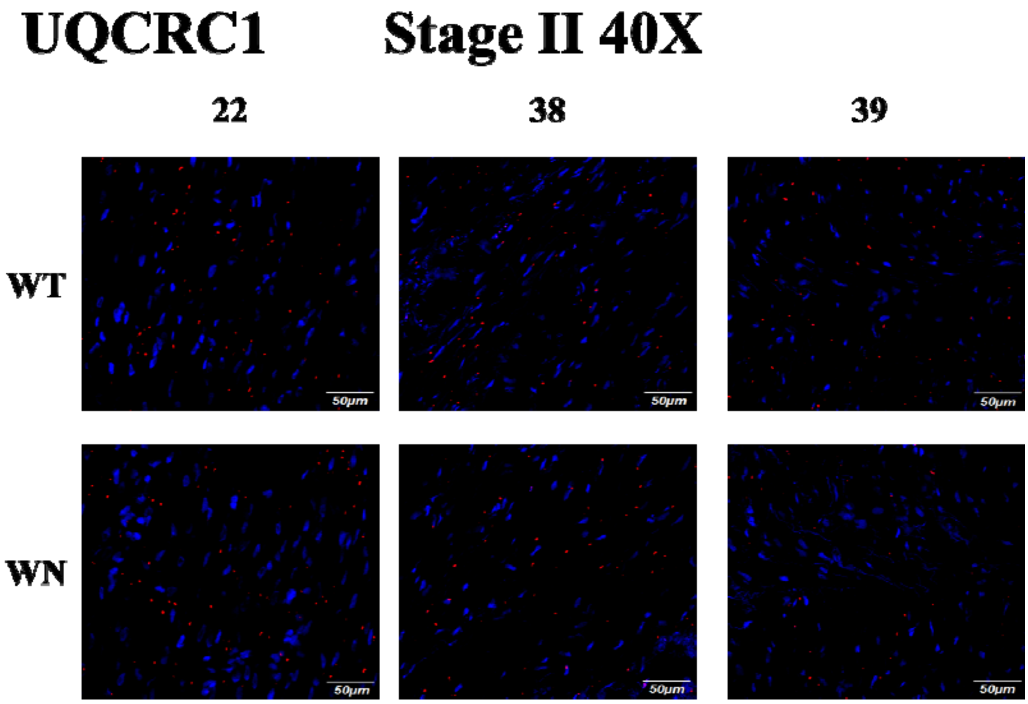
UQCRC1
Stage II 40X
22
38
39
WT
WN
50μm

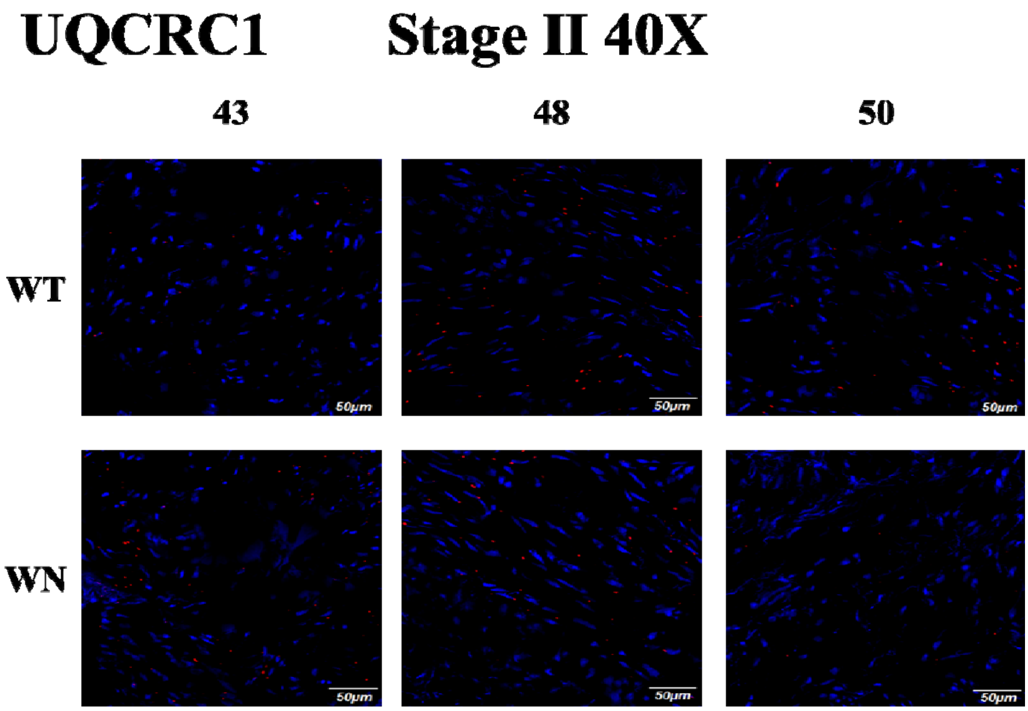
UQCRC1
Stage II 40X
43
48
50
WT
WN
50μm

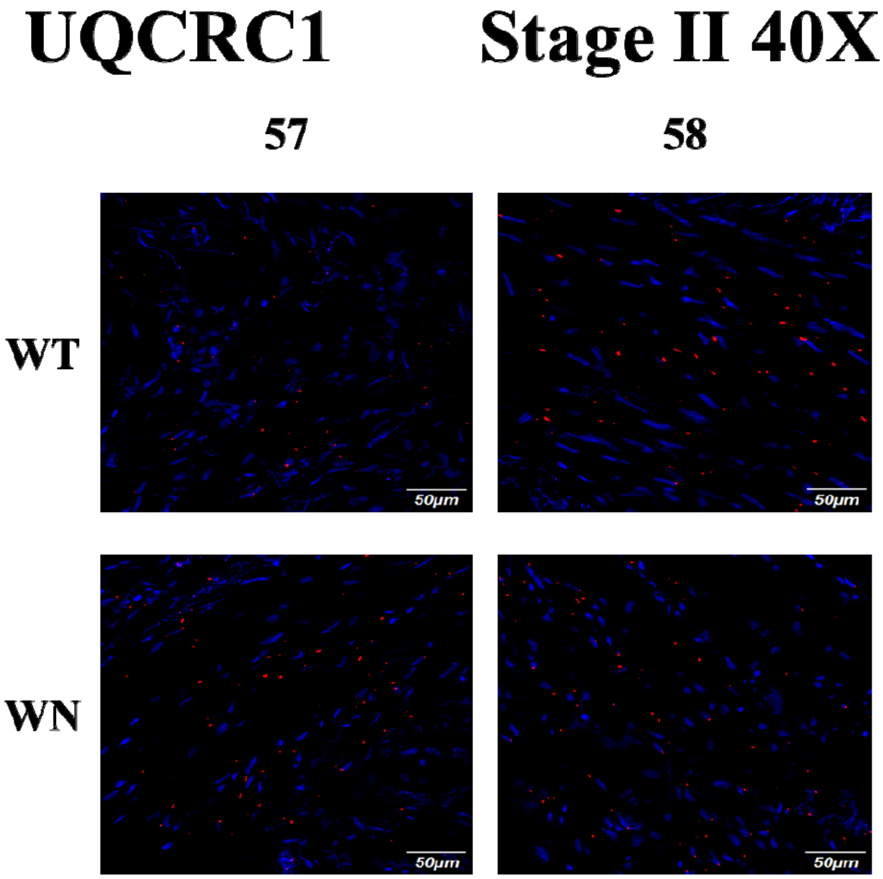
UQCRC1
Stage II 40X
57
58
WT
WN
50μm

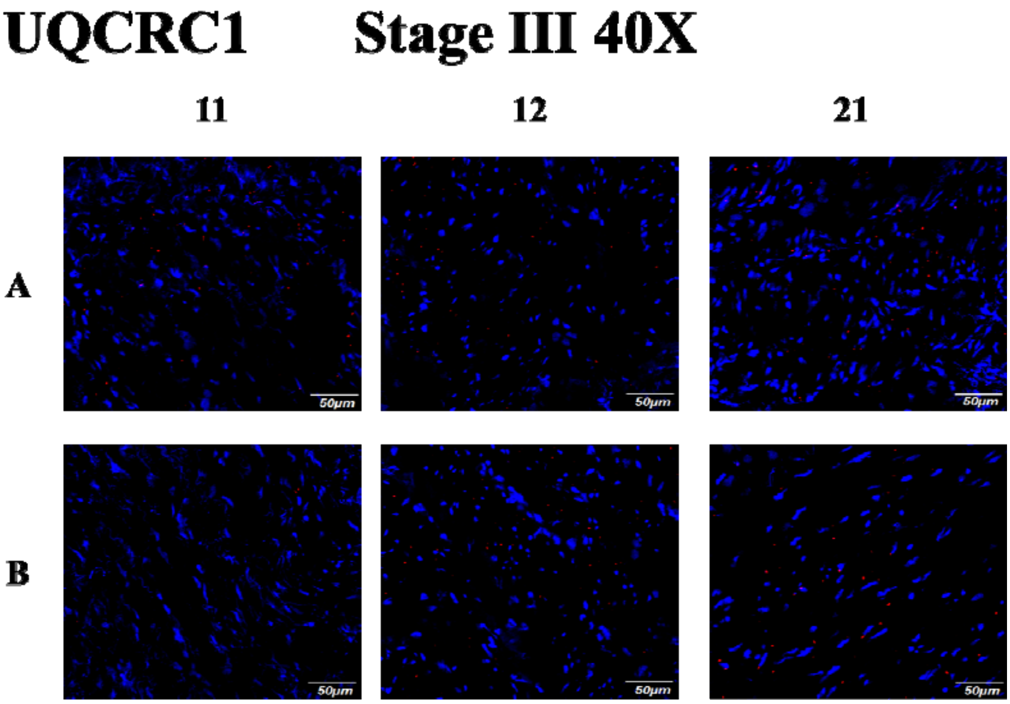
UQCRC1 Stage III 40X
11
12
21
A
B
50μm

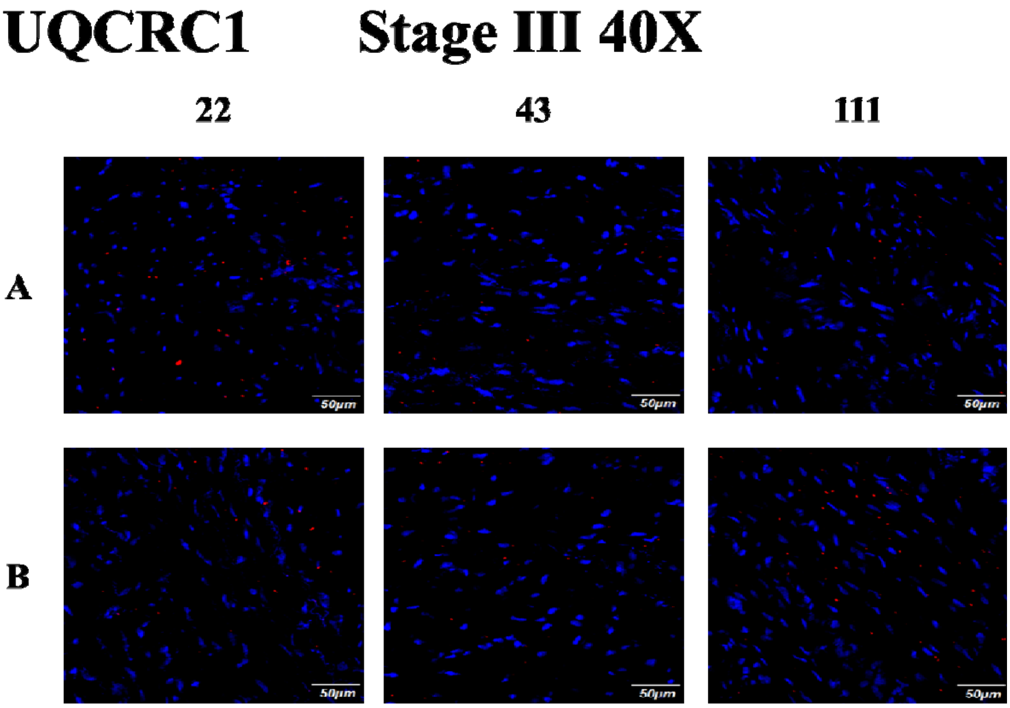
UQCRC1 Stage III 40X
22
43
111
A
B
50μm

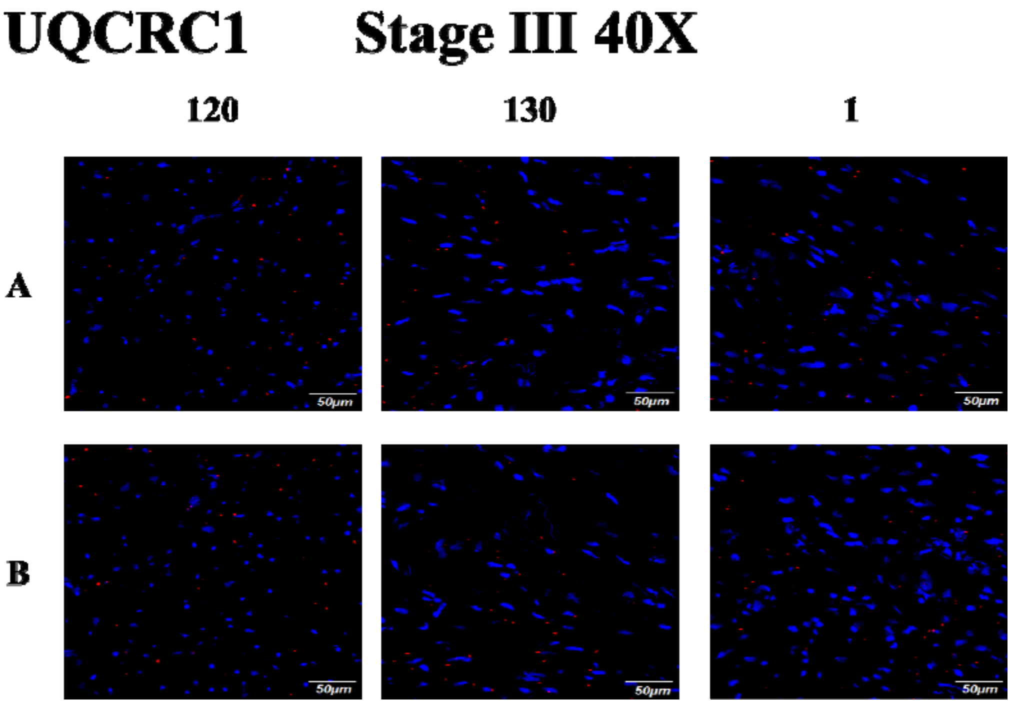
UQCRC1 Stage III 40X
120
130
1
A
B
50μm

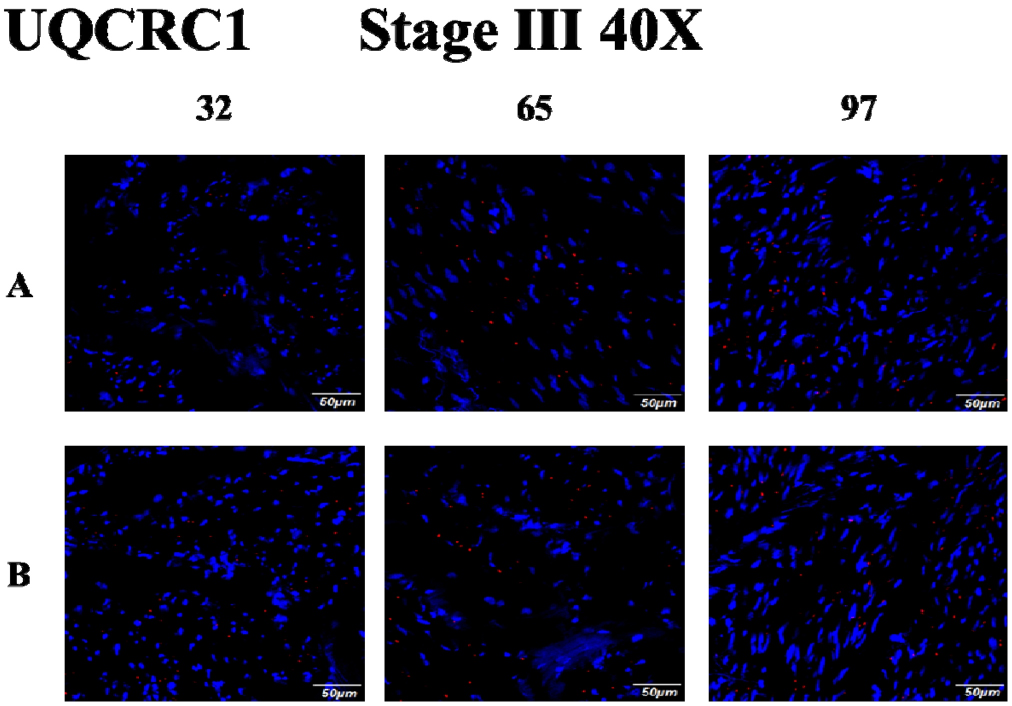
UQCRC1 Stage III 40X
32
65
97
A
B
50μm

UQCRC1 Stage III 40X

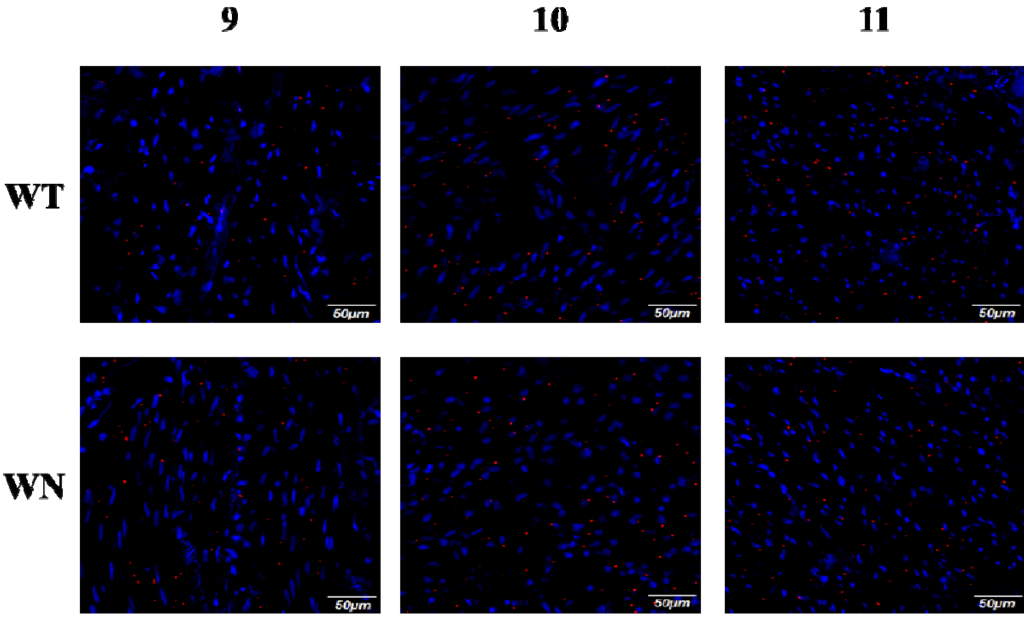

UQCRC1 Stage III 40X

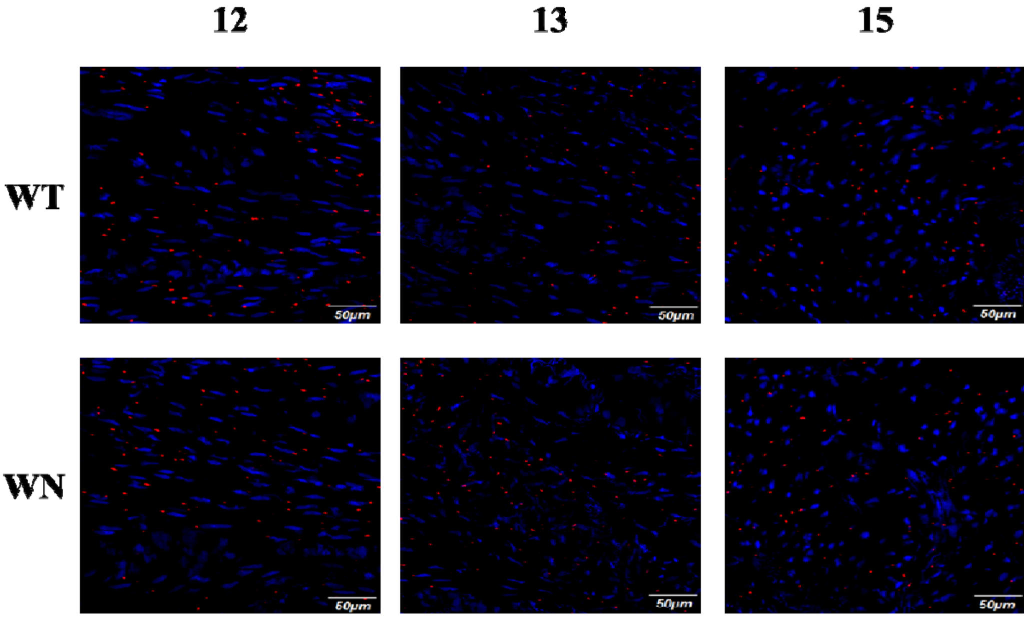

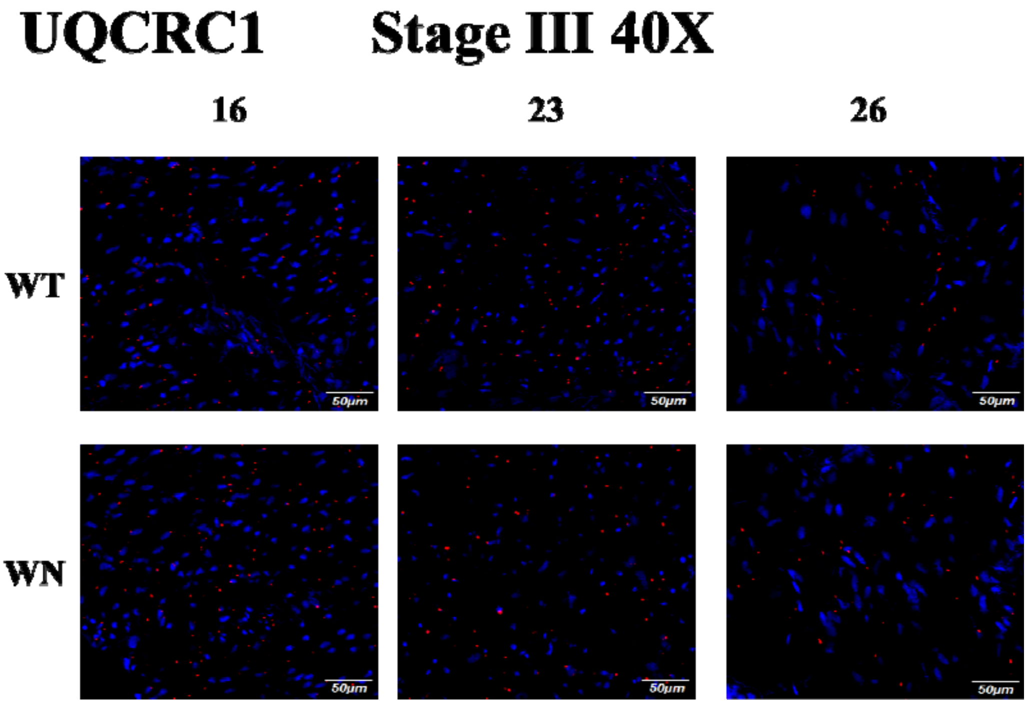
UQCRC1
Stage III 40X
16
23
26
WT
WN
50μm

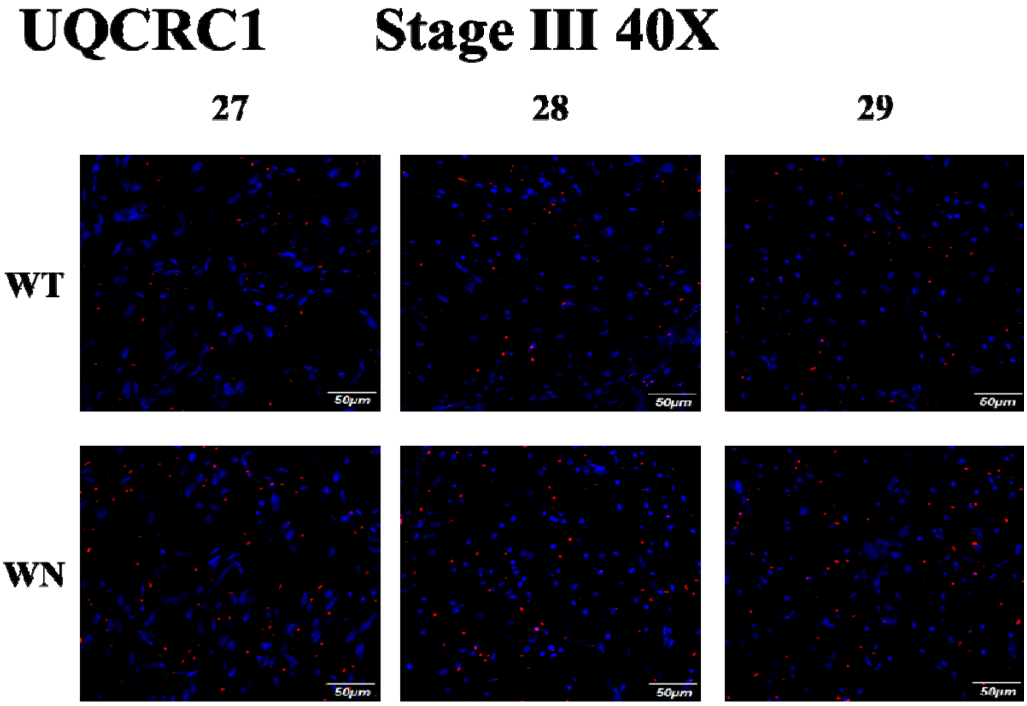
UQCRC1
Stage III 40X
27
28
29
WT
WN
50μm

UQCRC1 Stage III 40X

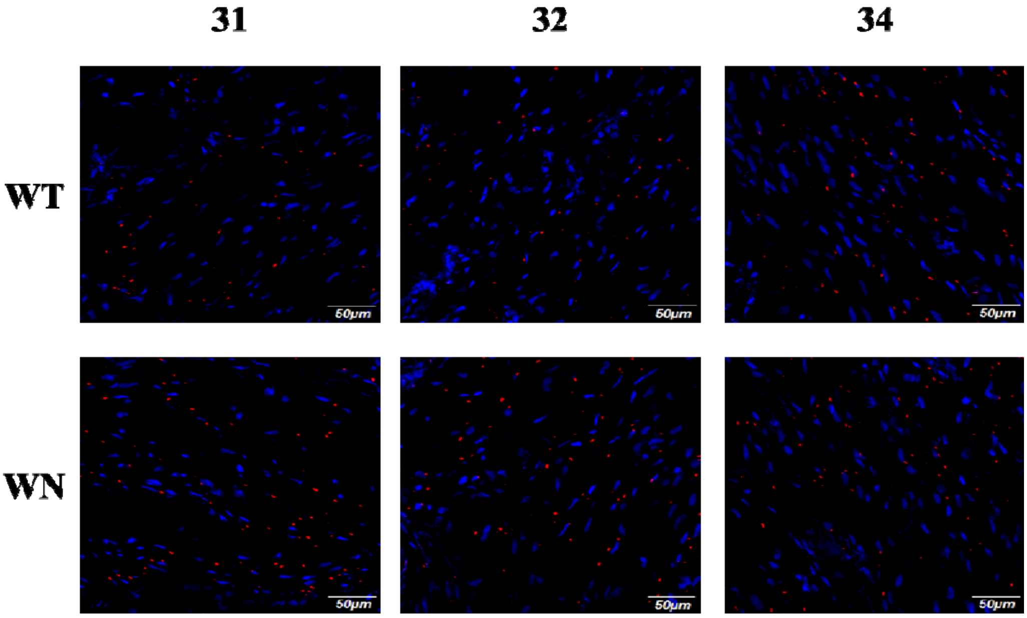

UQCRC1 Stage III 40X

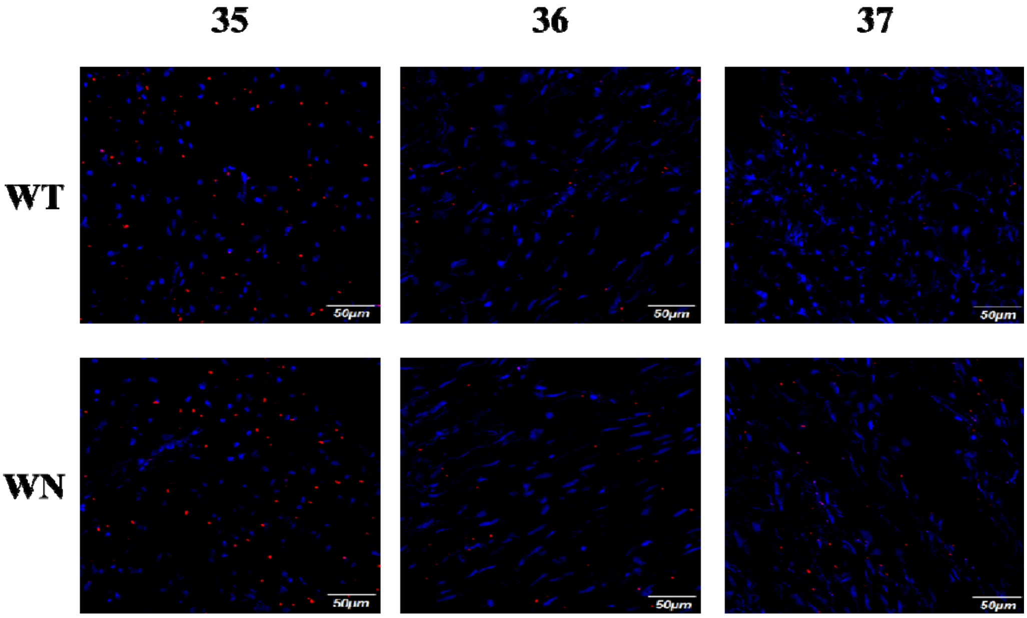

UQCRC1 Stage III 40X

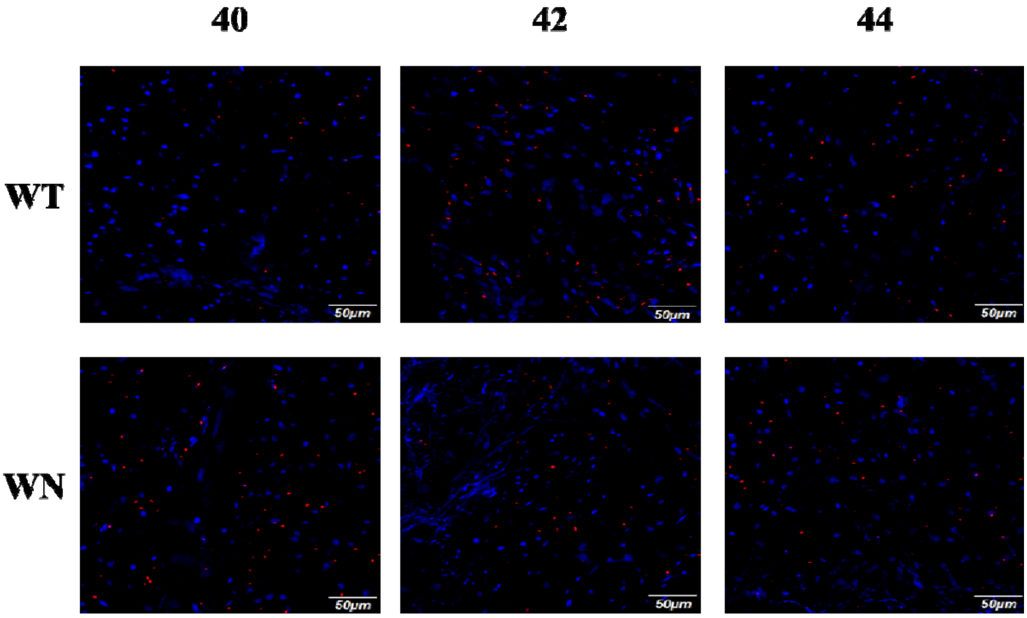

UQCRC1 Stage III 40X

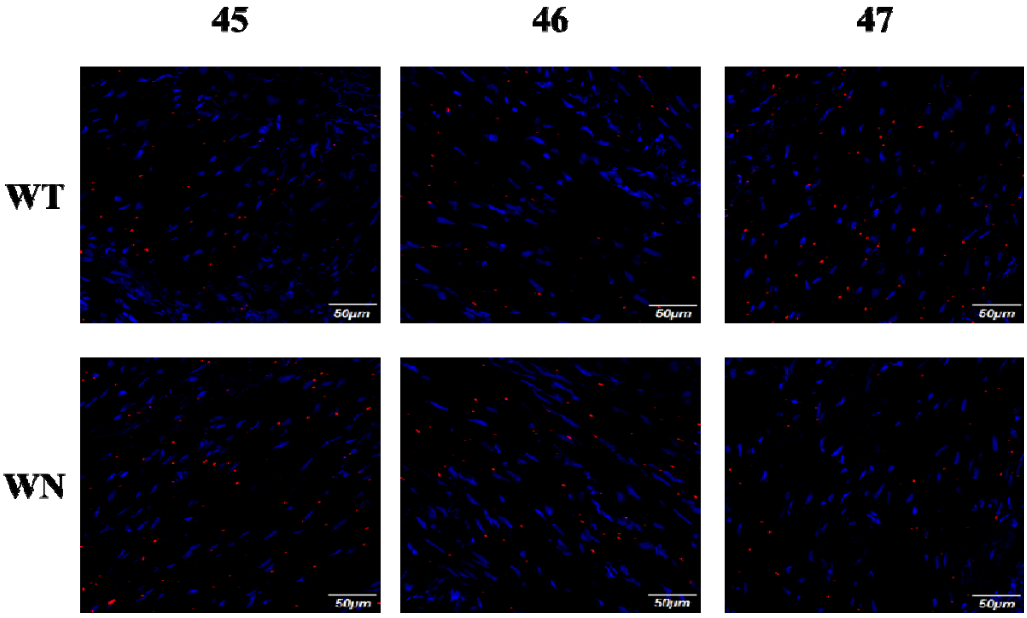

UQCRC1 Stage III 40X

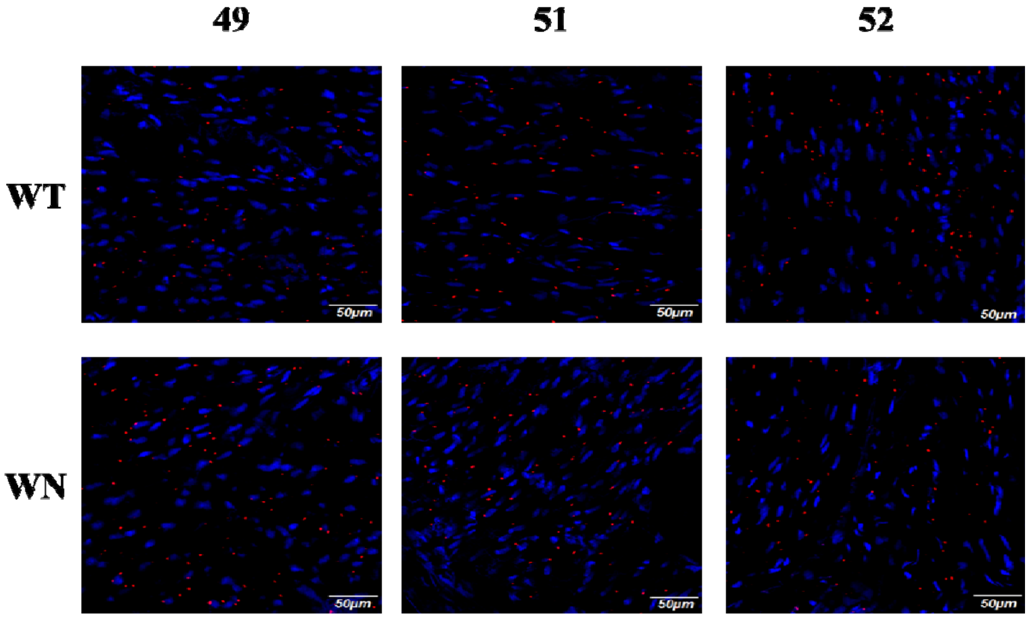

UQCRC1 Stage III 40X

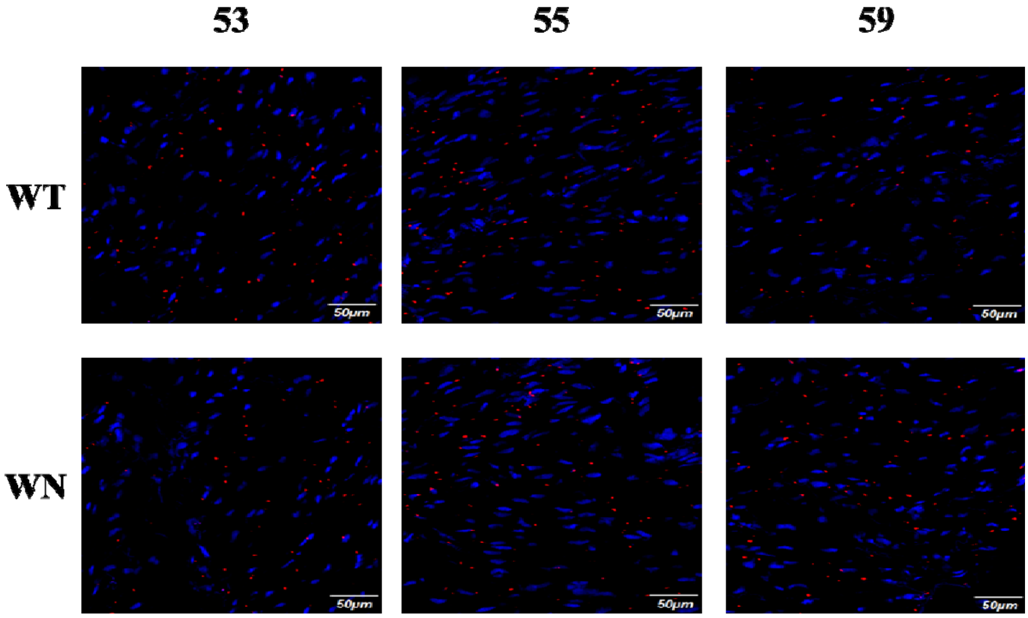

UQCRC1 Stage III 40X

60

WT

WN

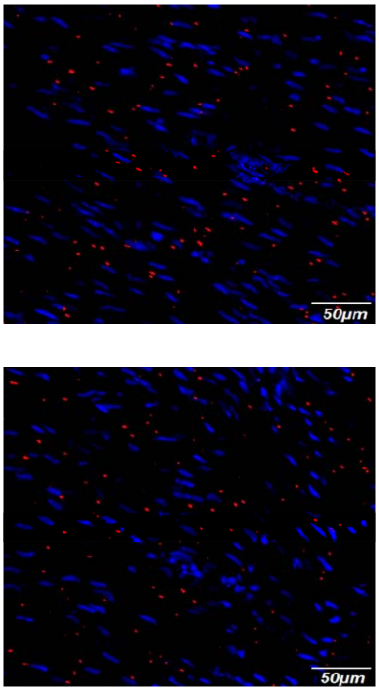

UQCRC1 Stage IV 40X

33 59 114

A

B

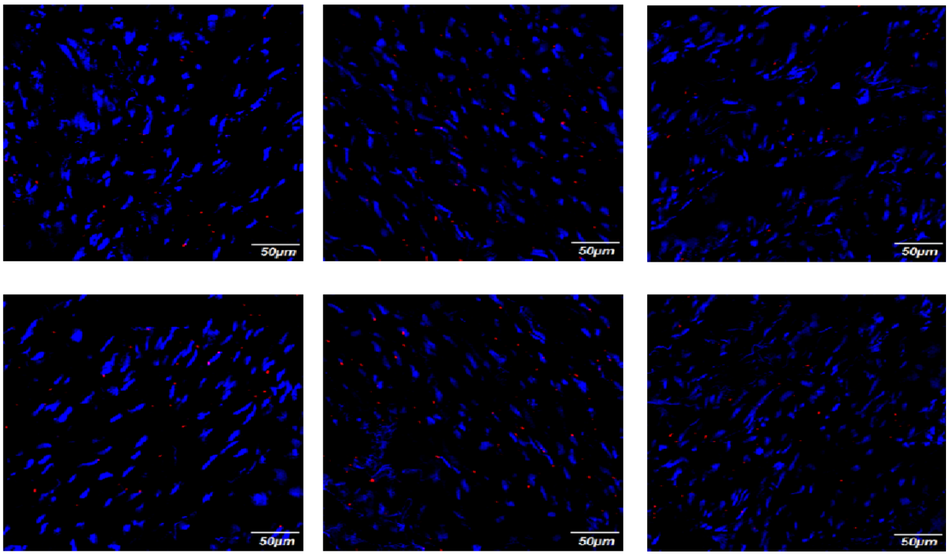

UQCRC1 Stage IV 40X

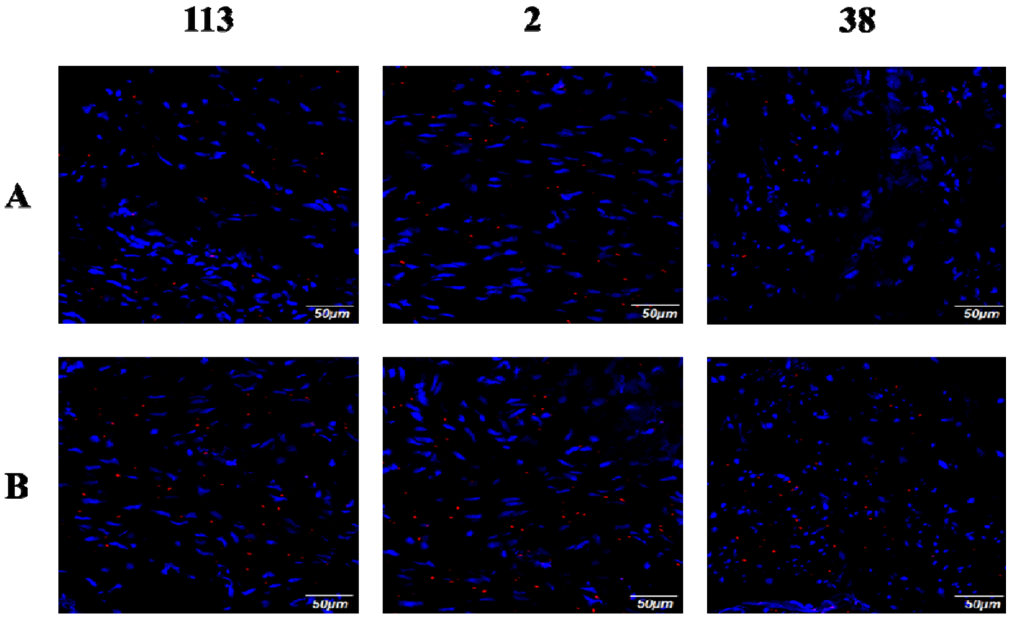

UQCRC1 Stage IV 40X

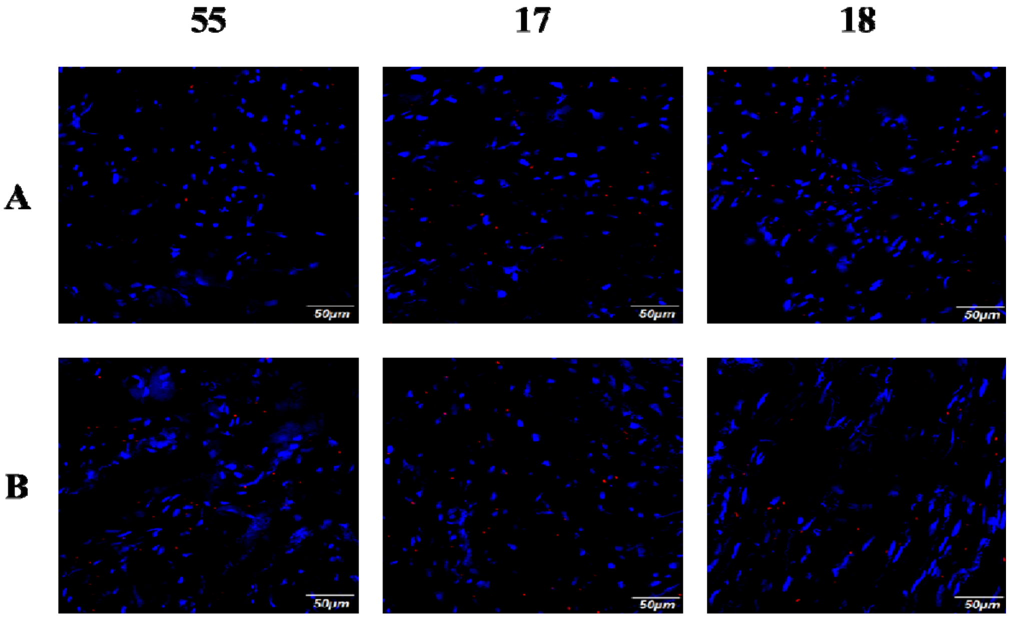

UQCRC1 Stage IV 40X

112

A

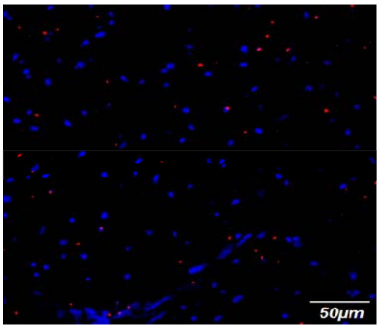

B

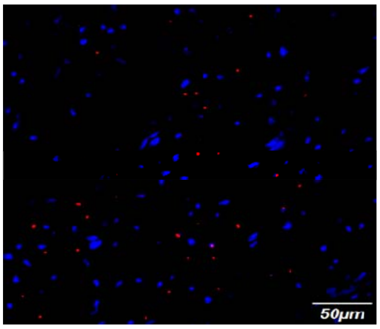

Supplementary Figure 3. Blocking experiment. The specificity of the antibodies was determined by preincubating the antibodies against ANXA1, NNMT, FBLN5 and UQCRC1 with their corresponding recombinant proteins before adding to tissue sections. Ab: frozen sections were incubated with specific antibodies; BL: antibodies were preincubated with corresponding recombinant proteins for 4 hours at room temperature.

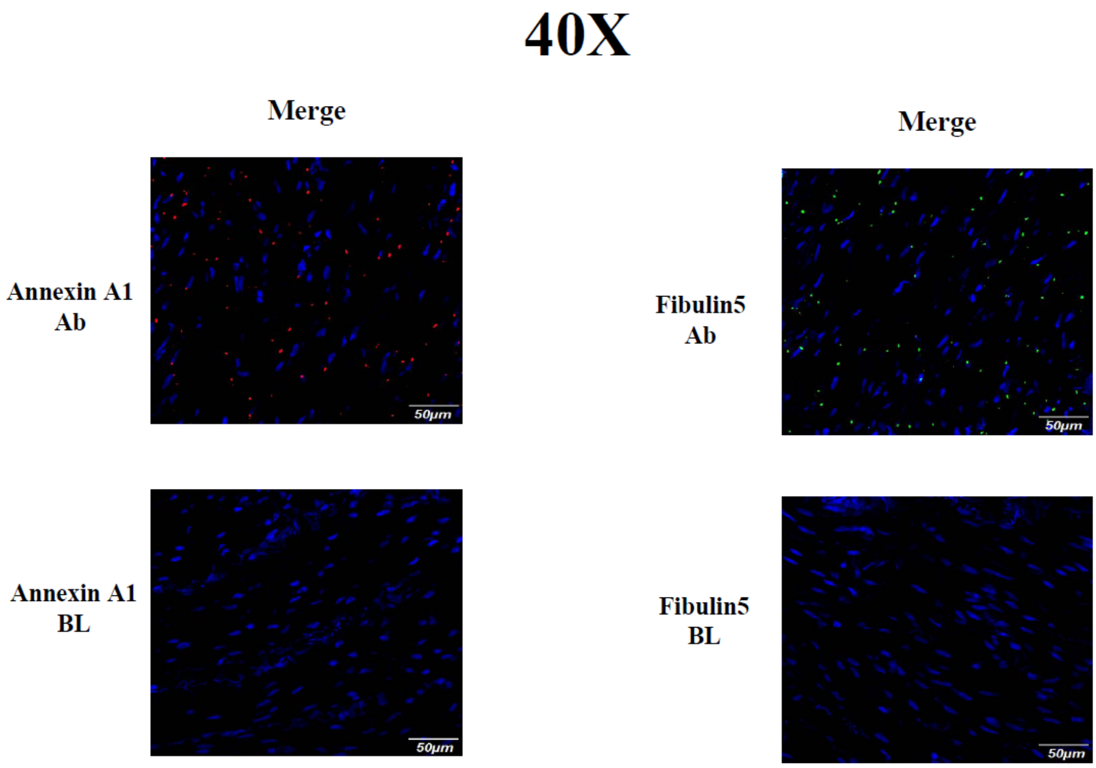

40X

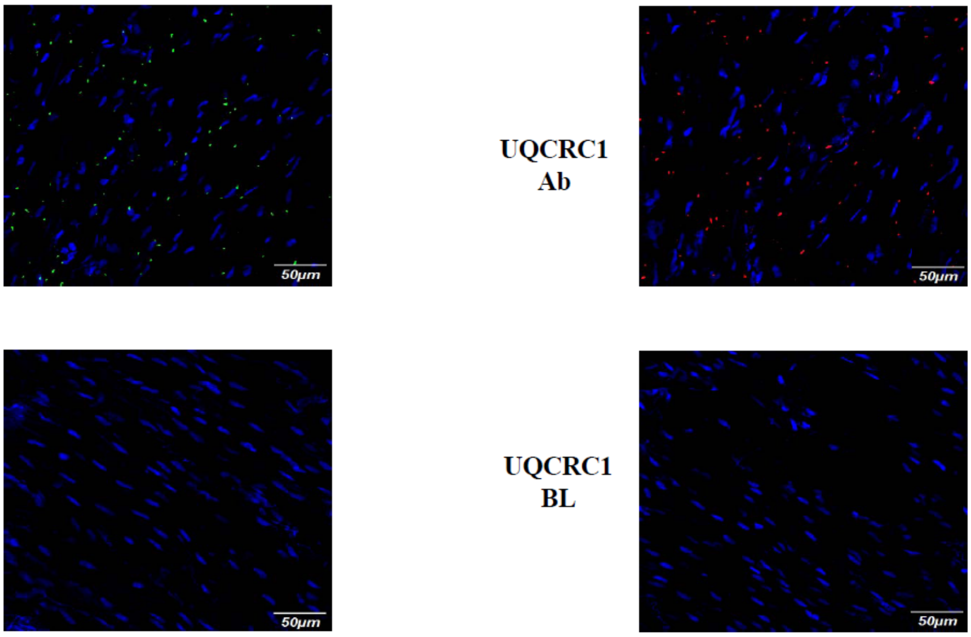

Merge | Merge

NNMT Ab | UQCRC1 Ab

NNMT BL | UQCRC1 BL

Supplementary Figure 4. Uncropped images of Western Blot for all the panels shown in Figures 4, 5, 6 and 15.

AnnexinA1，125A 125B

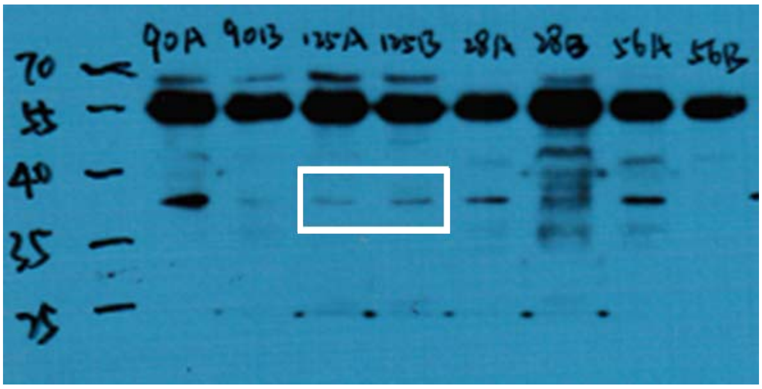

AnnexinA1-GAPDH，125A 125B

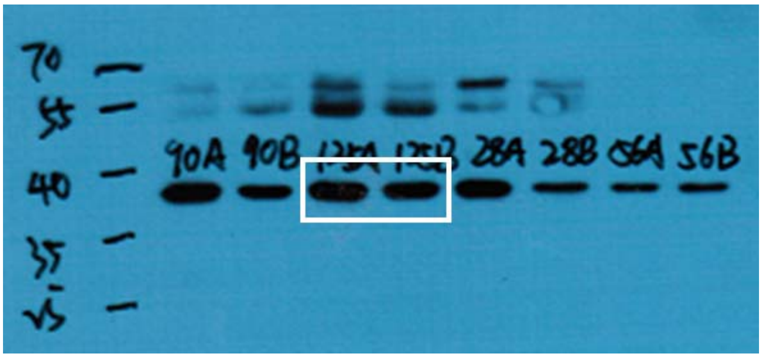

AnnexinA1，84A 84B

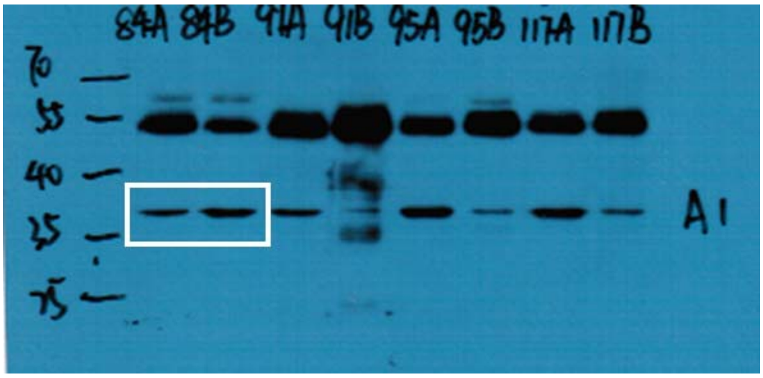

AnnexinA1-GAPDH，84A 84B

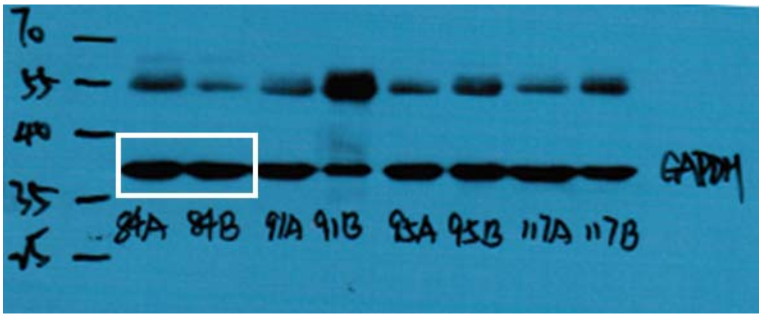

AnnexinA1，W2T W2N

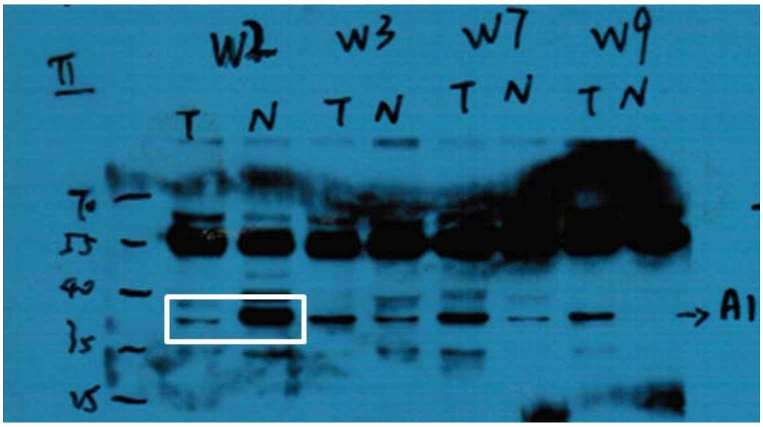

AnnexinA1-GAPDH，W2T W2N

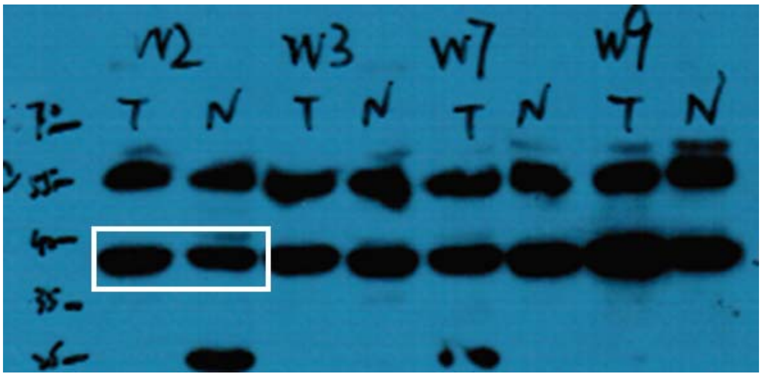

AnnexinA1，W8T W8N

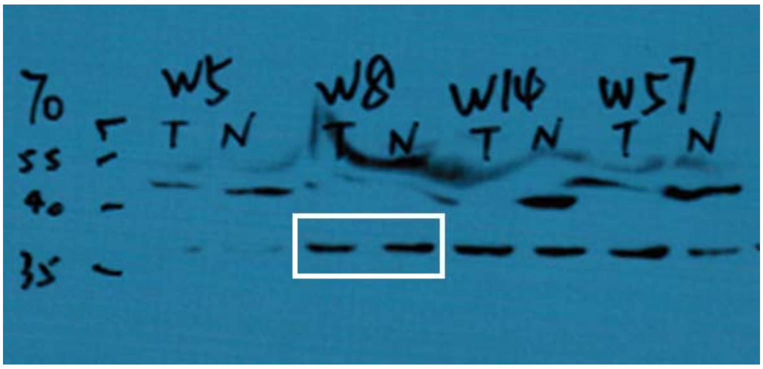

AnnexinA1-GAPDH，W8T W8N

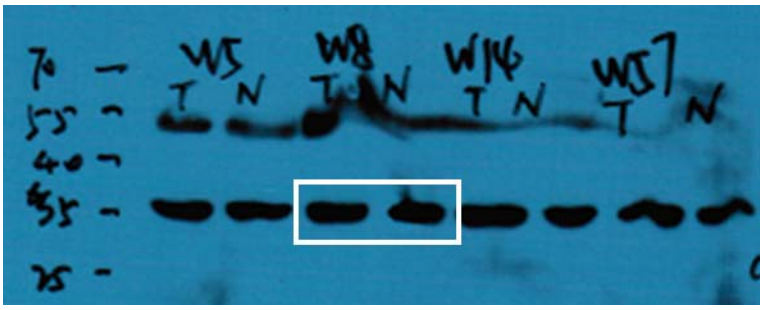

AnnexinA1，W46T W46N

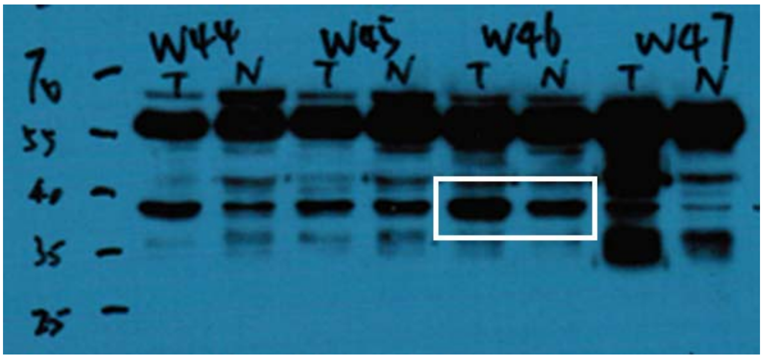

AnnexinA1-GAPDH，W46T W46N

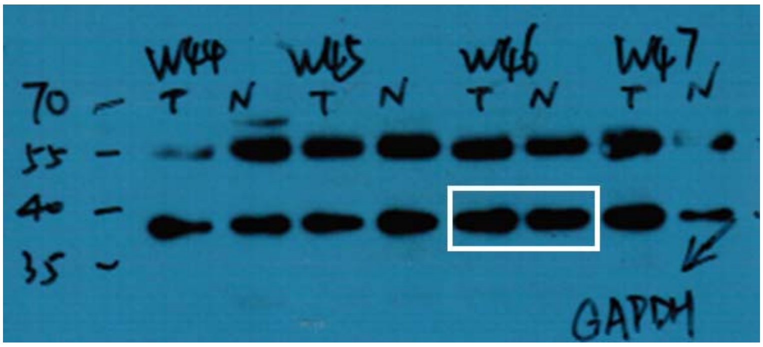

AnnexinA1，W49T W49N

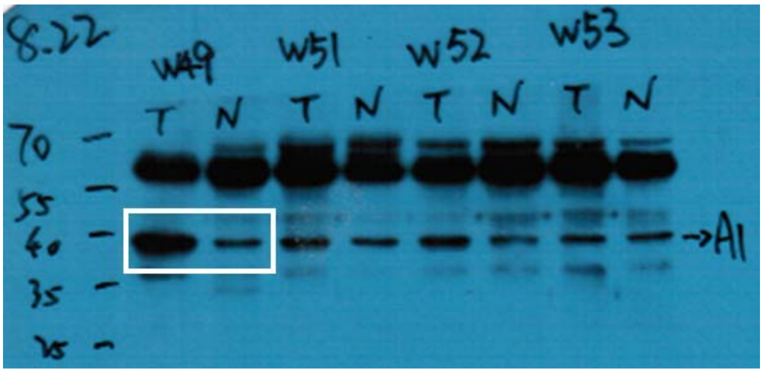

AnnexinA1-GAPDH，W49T W49N

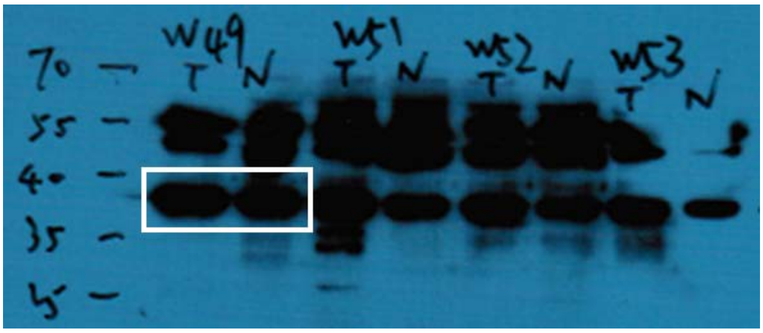

AnnexinA1，17A 17B

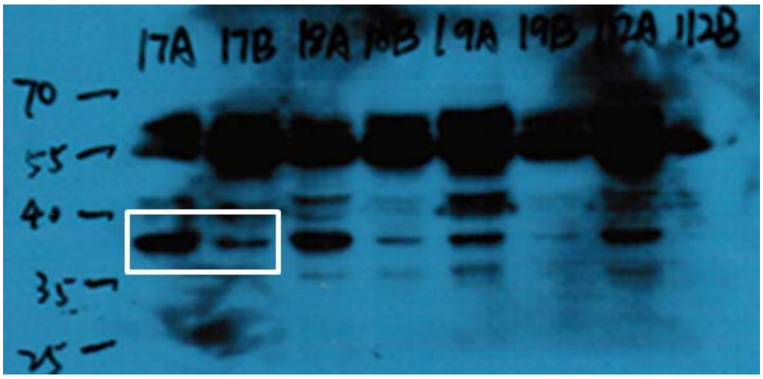

AnnexinA1-GAPDH，17A 17B

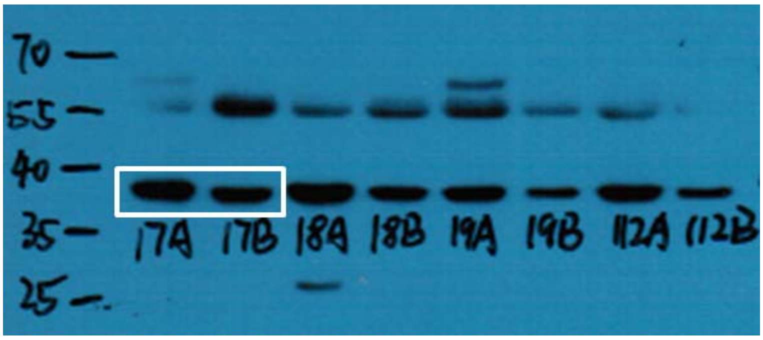

AnnexinA1，18A 18B

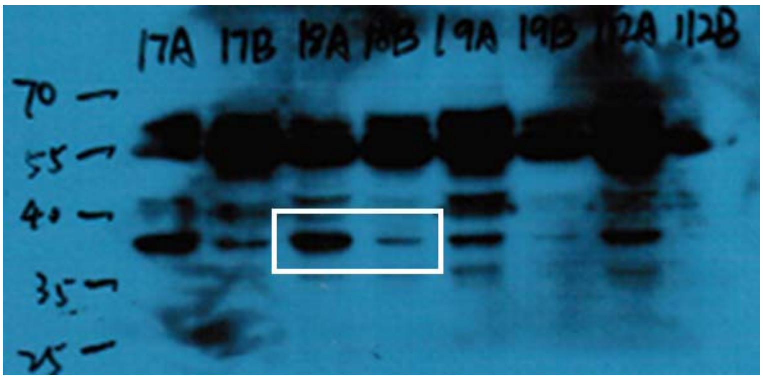

AnnexinA1-GAPDH，18A 18B

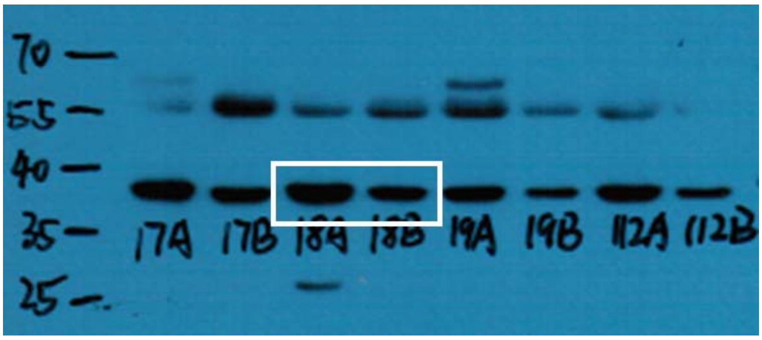

NNMT，95A 95B

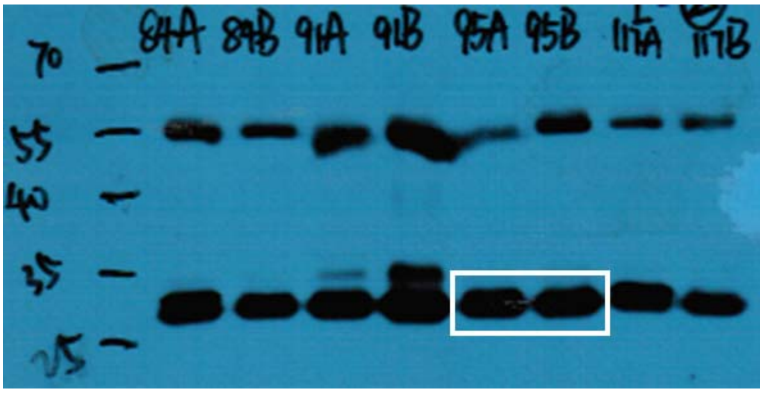

NNMT-GAPDH，95A 95B

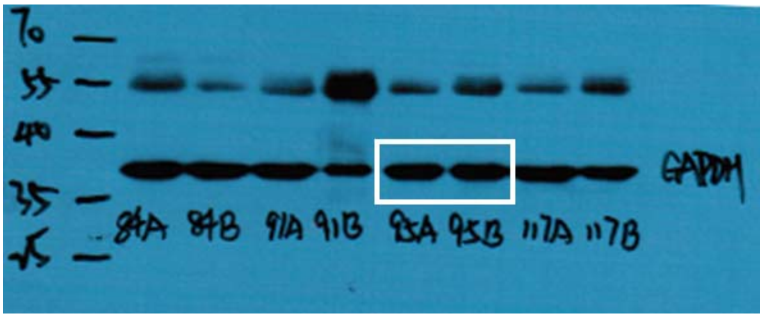

NNMT，125A 125B

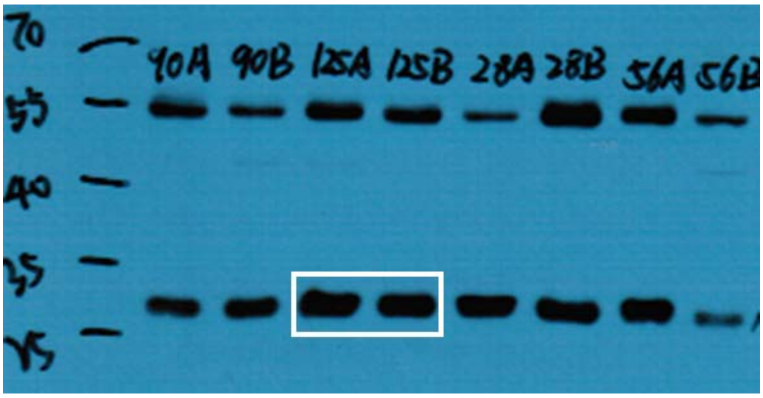

NNMT-GAPDH，125A 125B

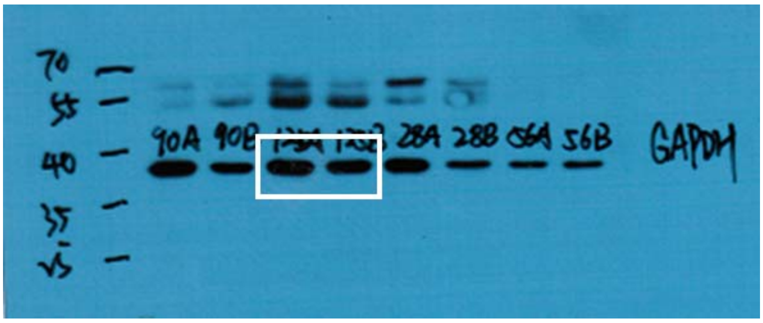

NNMT，W2T W2N

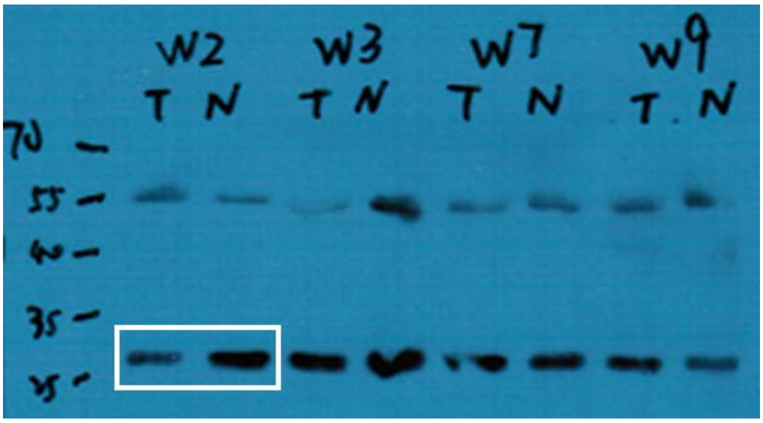

NNMT-GAPDH，W2T W2N

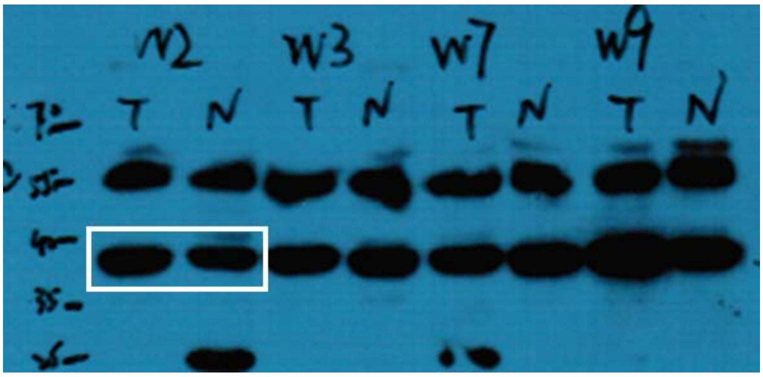

NNMT，W18T W18N

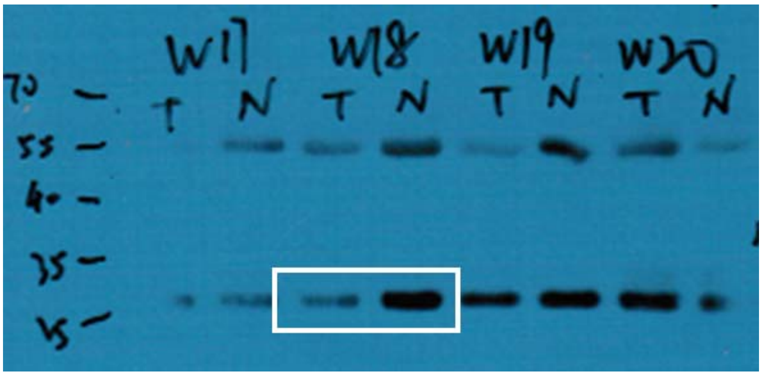

NNMT-GAPDH，W18T W18N

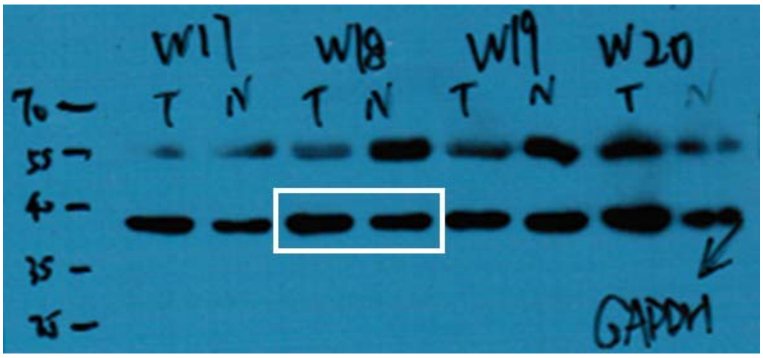

NNMT，12A 12B

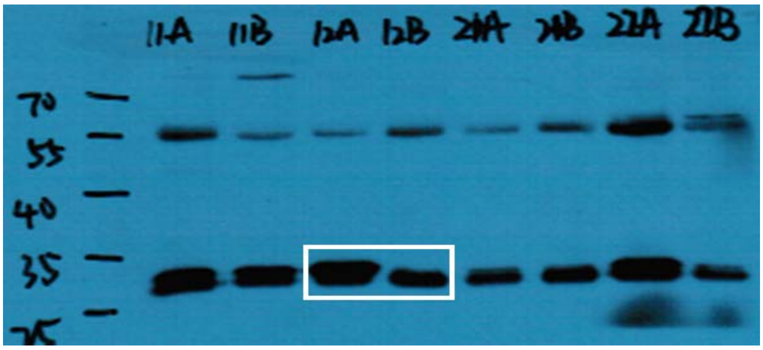

NNMT-GAPDH，12A 12B

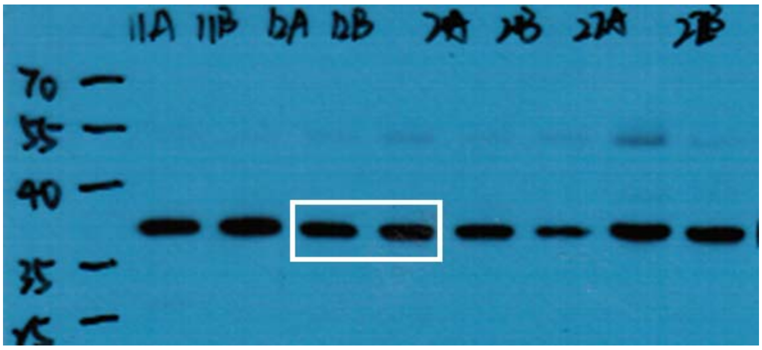

NNMT，97A 97B

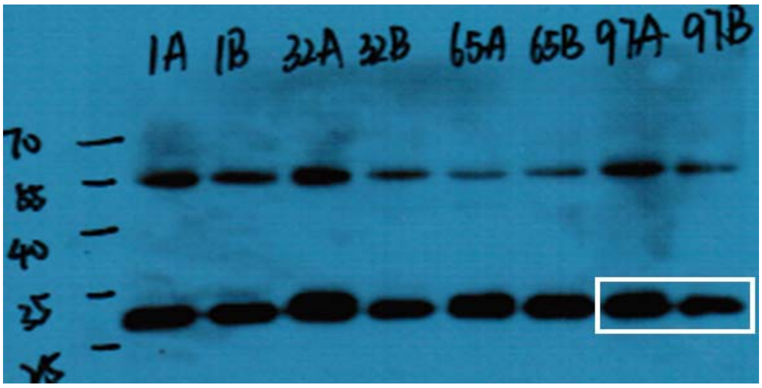

NNMT-GAPDH，97A 97B

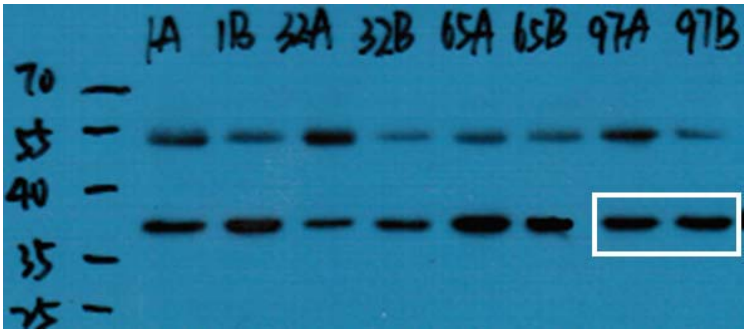

NNMT，18A 18B

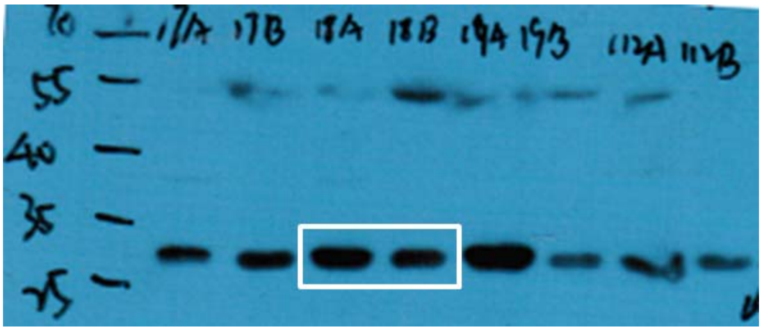

NNMT-GAPDH，18A 18B

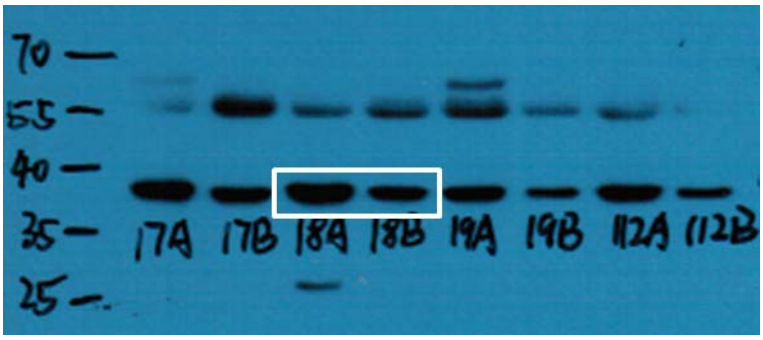

NNMT，38A 38B

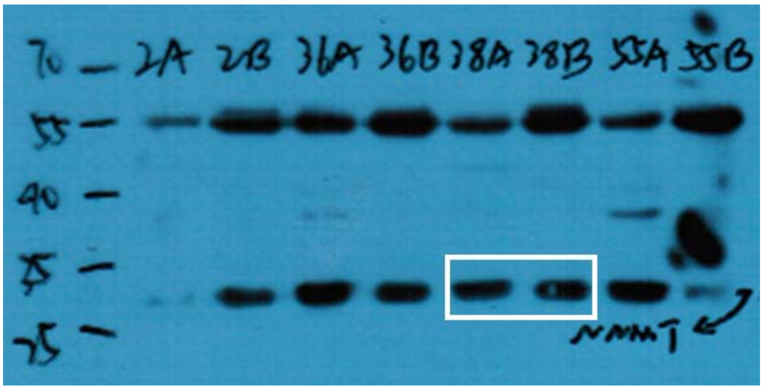

NNMT-GAPDH，38A 38B

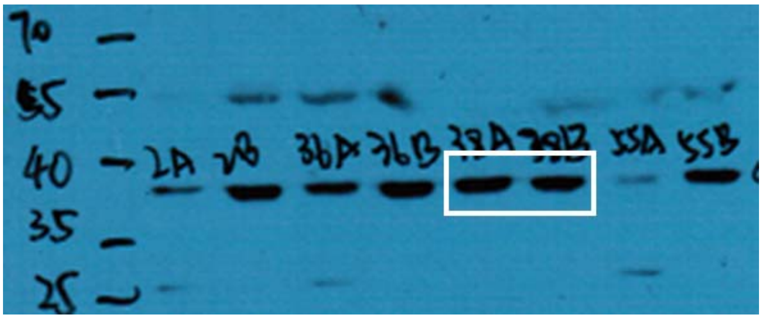

Fibulin5，35A 35B

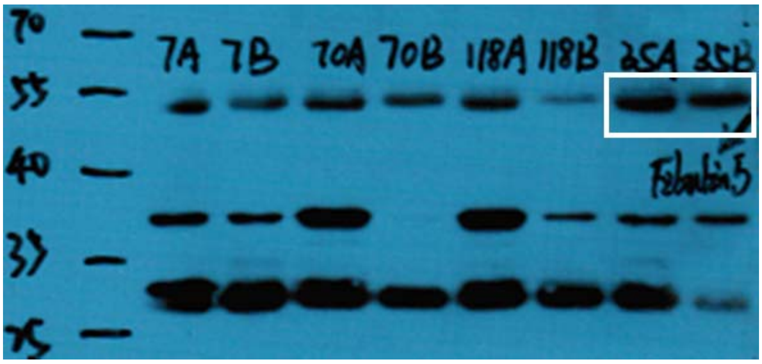

Fibulin5-GAPDH，35A 35B

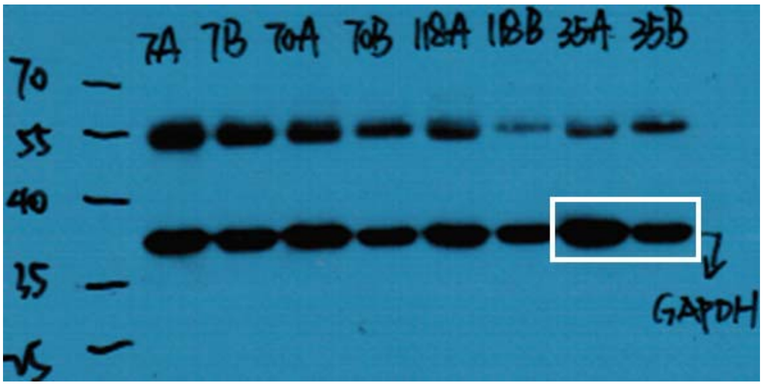

Fibulin5，W54T W54N

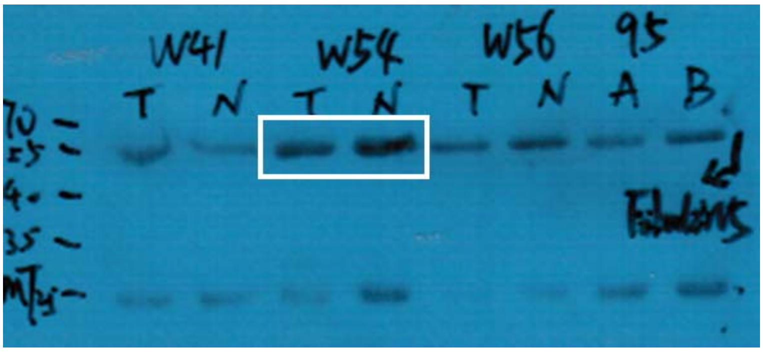

Fibulin5-GAPDH，W54T W54N

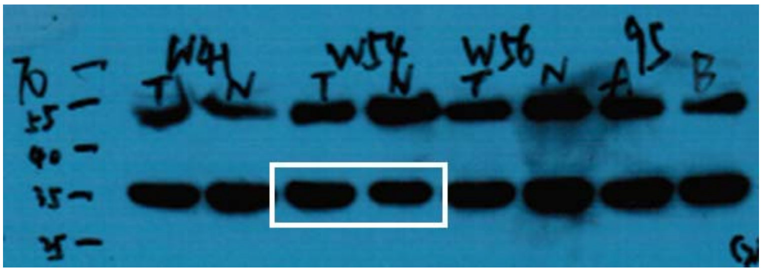

Fibulin5，109A 109B

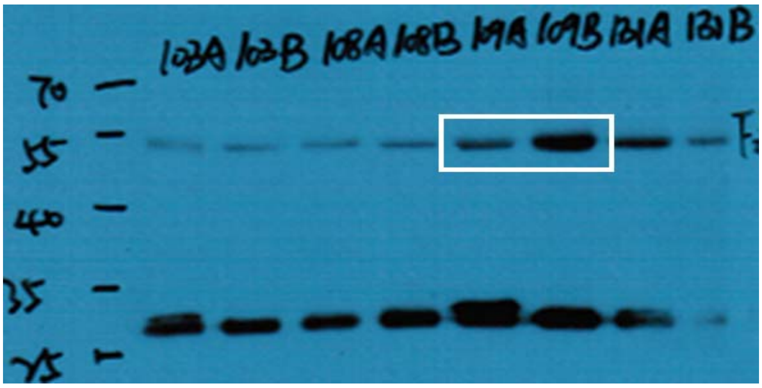

Fibulin5-GAPDH，109A 109B

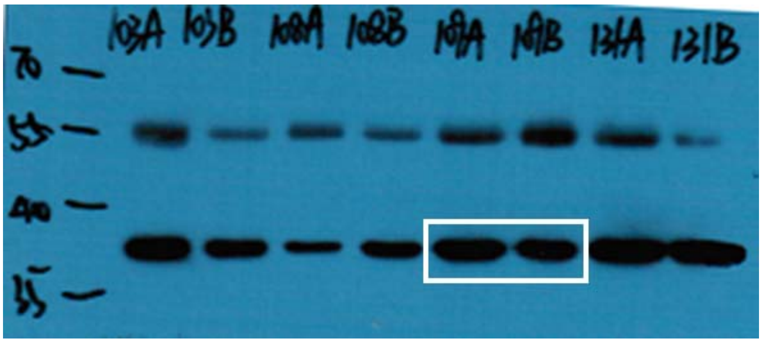

Fibulin5，W18T W18N

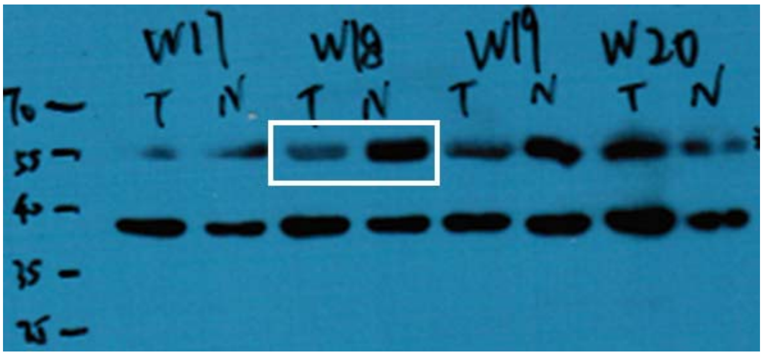

Fibulin5-GAPDH，W18T W18N

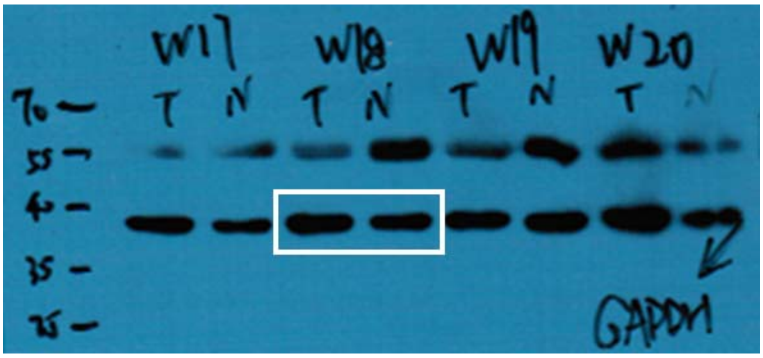

Fibulin5，W36T W36N

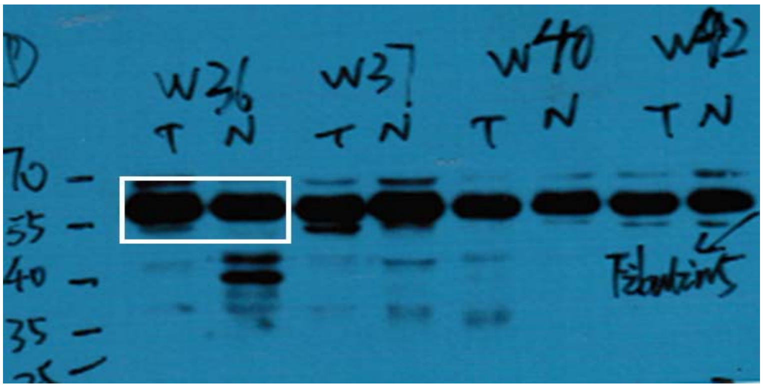

Fibulin5-GAPDH，W36T W36N

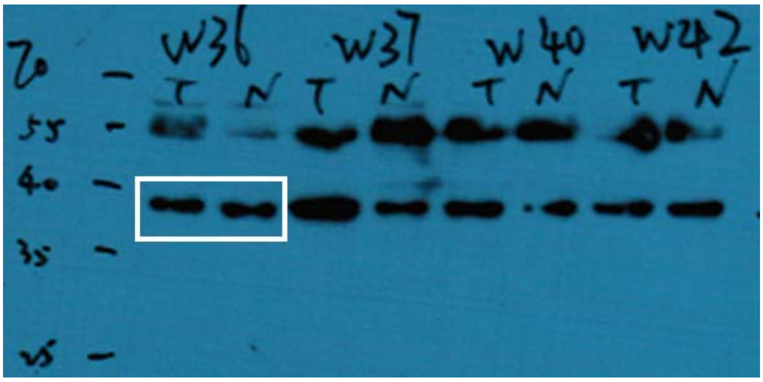

Fibulin5，W53T W53N

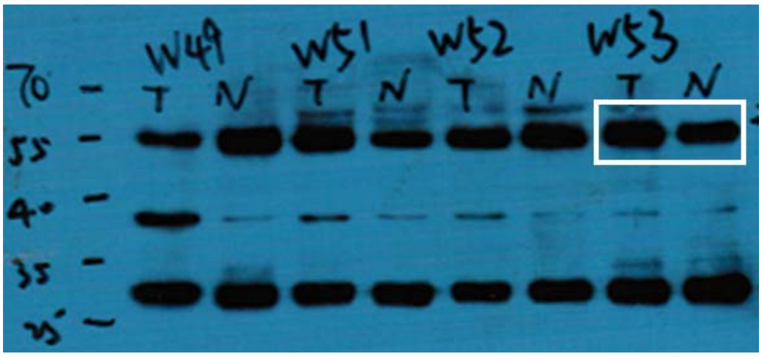

Fibulin5-GAPDH，W53T W53N

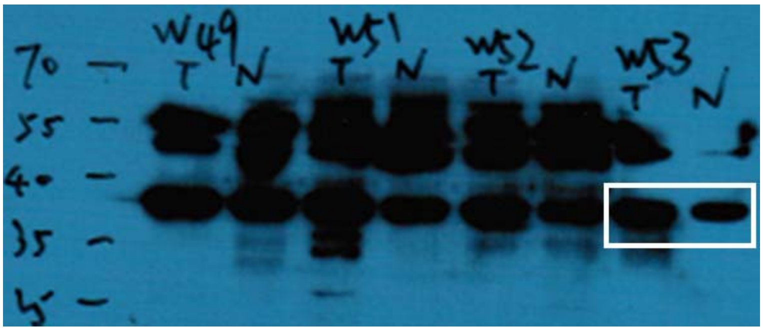

Fibulin5，18A 18B

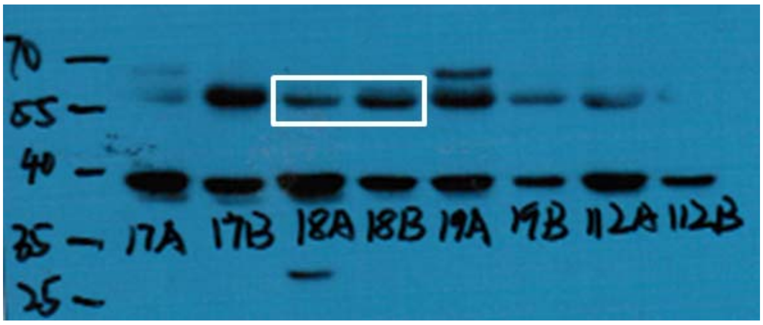

Fibulin5-GAPDH，18A 18B

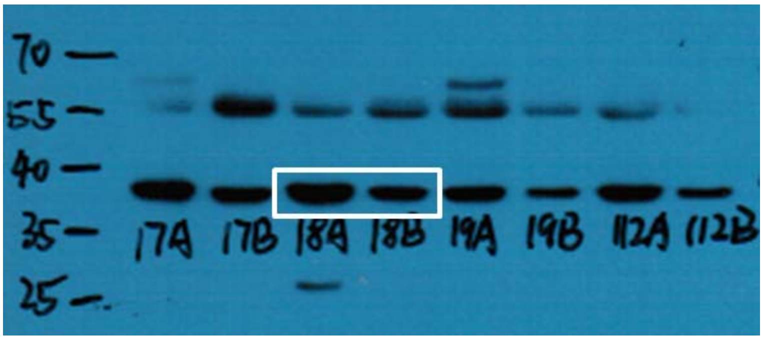

Fibulin5，112A 112B

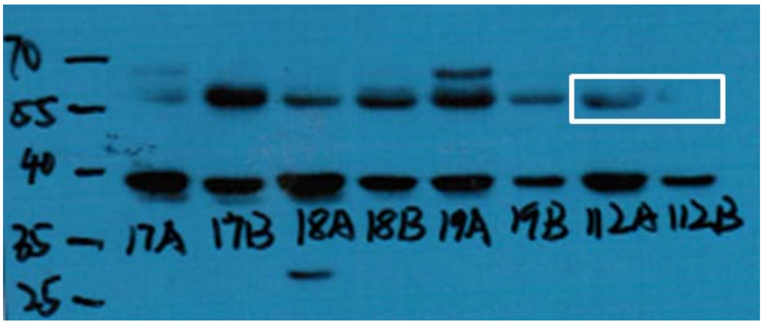

Fibulin5-GAPDH，112A 112B

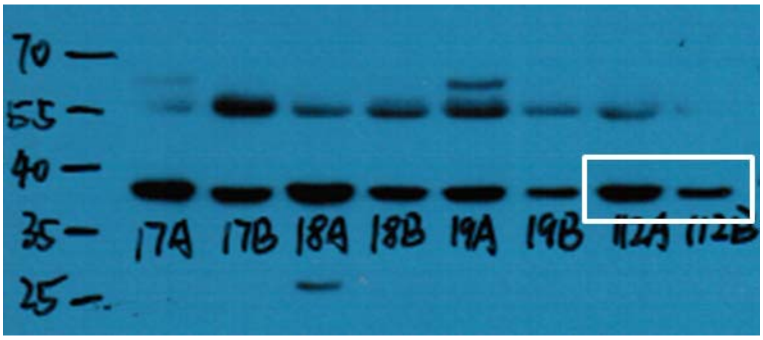

UQCRC1，W41T W41N

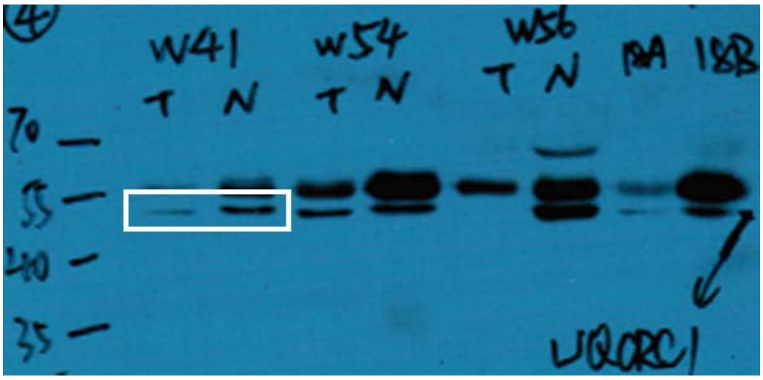

UQCRC1-GAPDH，W41T W41N

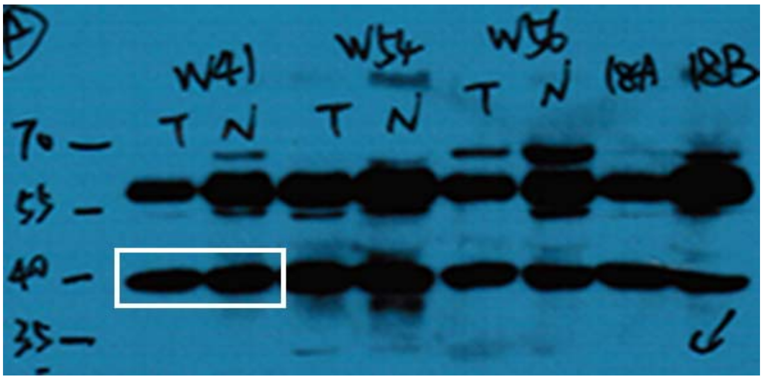

UQCRC1，W54T W54N

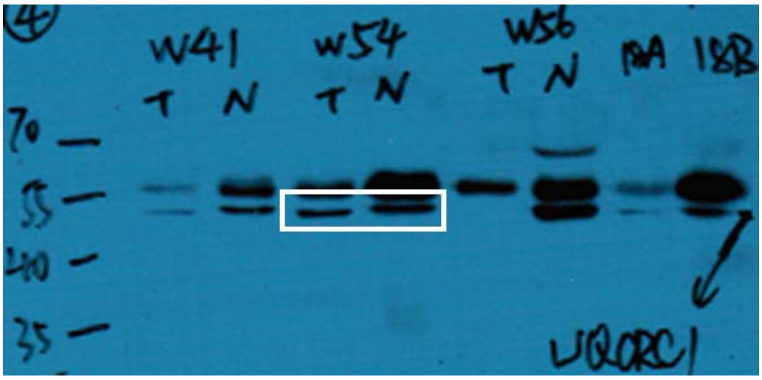

UQCRC1-GAPDH，W54T W54N

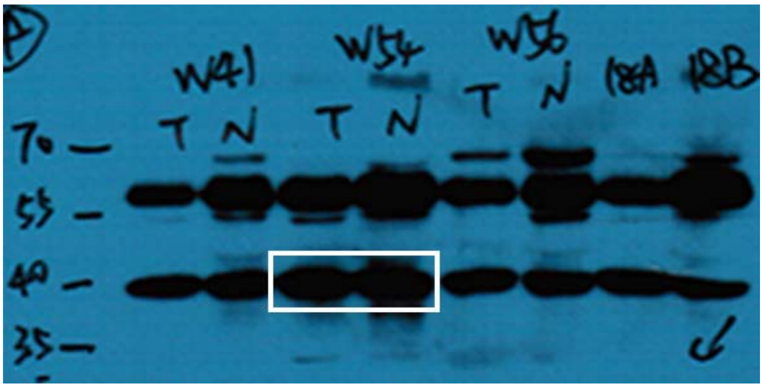

UQCRC1，W22T W22N

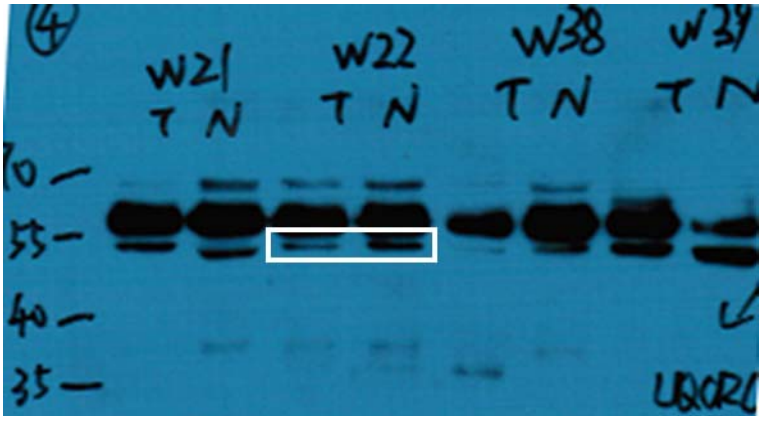

UQCRC1-GAPDH，W22T W22N

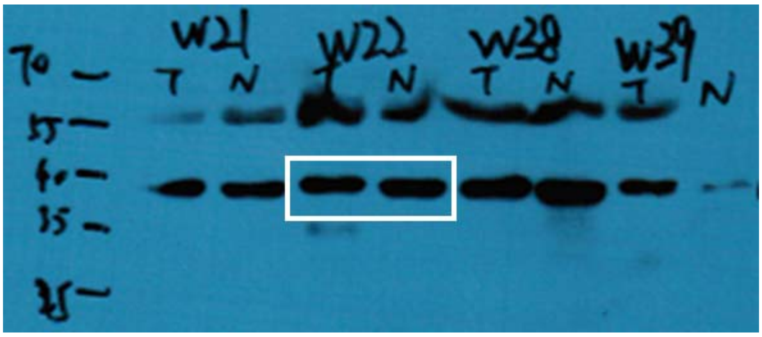

UQCRC1，W38T W38N

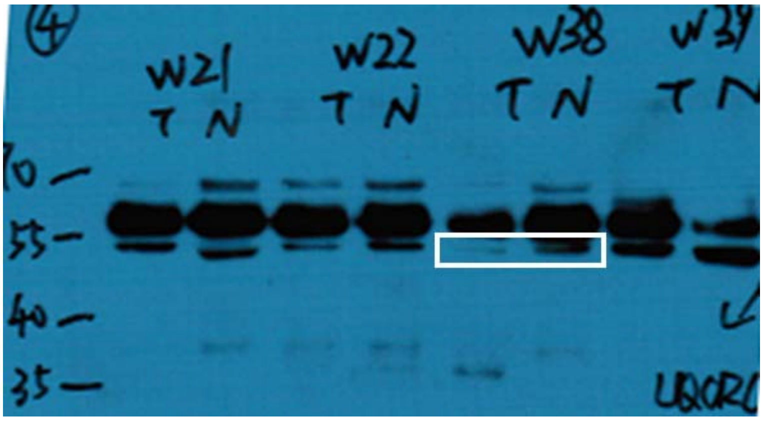

UQCRC1-GAPDH，W38T W38N

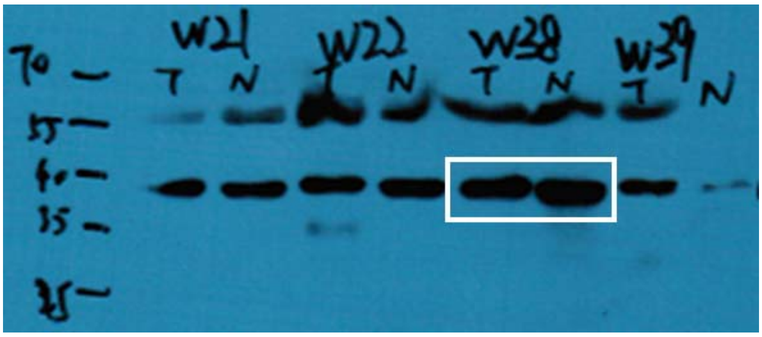

UQCRC1，12A 12B

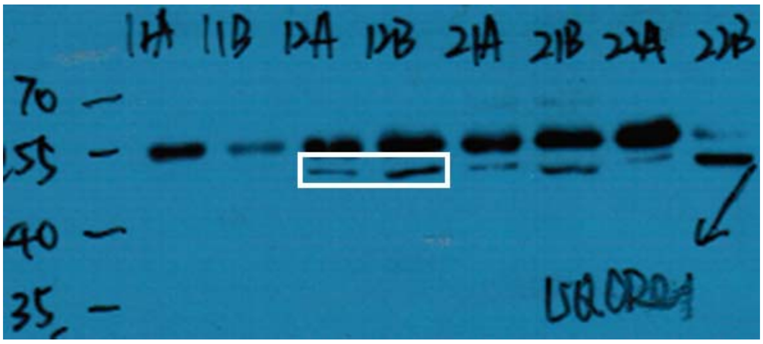

UQCRC1-GAPDH，12A 12B

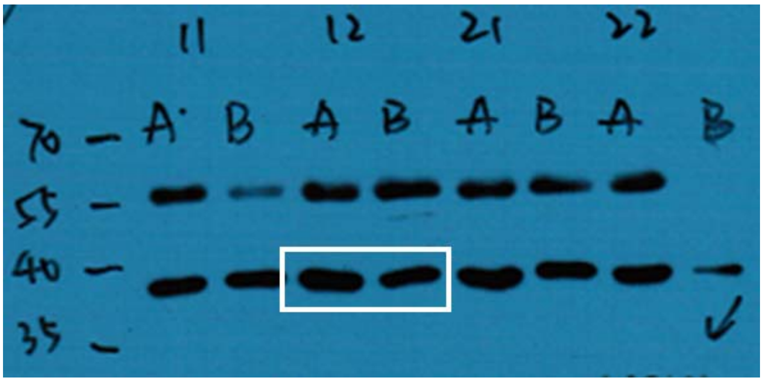

UQCRC1，21A 21B

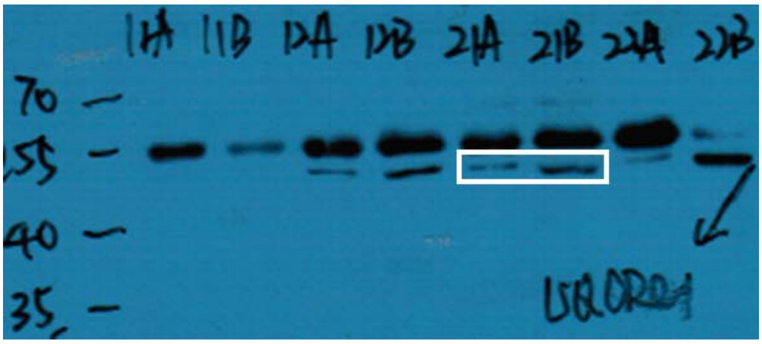

UQCRC1-GAPDH，21A 21B

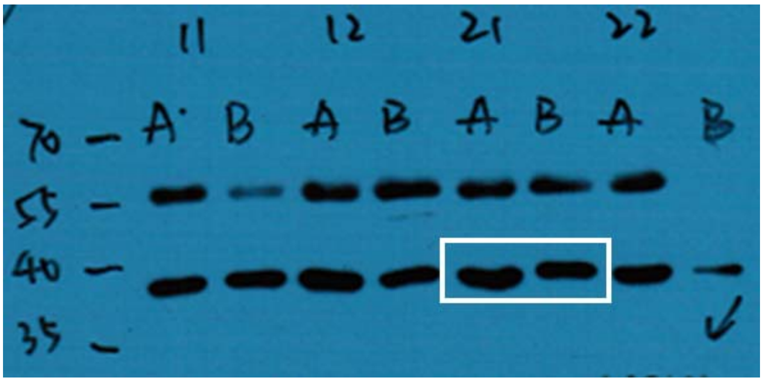

UQCRC1，59A 59B

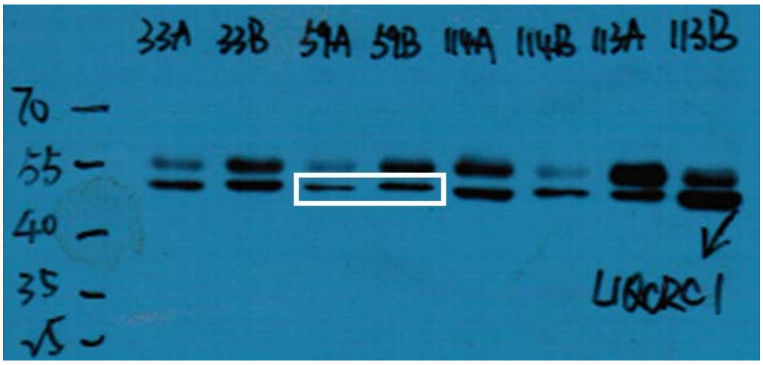

UQCRC1-GAPDH，59A 59B

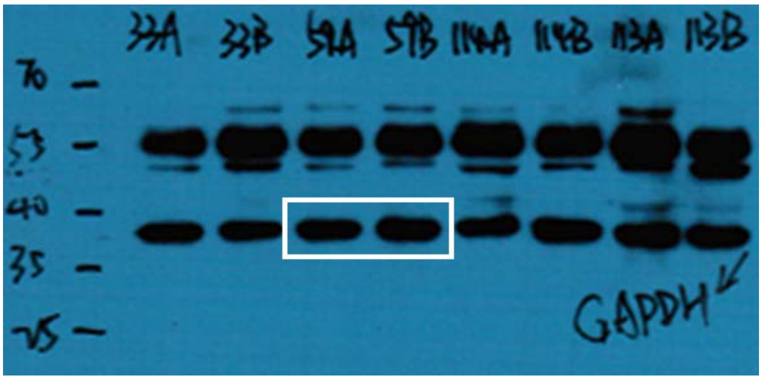

UQCRC1，113A 113B

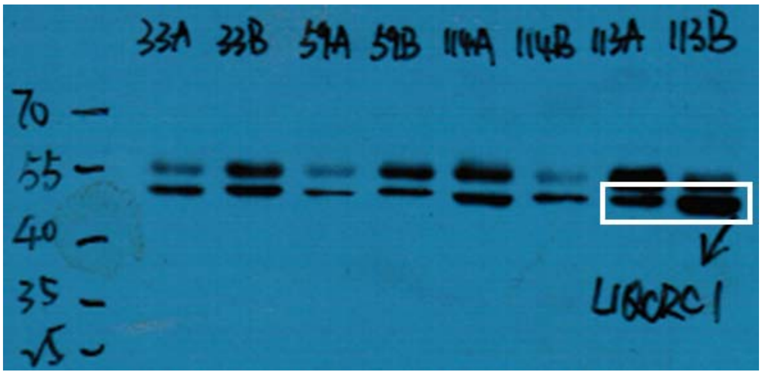

UQCRC1-GAPDH，113A 113B

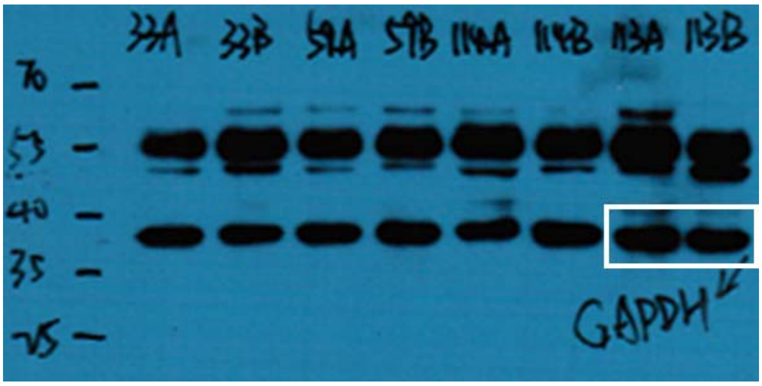

Supplement: Supplementary Figure [file srep38871-s1.pdf]
